# Repair-Assisted Damage Detection Reveals Biological Disparities in Prostate Cancer between African Americans and European Americans

**DOI:** 10.3390/cancers14041012

**Published:** 2022-02-17

**Authors:** Kimiko L. Krieger, Jie H. Gohlke, Kevin J. Lee, Danthasinghe Waduge Badrajee Piyarathna, Patricia D. Castro, Jeffrey A. Jones, Michael M. Ittmann, Natalie R. Gassman, Arun Sreekumar

**Affiliations:** 1 Department of Molecular and Cellular Biology, Baylor College of Medicine, Houston, TX 77030, USA; kimiko.krieger@bcm.edu (K.L.K.); jie.gohlke@bcm.edu (J.H.G.); danthasinghewaduge.piyarathna@bcm.edu (D.W.B.P.); 2 Center for Translational Metabolism and Health Disparities (C-TMH), Baylor College of Medicine, Houston, TX 77030, USA; 3 Mitchell Cancer Institute, University of South Alabama, Mobile, AL 36604, USA; kjlee@health.southalabama.edu; 4 Department of Pathology and Immunology, Baylor College of Medicine, Houston, TX 77030, USA; pcastro@bcm.edu (P.D.C.); mittmann@bcm.edu (M.M.I.); 5 Human Tissue Acquisition & Pathology Shared Resource, Dan L. Duncan Comprehensive Cancer Center, Baylor College of Medicine, Houston, TX 77030, USA; 6 Michael E. DeBakey Veteran Affairs Medical Center, Houston, TX 77030, USA; jajones@bcm.edu; 7 Department of Urology, Baylor College of Medicine, Houston, TX 77030, USA; 8 Dan L. Duncan Comprehensive Cancer Center, Baylor College of Medicine, Houston, TX 77030, USA; 9 Department of Pharmacology and Toxicology, University of Alabama at Birmingham, Birmingham, AL 35294, USA; 10 Verna and Marrs McLean Department of Biochemistry and Molecular Biology, Baylor College of Medicine, Houston, TX 77030, USA

**Keywords:** prostate cancer, cancer disparities, DNA damage, metabolism

## Abstract

**Simple Summary:**

Prostate cancer is the most diagnosed cancer among men in the United States. African American men are diagnosed with and succumb to prostate cancer at higher rates than other demographic groups. Previously published works described the biological differences in prostate tumors that may contribute to poorer outcomes in African American men compared to European American men. This study was designed to explore the DNA lesion profiles found in prostate tissues. Using tissue microarrays, we found that prostate tumors from African American patients have more uracil and pyrimidine damage, elevated UNG levels, and reduced XRCC1 levels than European American tumors, which may indicate defects in the base excision repair pathway. In addition, these men had higher UMP and lower expression of folate cycle metabolites, suggesting that metabolic rewiring may also contribute to the dysregulation of base excision repair.

**Abstract:**

African Americans (AA) are two times more likely to be diagnosed with and succumb to prostate cancer (PCa) compared to European Americans (EA). There is mounting evidence that biological differences in these tumors contribute to disparities in patient outcomes. Our goal was to examine the differences in DNA damage in AA and EA prostate tissues. Tissue microarrays with matched tumor-benign adjacent pairs from 77 AA and EA PCa patients were analyzed for abasic sites, oxidative lesions, crosslinks, and uracil content using the Repair Assisted Damage Detection (RADD) assay. Our analysis revealed that AA PCa, overall, have more DNA damage than EA PCa. Increased uracil and pyrimidine lesions occurred in AA tumors, while EA tumors had more oxidative lesions. AA PCa have higher levels of UMP and folate cycle metabolites than their EA counterparts. AA PCa showed higher levels of UNG, the uracil-specific glycosylase, than EA, despite uracil lesions being retained within the genome. AA patients also had lower levels of the base excision repair protein XRCC1. These results indicate dysfunction in the base excision repair pathway in AA tumors. Further, these findings reveal how metabolic rewiring in AA PCa drives biological disparities and identifies a targetable axis for cancer therapeutics.

## 1. Introduction

Prostate cancer (PCa) is the most diagnosed cancer among men in the United States [1]. African American (AA) men are two times more likely to be diagnosed with and succumb to prostate cancer than European Americans (EA) [1]. AA men often are diagnosed at an earlier age and present at an advanced grade and stage of disease than their EA counterparts [2]. While many socioeconomic and environmental factors contribute to these disparities, there is evidence of biological differences in the tumors of AA and EA PCa patients, particularly in tumor metabolism and genomic instability [3,4,5,6,7,8,9,10,11,12]. Our lab previously identified DNA repair as a top gene signature enriched in AA compared to EA across a pan-cancer gene set enrichment analysis (GSEA) of TCGA data [13]. We found this signature enriched in 22 out of 28 cancer gene sets, including PCa [13]. Therefore, this study explored DNA repair and genomic instability differences between AA and EA PCa patients.

One difficulty in understanding genomic instability is that gene mutations and expression changes do not directly correlate to changes in DNA repair capacity. Additionally, tissue is not compatible with functional DNA repair assays. Therefore, to understand the DNA repair landscape within tumors, a measurement of DNA lesion content is needed to identify where lesion removal and repair processes have failed. We developed the Repair Assisted Damage Detection (RADD) assay to measure total DNA damage and identify specific classes of DNA lesions within tissues [14,15,16,17,18]. RADD harnesses the specificity of DNA repair enzymes to detect and excise DNA damage then tags those sites with a fluorescent dye to quantify damage within a single nucleus [14,15,19]. RADD can measure DNA repair defects or indicate highly functional DNA repair in tissues [15,16]. Measuring proficiency or deficiency in the DNA repair pathways is a significant enhancement over gene expression signatures and somatic mutation analysis. Current methods only infer the impact of mutations or gene expression changes and cannot be correlated with DNA damage left within the genome. RADD directly measures DNA damage, which can then be correlated with gene or protein expression changes to more accurately recapitulate drivers of genomic instability within tumors. We have demonstrated the ability of RADD to reflect DNA repair defects and examined the relationship between DNA damage levels, DNA repair protein expression, and DNA repair capacity previously using triple negative breast cancer cell lines [19,20]. We have also demonstrated that RADD specifically detects DNA lesions induced by oxidative stress, ultraviolet radiation, and X-ray irradiation across isolated DNA, cell lines, and tissue models [14,15,17,18].

DNA-damaging chemotherapies are common neoadjuvant and adjuvant therapies for prostate cancer, and dysregulation of DNA damage response and repair machinery is often correlated with chemoresistance and poor survival outcomes [21]. Here, we examine DNA damage and DNA lesion class differences between AA and EA PCa tumors to understand potential biological determinants responsible for the disparities in PCa outcomes for AA.

## 2. Materials and Methods

### 2.1. RADD Analysis

Tissue microarrays (TMAs) were obtained from the Baylor College of Medicine Human Tissue and Pathology Core. RADD was performed as previously described in [15].

Slides were placed on a heat block set at 65 °C and incubated for 8 min to melt the paraffin. Slides were then placed directly in 100% xylene (Fisher Scientific, Waltham, MA, USA, X3P) and incubated twice for 10 min each. Slides were rehydrated to water through sequential incubations in ethanol and water mixtures. Specifically, slides were incubated for 5 min each in sequential order of 100% ethanol (VWR, 89125-170)-0% water; 70% ethanol-30% water; 50% ethanol-50% water; 30% ethanol-70% water; 0% ethanol-100% water. Rehydrated slides were then placed in glass Coplin jars with 200 mL of 10 mM sodium citrate (VWR, JT3646-1) in water and microwaved twice for 55 sec, with a 25 s rest between each, at 120 watts until the solution reached 47 °C for antigen retrieval. Slides were cooled with five changes of water. Slides were briefly dried, and the entire block of tissue cores was outlined with a PAP pen.

For broad-spectrum DNA damage detection (Full RADD), all the enzymes in Table 1, UDG, FPG, T4PDG, AAG, and EndoIV, were added to the TMA and incubated for 1 h at 37 °C. For oxidative lesions only (oxRADD, Table 2), FPG, EndoIV, and EndoVIII were added to the TMA. For crosslinks (T4PDG, Table 2), T4PDG and EndoVI were only added to the TMAs. For uracil detection (UDG, Table 2), UDG and EndoIV were added to the TMAs. The gap-filling solution (Table 1) was added directly to the lesion removal solution and incubated for 1 h at 37 °C. Slides were then washed three times in phosphate-buffered saline (PBS, Hyclone, Logan, UT, USA, SH30028FS) for 5 min each and blocked in 2% BSA (Jackson Immuno, West Grove, PA, USA, 001-000-162) in PBS for 30 min at room temperature (RT, ~24 °C). Anti-digoxigenin (Dig) antibody (Abcam, Cambridge, UK, #ab420 clone 21H8) was incubated at a dilution of 1:250 in 2% BSA in PBS at 4 °C overnight. For multiplexed experiments, the antibody for the protein of interest was also incubated with the anti-Dig primary. Anti-UNG (1:200, Genetex, Irvine, CA, USA, GTX113860) and anti-XRCC1 (1:100, Abcam, Cambridge, UK, ab134056).

The next day slides were washed three times in PBS for 5 min each, and Alexa Fluor 546 goat anti-mouse secondary (Life technologies, Carlsbad, CA, USA, A11003) was incubated at a dilution of 1:400 in 2% BSA in PBS for 1 h at RT. If a multiplexed protein marker was used, Alexa Fluor 647 goat anti-rabbit secondary (Life Technologies, Carlsbad, CA, USA, A21235) was also incubated at a dilution of 1:400 in 2% BSA in PBS for 1 h at RT. Hoechst 33342 (Life Technologies, Carlsbad, CA, USA, PI62249) was added at a final dilution of 1:1000 for 15 min at RT to stain the nuclei. Slides were washed three times in PBS for 5 min each, briefly dried, and mounted with coverslips using ProLong Gold Antifade reagent (Life Technologies, Carlsbad, CA, USA, P36930). Slides were allowed to dry overnight in the dark at RT and visualized using a Nikon A1R confocal microscope or stored at 4 °C until analysis.

Images were acquired using a Nikon A1r scanning confocal microscope with a Plan-Apochromat 10x/0.5 objective. Image acquisition settings were obtained for the full RADD samples. Reference kidney and liver samples on both TMAs were then used to normalize settings for comparison between AA and EA slides (Supplemental Figure S1). This normalization of the signal allowed the slides to be compared and controls differences in staining. These imaging conditions were then used for all tissue imaging allowing for direct comparisons and analysis between tissues on each slide. Each core was imaged at 10x with 1024 × 1024 resolution. Intensity for each core is then recorded for each condition. The values for replicate cores were averaged when available, and the final mean fluorescent intensity for each patient’s tumor and benign-adjacent tissue core is reported in arbitrary units ± standard error of mean (SEM). All cores were verified for tumor or benign adjacent tissue content using H&E analysis by a prostate pathologist prior to inclusion of the data analysis. Raw data are available in Supplemental Tables S1 and S2. Representative images for the patients are also provided in Figure S2.

### 2.2. Tissue Metabolite Profiling

Metabolic data were examined using a metabolomics dataset that was published by our group previously [3].

### 2.3. Statistics

Data were plotted using GraphPad Prism software and analyzed for significance using Student’s *t*-test with a Mann–Whitney post-hoc analysis for mean comparisons. Metabolomics data were analyzed using a Wilcoxon matched pairs signed rank test.

## 3. Results and Discussion

**Figure 1:**
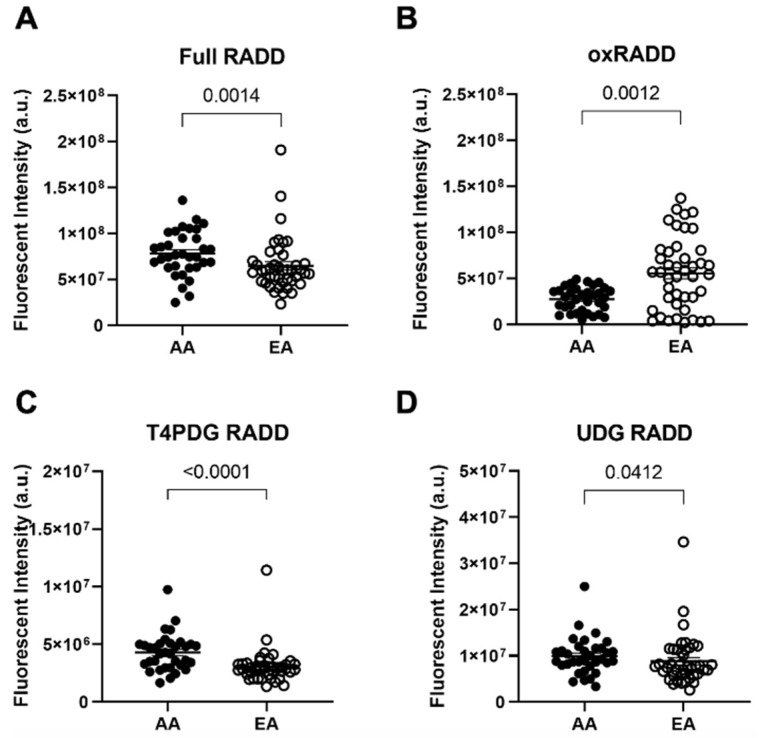
DNA damage detected by RADD demonstrates biological differences in DNA lesion types between African American and European American prostate cancer patients. Quantitative graphs measuring immunofluorescence for (**A**) Full RADD, (**B**) oxRADD, (**C**) T4PDG RADD, and (**D**) UDG RADD in AA and EA PCa patients (*n* = 34 AA and 43 EA PCa patients). Data represented are the mean ± the standard error of the mean. The Mann–Whitney test was used to calculate statistical significance between groups. Images for each patient are provided in Supplemental Figure S2.

To examine differences in DNA damage and lesion content in PCa, we used formalin-fixed, paraffin-embedded (FFPE) tissue microarrays containing 77 matched tumor-benign adjacent tissue pairs from AA and EA PCa patients with primary localized prostate cancer and resected at prostatectomy (*n* = 34 AA, 43 EA). We applied the full RADD assay to these arrays to measure abasic sites, alkylation and oxidative lesions, pyrimidine crosslinks, uracils, and strand breaks [15]. The Full RADD cocktail is composed of the bacterial repair enzymes Uracil DNA glycosylase (UDG), recognizing uracil lesions; Fapy-DNA glycosylase (FPG), oxidative lesions; T4 pyrimidine dimer glycosylase (T4 PDG), pyrimidine crosslinks; 3-alkyladenine DNA glycosylase (AAG), alkylating lesions; and Endonuclease IV (Endo IV), abasic sites (Table 1). Once these glycosylase enzymes removed the lesion, Klenow polymerase without proofreading activity is used to tag the DNA damage sites and strand breaks with a modified dUTP base for fluorescent detection (see [14,15] for more details). Each TMA slide featured matched kidney and liver sections from the same patient (Supplemental Figure S1), which were used to normalize the fluorescent signal between each slide for analysis. Using this method, AA PCa had significantly more DNA damage than EA PCa (Figure 1A, and Supplemental Figure S2, *p* < 0.001, two-tailed *t*-test).

We then used lesion-specific cocktails to measure only oxidative lesions (oxRADD, FPG + EndoVIII + EndoIV), crosslinks (T4PDG, T4PDG + EndoIV), or uracils (UDG, UDG + EndoIV) within the tissue microarrays (Table 2). This lesion class-specific analysis showed AA tumors have significantly less oxidative lesions than EA tumors (Figure 1B, *p* < 0.001, two-tailed *t*-test). AA PCa also had elevated pyrimidine crosslinks than EA PCa (Figure 1C, *p* < 0.0001, two-tailed *t*-test). Finally, AA tumors also had elevated uracil lesion content over EA tumors (Figure 1D, *p* < 0.05, two-tailed *t*-test). Overall, these data reveal that AA PCa have a more diverse set of DNA lesions associated with their disease than EA PCa, which largely have oxidative DNA lesions.

**Figure 2:**
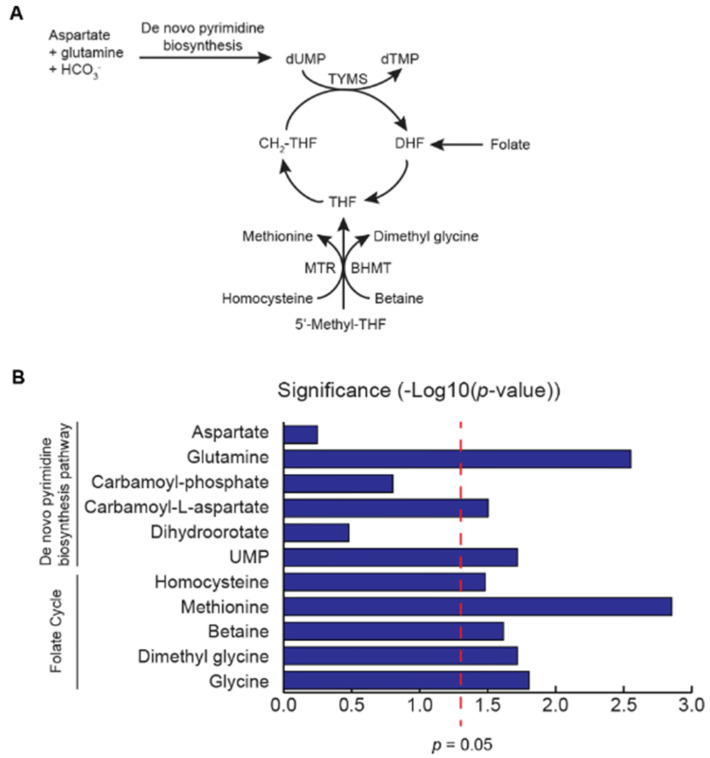
Metabolomics analysis identifies upregulation of metabolites involved in de novo pyrimidine biosynthesis and downregulation of metabolites necessary to drive the folate cycle. (**A**). Diagram of the de novo pyrimidine biosynthesis pathway and the folate cycle. (**B**). Alterations in metabolites associated with folate cycle and de novo pyrimidine synthesis in AA tumors (*n* = 50) in a previously published metabolomics dataset (3). X-axis represents the degree of significance of altered metabolites in AA tumors vs benign adjacent tissue. Bars extending the right of the red dotted line indicates the statistical significance of the metabolic alterations (*p* ≤ 0.05). The Wilcoxon matched pairs signed rank test was used to calculate statistical significance.

The uracil lesion content within AA tumors is particularly interesting because the de novo pyrimidine biosynthesis pathway is a large source of uracil production. Genomic uracil content can result from an accumulation of dUMP that is not metabolized to deoxythymidine monophosphate (dTMP), known as thymidylate stress (Figure 2A) [22,23]. Thymidylate stress is caused by a defective folate cycle that cannot metabolize tetrahydrofolate (THF) to 5′,10′-methylene-tetrahydrofolate (MTHF). Metabolites from the homocysteine-methionine and betaine-dimethyl glycine pathways help convert 5′-methyl-THF to THF to ensure the progression of the folate cycle (Figure 2A). Dysregulation of homocysteine metabolism, as well as de novo pyrimidine biosynthesis, can lead to uracil accumulation in DNA, resulting in DNA aberrations and defects in repair [24].

We examined a metabolic dataset we published earlier for metabolites in the de novo pyrimidine biosynthesis pathway to determine if upregulation of uracil production occurs in AA patients [3]. Glutamine, carbamoyl-*L*-aspartate, and UMP levels were all significantly upregulated in AA PCa compared to matched benign adjacent prostate tissue (*n* = 50 pairs) (*p* = 0.0028, 0.0313, and 0.0191, respectively; paired *t*-test) (Figure 2B). AA men are reported to have lower folate levels than EA men [25]. We then examined metabolites fueling the folate cycle in the same published dataset to examine metabolic defects in the progression of the folate cycle (Figure 2B). Using this metabolomics data, our lab has previously shown that AA PCa patients have elevated levels of homocysteine [3]. We previously also observed that betaine-homocysteine methyltransferase (BHMT) protein expression is significantly lower in AA PCa [3], suggesting an upregulation of betaine that may not be converted to dimethyl glycine. In this dataset, we also confirmed that betaine levels are significantly elevated in AA PCa (*p* = 0.0242, paired *t*-test) (Figure 2B). These data suggest that the accumulation of homocysteine and betaine could impede folate cycle progression, leading to an accumulation of UMP.

**Figure 3:**
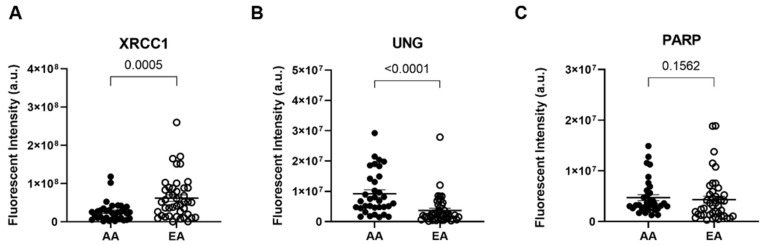
African American prostate tumors exhibit lower XRCC1 expression and higher UNG expression than European American tumors, impairing base excision repair of uracil and pyrimidine damage. Quantitative graphs measuring immunofluorescence for (**A**) XRCC1, (**B**) UNG, and (**C**) PARP1 in AA and EA PCa patients (*n* = 34 AA and 43 EA PCa patients). Data represented as the mean ± the standard error of the mean. The Mann–Whitney test was used to calculate statistical significance. Images for each patient are provided in Supplemental Figure S1.

In humans, Uracil DNA glycosylase (UNG) detects and excises the damaged base, which signals for downstream repair proteins in the base excision repair (BER) pathway, such as poly(ADP-ribose) polymerase 1 (PARP1), X-ray cross-complementing 1 (XRCC1), and DNA polymerase β (Polβ), to be recruited to the damaged site to complete repair [26]. Since we observed differences in oxidative lesions and uracil content in AA patients, both BER substrates, we examined changes in the expression XRCC1, PARP1, and UNG using immunofluorescence in our tissue microarrays. XRCC1 expression was significantly reduced in AA PCa compared to EA PCa (Figure 3A, *p* < 0.001, two-tailed *t*-test). Surprisingly, we found that UNG expression was significantly higher in AA PCa than EA PCa, even as more unrepaired uracil lesions were detected in AA tumors (Figure 3B, *p* < 0.0001 two-tailed *t*-test). We observed a 5 ± 1-fold (mean ± SEM) reduction of XRCC1 expression coupled with a 2.2 ± 0.2-fold increase in UNG expression compared to the benign adjacent tissue in the AA samples (Supplemental Figure S3). These differences in protein content contribute to the 1.5 ± 0.2-fold increase in uracil lesions observed in AA over EA tumors. We also checked for the presence of PARP1, which interacts with and recruits XRCC1 for BER. PARP1 expression in AA PCa was not significantly different compared to EA PCa (Figure 3C, *p* = 0.16 two-tailed *t*-test).

**Figure 4:**
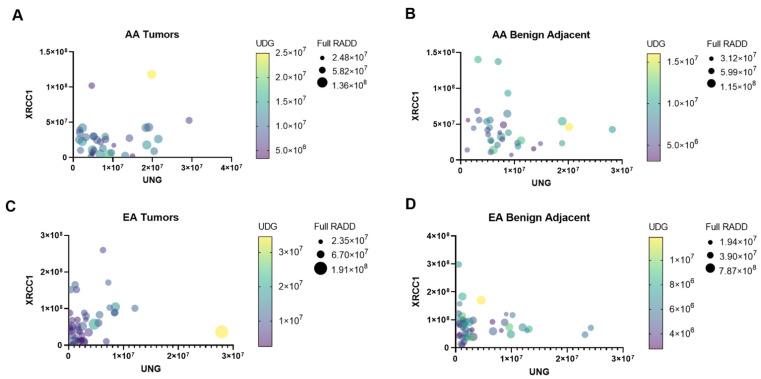
DNA repair protein expression and damage profiles show unique signatures between AA and EA tumor and benign-adjacent samples. Multiple variable graphs of XRCC1 versus UNG expression in AA tumor (**A**), AA benign-adjacent (**B**), EA tumor (**C**), and EA benign-adjacent (**D**). The symbol color intensity corresponds to the uracil damage content (UDG RADD), while the symbol size reflects the overall DNA damage content (Full RADD).

The alterations in the BER protein expression result in different lesion fates between AA and EA PCa patients. EA PCa patients show increased oxidative lesions, while AA PCa patients show increased uracil lesions. These data demonstrate that nucleotide pool changes through the alterations in metabolism influence the lesion content of the AA PCa tumors and the dysregulation of BER through imbalanced UNG and XRCC1 expression. More importantly, these data demonstrate unique clusters of DNA damage lesions within AA and EA tumors that differentiate them from their adjacent benign tissues. A multivariate graph of protein expression of XRCC1 and UNG, along with their full DNA damage profile and uracil content, highlights the distinct shifts in protein expression and DNA lesion loads between benign adjacent and tumor tissues for AA and EA patients (Figure 4). Each circle denotes a PCa patient of AA (Figure 4A,B) or EA (Figure 4C,D) origin. The separation on the x-y axis denotes the correlation between the expression of XRCC1 and UNG in these patients, while the circle size and color denote the DNA lesion content for the full RADD and the UDG RADD, respectively. Compared to the benign adjacent tissues, the AA tumors show a clear shift to lower XRCC1 expression with their collapse toward the origin and increased UNG expression (the spread along the X-axis, Figure 4A,B). The AA tumors also show a corresponding increase in uracil lesions, with higher overall lesion content (up to 2.5 × 10^7^) and overall DNA damage (increased symbol size, Figure 4A,B) compared to the benign adjacent tissues. The EA tumors show more similar clustering between benign adjacent and tumor tissues with marginal changes in XRCC1, UNG, and uracil content (Figure 4C,D). This visual representation supports potential future patient stratification using BER protein expression levels or even DNA damage measurements for BER targeted therapeutics.

The increase in mutagenic DNA lesion content and the resulting genomic instability may contribute to biological differences associated with PCa disparities. As observed in Figure 4, several publications have noted imbalances in BER attenuated response to chemotherapeutics, which has contributed to the aggressiveness of cancers [20,27]. In particular, overexpression of UNG attenuated response to pemetrexed in lung cancer and is also considered a negative prognostic marker for survival in melanoma and liver cancers [28,29,30]. Interestingly, we earlier demonstrated that reduced expression of XRCC1 sensitized breast cells to PARP inhibitors, and several groups have shown XRCC1 deficiency in breast cancer increases response to PARP, WEE1 G2 Checkpoint Kinase (WEE1), ataxia telangiectasia mutated (ATM), and ataxia telangiectasia and Rad3-related protein (ATR) inhibitors [20,27,31]. Expression changes in XRCC1 have not been previously examined in PCa or between racial groups, but more than 30 studies have examined prostate cancer patients for polymorphisms in XRCC1, particularly R194W, R280H, and R399Q. Unfortunately, the associations between these SNPs and PCa risk, onset, ancestry, and treatment response are still poorly understood due to conflicting results [32,33,34,35,36]. Our results demonstrate the need for further investigation into changes in the expression of XRCC1 and the observed alterations in DNA repair activity [37,38,39,40].

## 4. Conclusions

Our study highlights the differences in DNA lesion classes in PCa tumors from AA and EA patients detected by the RADD assay. The alterations of metabolites in the de novo pyrimidine biosynthesis pathway and the folate cycle suggests that these pathways contribute to the underlying biological differences in these tumors. The underlying biological differences between AA and EA tumors also suggest that AA patients should be stratified by their BER protein content and targeted with therapies that exploit deficiencies in this pathway, such as PARP inhibitors or emerging UNG inhibitors. Using the RADD assay in combination with protein and metabolic data, we have obtained critical insight into the DNA lesion landscape of AA PCa and identified putative therapeutic targets for AA PCa patients.

## Figures and Tables

**Table 1:** RADD reaction conditions. RADD is performed in two sequential reactions without aspirating reagents between reactions. The lesion processing mix (left) is placed on prepared tissues and placed in a humidified incubator. The gap-filling mix (right) is added directly to the lesion processing mix and incubated for an additional hour. The reagents are then aspirated, and the cells are washed and incubated with anti-digoxigenin antibody.

Full RADD Lesion Processing Mix	Per 100 µL Reaction Volume	Gap-Filling Mix	Per 100 µL Reaction Volume
UDG (NEB M0280)	2.5 U	Klenow exo^-^ (Thermo Fisher, Waltham, MA, USA, EP0422)	1
FPG (NEB M0240)	4 U	Digoxigenin dUTP (Sigma Aldrich, St. Louis, MO, USA, 11093088910)	0.1
T4 PDG (NEB M0308)	5 U	Thermo Pol Buffer	10 µL
		(NEB B9004)	
EndoIV (NEB M0304)	5 U		
AAG (NEB M0313)	5 U		
NAD^+^ (100x, NEB B9007)	500 µM		
BSA (Sigma Aldrich, St. Louis, MO, USA)	200 µg/mL		
Thermo Pol Buffer	10 µL		
(NEB B9004)			

**Table 2:** RADD lesion-specific cocktails. For detecting specific lesion classes, the lesion processing mix is modified with only the specific DNA repair enzymes of interest for that lesion class.

Cocktails	Lesion Processing Mix	Lesions
oxRADD	FPG + EndoIV + EndoVIII	Removes various types of oxidized purines, urea, 5, 6-dihydroxythymine, thymine glycol, 5-hydroxy-5-methylhydanton, 6-hydroxy-5,6-dihydrothymine and methyltartronylurea, abasic sites, and strand breaks.
T4PDG	T4 PDG + EndoIV	Removes cyclobutane pyrimidine dimers and 6-4 photoproducts along with abasic sites.
UDG	UDG + EndoIV	Removes uracil lesions and abasic sites.
Full RADD	AAG + FPG + T4 PDG + UDG + EndoIV	All of the above lesions plus the removal of various alkylated and oxidative DNA damaged sites, including 3-methyladenine, 7-methylguanine, 1, N6-ethenoadenine, and hypoxanthine.

## Data Availability

The data presented in this study are available in this article (and supplementary material).

## Supplementary Materials

The following supporting information can be downloaded at: https://www.mdpi.com/article/10.3390/cancers14041012/s1, Figure S1: Normalization of RADD fluorescent signal between EA and AA kidney and liver samples from a single patient seeded at different positions within the TMA, Figure S2: Representative patient TMA images for each variable analyzed, Figure S3: African American prostate cancer tumors exhibit lower XRCC1 expression, impairing base excision repair of uracil and pyrimidine damage; Table S1: AA Fluorescent Intensities, Table S2: EA Fluorescent Intensities.
