# Supplementary material for: Repair-Assisted Damage Detection Reveals Biological Disparities in Prostate Cancer between African Americans and European Americans
Source: Cancers (Basel). 2022 Feb 17;14(4):1012. doi: 10.3390/cancers14041012 (PMC8870190; doi:10.3390/cancers14041012)
Supplement: Supplementary file 1 [file cancers-14-01012-s001.zip › cancers-1574854-supplementary.pdf]

Supplemental Table S1. AA Fluorescent Intensities

AA	Full RADD	oxRADD	UDG RADD	T4PDG RADD	XRCC1	UNG	PARP1
AA-1-7-T	64781494.5	34575091.5	9257338	3534868	10007378	1813431	2527806
AA-1-7-N	39883836.67	35632300.3	5159039	2744122	22824745	14824364	5078146
AA-1-19-T	40614413	27437425	3340911	2054886	1754065	14951899	6140309
AA-1-19-N	43256501	35859552	3084970	2987546	15546397	13551725	2249505
AA-2-1-T	53980843	9655674.5	8691425	1651458	1344377	4451085	6903592
AA-2-1-N	45209384	23134215	5481285	1687494	13954806	1204739	2932853
AA-2-7-T	62395701	38341004.3	7798350	2954305	39020425	1590085	1697330
AA-2-7-N	68210696.67	43577612	6357375	3039545	23557562	10568211	3844155
AA-2-13-T	54857117	20981439	4691730	2604366	1.02E+08	4649876	2643247
AA-2-13-N	80064354.33	17943678.3	4039125	1963814	48952704	7941509	1997516
AA-3-1-T	84137123	23882912	10893001	3285760	2288214	13095569	2317927
AA-3-1-N	79642105	38997770.7	8048980	3754300	55604293	5454773	7845927
AA-3-13-T	102311100	39661433	8823966	5433262	42915533	19040082	11271688
AA-3-13-N	110987395.7	35445010.7	9683822	4614739	32493470	7503776	4043218
AA-3-19-T	63175387.33	34631514.3	6147506	4162657	52649055	29247707	3294424
AA-3-19-N	31239160	15659362.7	4456609	2551528	7069597	9414830	4234298
AA-4-1-T	68905368	11843630.3	8521736	2756472	6757534	9605878	3278074
AA-4-1-N	47459212.67	22127104.5	5092637	3896374	26173701	5648086	29997625
AA-4-7-T	107399465.5	35787431	13697319	5045015	17685537	18559674	3461200
AA-4-7-N	113564076.7	26416545.3	11959134	2693271	13940079	6081041	21706912
AA-4-19-T	94579250.67	32442775.3	12033259	4812786	26111202	21463914	8820307
AA-4-19-N	35640725	15752405.5	4482769	2818250	38263316	7242241	3603251
AA-5-7-T	85340201.33	49024490	8742769	4860903	24755979	6000161	2420797
AA-5-7-N	79632806.33	45971729.3	9224512	5213165	42481836	28172896	6892502
AA-5-13-T	115163339.7	20930087	14942634	5186893	10581552	4861298	5637315
AA-5-13-N	78077595.67	21122616.7	9614735	3243849	1.37E+08	6960953	4717515
AA-5-19-T	68388721	19147059	6675016	4165698	25541918	8161052	2875721
AA-5-19-N	60978227.67	18213936.7	6518722	2781432	10949886	5574840	2653046

AA-6-1-T	74421617	11587336.5	10657219	3604025	12910663	7466625	3003635
AA-6-1-N	73242177.67	24434972	8916025	3578474	53761599	4801282	2989886
AA-6-7-T	87256888	32503146.7	10805724	4783652	25073726	1479613	1309793
AA-6-7-N	85799326	51301822.3	11077366	4861710	1.4E+08	3208531	4736266
AA-7-1-T	62712033	5406888.5	8840227	3537586	6415825	4776673	1718729
AA-7-1-N	78699457.67	19056371	9805944	3985200	27374245	5583847	5353793
AA-7-7-T	110813790.3	44467629	13414242	6255322	22694695	2258038	3144525
AA-7-7-N	69770466	23980935.3	8827823	4880259	45249319	5381178	3813802
AA-7-13-T	105570813	24617603	11638874	4290086	41965794	2308565	5002268
AA-7-13-N	99507270.33	26006933	7690047	4432305	64574206	8655119	11534129
AA-7-19-T	136135406	42498631.3	16584601	7038562	3921056	8963619	3226335
AA-7-19-N	61558858.33	23840919.3	10008518	3683423	23320654	18846007	13895144
AA-8-1-T	76564537	10785498	10412457	5074722	8877292	20404478	2308713
AA-8-1-N	71235927.67	22384837	8905589	3937206	38084399	8862836	7281995
AA-8-7-T	82857638.33	46610434.3	10980204	6332666	30092144	5087036	3614868
AA-8-7-N	76994867	48579417.7	10096216	5230471	93157433	8758252	4267222
AA-8-13-T	72267983.33	36159654.3	11632607	4888757	29753558	8075741	12780114
AA-8-13-N	59713001.67	21686537	5755854	2988550	68456620	3080531	8359808
AA-8-19-T	94930068.67	29825626	11044391	4984557	42101379	18319401	11567598
AA-8-19-N	65915857.67	20735428.7	7679804	2630730	20885151	6824895	6878041
AA-9-1-T	101393607.5	15355644.5	13072032	4993885	4857111	6737707	4236788
AA-9-1-N	102378095.7	39432143	11682284	6186609	27158711	11211244	5009249
AA-9-7-T	74216992	42950462.5	8657968	4705989	29732947	5180103	7605483
AA-9-7-N	64587204	37502406.5	6095566	2890208	41790668	4982861	6230978
AA-9-13-T	83209868.33	36790285.7	10010601	3510005	28648346	3151844	3205259
AA-9-13-N	32991745.67	15778941.7	3800685	2104384	55521598	1351724	3326015
AA-9-19-T	104616559.5	45783362.5	25022193	9735486	1.18E+08	19799075	14920955
AA-9-19-N	107146841.7	32730369	16011217	5675701	46066816	20132200	10646070
AA-10-1-T	48224391.5	10722628	6243849	4695703	10706842	5140325	3027150
AA-10-1-N	68757766.67	18633404.7	6279030	3450234	55775939	3426902	3965772
AA-10-7-T	77531036.5	33952875.5	8947931	3214485	34803583	1640179	2768232

AA-10-7-N	49777406.5	43221087.7	6748177	2584389	44359789	2400429	4022843
AA-10-19-T	68845142.33	19036434	8208078	3431111	22021917	6865403	4731104
AA-10-19-N	66728947.33	26365627	8776487	3066417	18934191	10643325	5284602
AA-11-1-T	31720767.67	7561325	4968022	2444996	17191980	10235776	1250647
AA-11-1-N	51949392	22227785	5187809	2712223	35508457	5088705	5021744
AA-11-7-T	74533134	28976165.5	7981832	2954620	27370577	14171412	4046787
AA-11-7-N	114789376.3	36158225.7	10541313	4319360	53987091	18875669	9936540
AA-12-1-T	24841734	8374691.5	4396631	2797450	5180214	4770444	3681652
AA-12-1-N	56542226	13333601	5519675	2928994	28785787	7706989	4869311

Supplemental Table S2. EA Fluorescent Intensities

EA	Full RADD	oxRADD	UDG RADD	T4PDG RADD	XRCC1	UNG	PARP1
EA-1-7-T	48418907.3	29864930.3	5780405	2789911	36067162	2002636	4578887
EA-1-7-N	24598530.3	22796912.7	2762587	1472047	64386470	2921468	2332547
EA-1-13-T	80196219.3	64376268.7	6939278	3586541	34974767	3650474	2234953
EA-1-13-N	59925728.3	63632138.3	5130992	2737248.667	58862252	6695767	1142396
EA-1-19-T	45970265.3	51925370.3	4093626	2000621.333	69382578	1611105	318618
EA-1-19-N	63139233	56601606	3659020	1462068.667	39014055	3155128	613371.7
EA-2-1-T	35188790	7652818.5	4445969	1965215.5	15252585	2271759	739361.5
EA-2-1-N	29359426	22336483	4321834	1839073	70387453	1290089	2328397
EA-2-7-T	65283925.7	49849680.3	7641421	3772152.333	26169374	649898.3	1043638
EA-2-7-N	35955626.3	38647975.3	3022703	1924109.333	42326110	612859.7	1128688
EA-2-13-T	53283748.3	105217160	6627830	3051791.333	68016307	635247	608735.7
EA-2-13-N	33820404.7	76711242.3	3832225	1897299.667	47101825	794965.7	971309.3
EA-2-19-T	60274562.7	107878072	8069072	2655291.333	88488336	8352189	798666.7
EA-2-19-N	51409374	58370997.3	4705176	1555876.333	97845232	3116216	1386464
EA-3-1-T	40432009.7	15031769.7	4252981	2406988.333	12568522	1653712	546141
EA-3-1-N	39416886.7	34316999.3	4992612	2497493.333	36943158	1240761	11274617
EA-3-7-T	76613681	62993314.5	7447373	3047621	41411477	1672206	1343317
EA-3-7-N	48534135.7	65561517	5352518	2197131.667	65648778	1752210	6373645
EA-3-13-T	59810217	104579340	6096240	2982381.5	38045072	462387	1945360
EA-3-13-N	28129129.7	43644557	3235482	1239359.333	79884517	720778.7	1825947
EA-3-19-T	91576005.5	113338942	10744165	2917570.5	51585059	2506021	1334774
EA-3-19-N	51174705.3	57263787.7	4852401	1985236.667	90787781	8776600	1873852
EA-4-7-T	53213009	64270297	5049853	2356710	87974926	196672.5	649838
EA-4-7-N	50766474.7	108242201	6802670	3191077.333	92356116	1793487	4254956
EA-4-13-T	62326574	81339666	8037484	2655338	51865862	1586186	4175474
EA-4-13-N	41517554	63768098.7	5306688	1973065.667	86272355	1439206	4752363
EA-4-19-T	190835129	137232950	34635069	11430147	36416111	27916616	18829701
EA-4-19-N	34805425	38200641	6305035	2963993	71289465	24223927	3169258
EA-5-1-T	90257719	4616973	6918988	4245042	10000316	2742048	18875630

EA-5-1-N	59016329.3	41286510.3	6720703	3190142.667	72758910	11990158	4303055
EA-5-7-T	56683028	39969212	7065177	2932910	1.52E+08	1418637	2602811
EA-5-7-N	78664739	123526301	7410258	3425531.333	94660889	810562.3	2644105
EA-5-13-T	40397046	66266336.3	4799138	1992565.333	43789375	431238.7	1074866
EA-5-13-N	57974328.3	95611226	6965115	3316732.667	48137371	9881968	4669517
EA-5-19-T	70182077.7	84672310.7	8172122	2976563.333	60728514	5429712	4962815
EA-5-19-N	30058864	33626164.5	3210023	1198401.5	92560503	6591323	2293530
EA-6-1-T	23511322	3892354	2610564	1426694.5	16076407	1535667	471036
EA-6-1-N	38132979.3	28590265	3840251	1836145.667	1.01E+08	1304302	3098122
EA-6-7-T	93182637.5	78940623	7888948	3231271.5	51787607	462794.5	4243876
EA-6-7-N	43386976	53271551	3950839	1862369	86346373	661398.3	4249600
EA-6-13-T	140430452	125103986	19604242	5370652.333	57437885	4647273	11508381
EA-6-13-N	55861293	100444487	9316739	2737875.333	1.14E+08	1144385	3345522
EA-6-19-T	57702662	71307568.3	11556896	3305638.333	1.01E+08	12079429	7110700
EA-6-19-N	26078662.3	39668011	6564868	1628515.667	61491913	12754577	4965544
EA-7-13-T	65827490	122018472	11766565	3353917.5	79277938	3181108	7569734
EA-7-13-N	72289535	69495079	7096286	4101673.667	92032381	2414945	6739537
EA-7-19-T	60292725.3	71473027	11386809	2829478.333	1.65E+08	1176277	13780710
EA-7-19-N	19358132	42604208.5	5055496	1263629.5	1.2E+08	9048667	2162980
EA-8-7-T	41680525	36245858.5	4723185	1341713	50442477	304722.5	1743968
EA-8-7-N	41994831.7	61309654.7	5357485	1908871.667	1.22E+08	502992.7	4697225
EA-8-13-T	50380845	119769041	7670559	3037674.667	88207601	2843789	7571461
EA-8-13-N	40605265	76203280.7	7280407	2423837	2.98E+08	534536	2555878
EA-8-19-T	47925291.3	80436658.3	7842827	2859974.333	2.6E+08	6252750	3637686
EA-8-19-N	56015269	62134339.7	9316513	2334804.333	73649951	9597349	5010716
EA-9-1-T	51221655	3938971	6276159	2027583.667	10244572	6861394	4061729
EA-9-1-N	46531665.7	24975180	9335041	2648926	36864968	2819703	4971839
EA-9-7-T	59768253	56677243	10164109	3019131	19622290	1244354	3090306
EA-9-7-N	58804959.3	42795609	7618098	2437444	81957953	1466032	10407257
EA-9-13-T	67233484.7	58547683.3	12860591	2765748.667	89452779	8402532	5258605
EA-9-13-N	57647461.7	48007212.7	8078303	2457196.333	1.83E+08	1205485	5320787
EA-9-19-T	45112634.5	57044488.5	11424048	3562724.5	1.71E+08	7226901	5349489

EA-9-19-N	32940722	40247859	6461277	1793992.333	1.17E+08	10196671	2346809
EA-10-1-T	36042885	6059818	3910615	1958112.5	13489511	2548863	1692025
EA-10-1-N	36972419.3	24587056.7	5365533	2133595.667	58450961	2501239	3035097
EA-10-7-T	52480934	32806195.7	5849972	2002846	26643769	2433324	2437619
EA-10-7-N	39796608	25227264.7	4410986	1729590.667	20662562	1501575	6933580
EA-10-13-T	65954302	29584888.5	12758545	3150404.5	83042611	5623253	10775612
EA-10-13-N	70241420	30525100	11885323	3416396.667	1.7E+08	4561271	13845348
EA-10-19-T	59922583.5	51706365	12458374	3283019.5	1.52E+08	393892	3169128
EA-10-19-N	40346146.7	54403929.5	5923583	1926822	46772297	23174059	5530121
EA-11-1-T	116209780	4056072.5	11326378	4107924.5	9323429	873041.5	2016909
EA-11-1-N	35575300.7	13000982.3	4817904	2146086.667	50502524	1753449	3605106
EA-11-7-T	56207768	21911257	4174381	1726478	11568491	2152342	1495635
EA-11-7-N	63556371.3	32553525.7	8697147	3238958	36982064	2020351	7567192
EA-11-13-T	46538977.5	29049660.5	12142421	2862378	1.03E+08	7467649	6630290
EA-11-13-N	29210524.3	27247698	6505065	2080281.667	1.57E+08	719730	3494628
EA-11-19-T	90449209.3	62587659.7	16709260	3851901.333	1.05E+08	8531494	4316766
EA-11-19-N	45658127.3	42704635.3	8331932	2095039	66753607	13169998	3868458
EA-12-1-T	82371465	2182043.5	8217103	2584089	553751	906249	5917974
EA-12-1-N	56960207.7	6028025	5300354	2276176	13226593	1070920	2381154
EA-12-7-T	62932805	15596674	7272743	2529470.667	7690211	2794019	1232013
EA-12-7-N	45432873.7	17601344.3	5860421	2424688.667	38121713	1796641	3160447
EA-12-19-T	34915730.7	54167298	6969007	2876810.667	69996616	3122236	3637870
EA-12-19-N	20762325.7	14685453	3392730	841497.6667	61544990	8182742	1342255
EA-13-1-T	59510995.7	3002663.33	5829941	3261013	193275	770402.5	1080365
EA-13-1-N	37020535	6079385.33	3748625	2194544.333	10302462	1142238	1757747

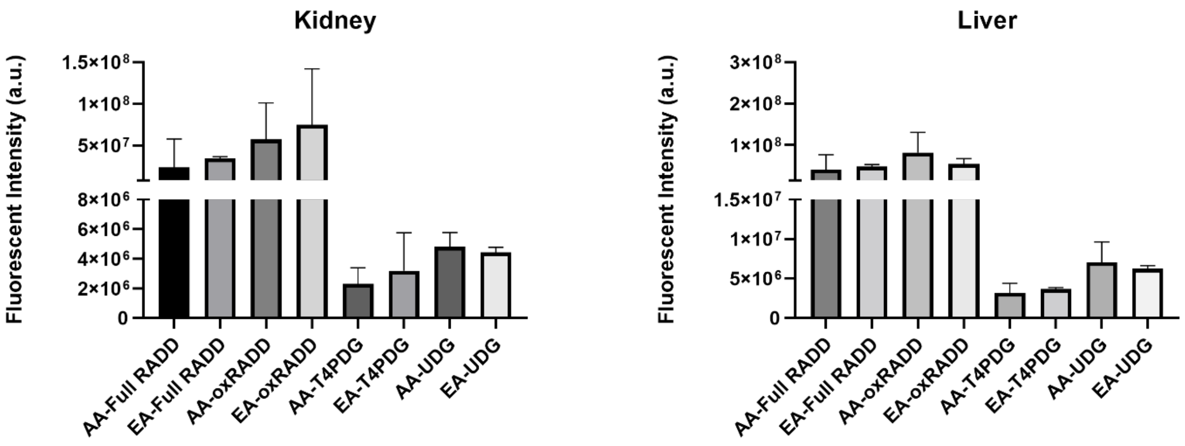

Supplemental Figure S1. Normalization of RADD fluorescent signal between EA and AA kidney and liver samples from a single patient seeded at different positions within the TMA.

The mean fluorescent intensity ± standard deviation (n = 1, with 6 technical replicates per slide) for each slide sample is shown, and imaging conditions that achieved this level of intensity equity between the two slides were used to image the entire slide for comparison.

Supplemental Figure S2. Images from TMAs for each patient.

Images for multiple TMAs are grouped into individual patient panels to show signal variation across analysis groups. Full RADD, oxRADD, T4PDG, UNG, and XRCC1 were imaged on separate TMA slides. UDG and PARP1 were imaged on the same TMA slide. All the images were captured at the same magnification, using a Nikon A1r scanning confocal microscope with a Plan-Apochromat 10x/0.5 objective. Each core was imaged at 10x with 1024 × 1024 resolution. Data was normalized using reference kidney and liver samples, as described in Methods. To create the composite image for each patient core, individual images were combined into a single panel using the montage feature in Nikon NIS Elements software. No changes were made to the core images or their respective backgrounds, in the combined panel.

Row 1 Full RADD

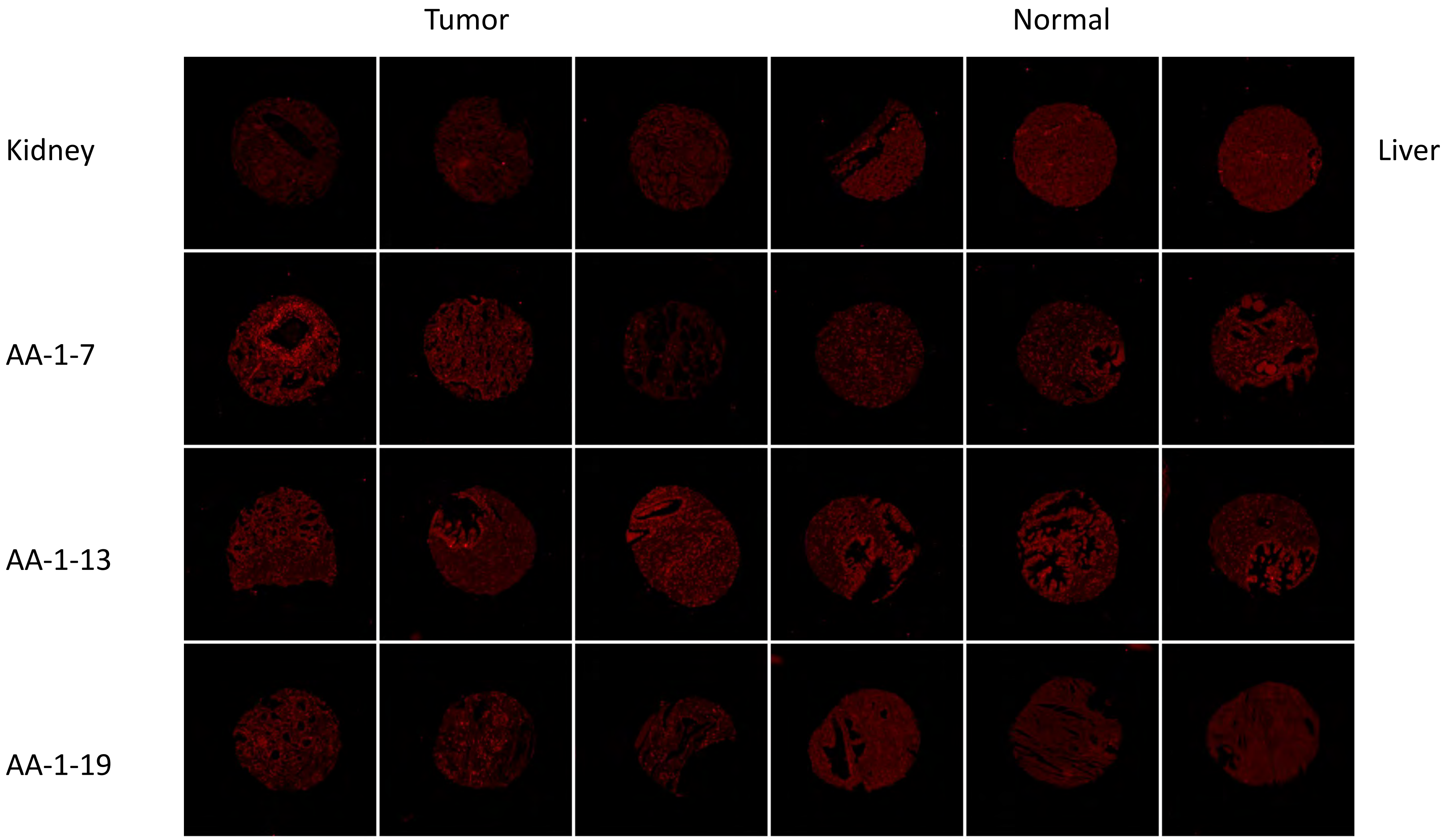

Row 1 oxRADD – Kidney and liver removed

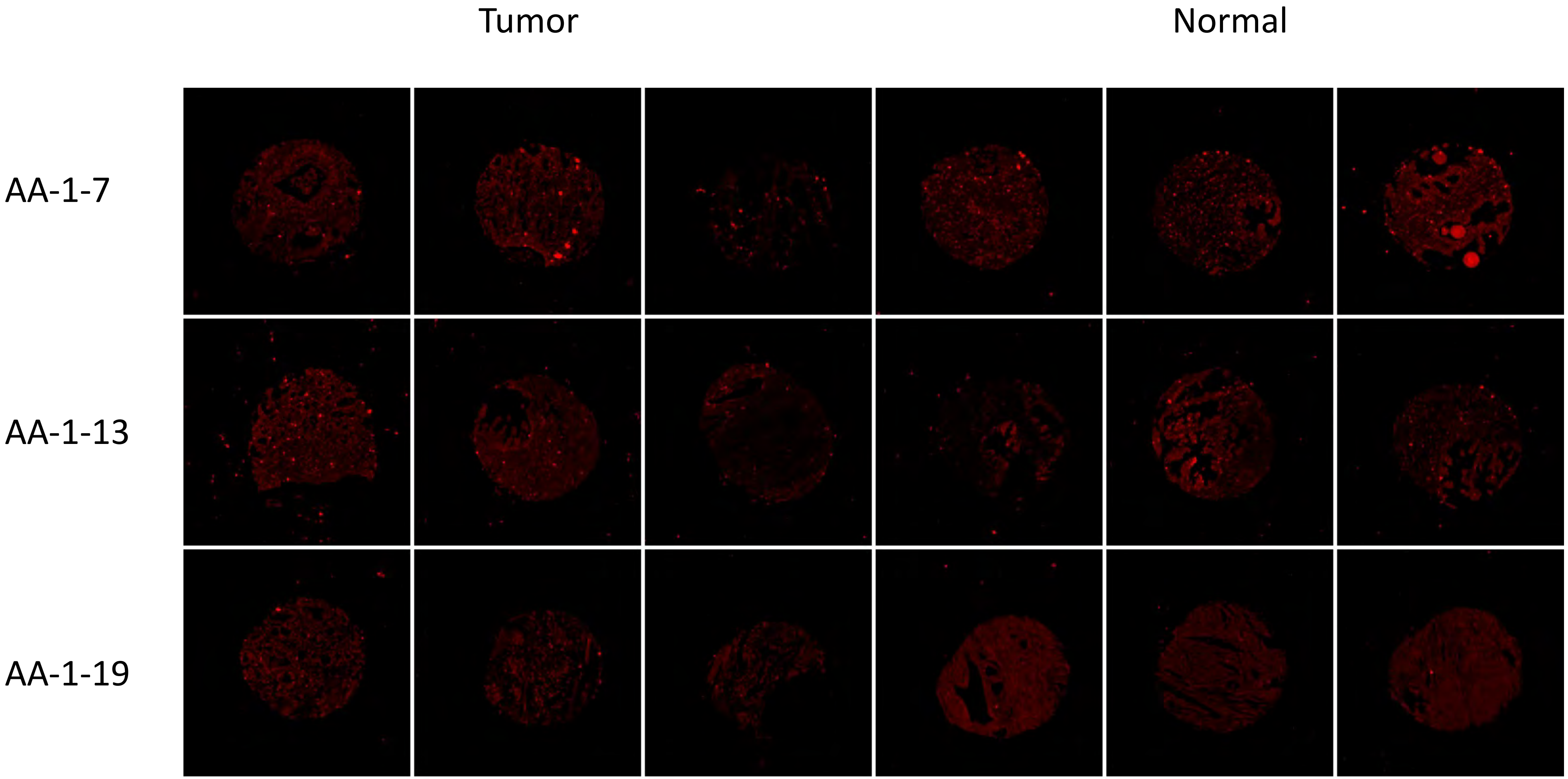

Row1- UDG

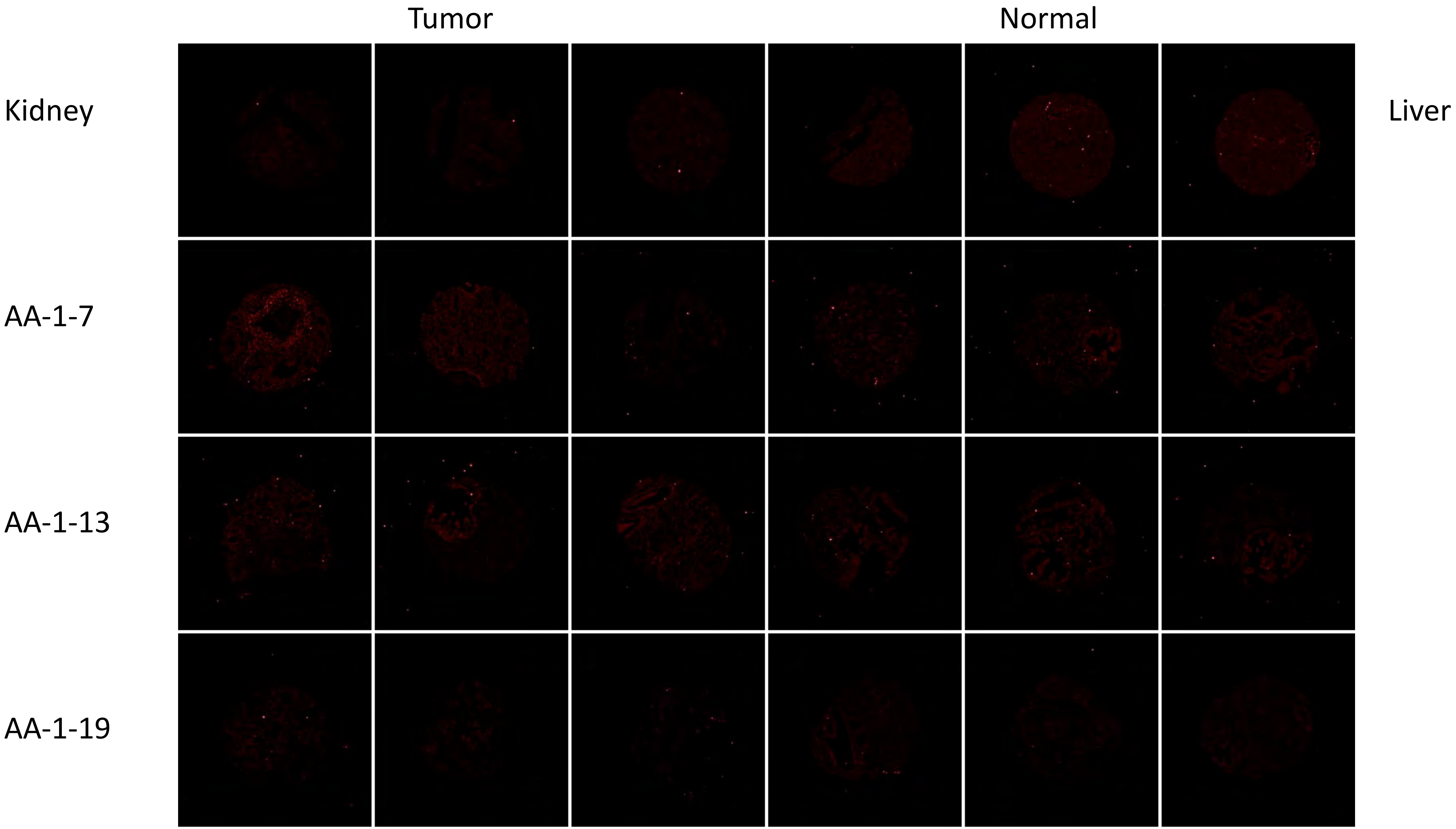

Row 1- T4PDG

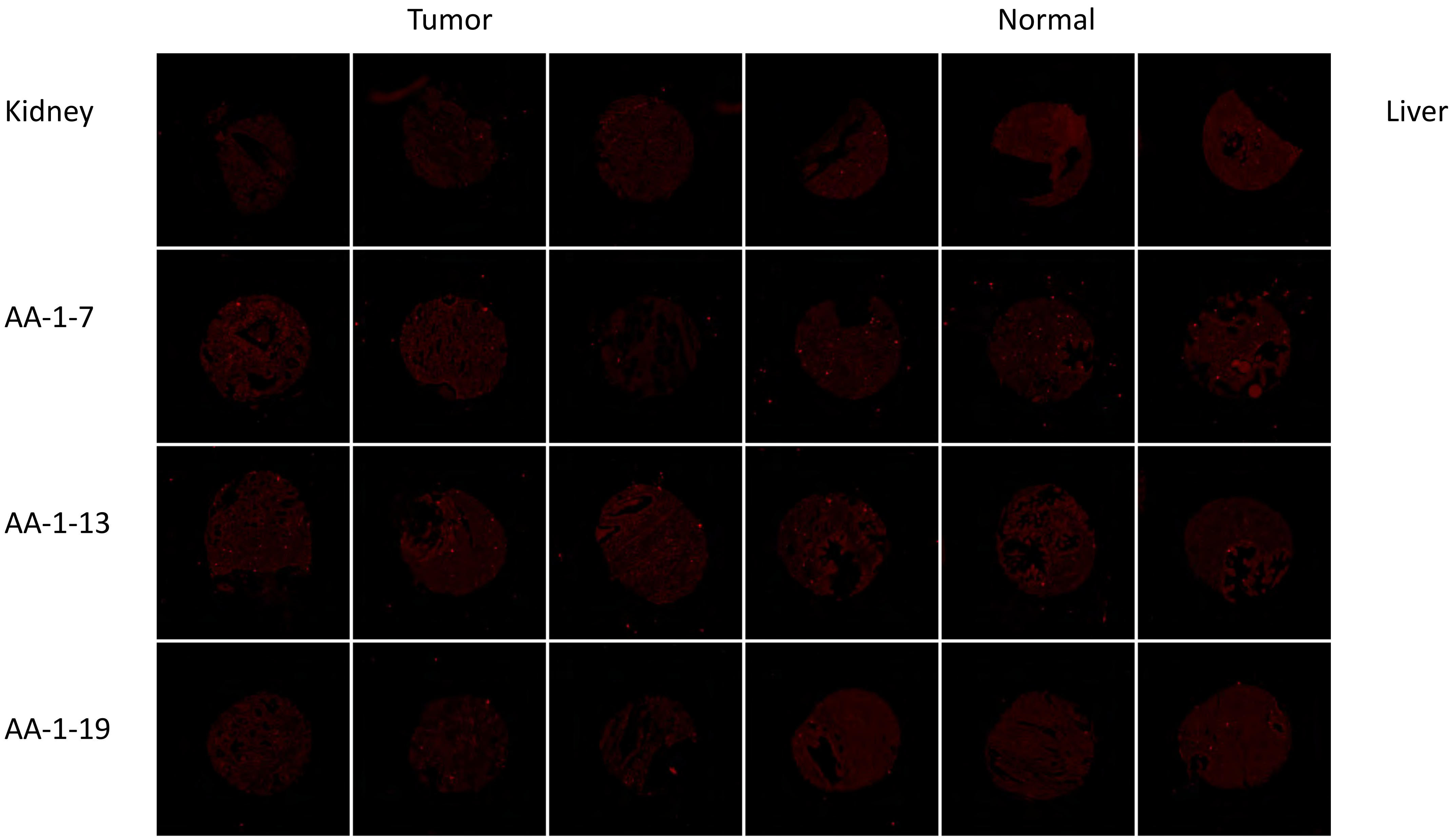

Row 1- XRCC1

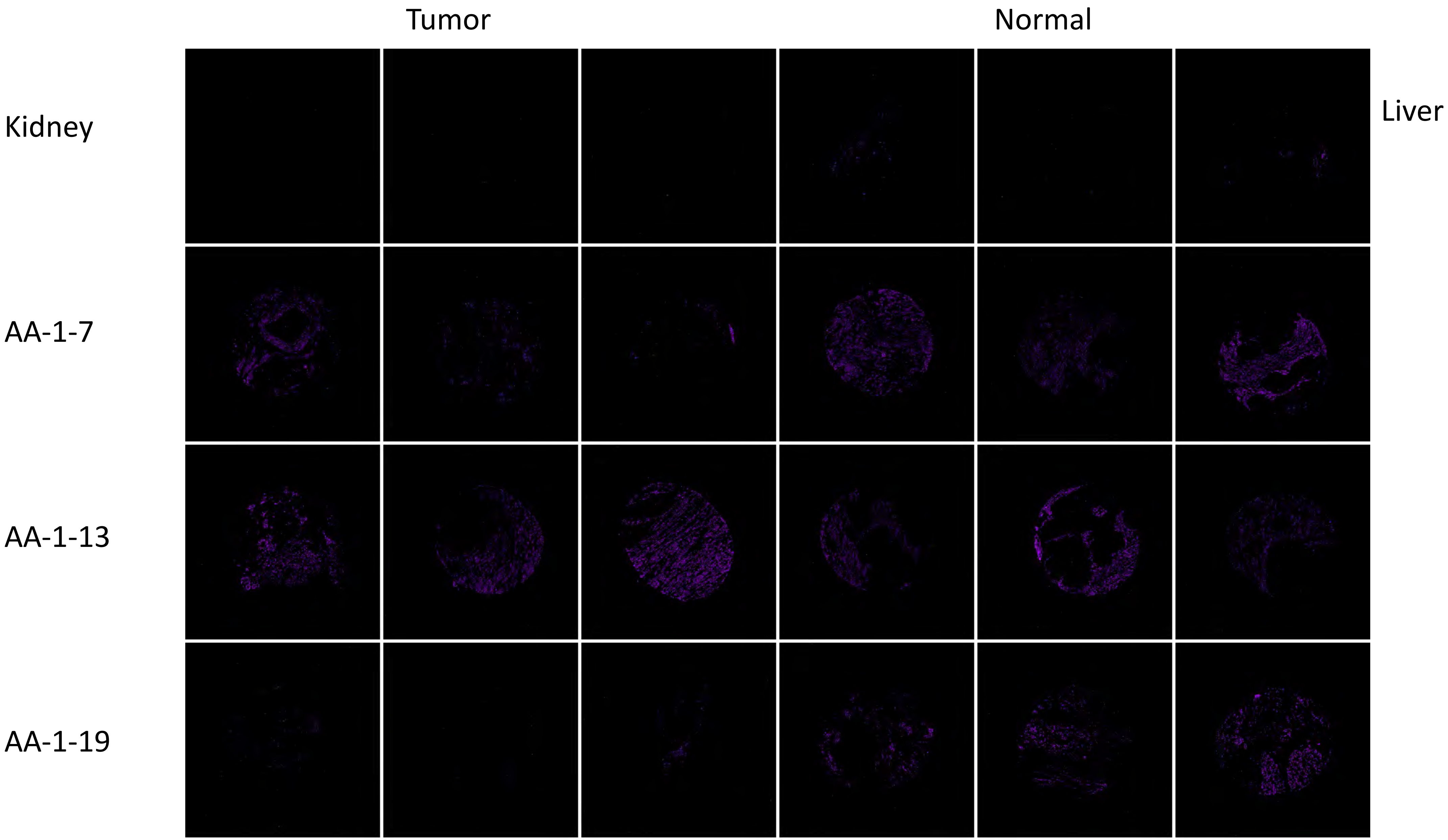

Row 1 PARP1

Tumor Normal

Kidney Liver

AA-1-7

AA-1-13

AA-1-19

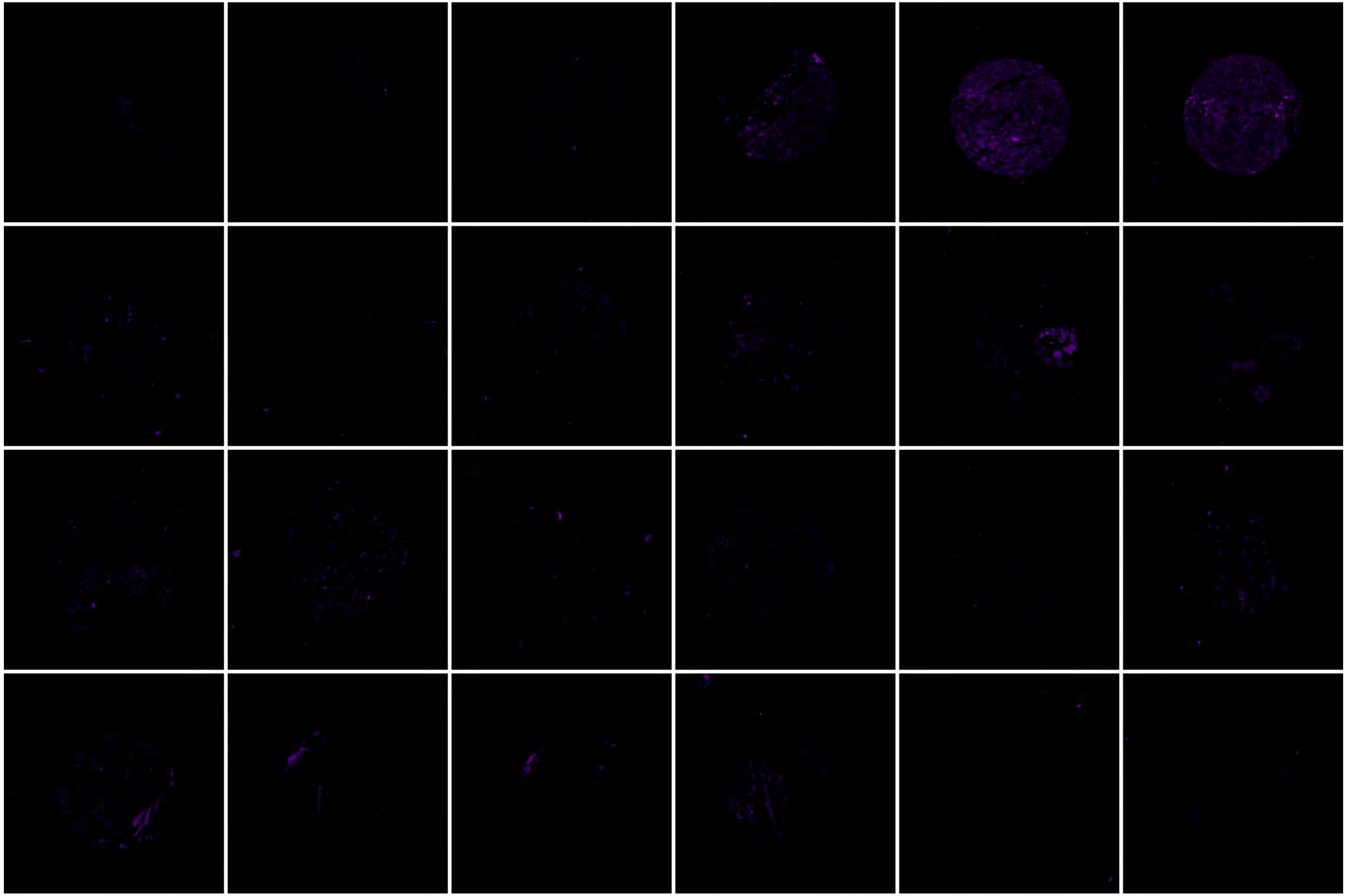

Row 1- UNG

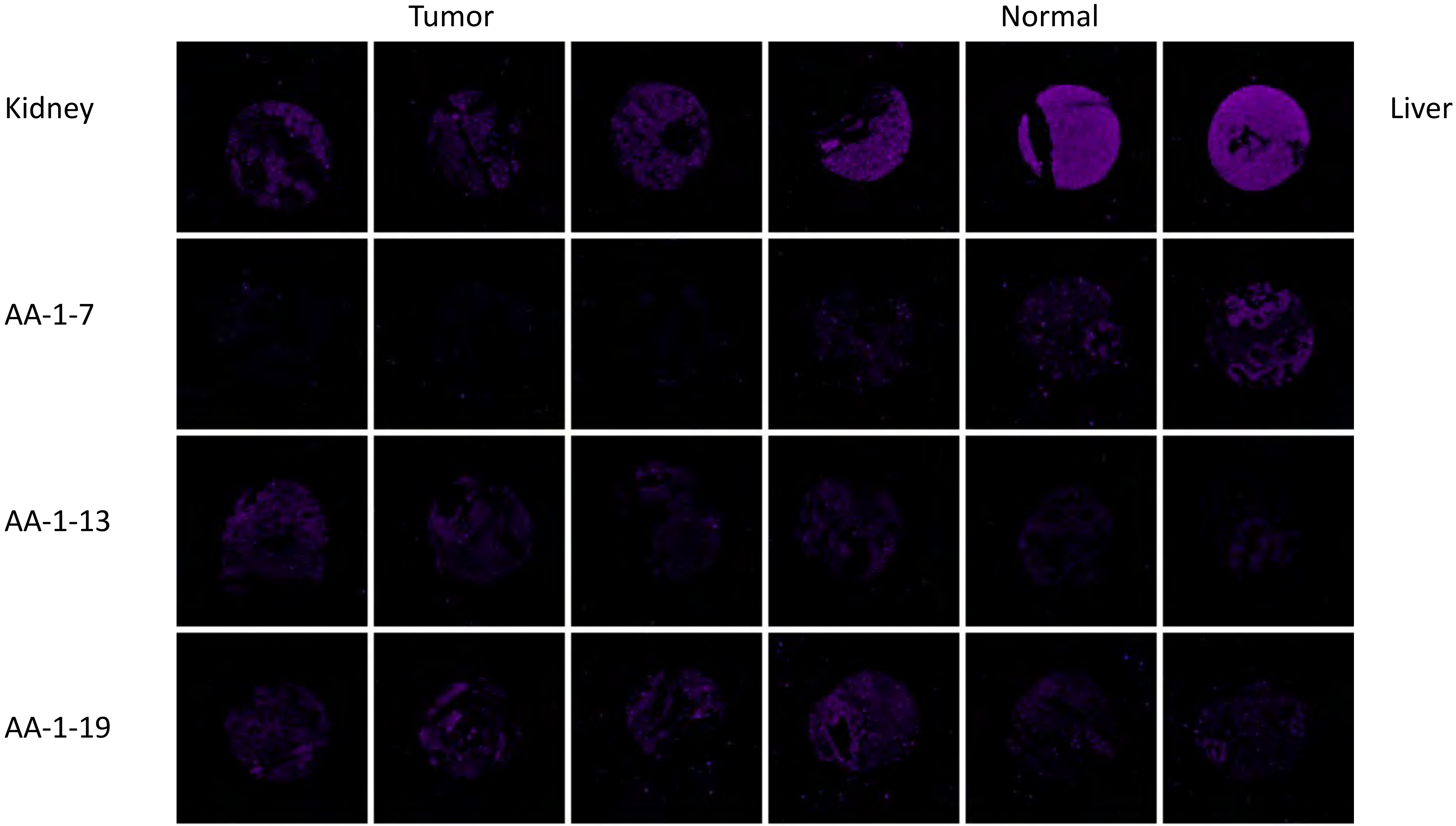

Row 2- Full RADD

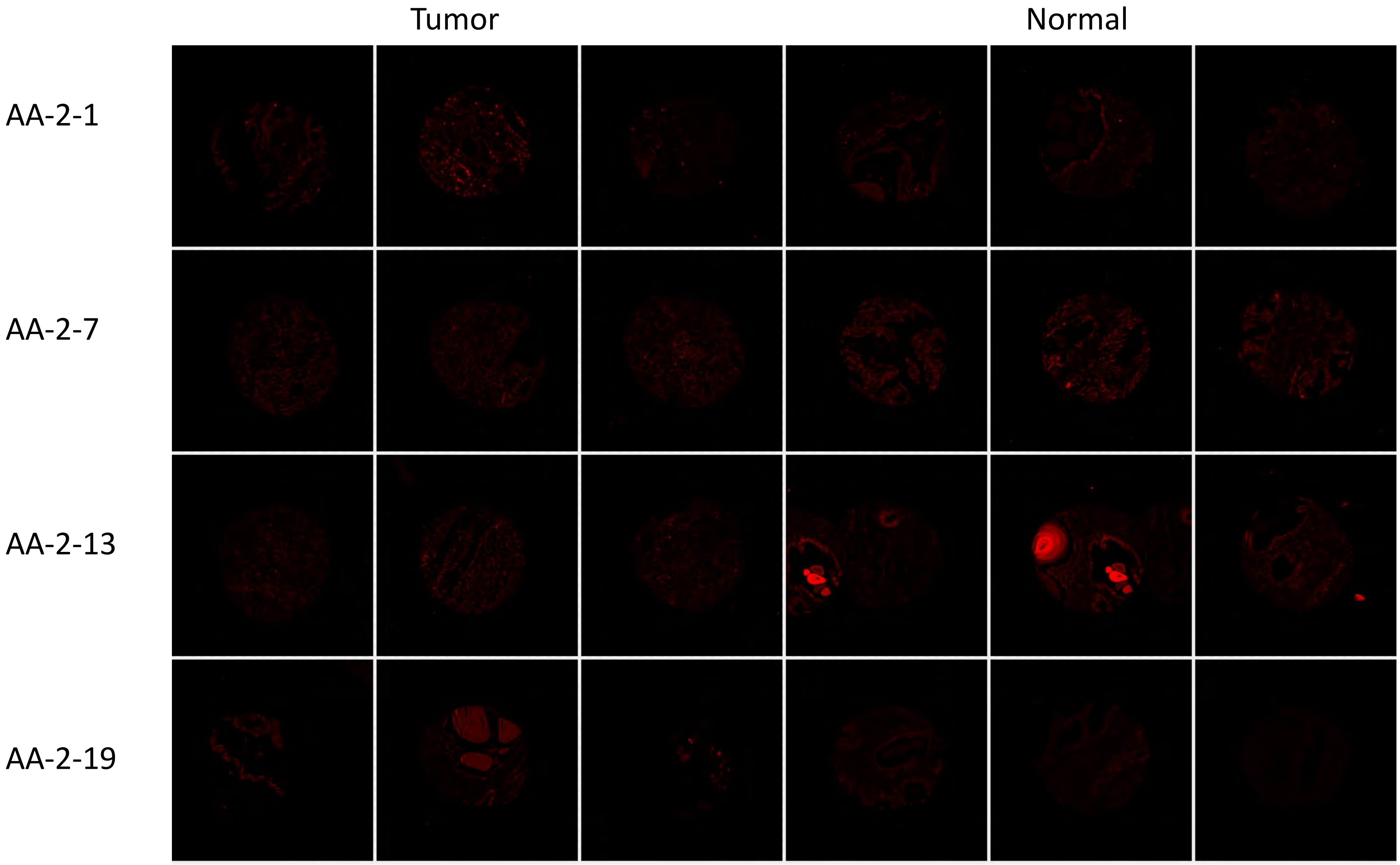

Row 2- oxRADD

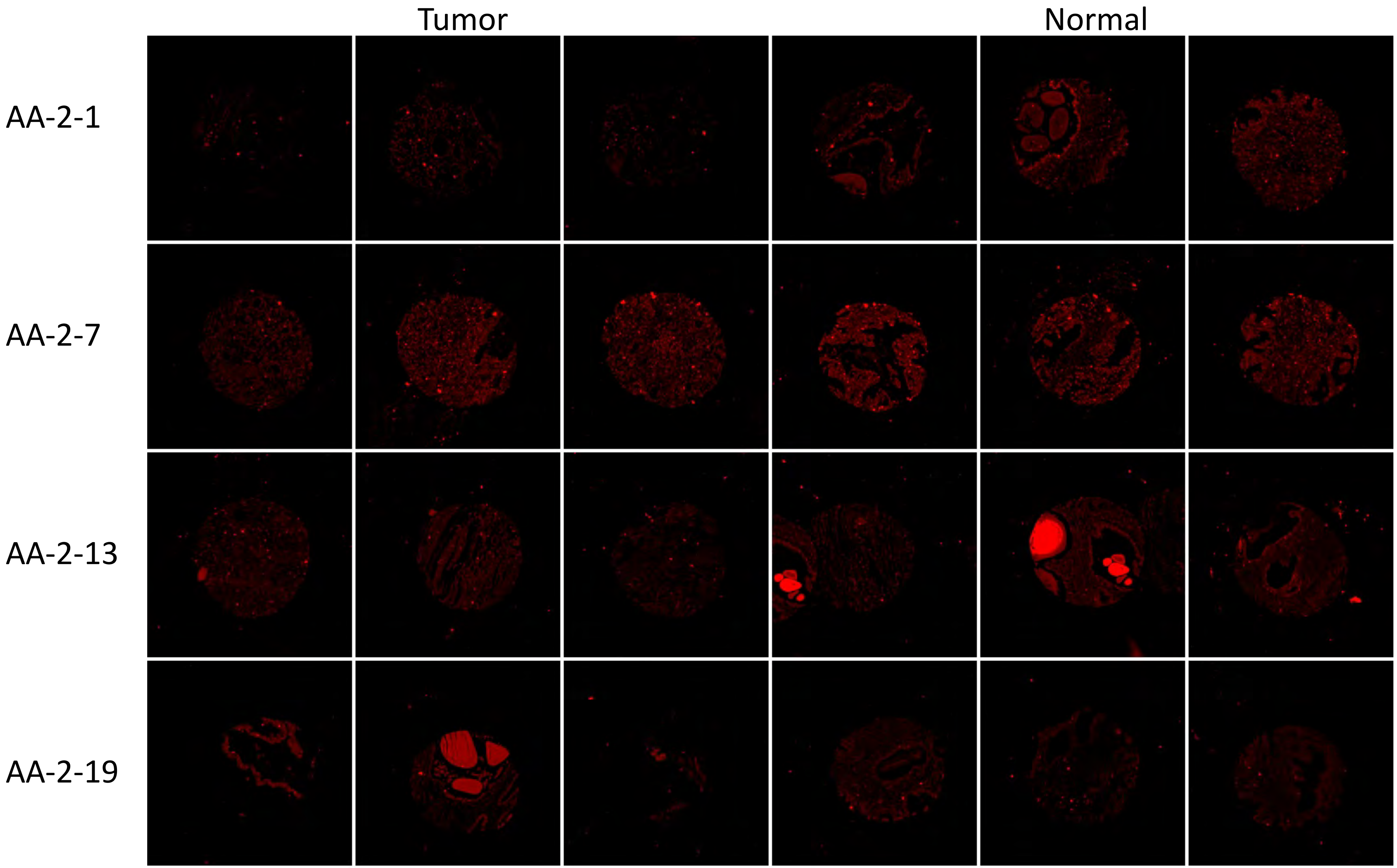

Row 2- UDG

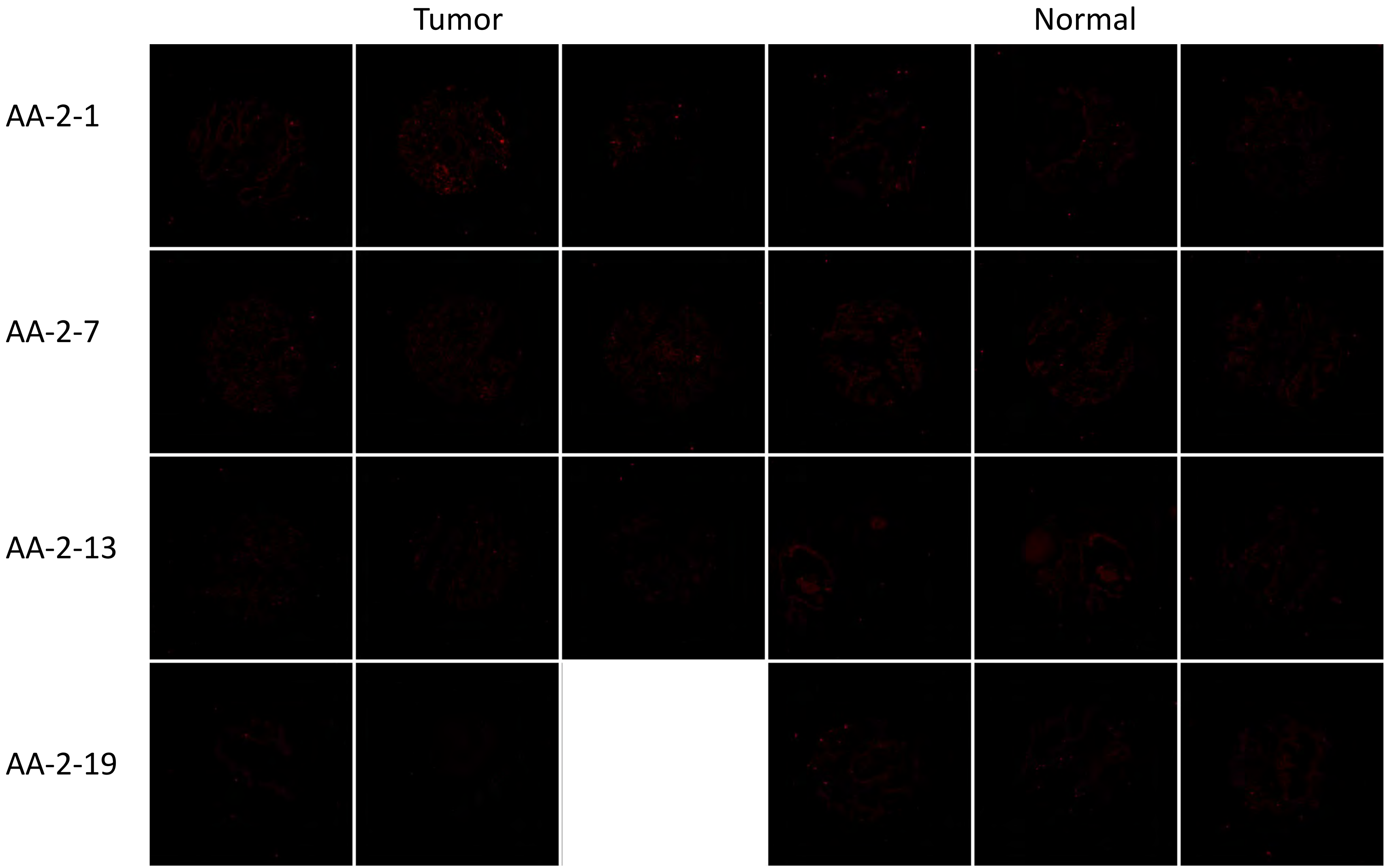

Row 2- T4PDG

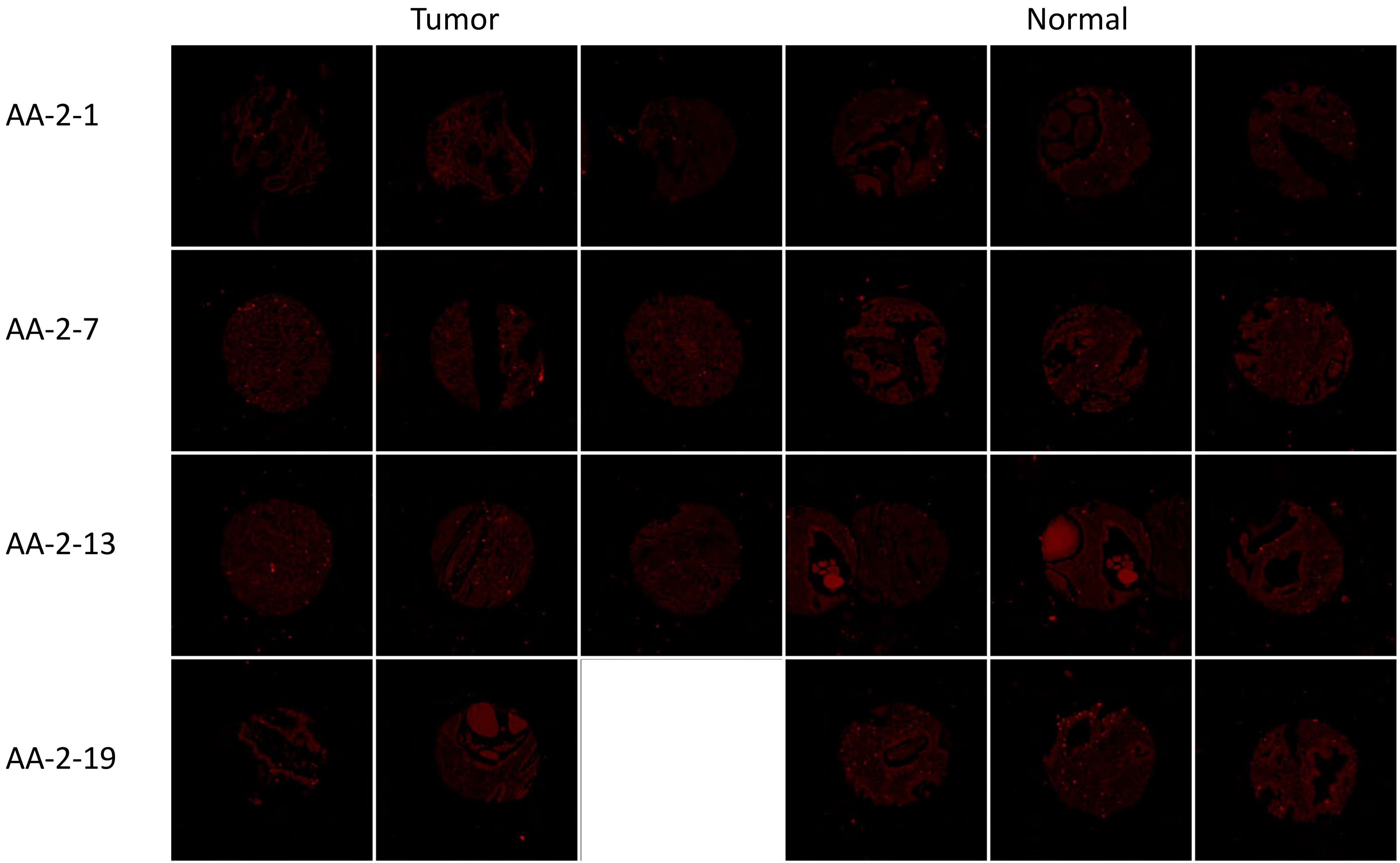

Row 2- XRCC1

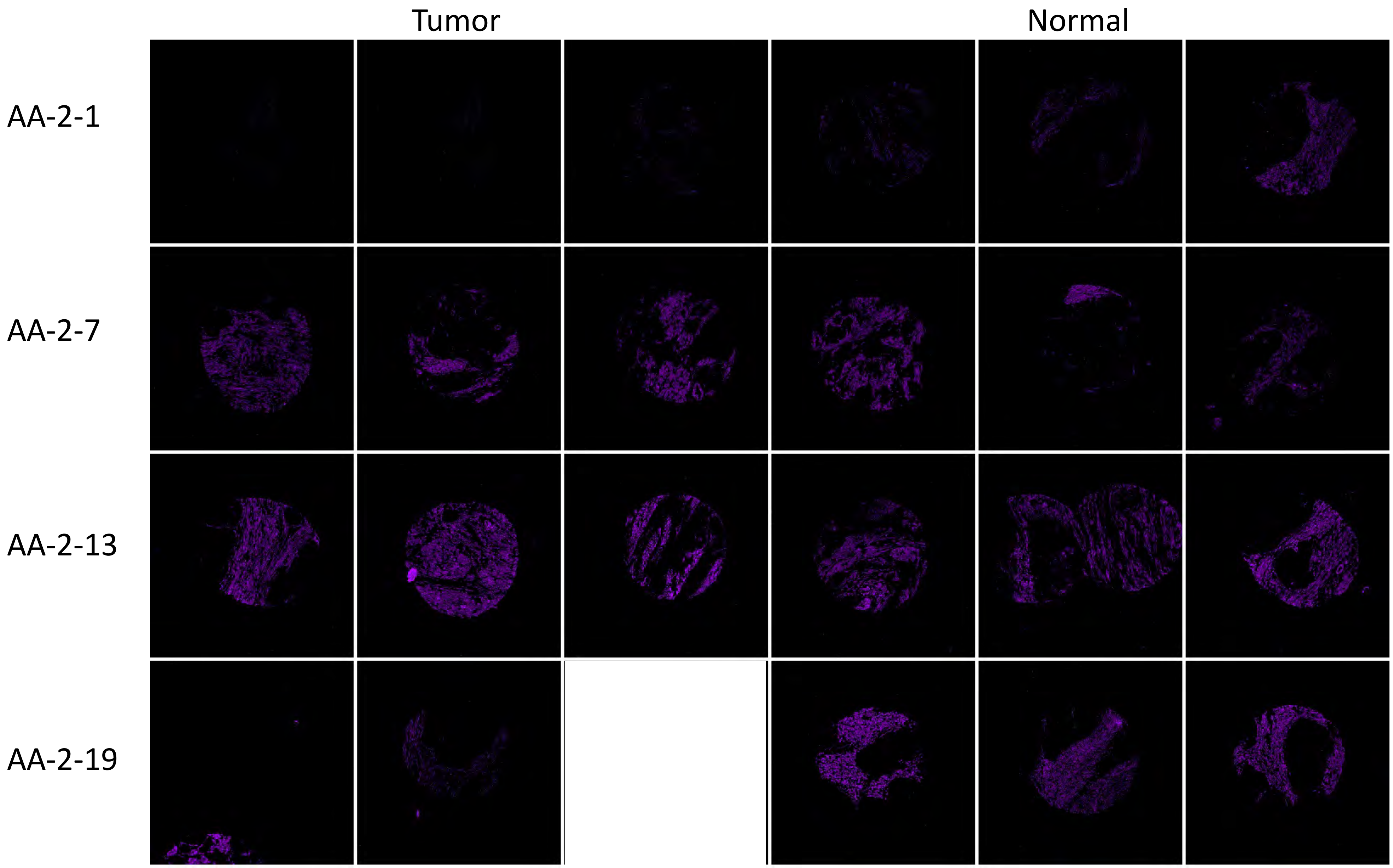

Row 2- PARP1

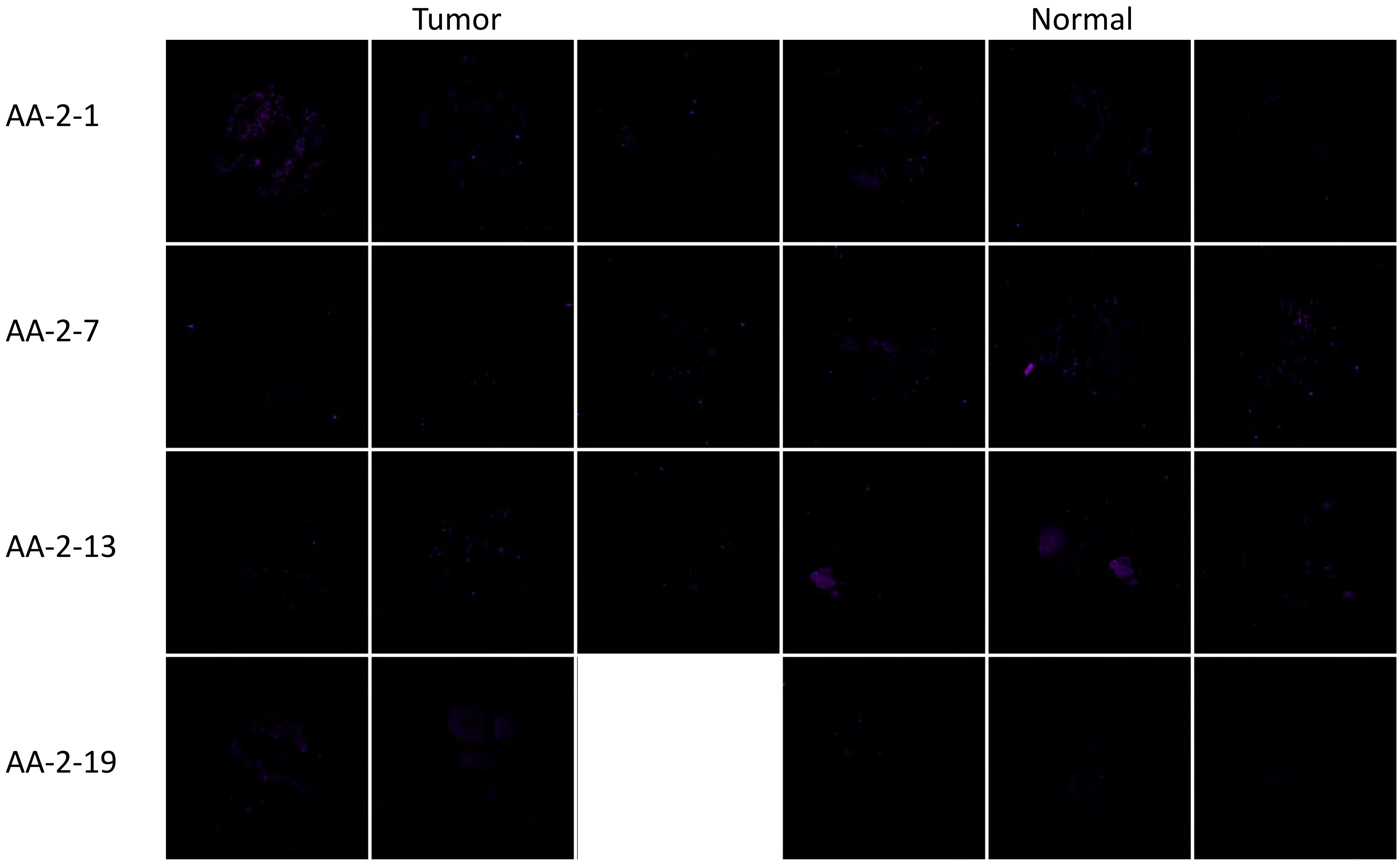

Row 2- UNG

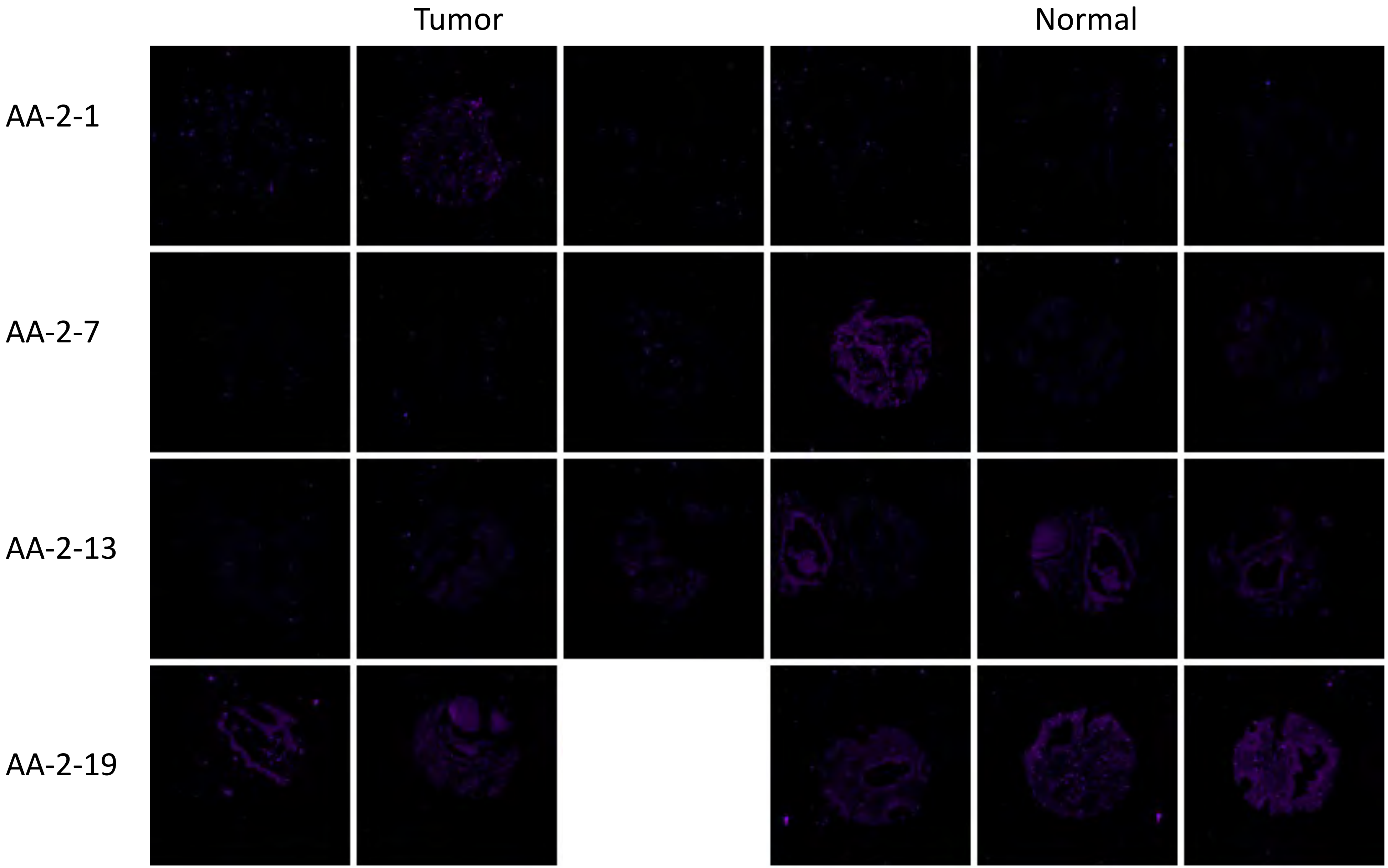

Row 3- Full RADD

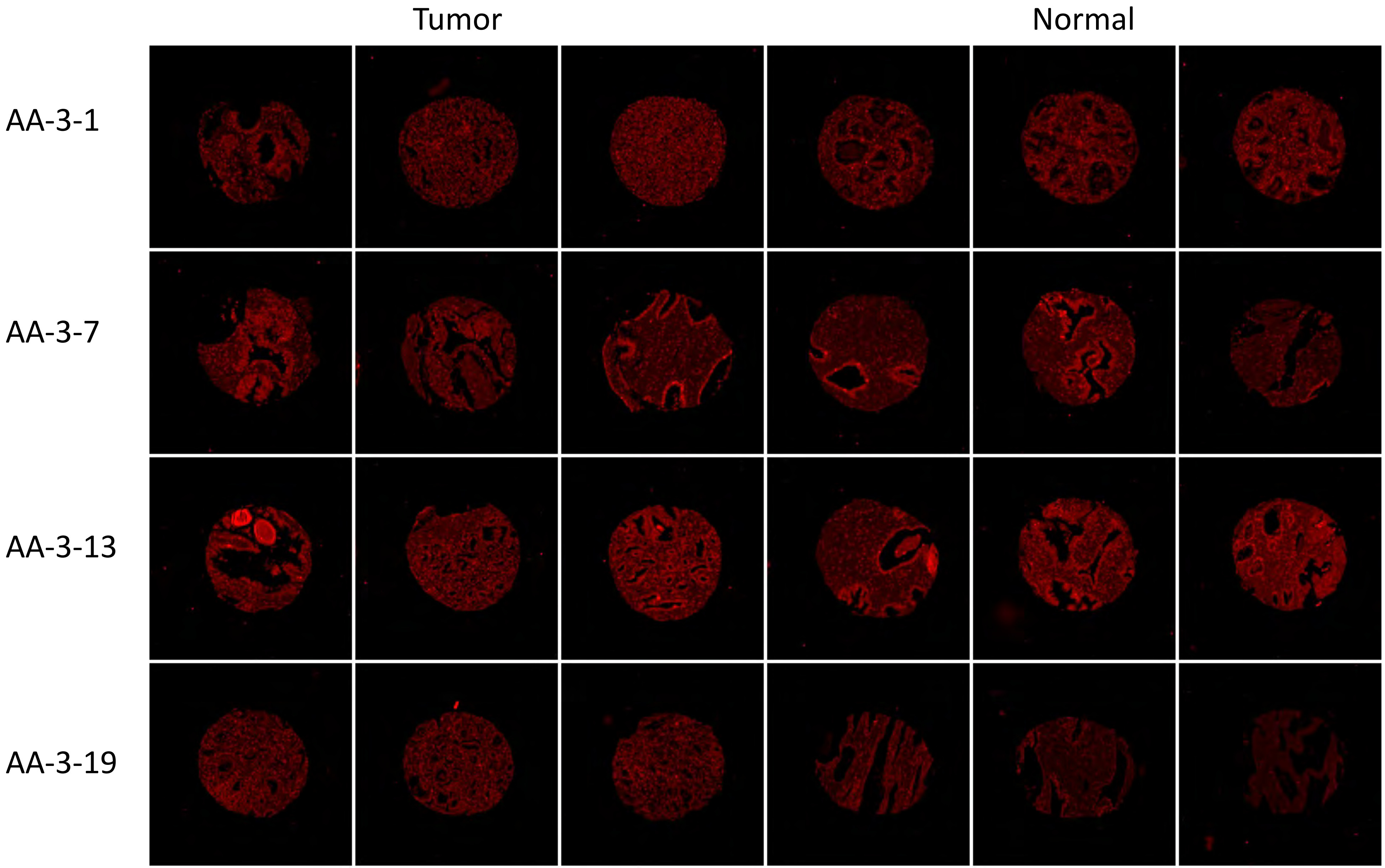

Row 3- oxRADD

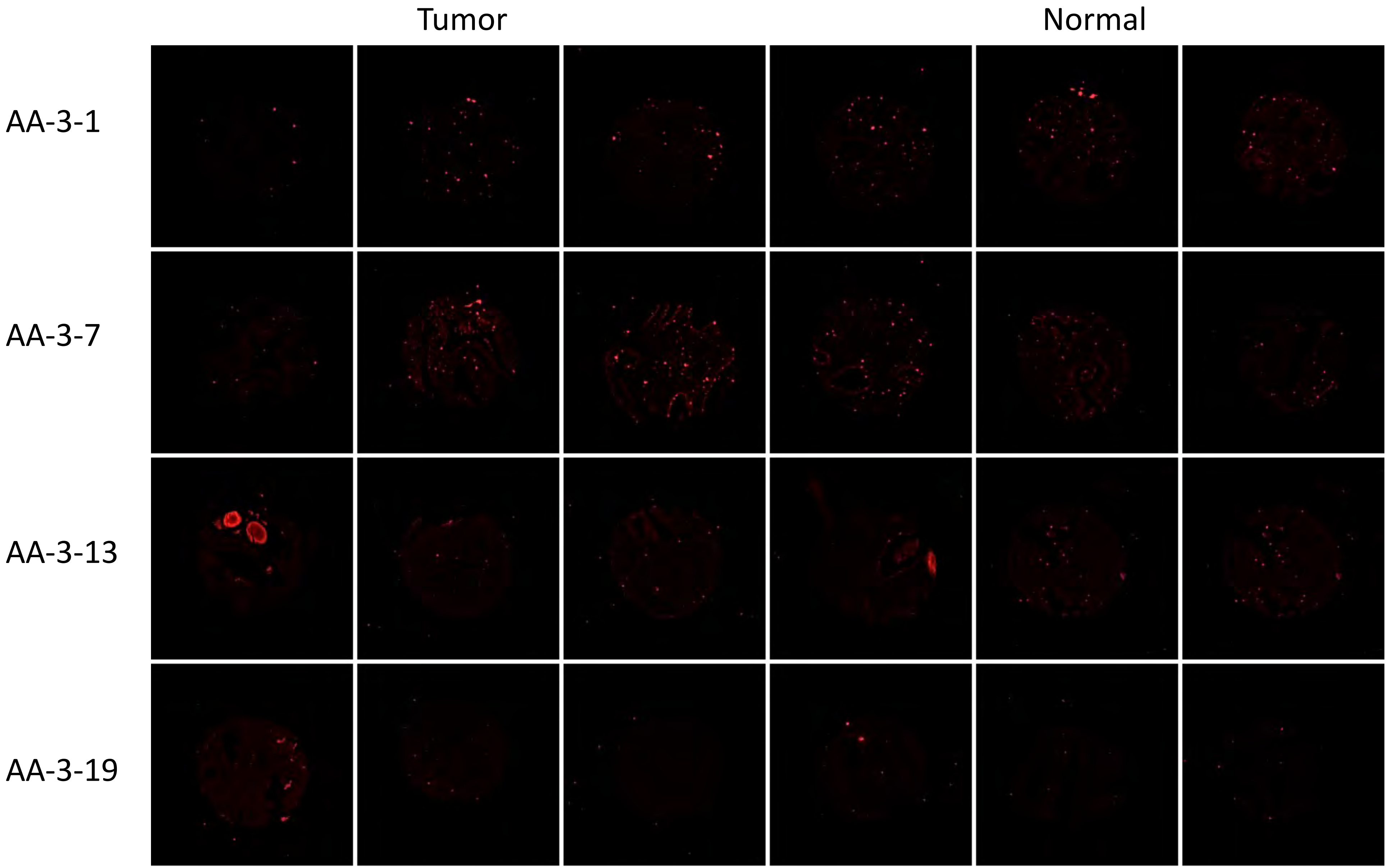

Row 3- UDG

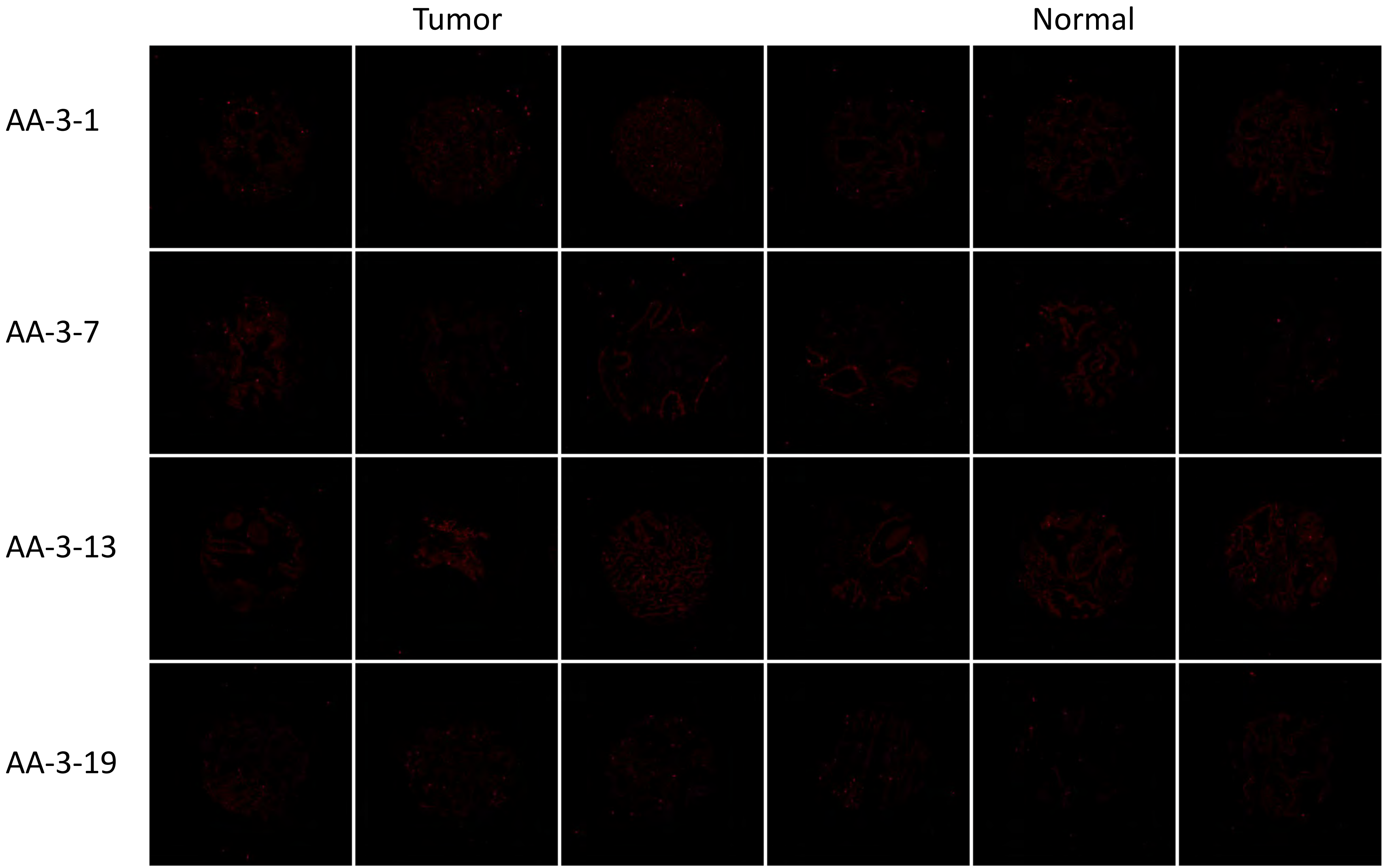

Row 3- T4PDG

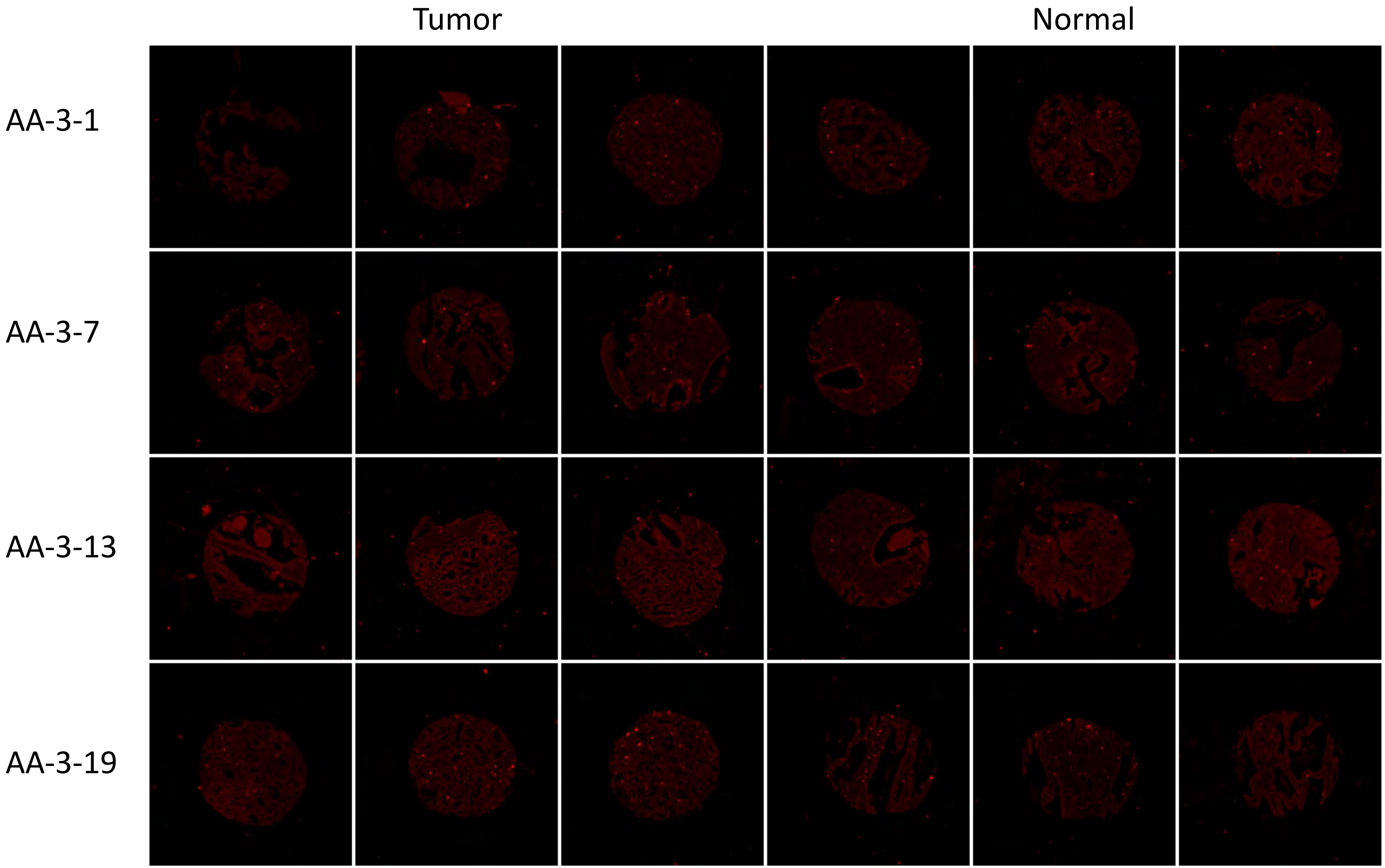

Row 3- XRCC1

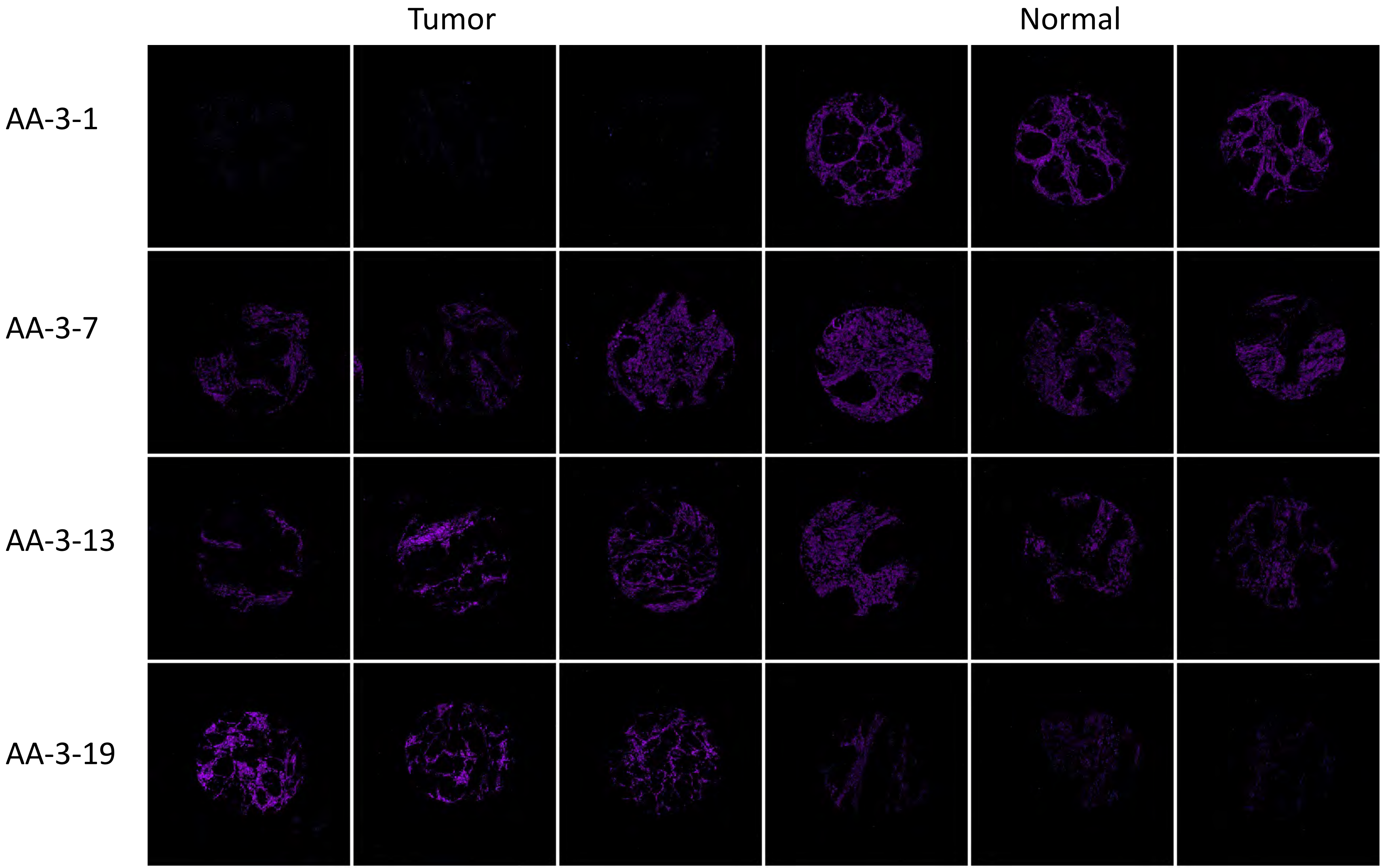

Row 3- PARP1

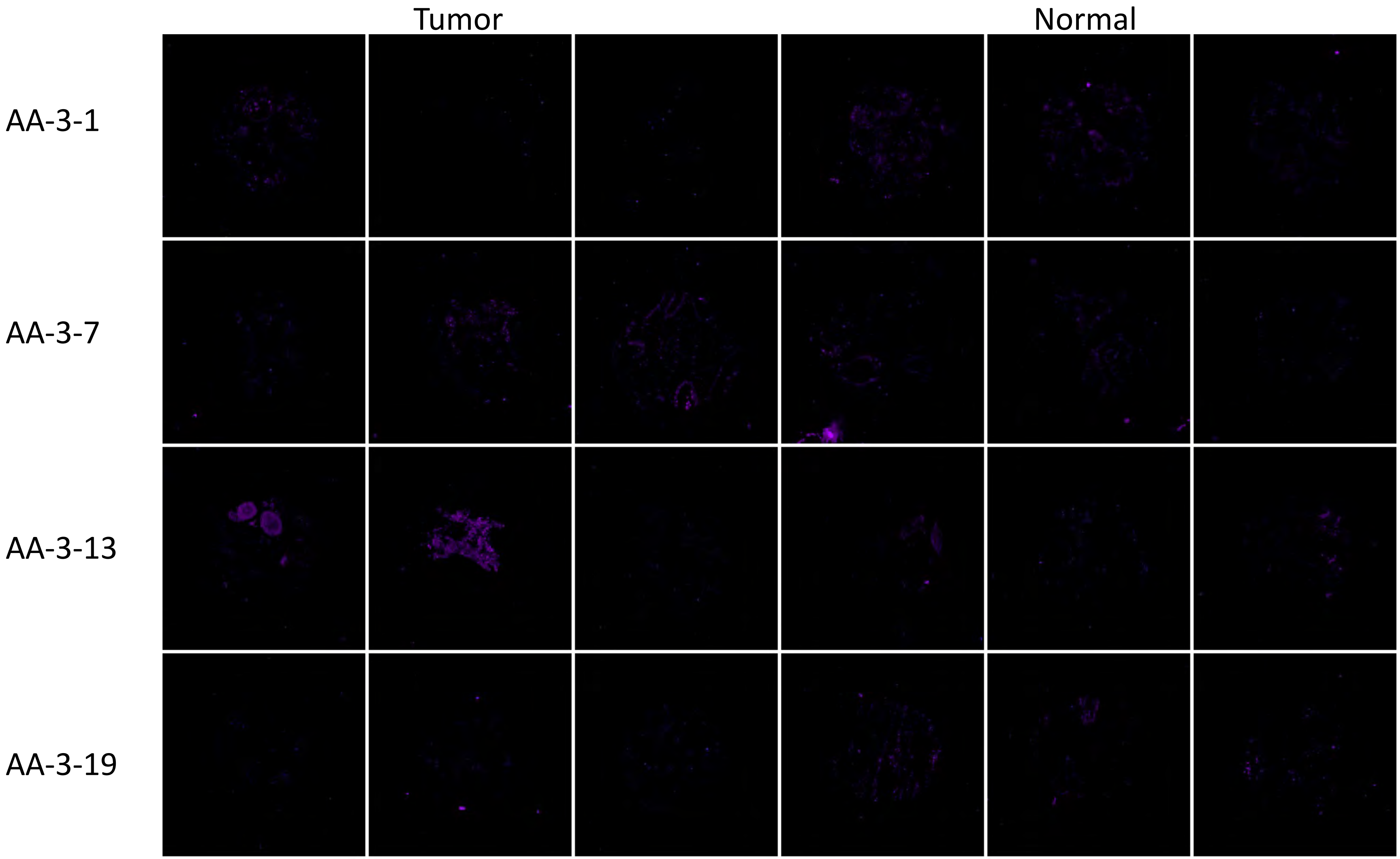

Row 3- UNG

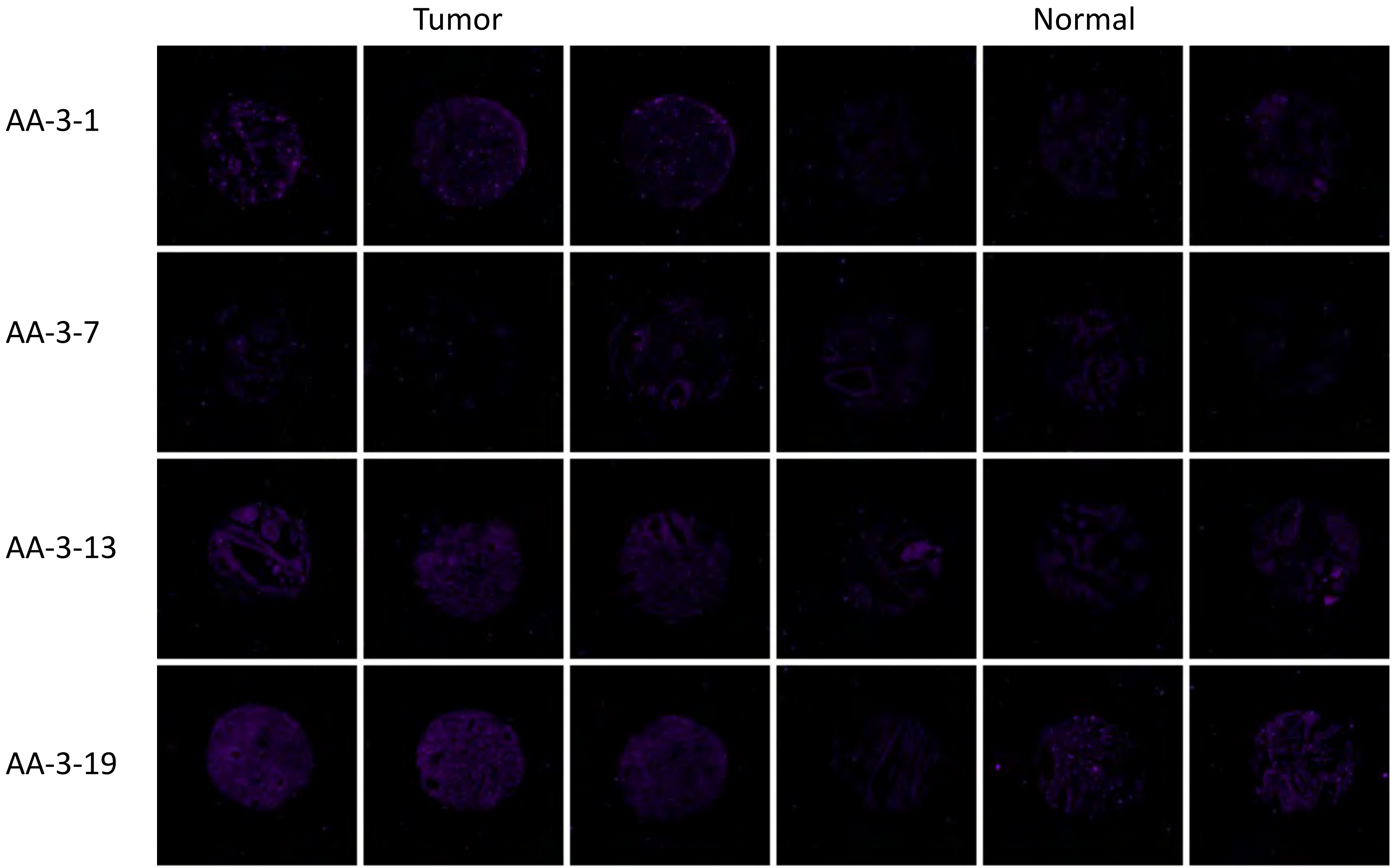

Row 4- Full RADD

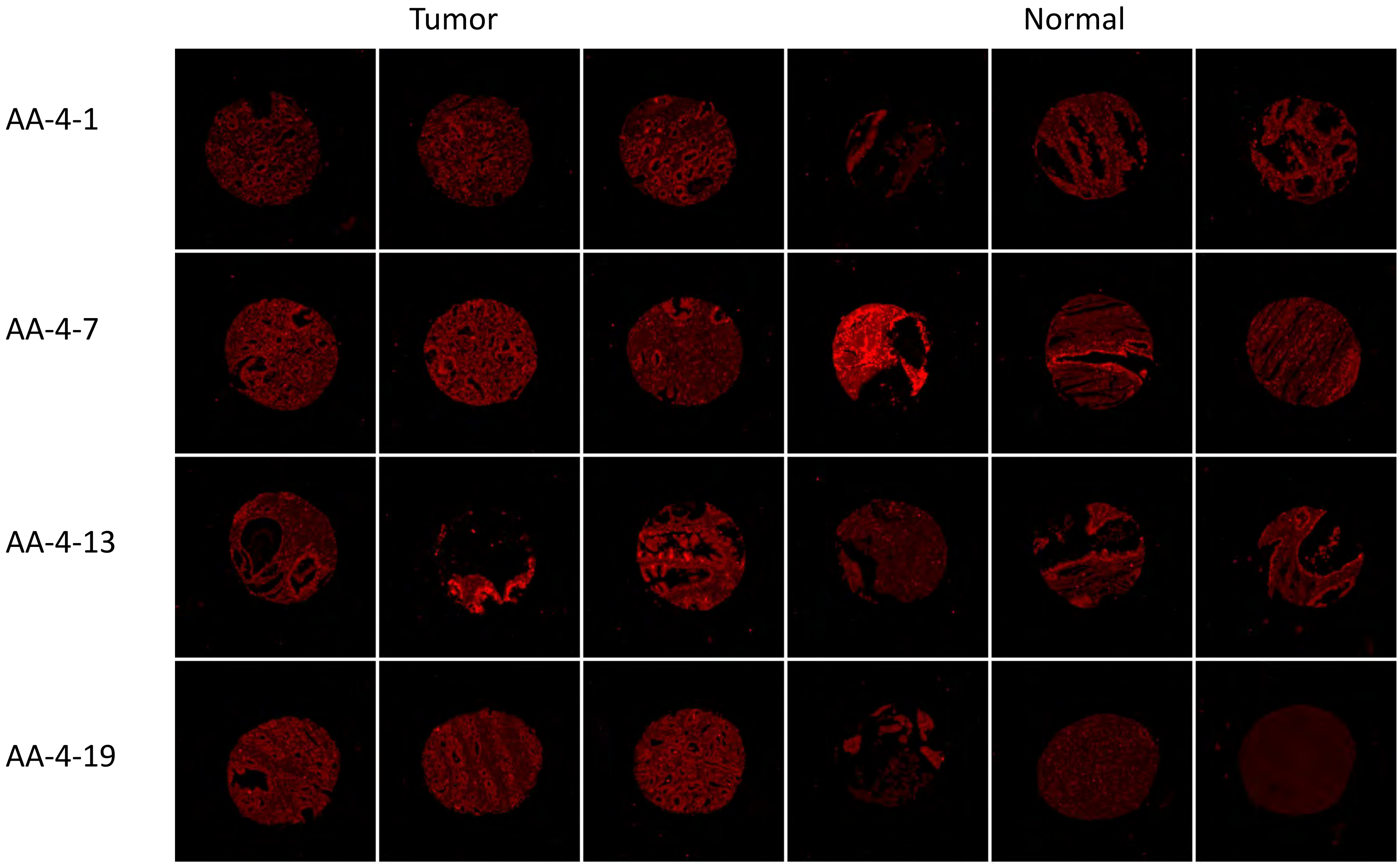

Row 4- oxRADD

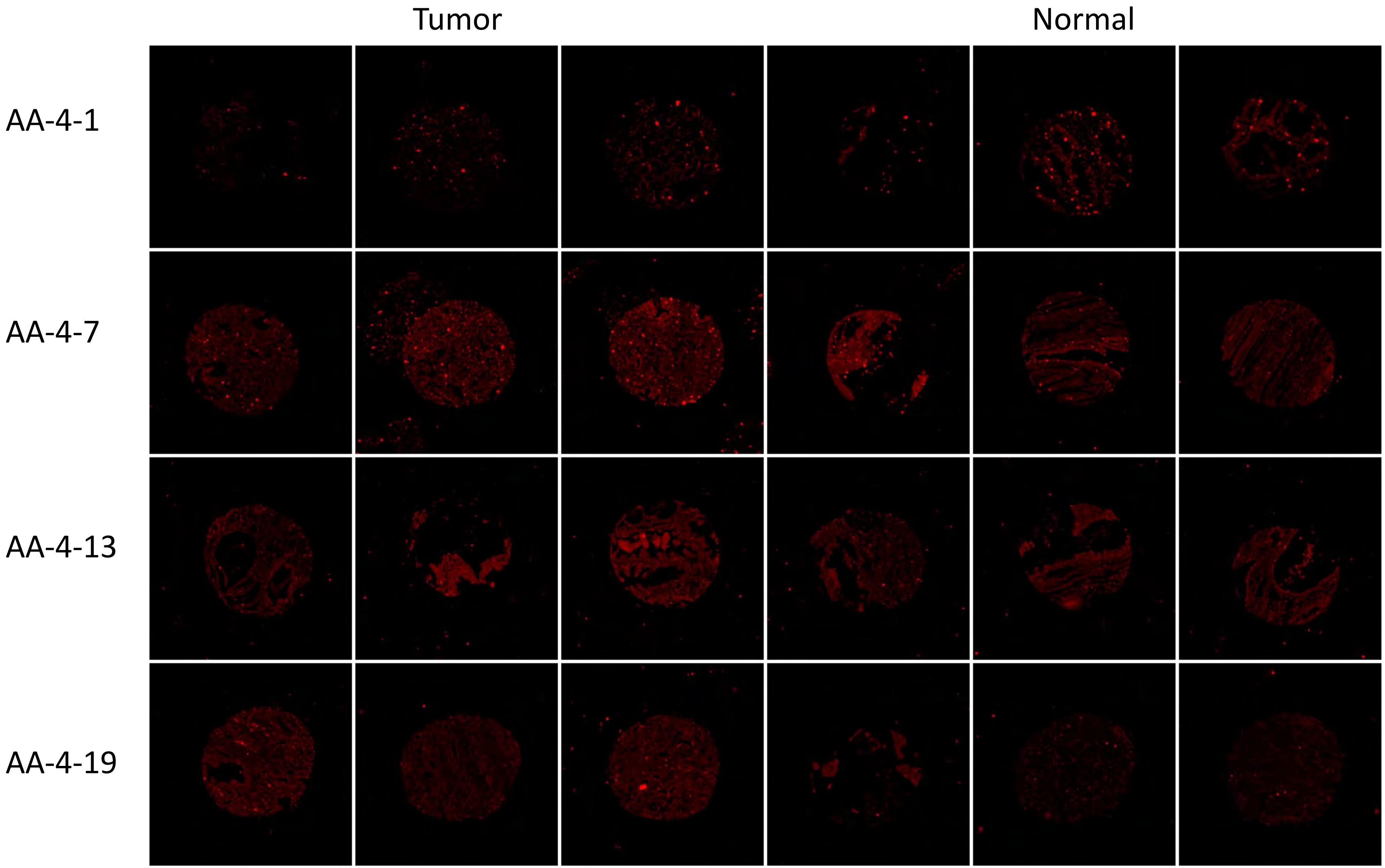

Row 4- UDG

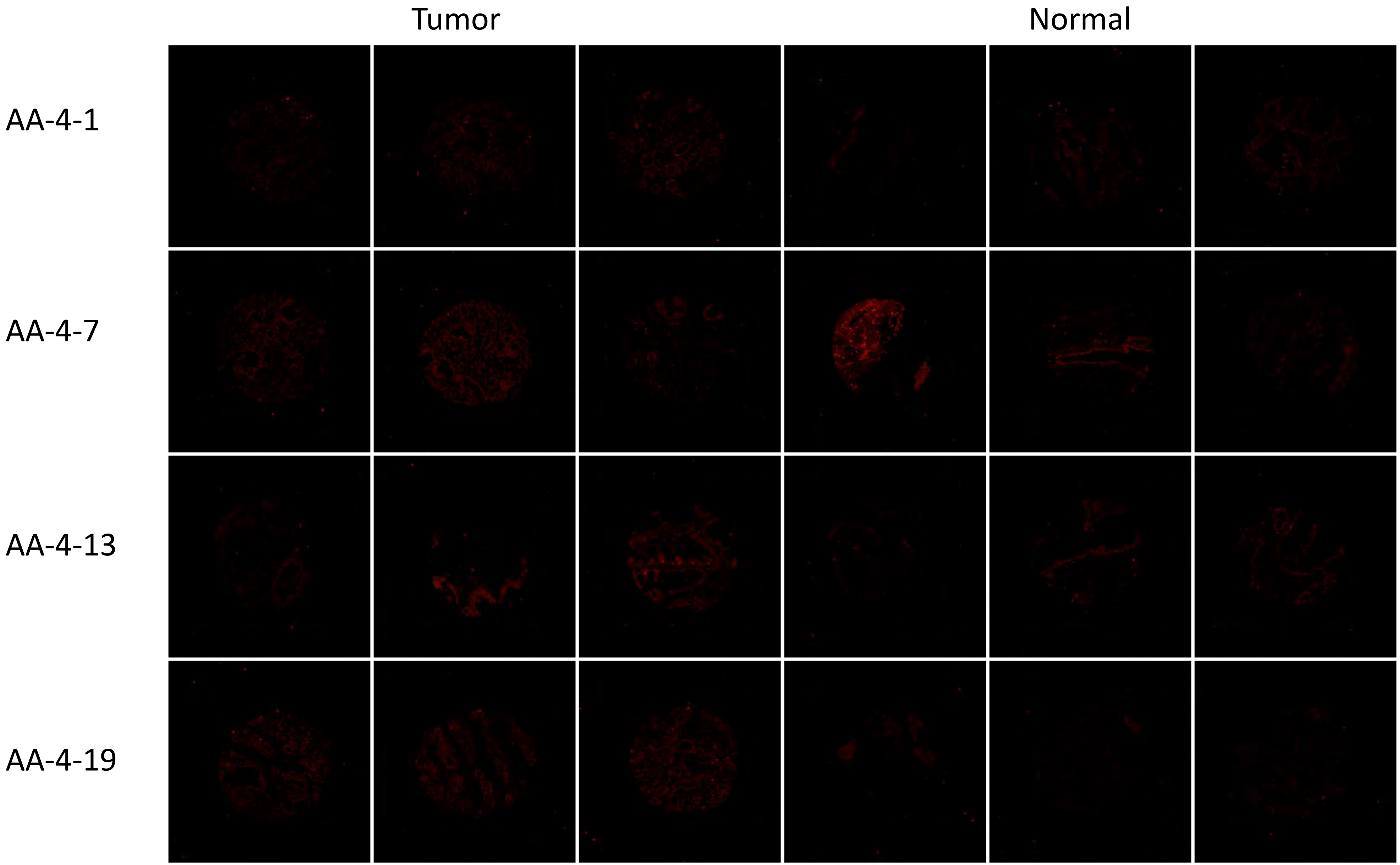

Row 4- T4PDG

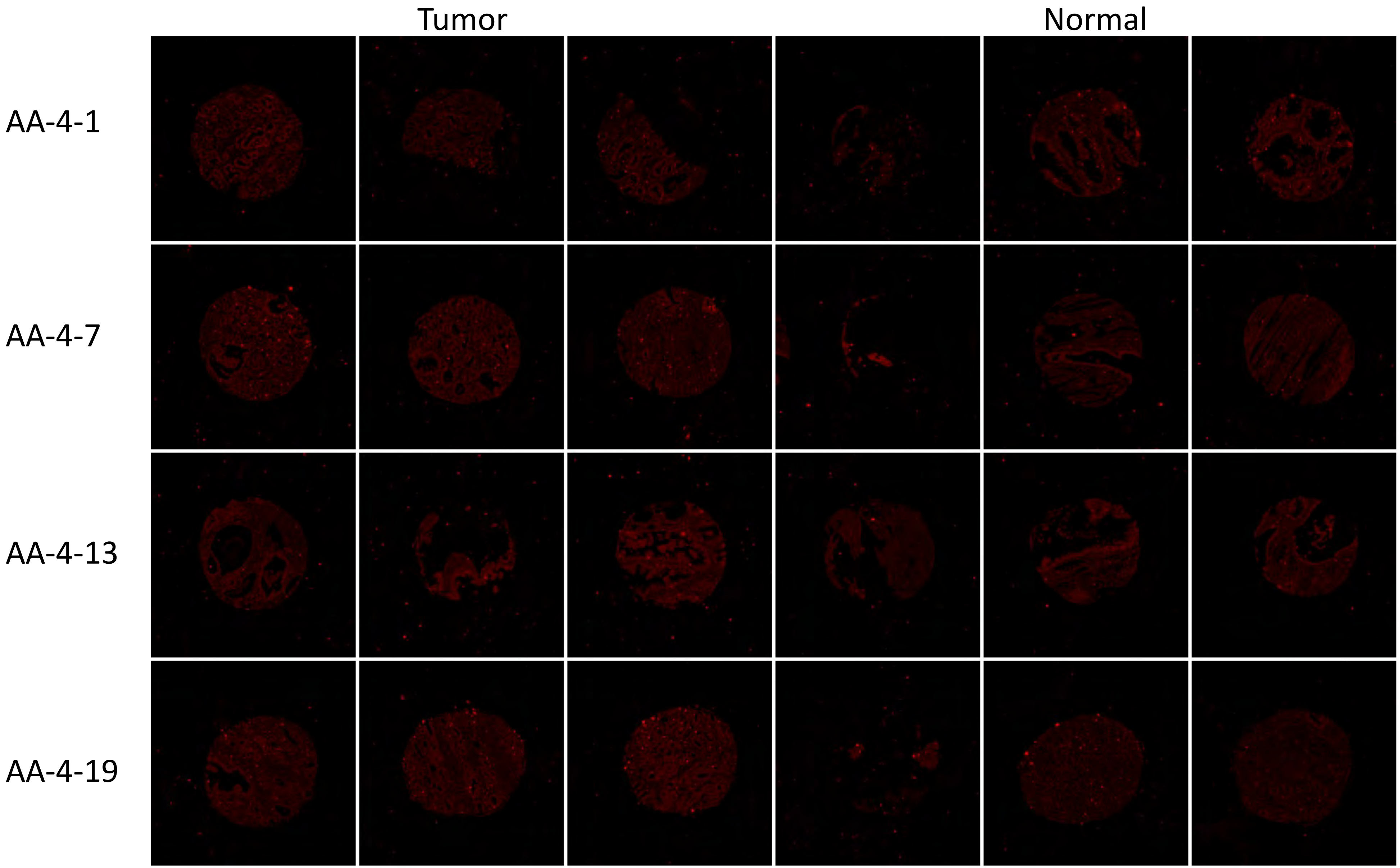

Row 4- XRCC1

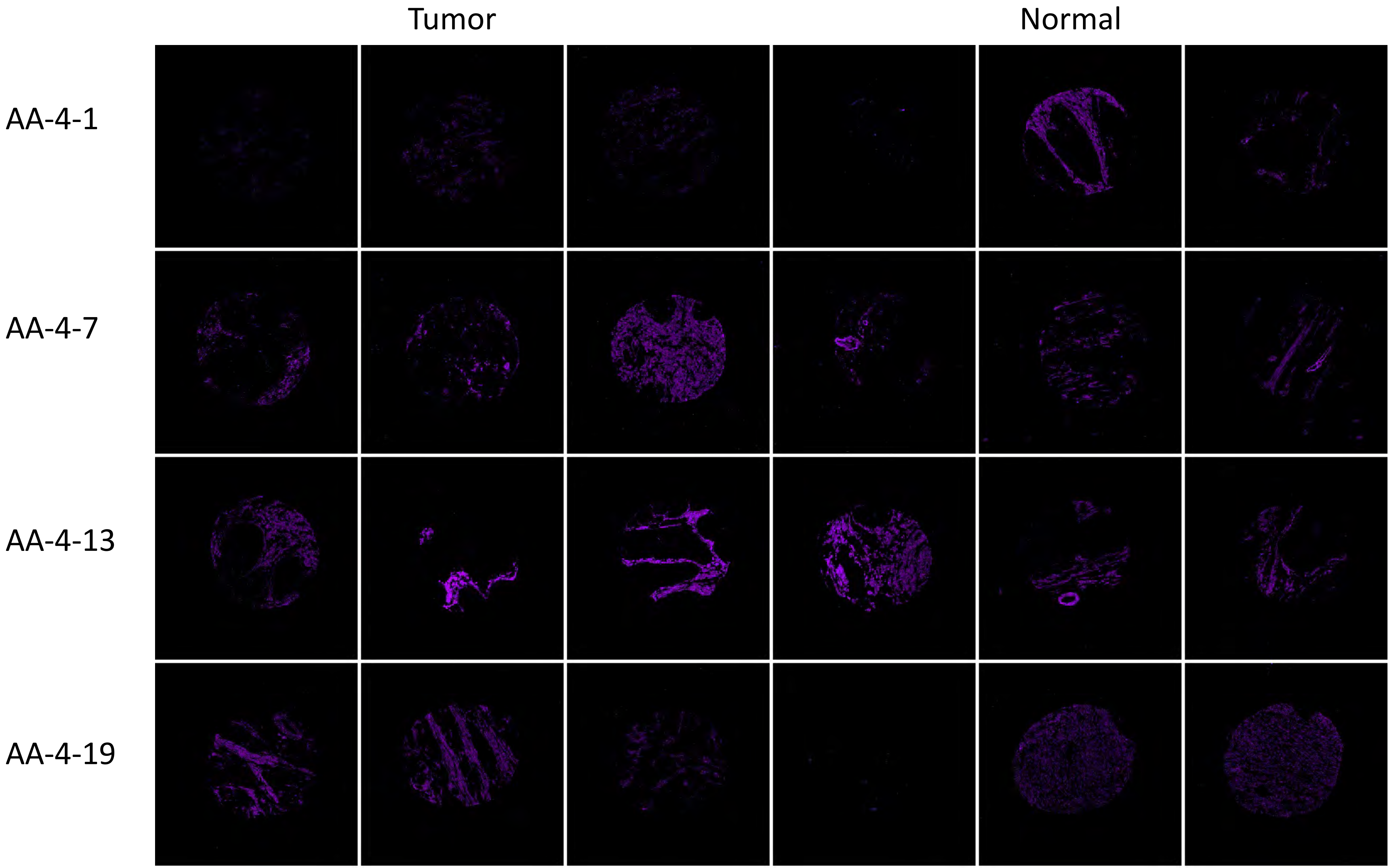

Row 4- PARP1

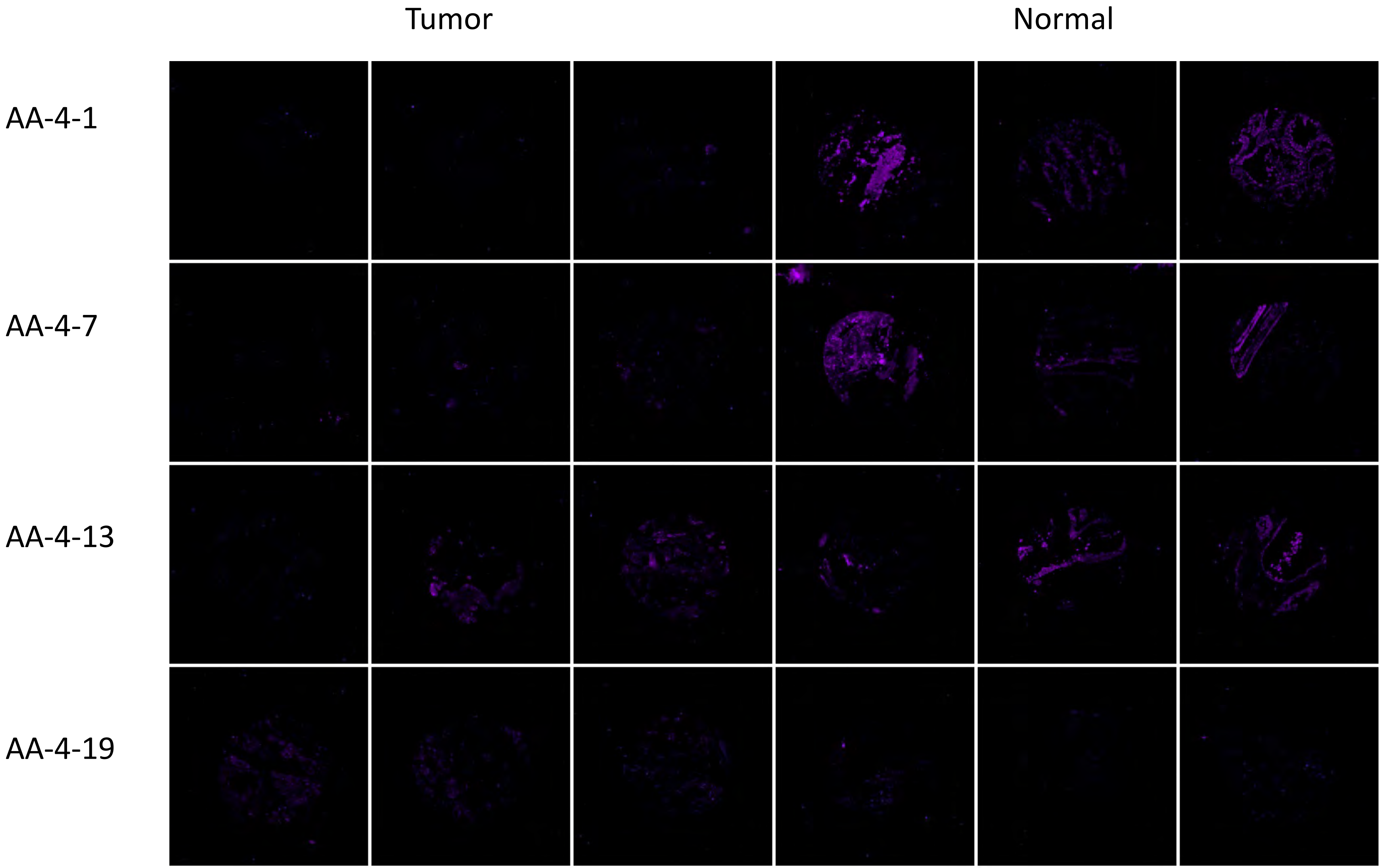

Row 4- UNG

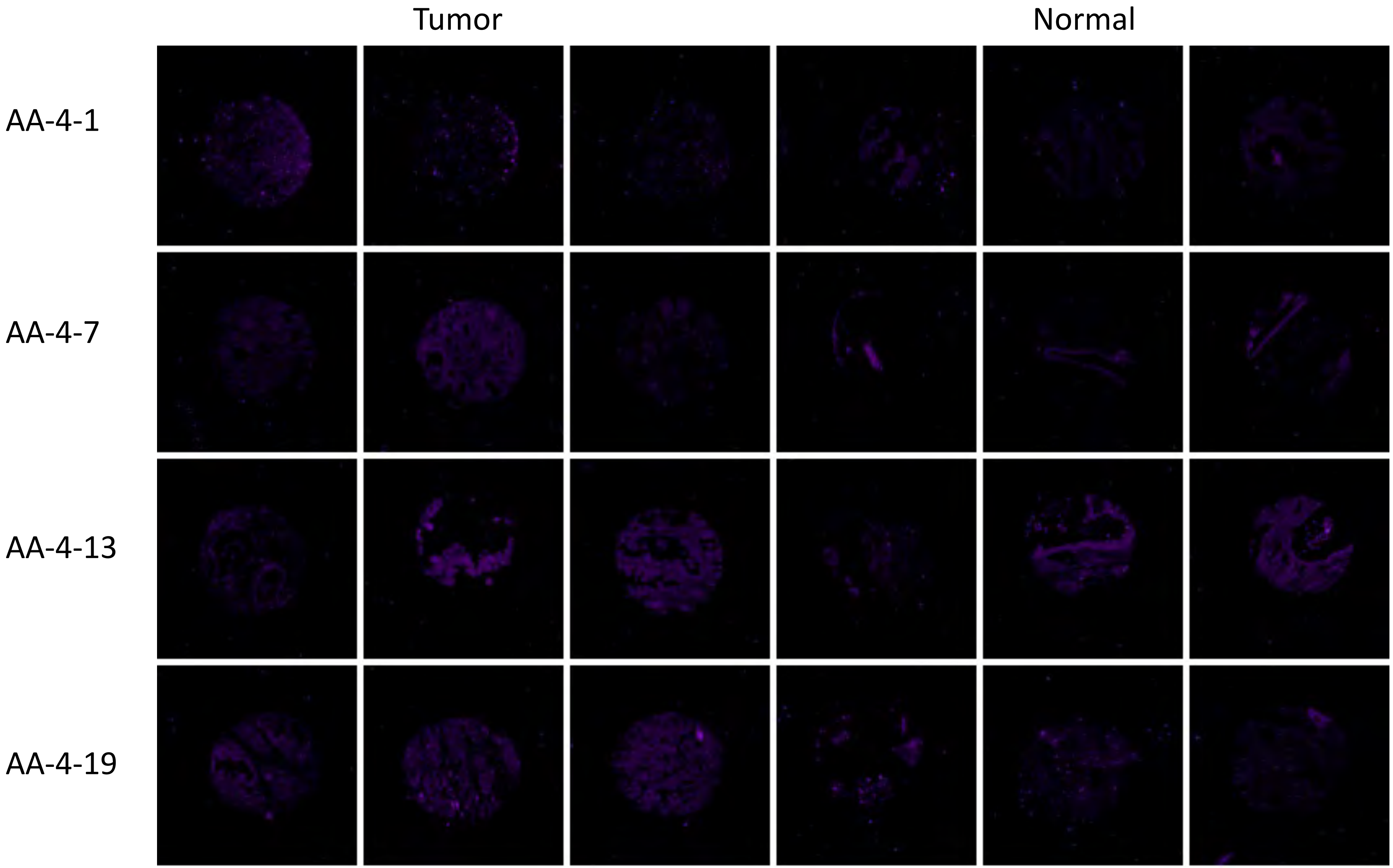

Row 5- Full RADD

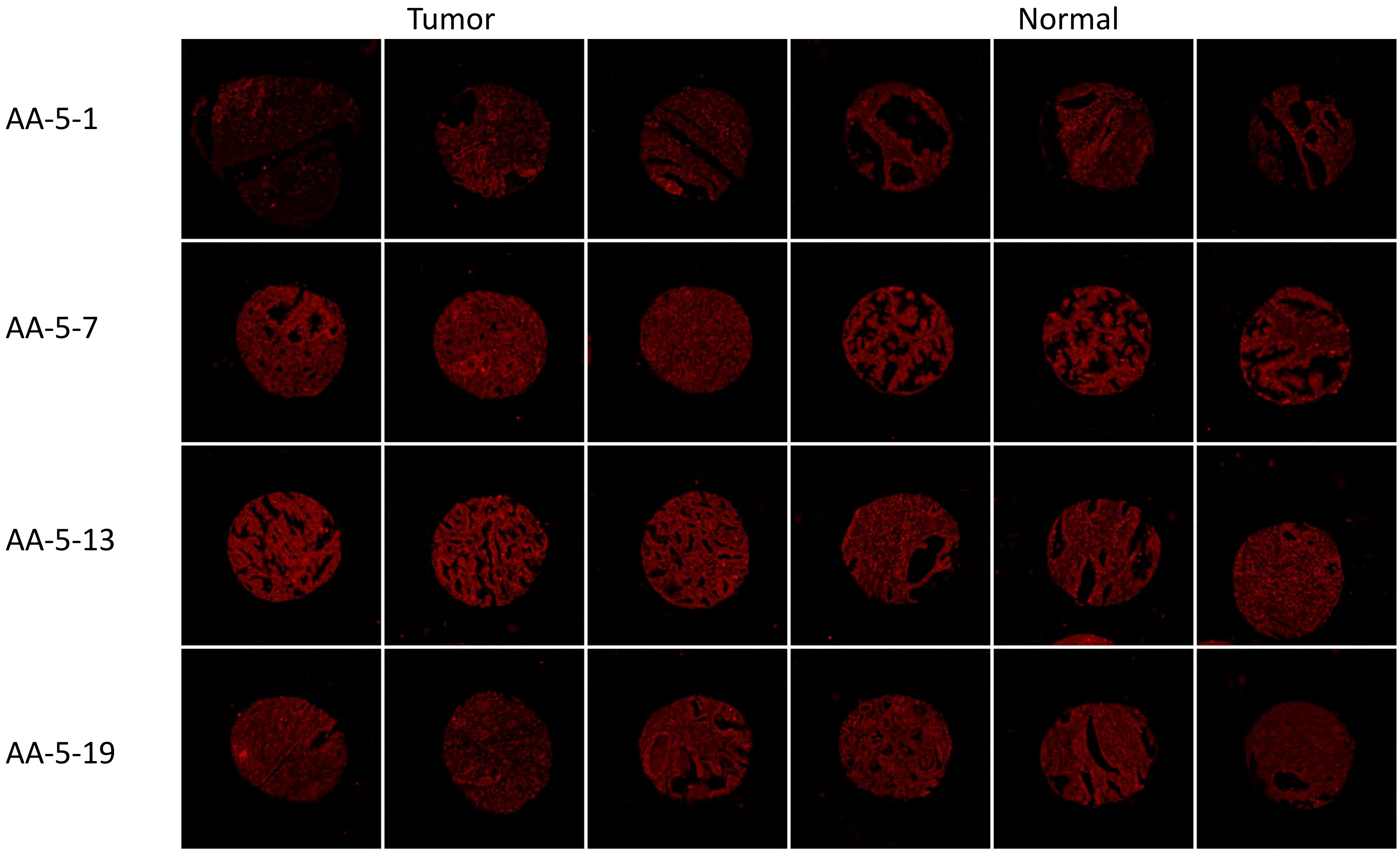

Row 5- oxRADD

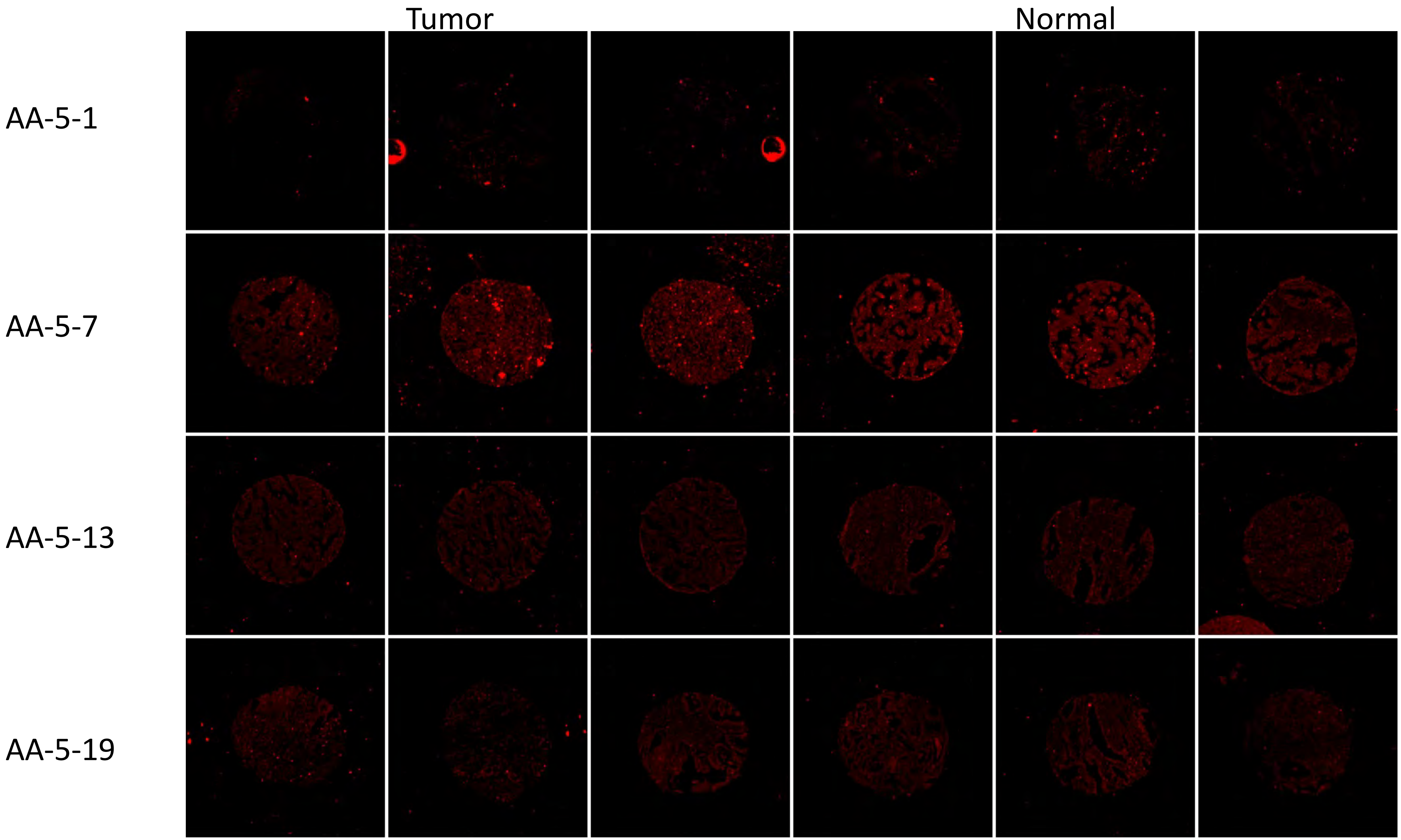

Row 5- UDG

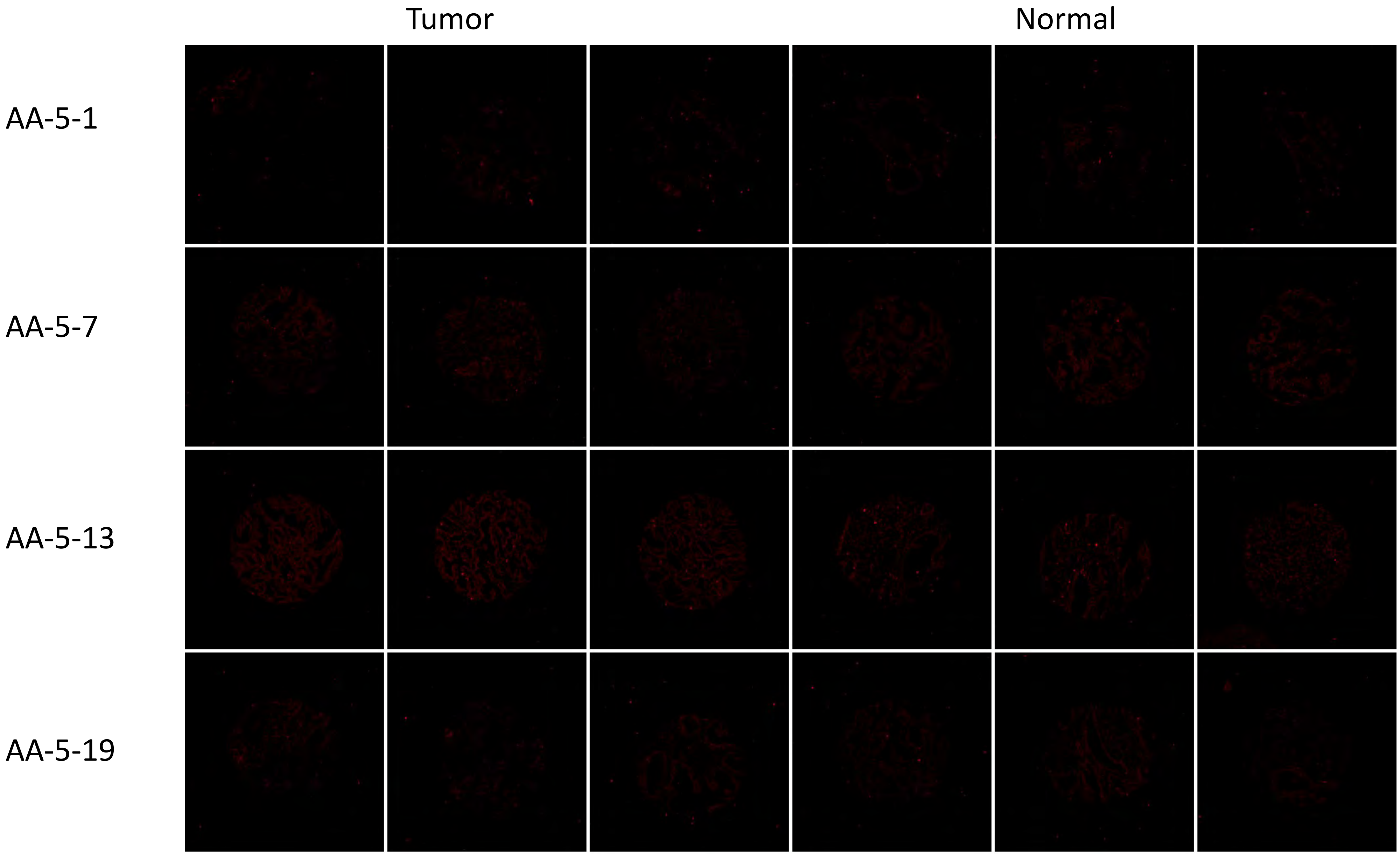

Row 5- T4PDG

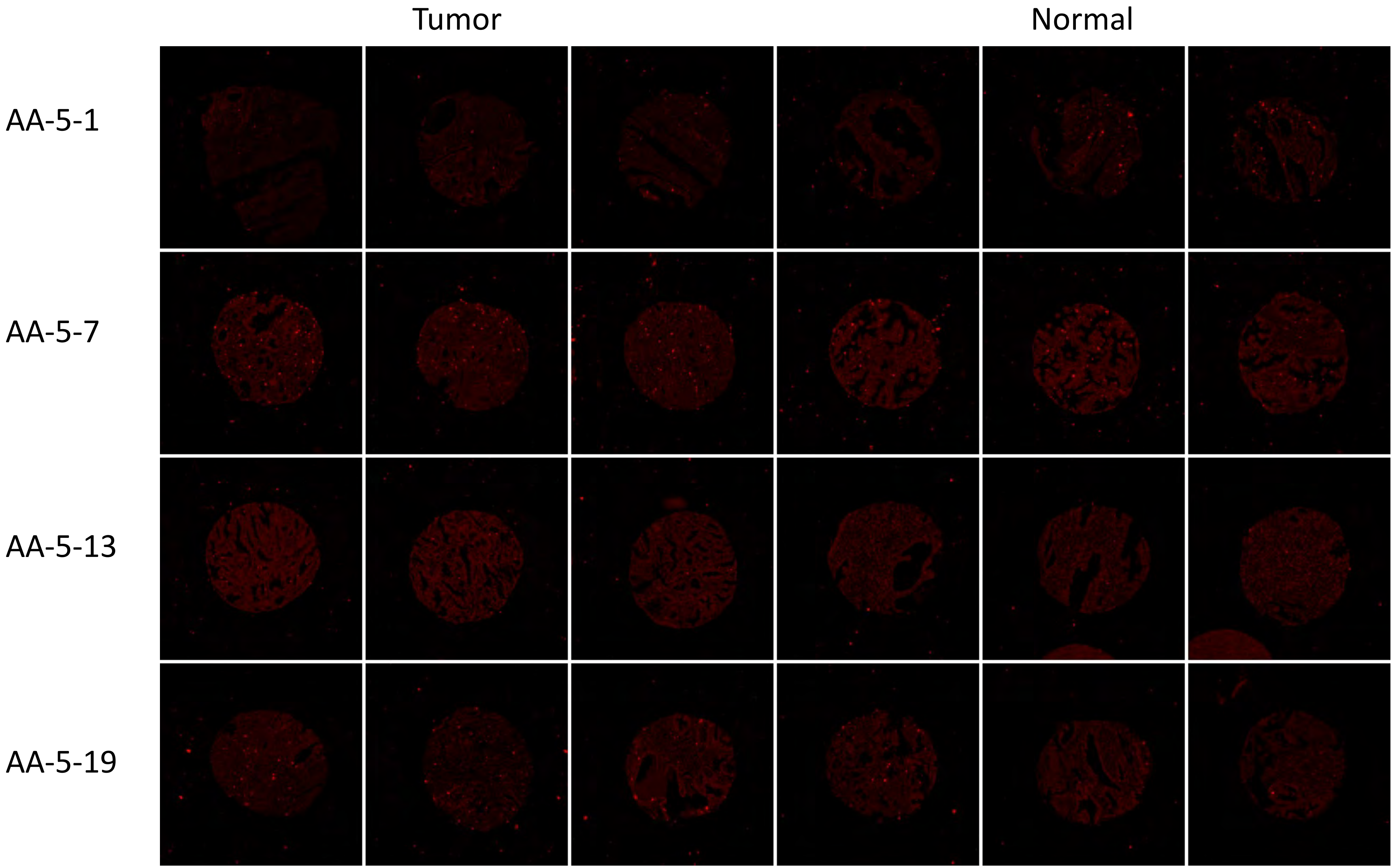

Row 5- XRCC1

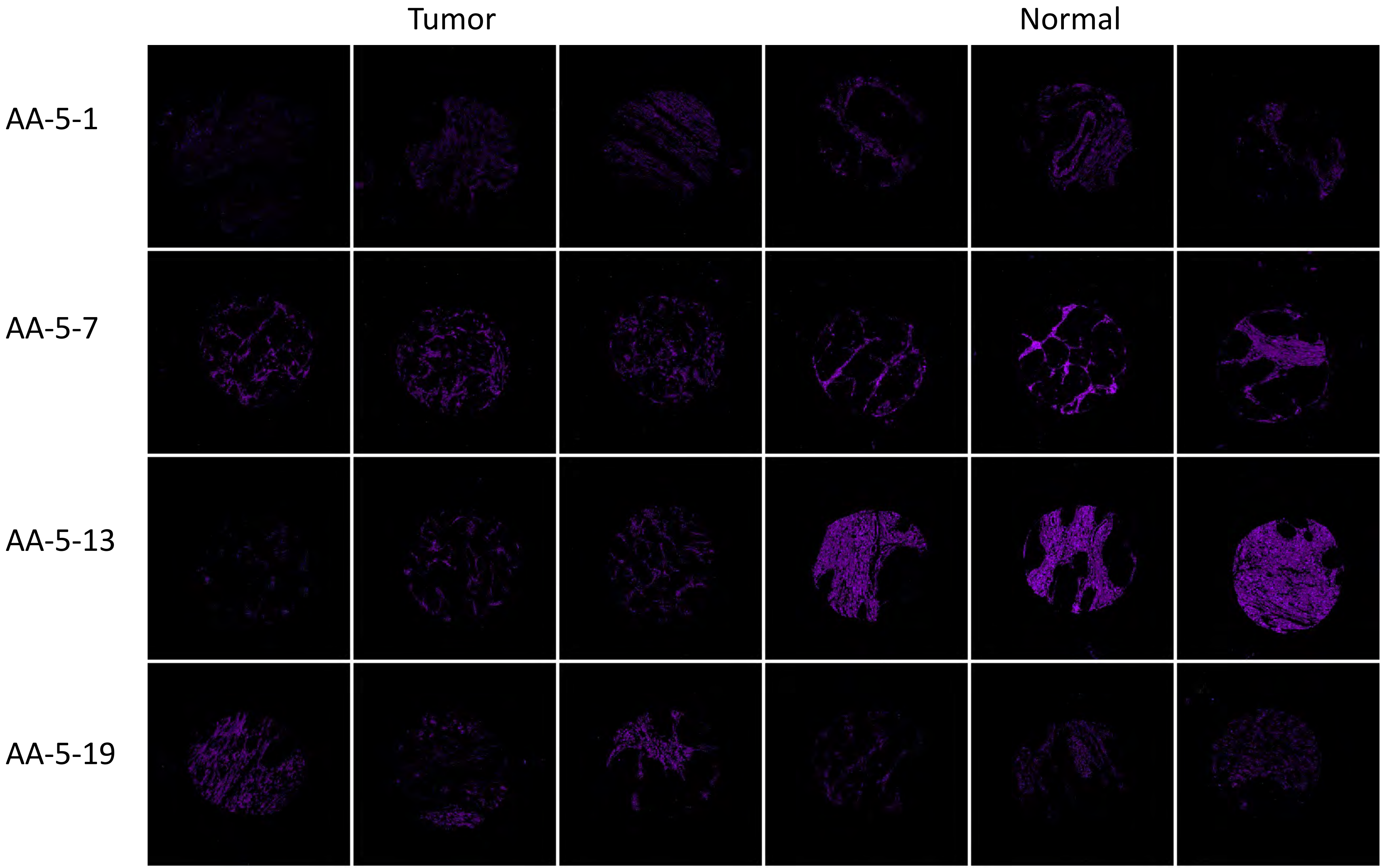

Row 5- PARP1

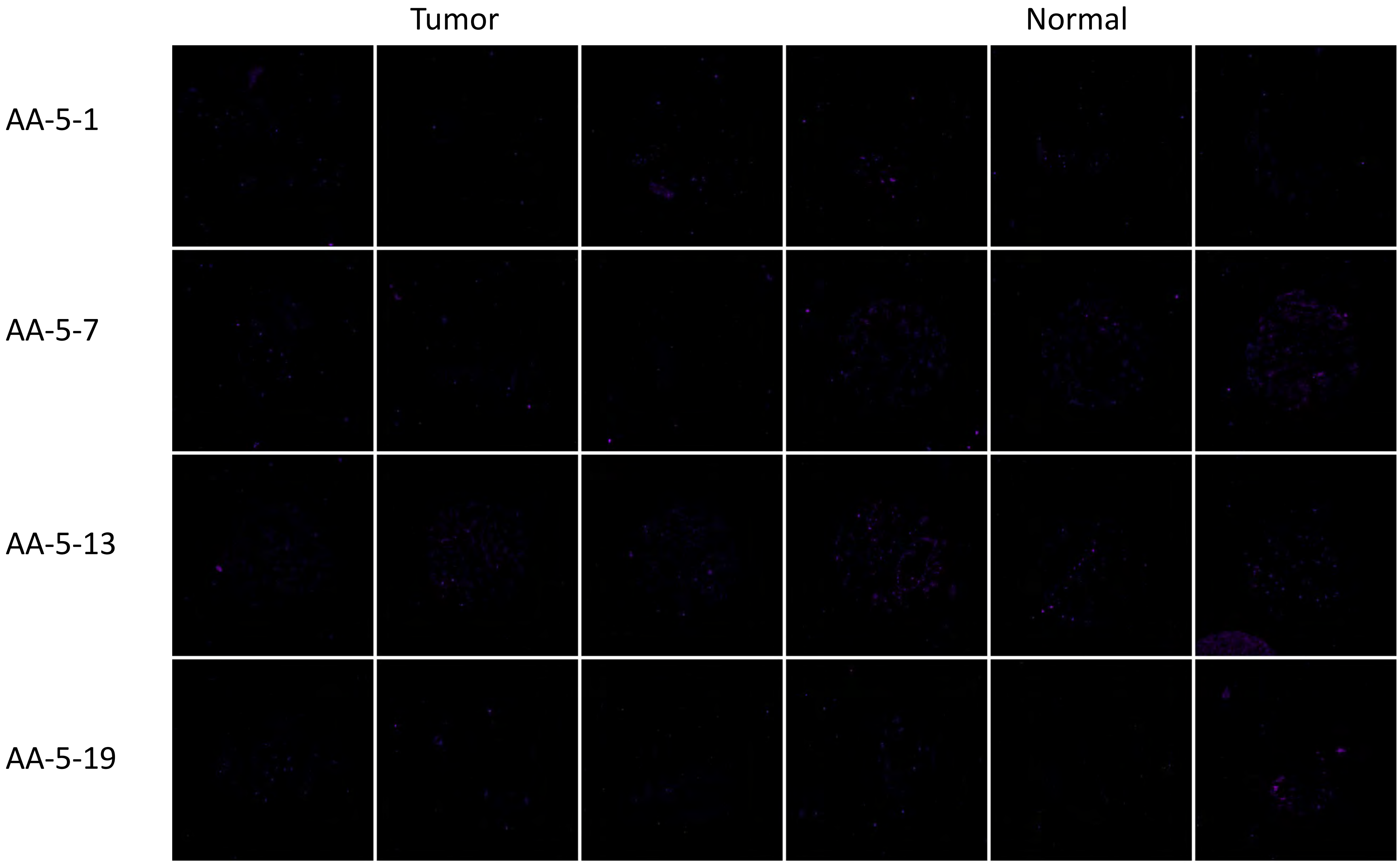

Row 5- UNG

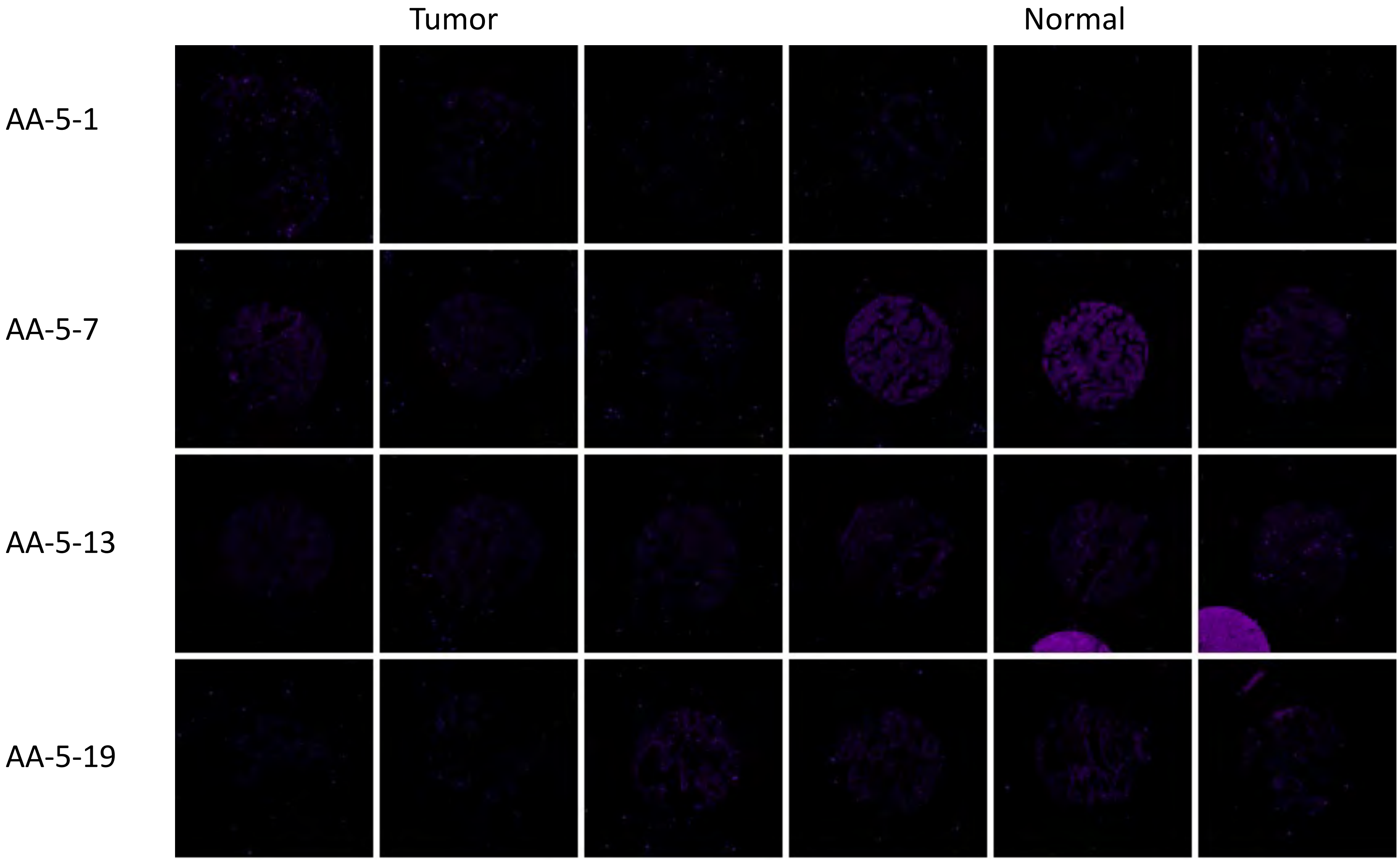

Row 6- Full RADD

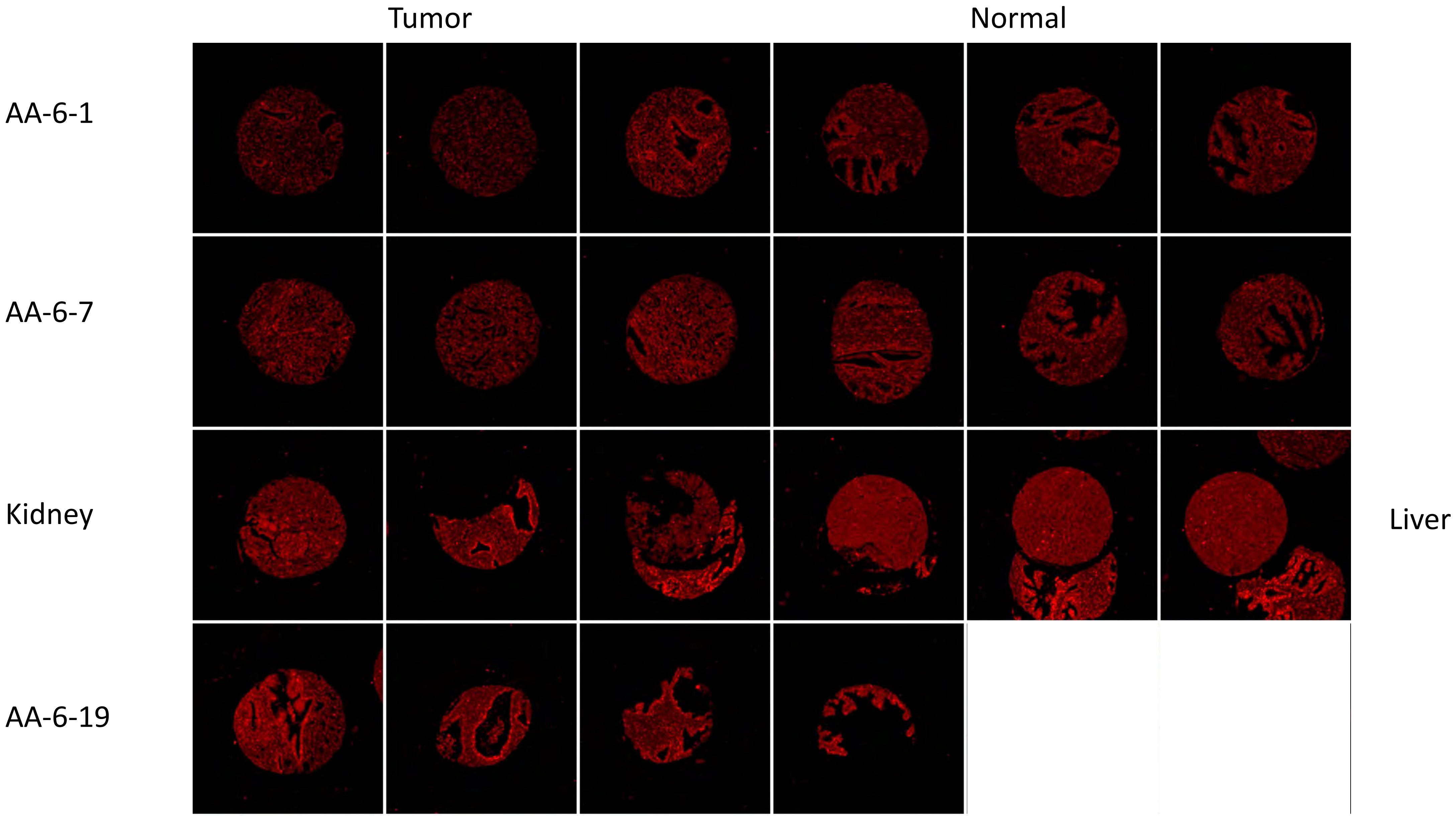

Row 6- oxRADD

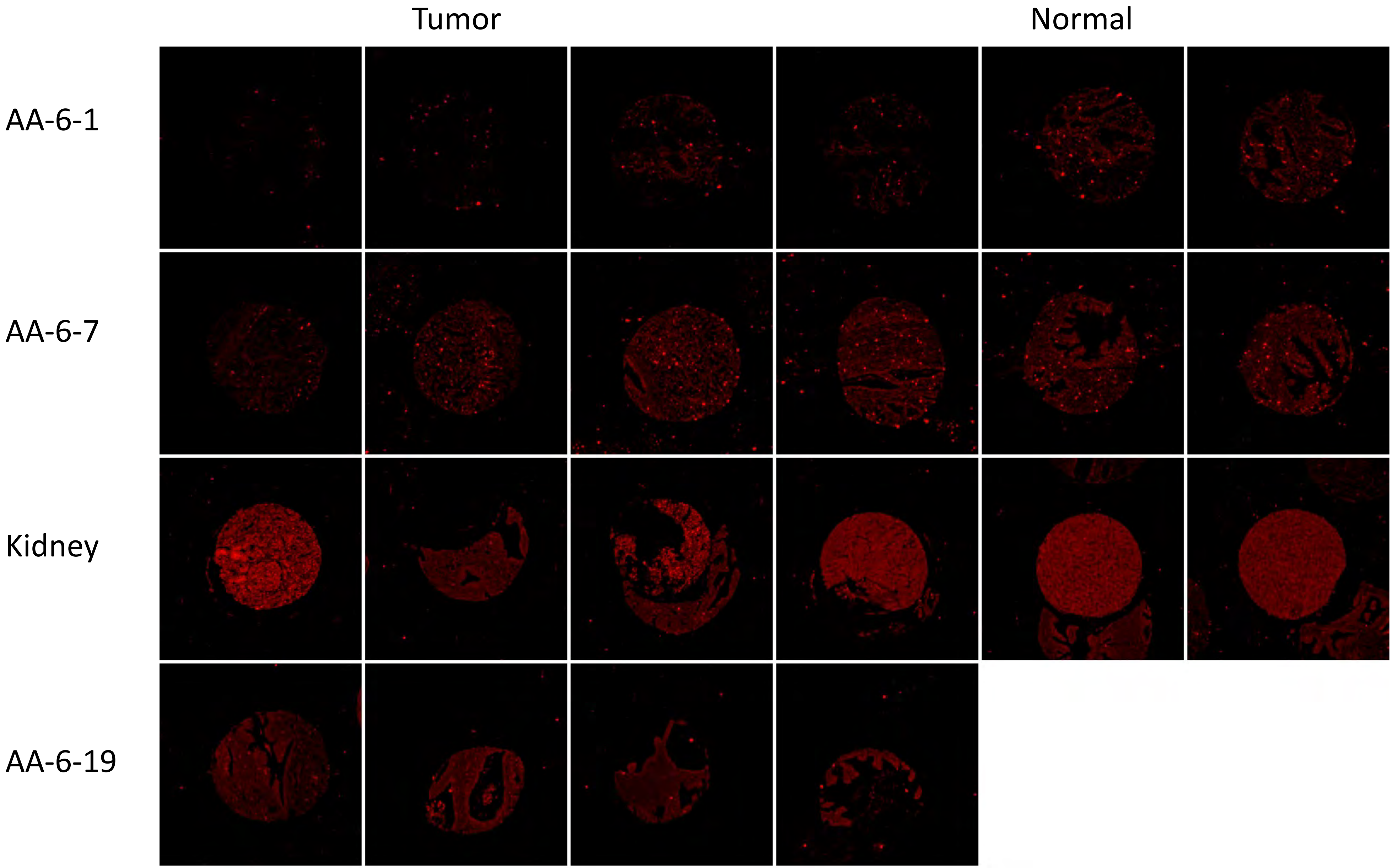

Row 6- UDG

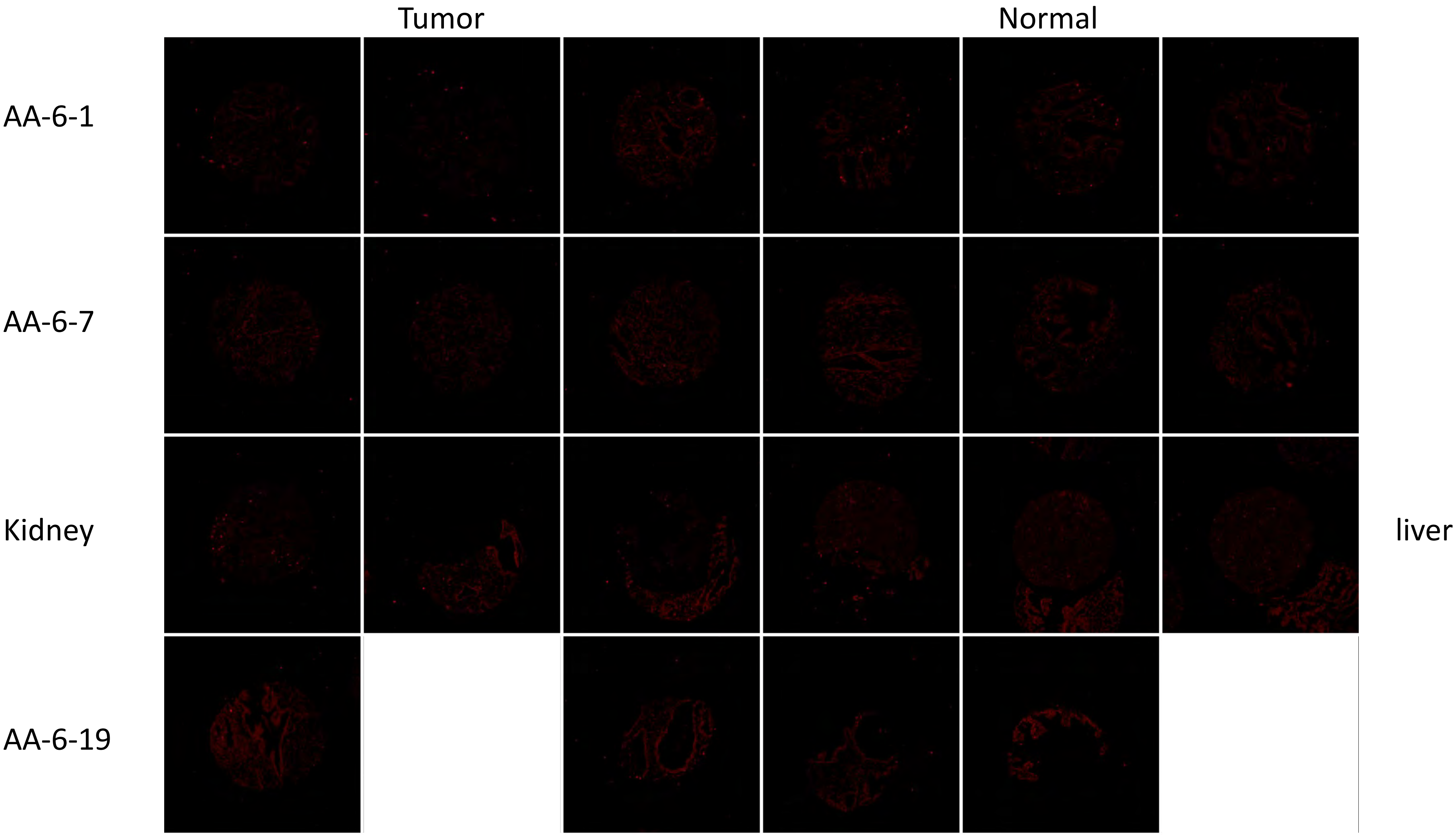

Row 6- T4PDG

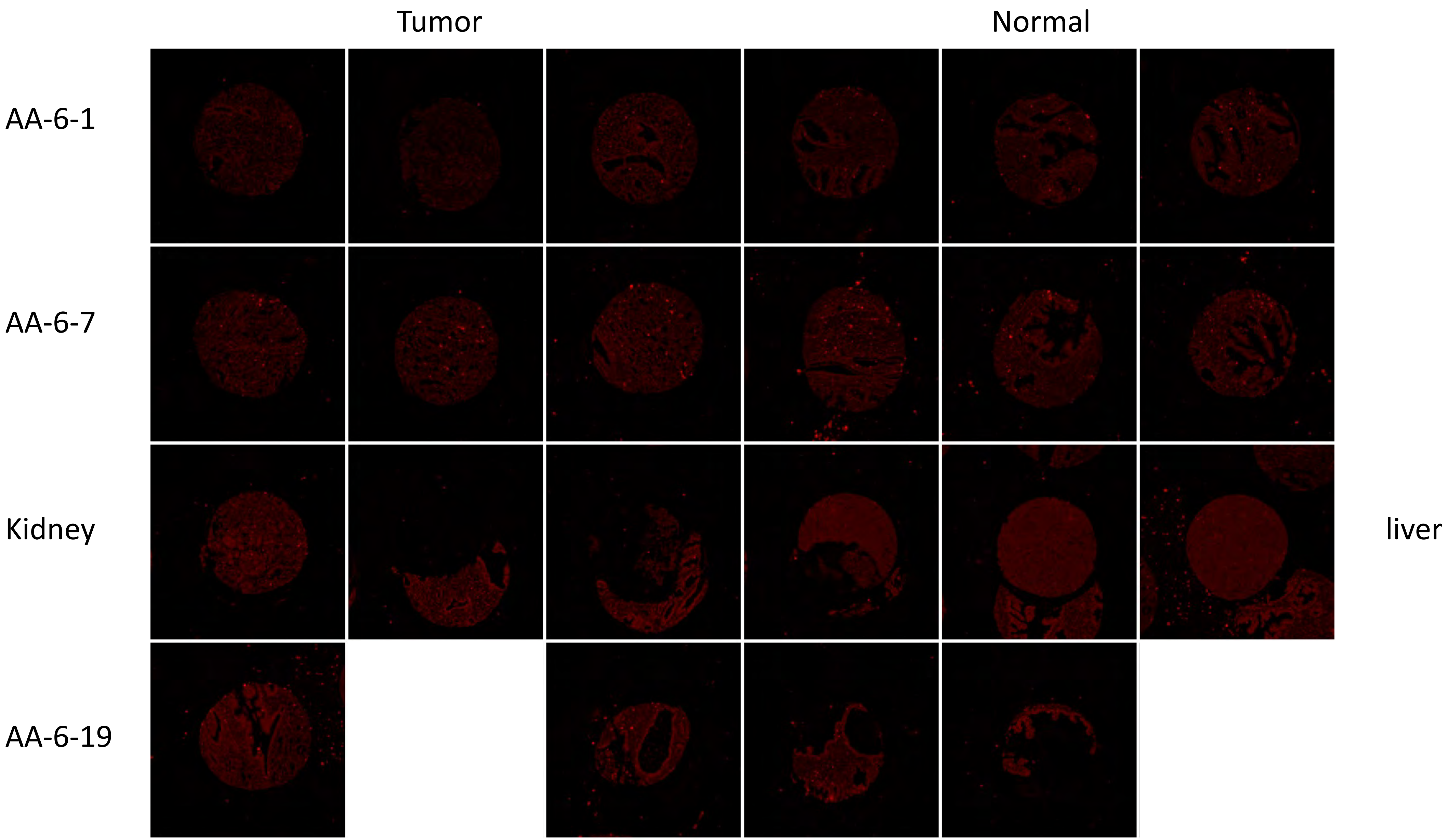

Row 6- XRCC1

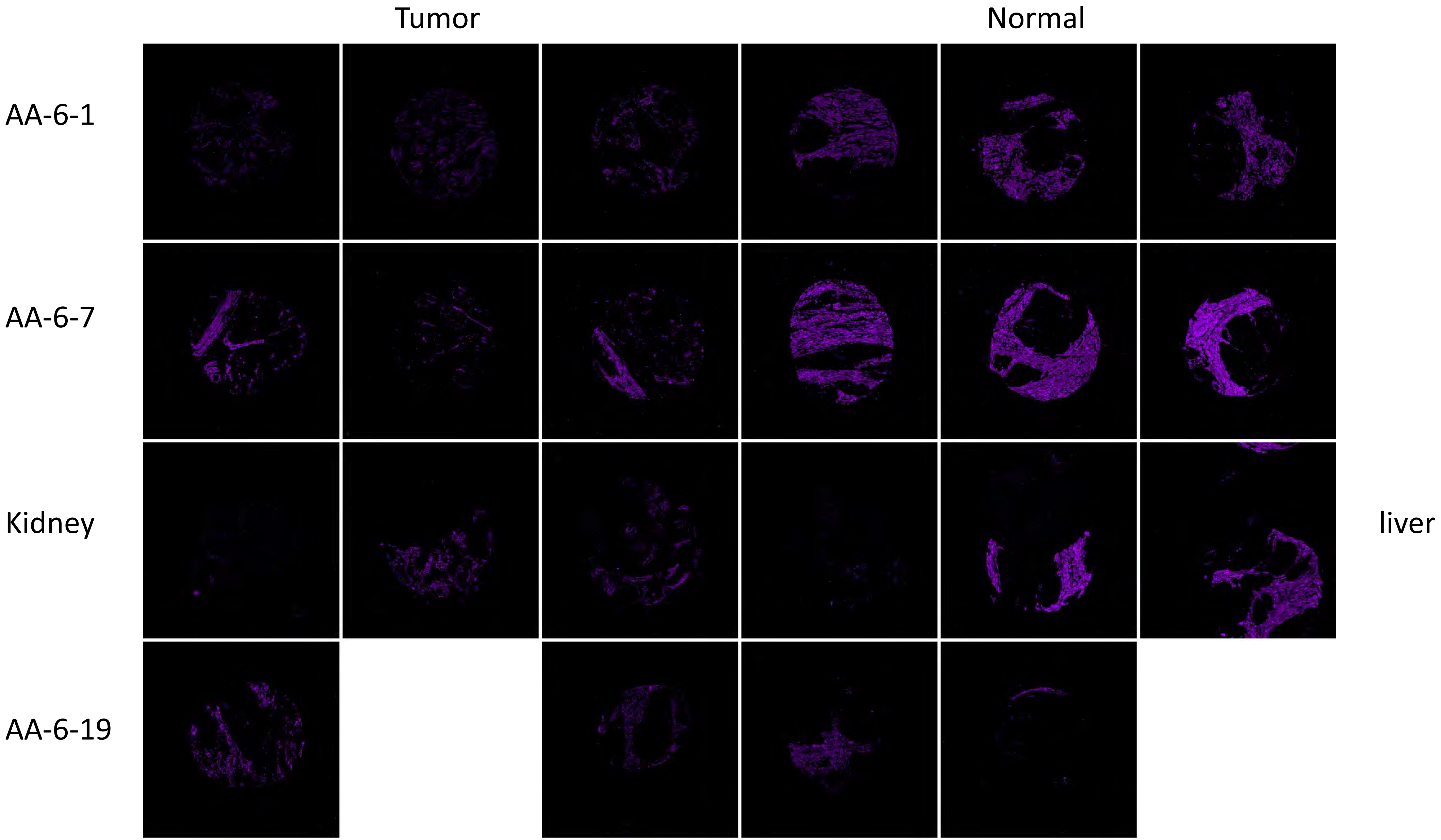

Row 6- PARP1

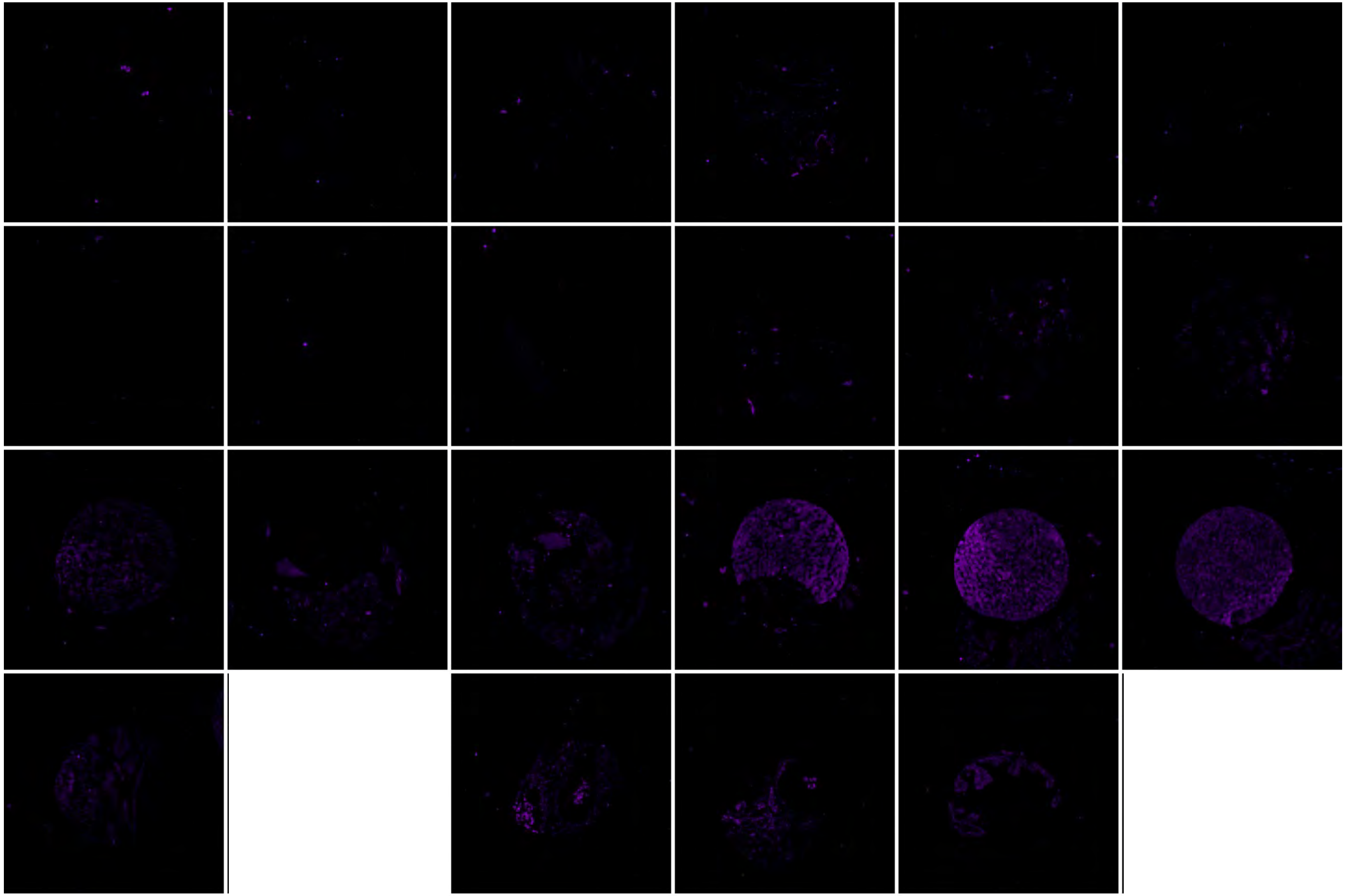

Row 6- UNG

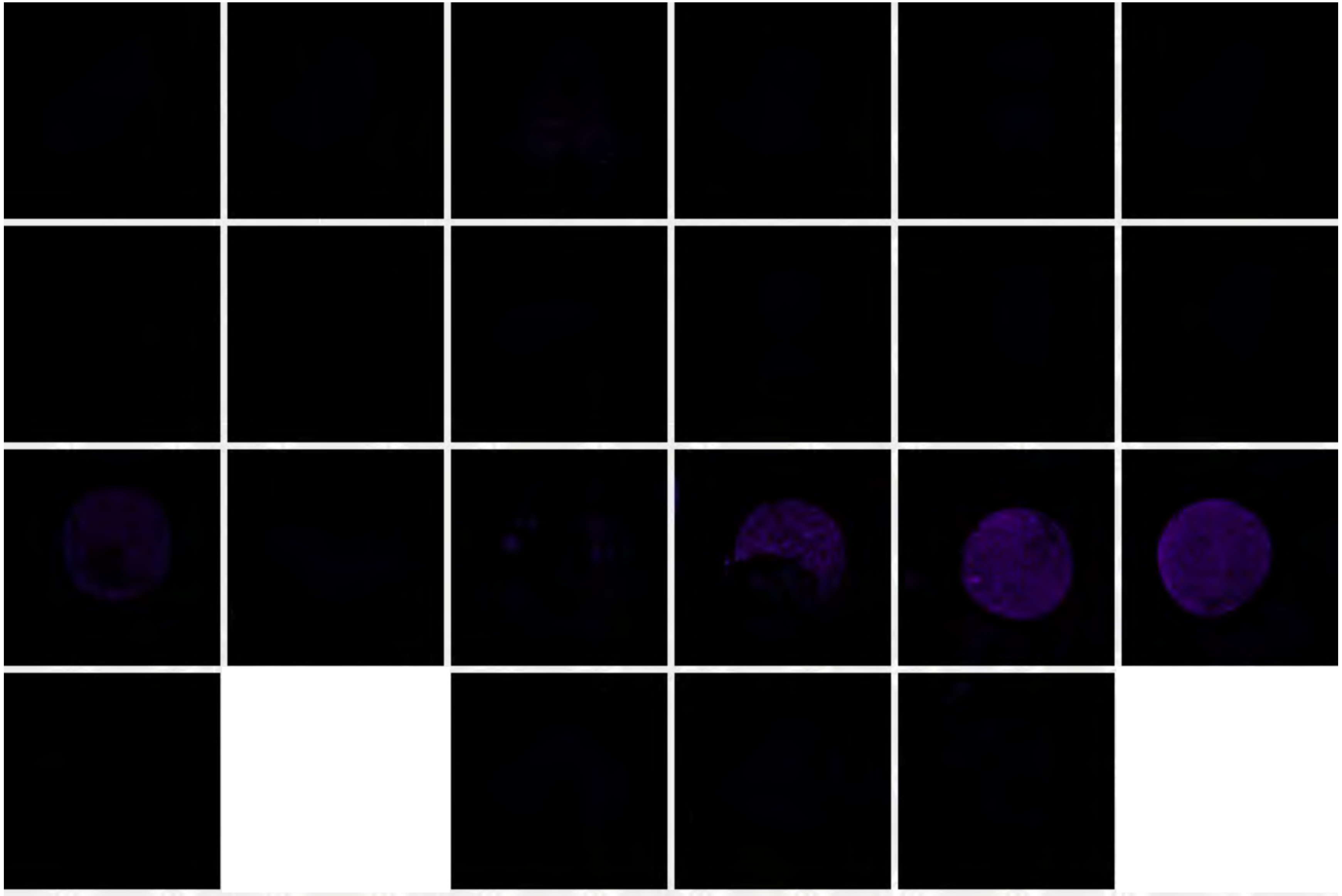

Row 7- Full RADD

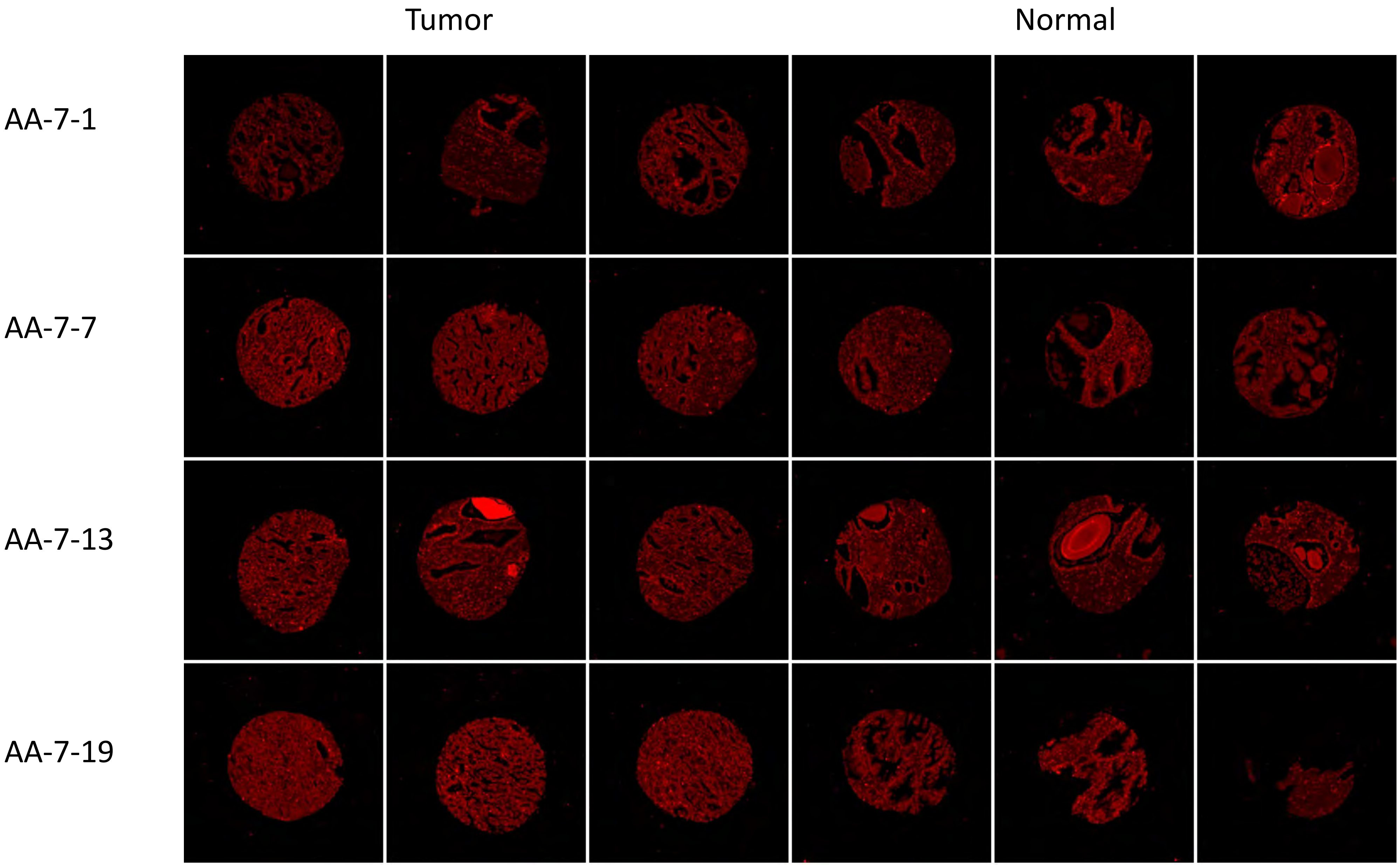

Row 7- oxRADD

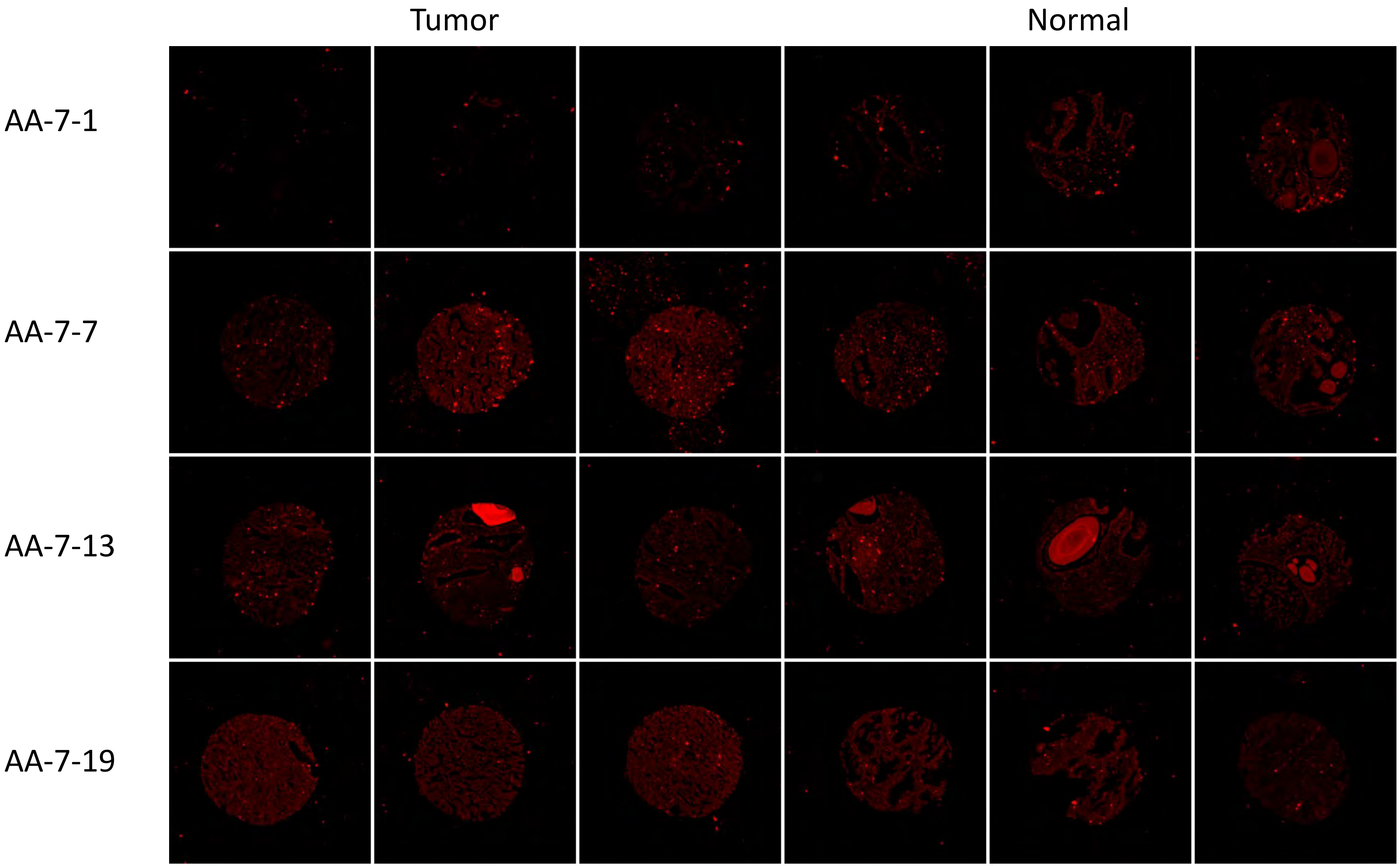

Row 7- UDG

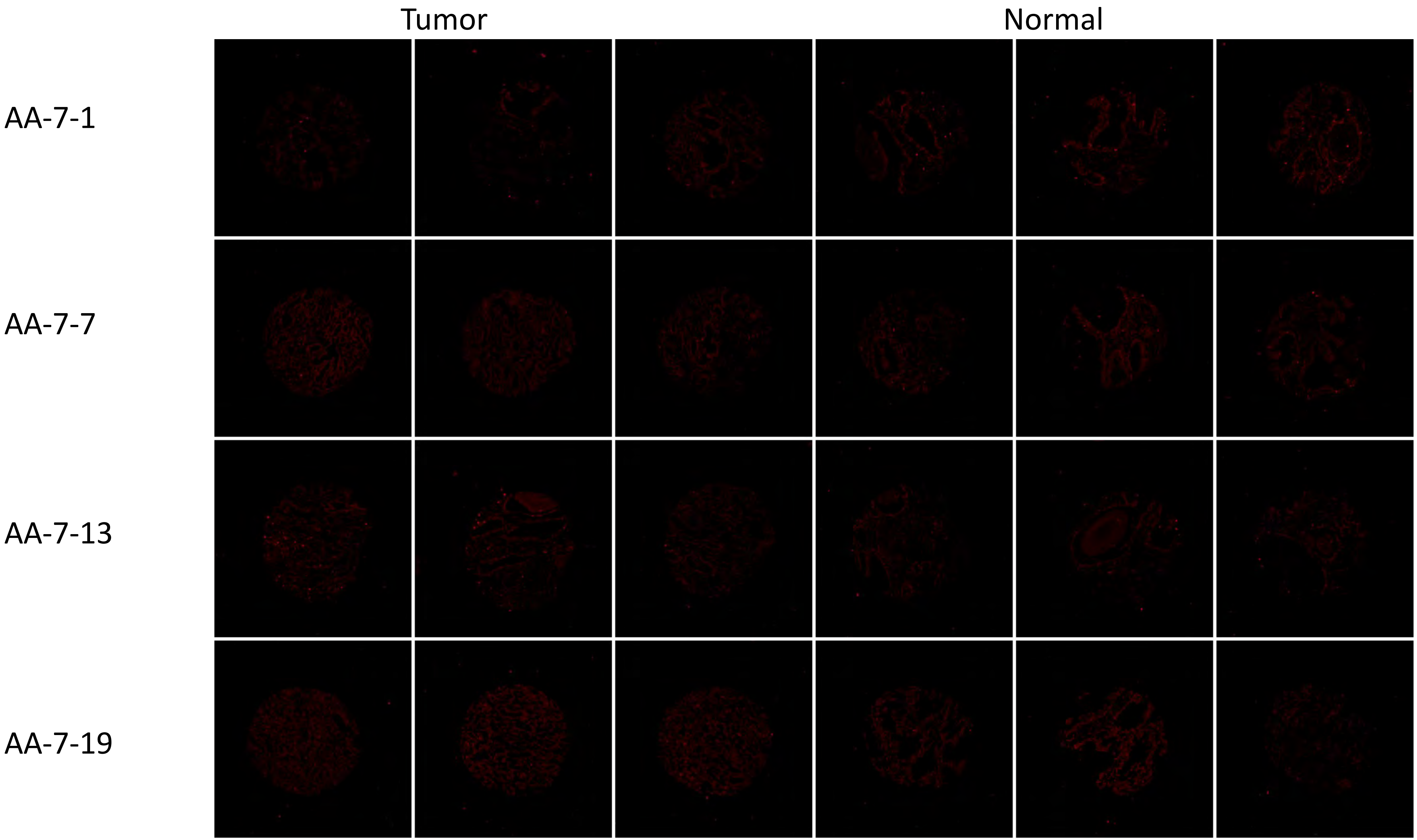

Row 7- T4PDG

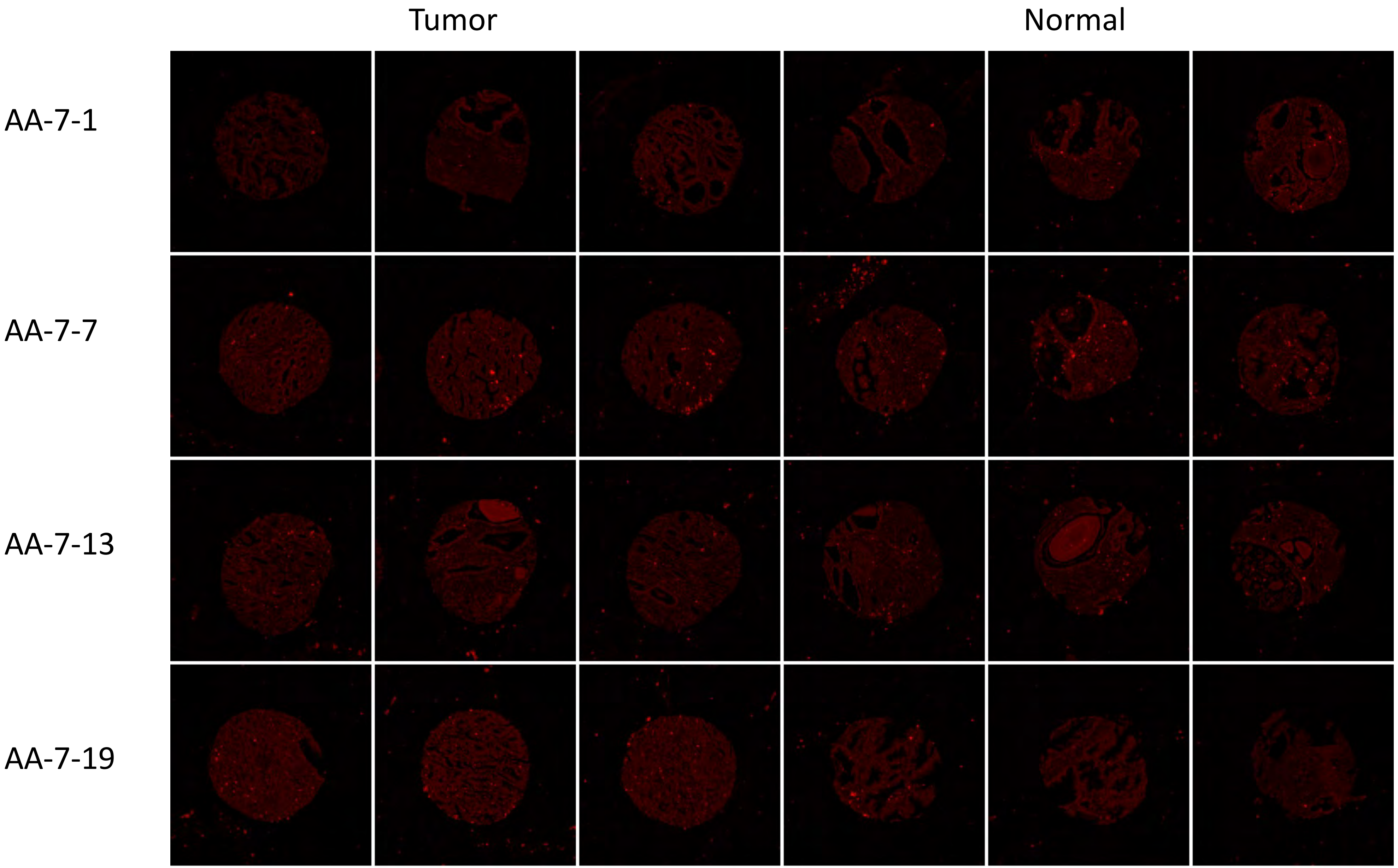

Row 7- XRCC1

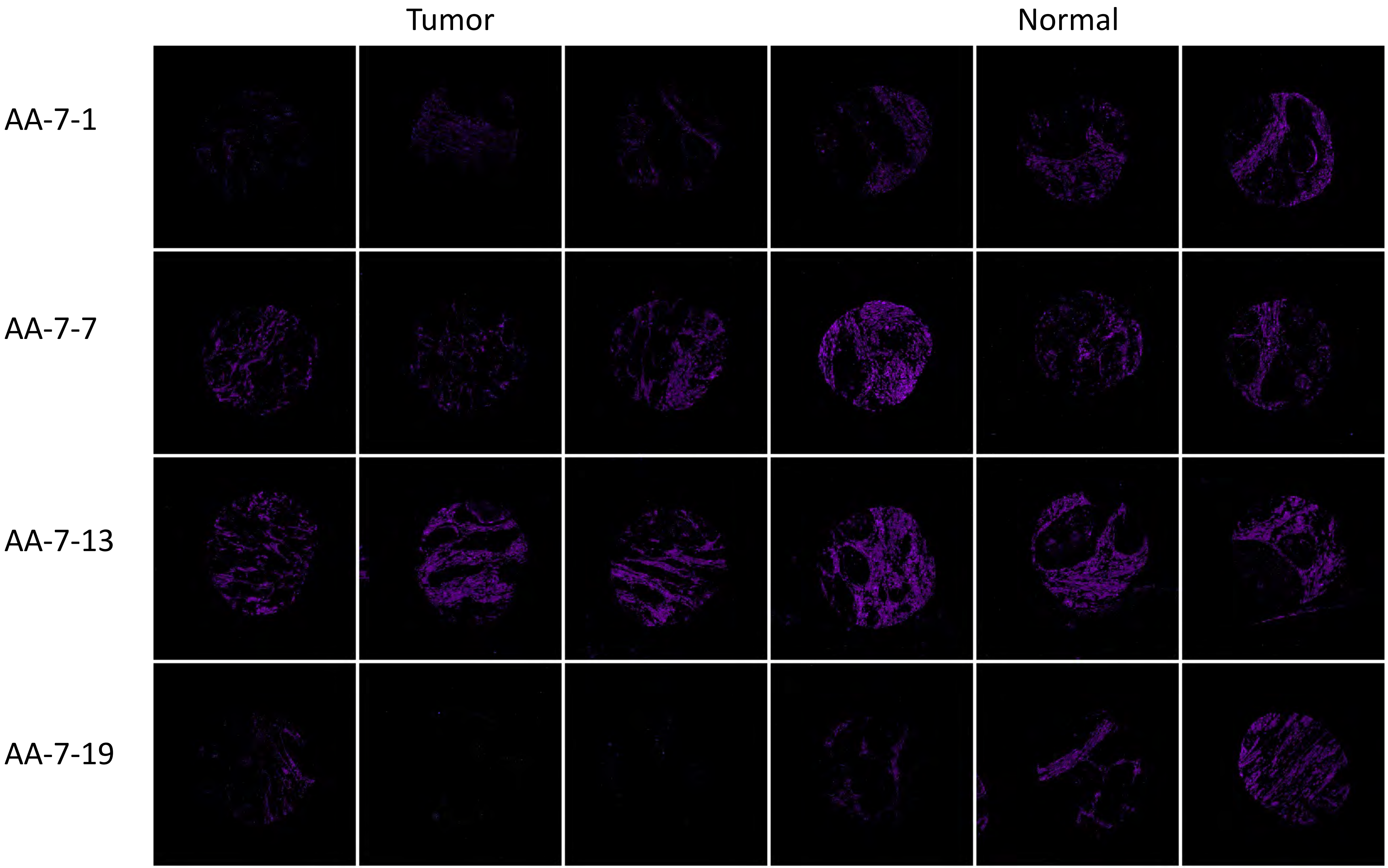

Row 7- PARP1

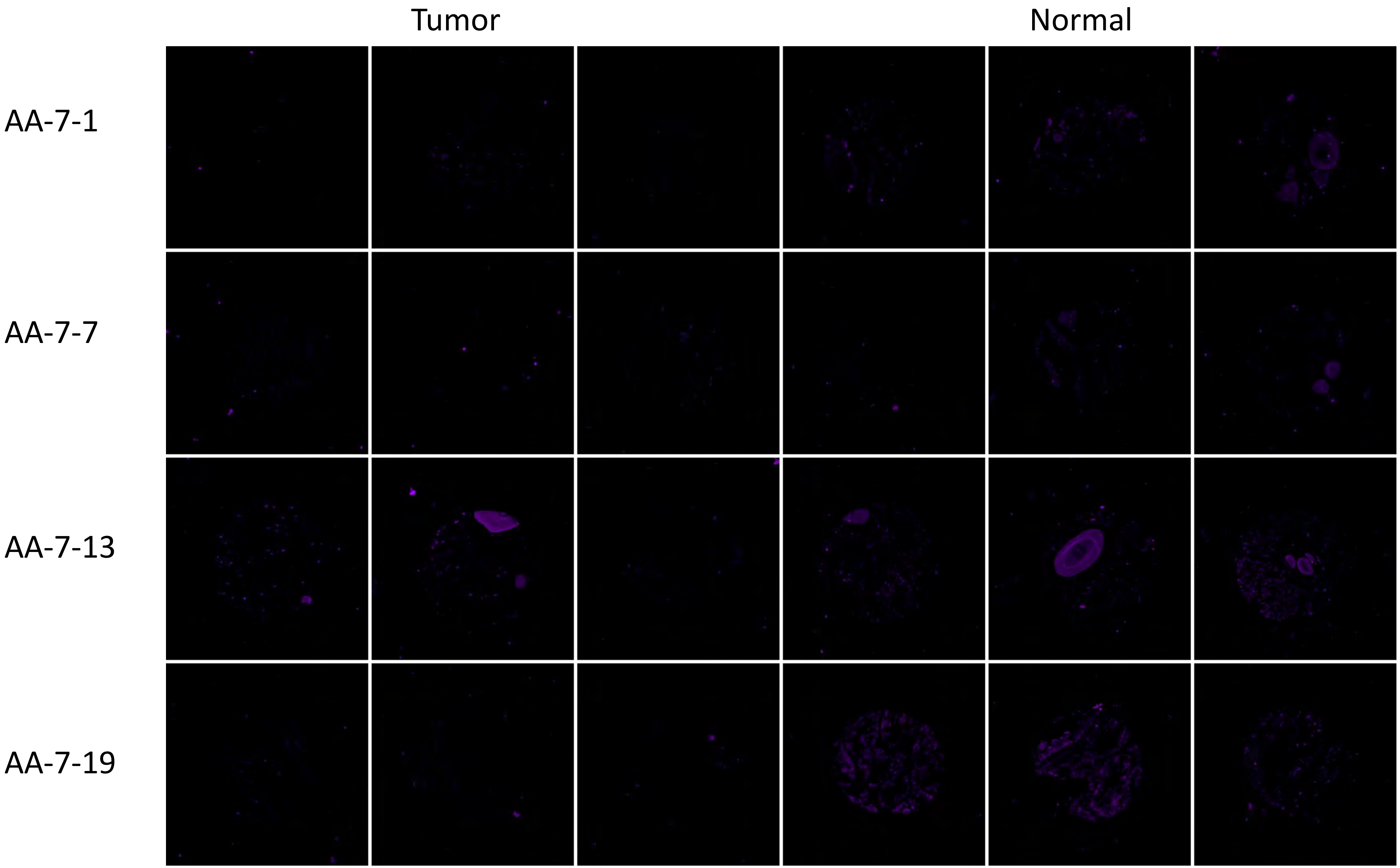

Row 7- UNG

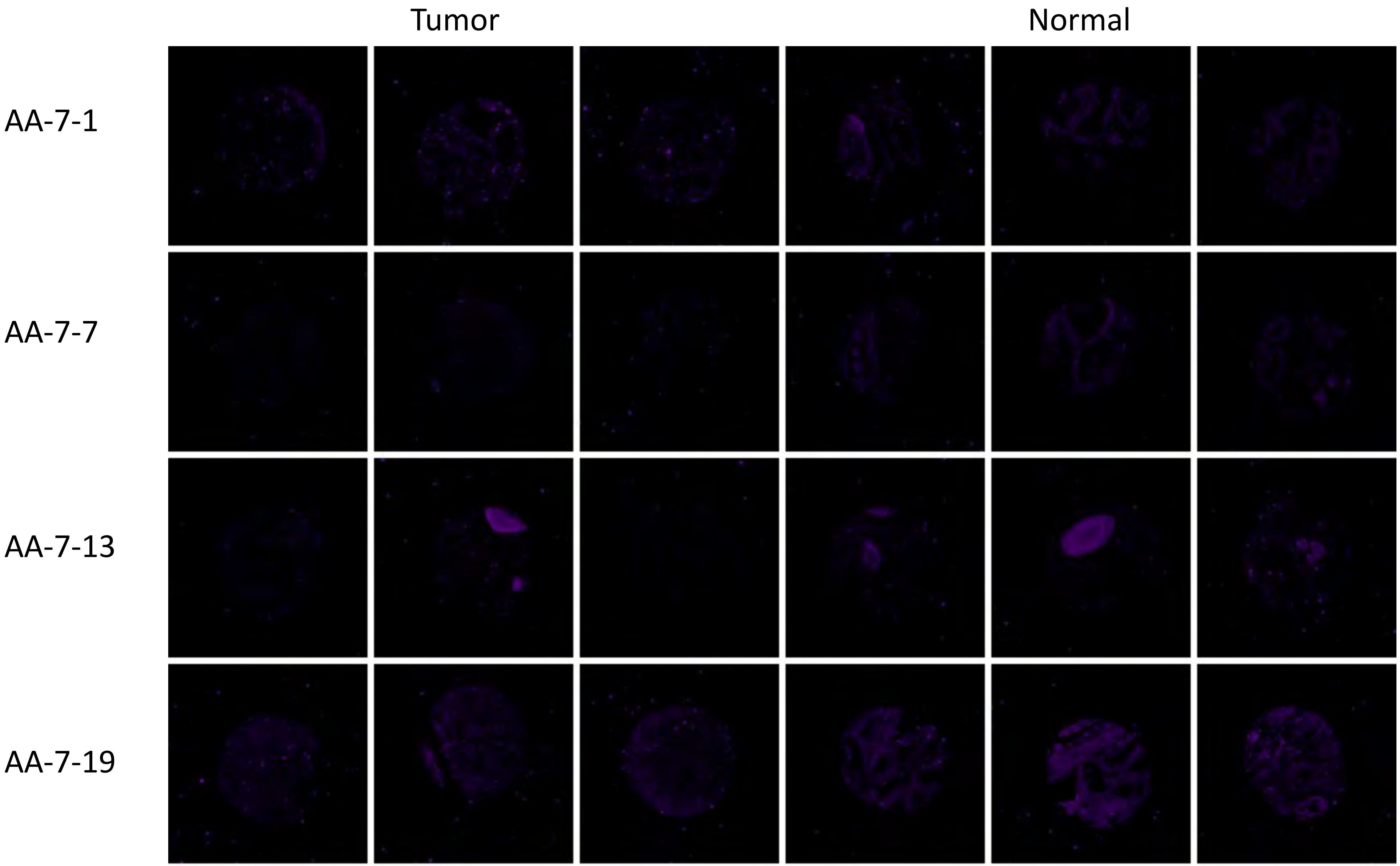

Row 8- Full RADD

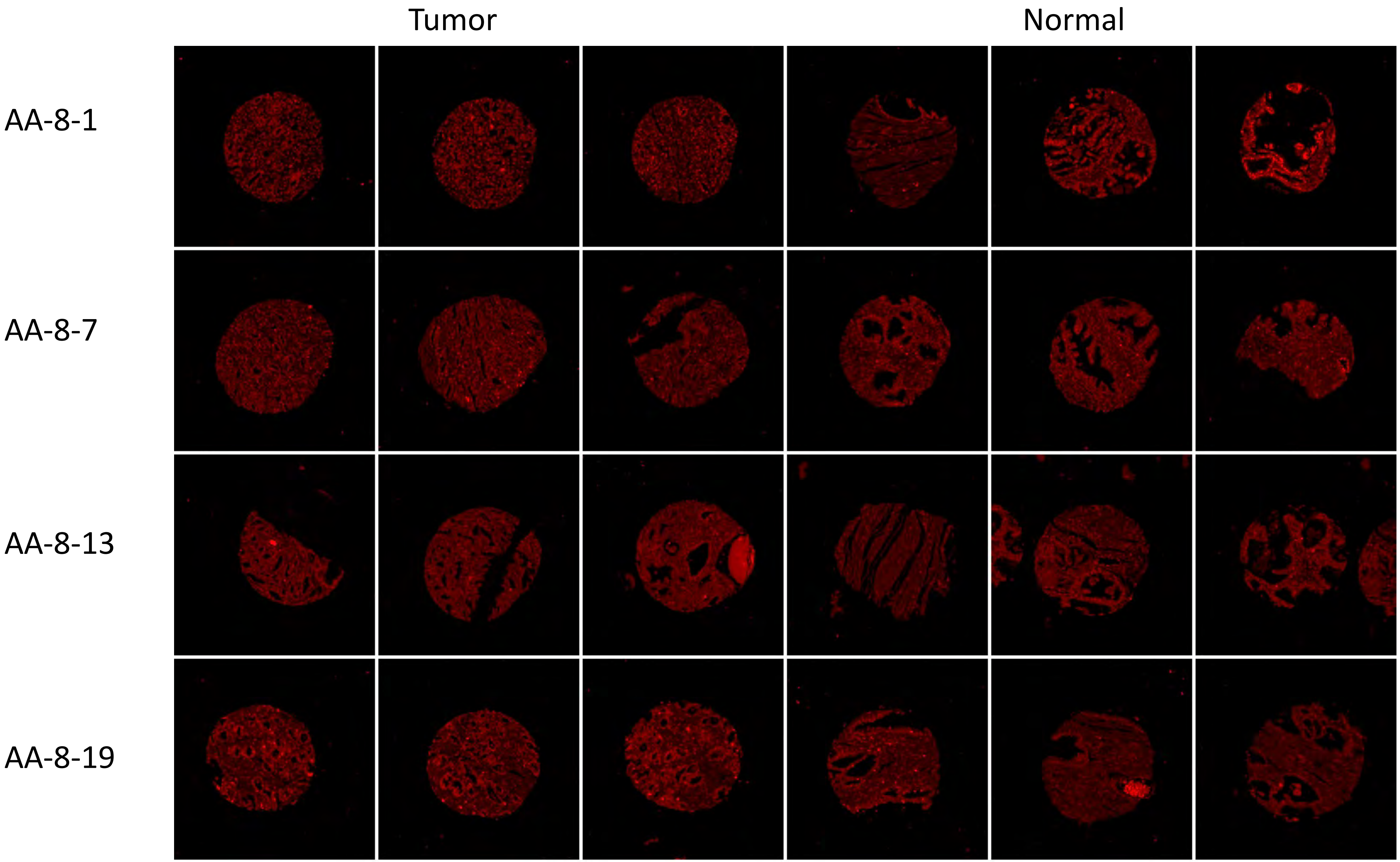

Row 8- oxRADD

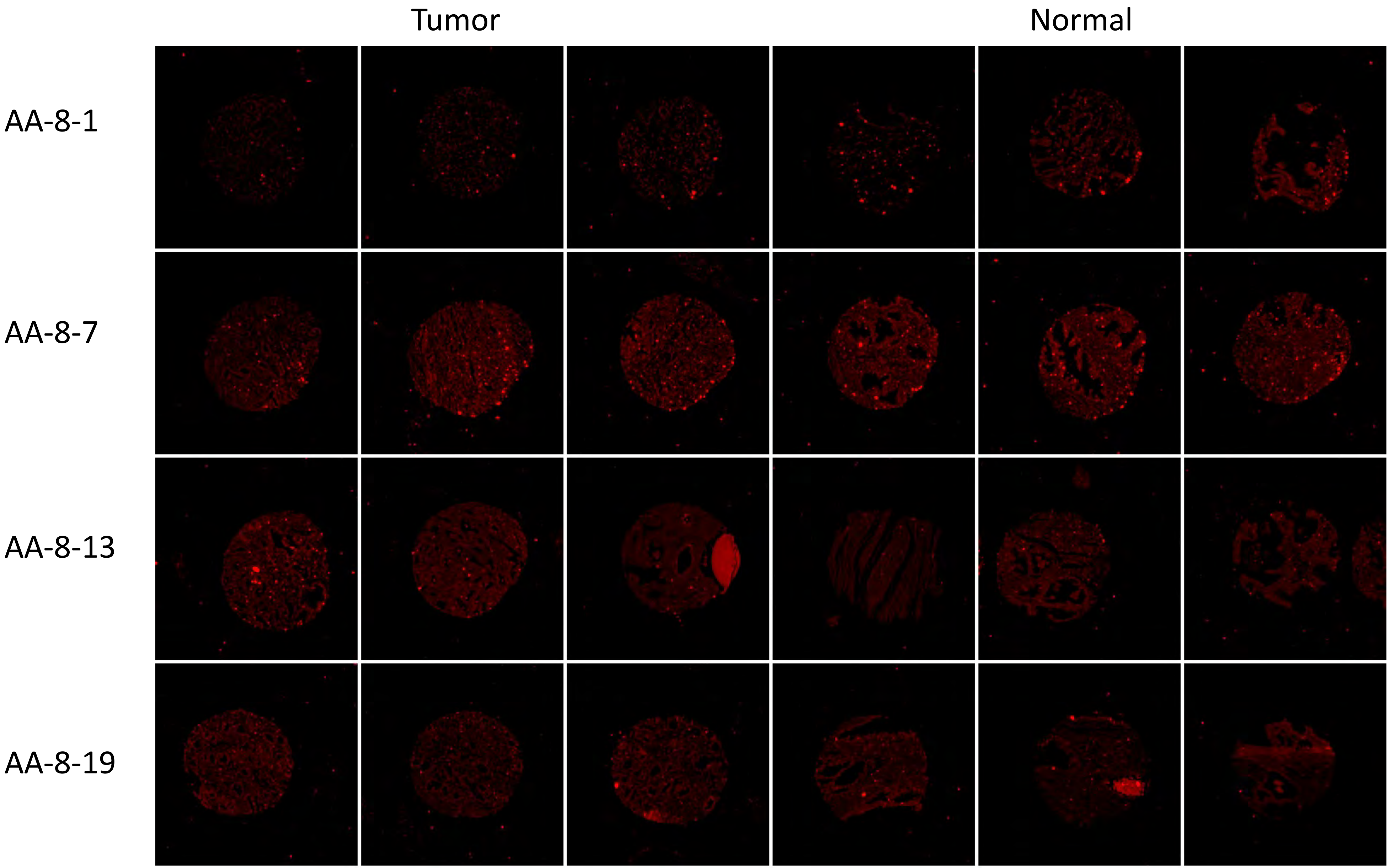

Row 8- UDG

Tumor | Normal

AA-8-1

AA-8-7

AA-8-13

AA-8-19

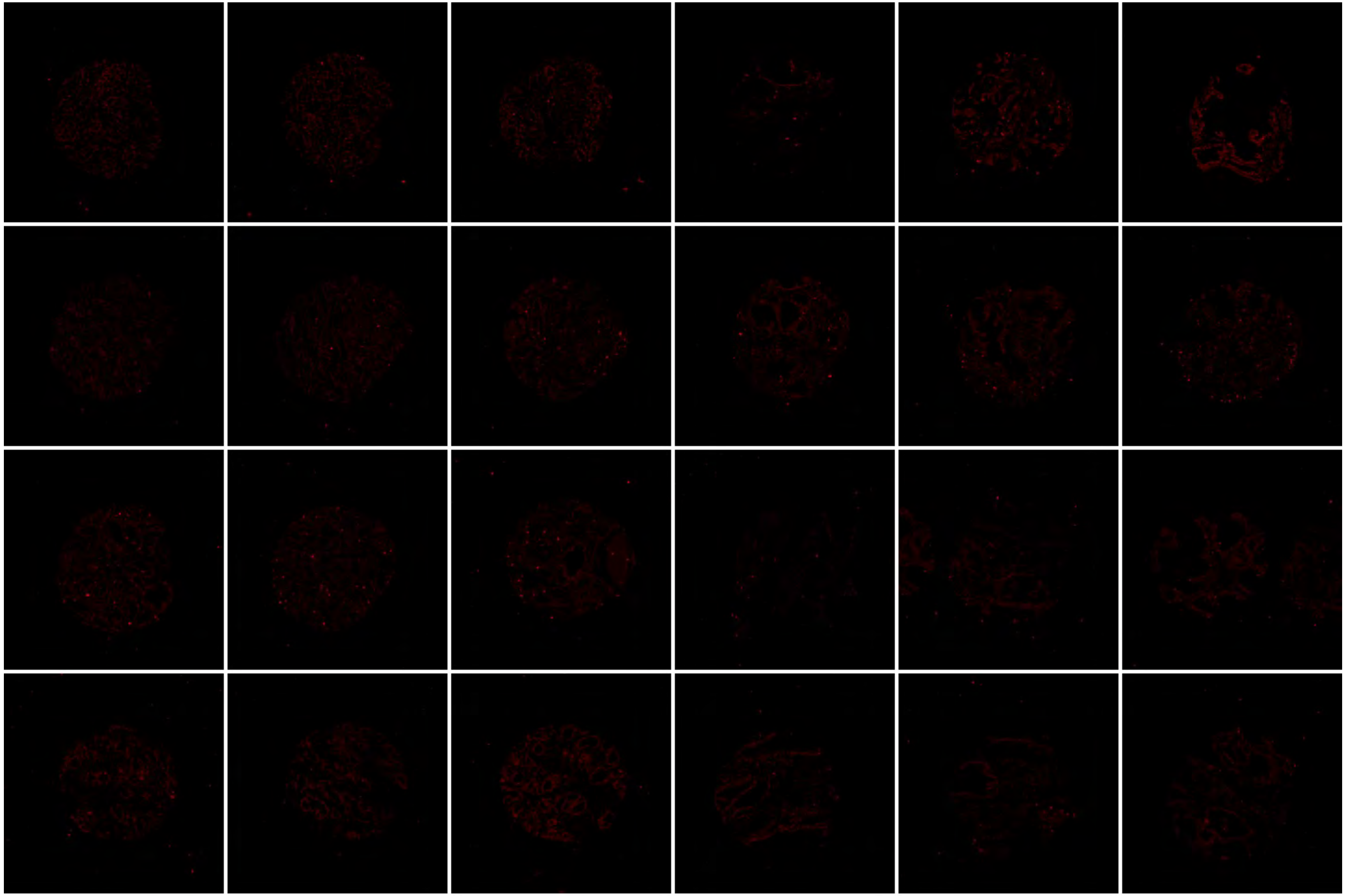

Row 8- T4PDG

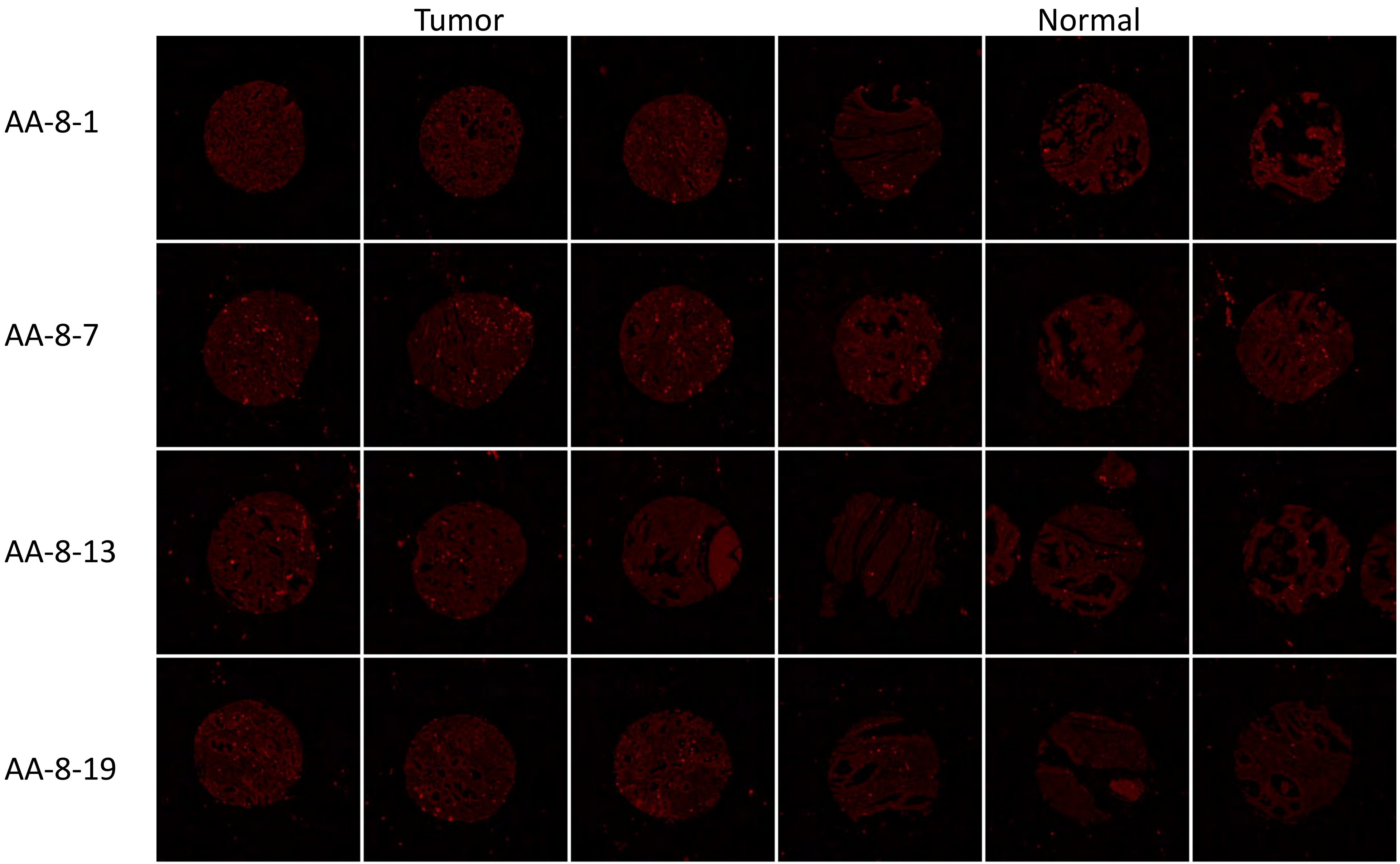

Row 8- XRCC1

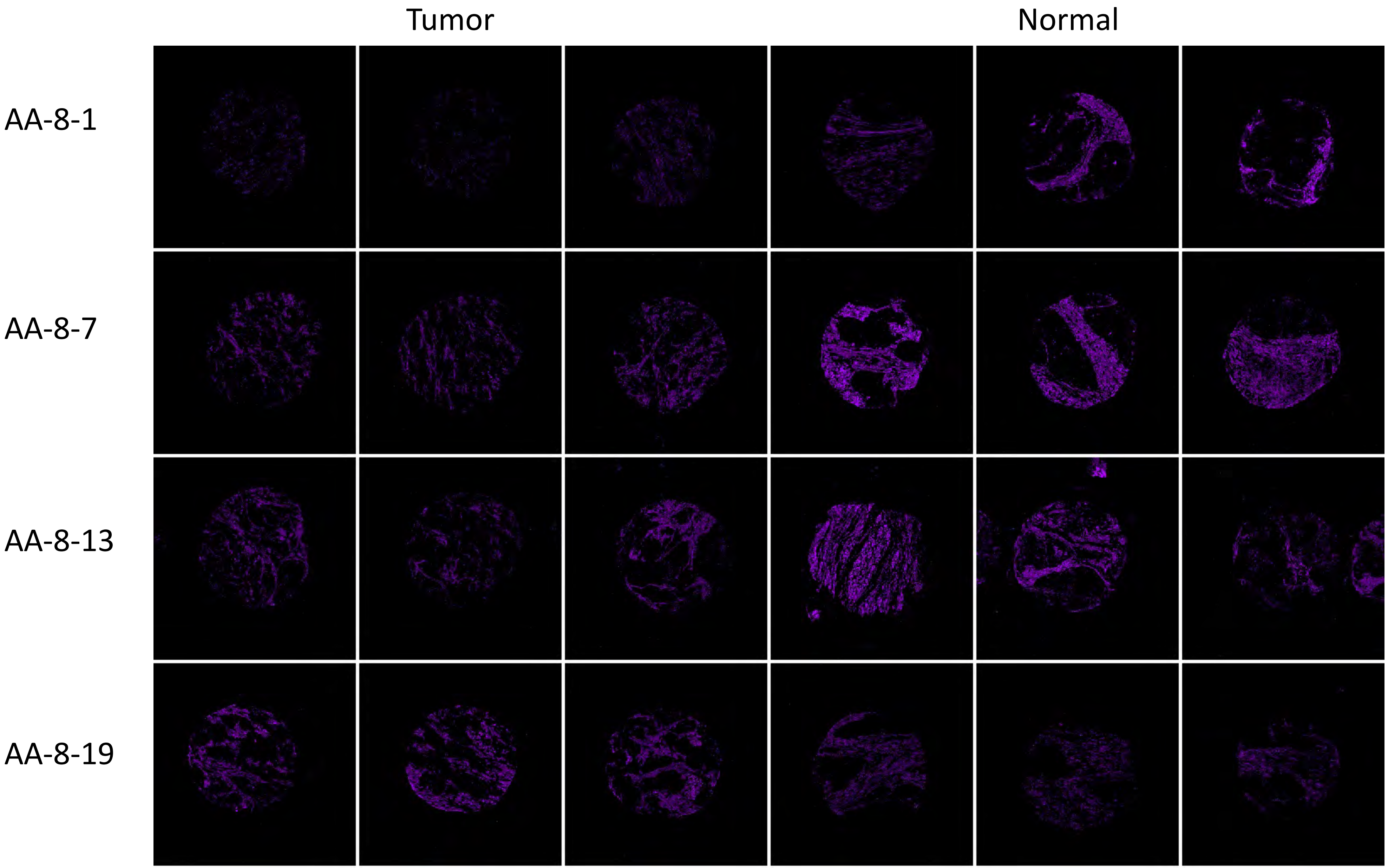

Row 8- PARP1

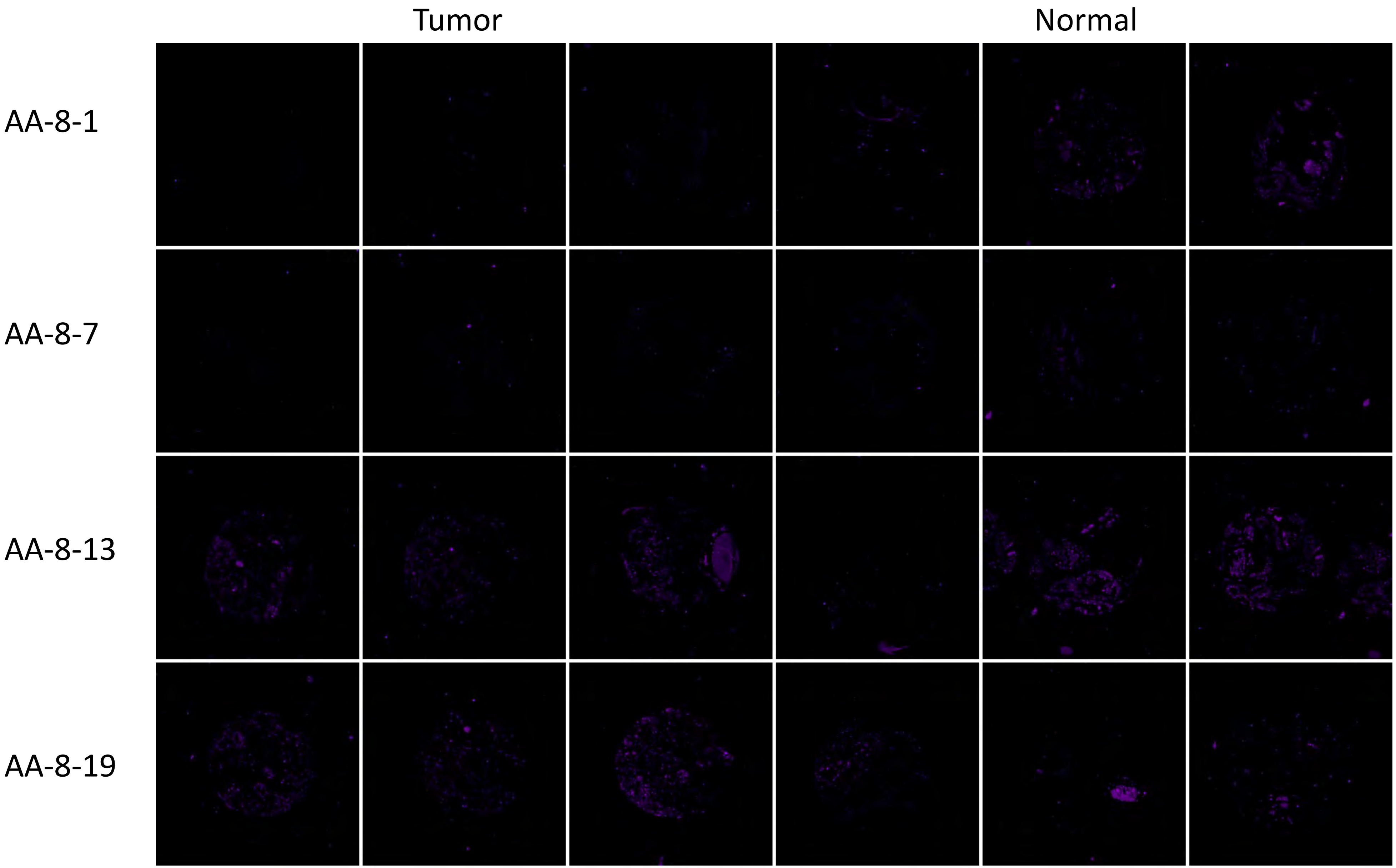

Row 8- UNG

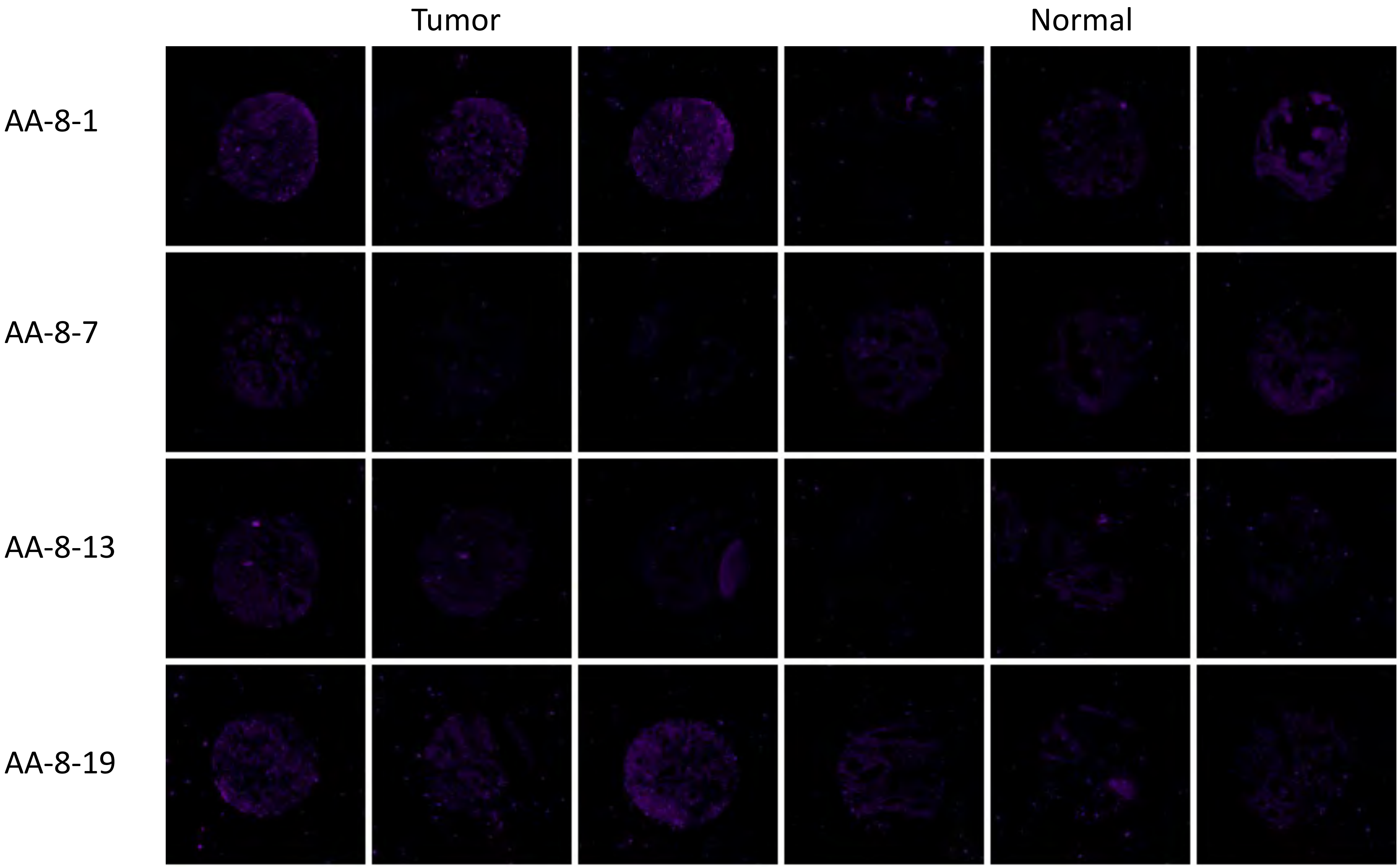

Row 9- Full RADD

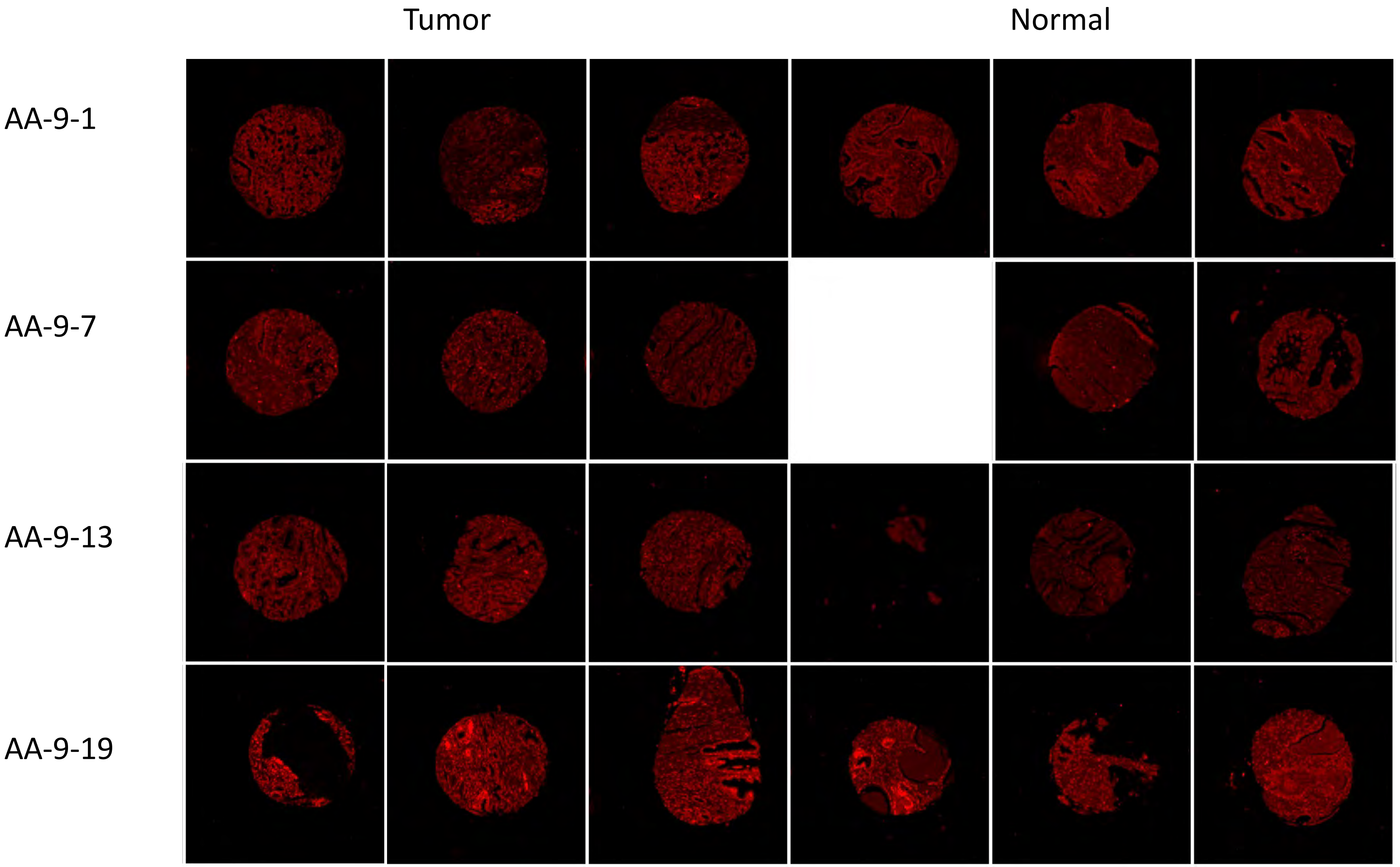

Row 9- oxRADD

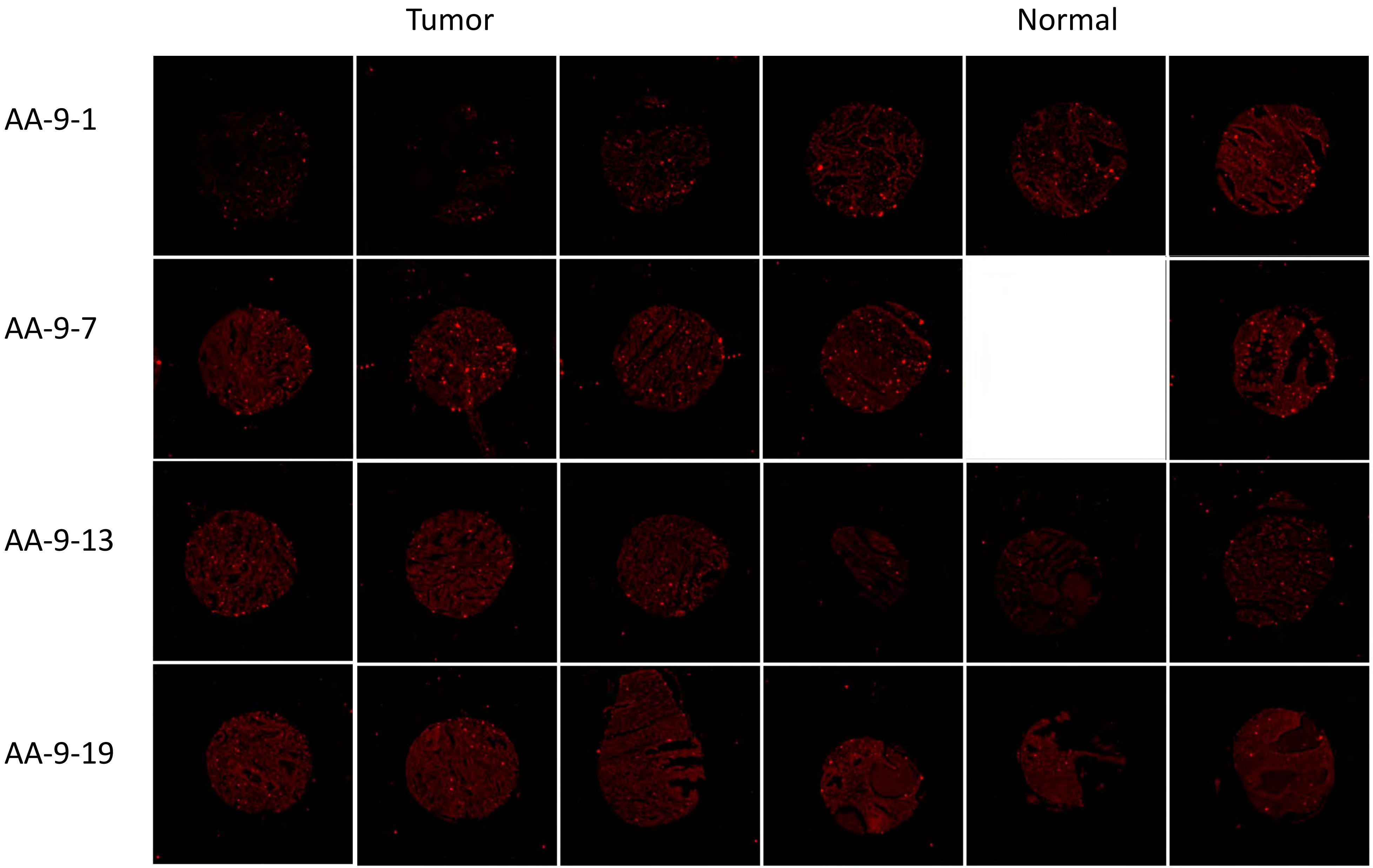

Row 9- UDG

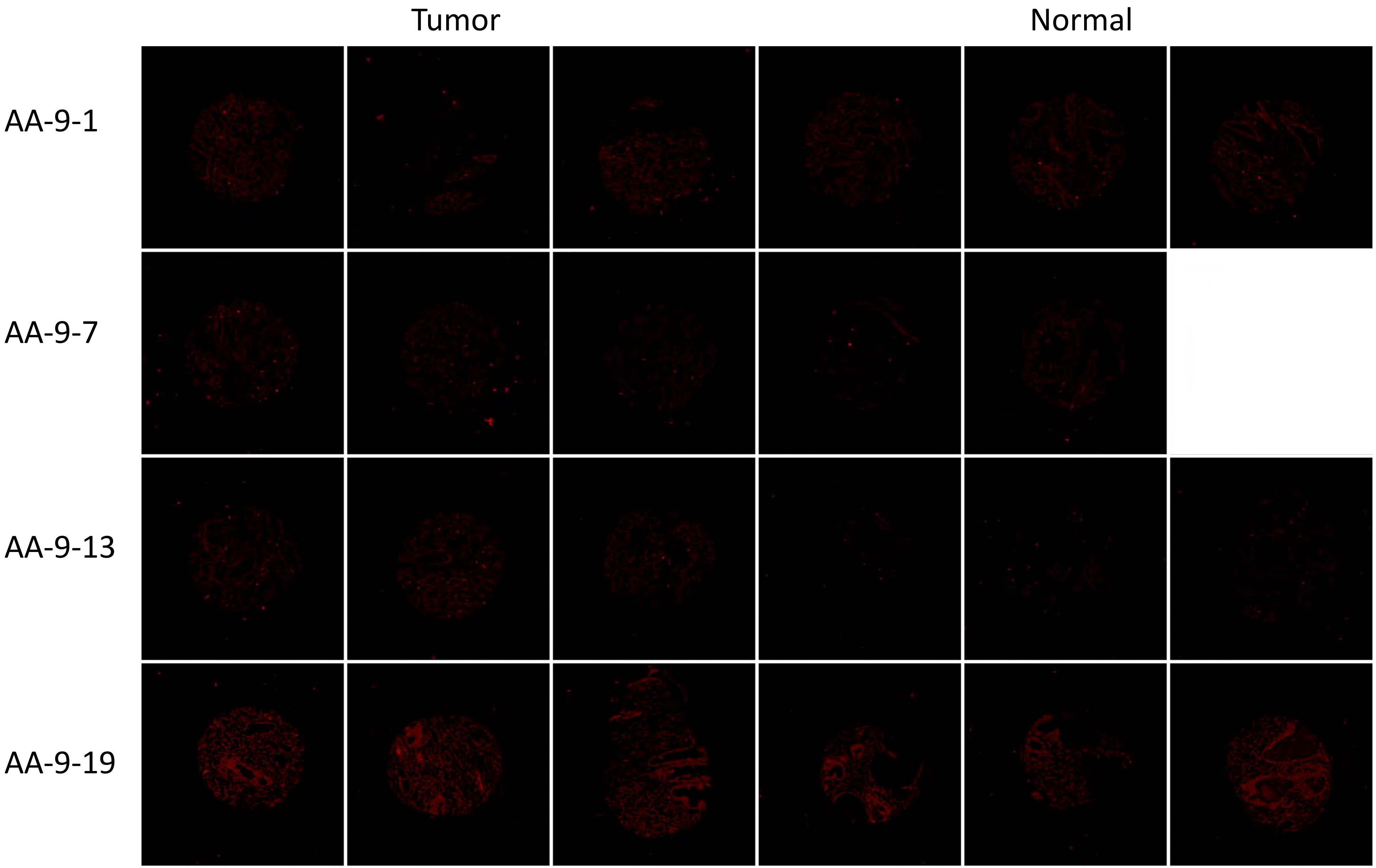

Row 9- T4PDG

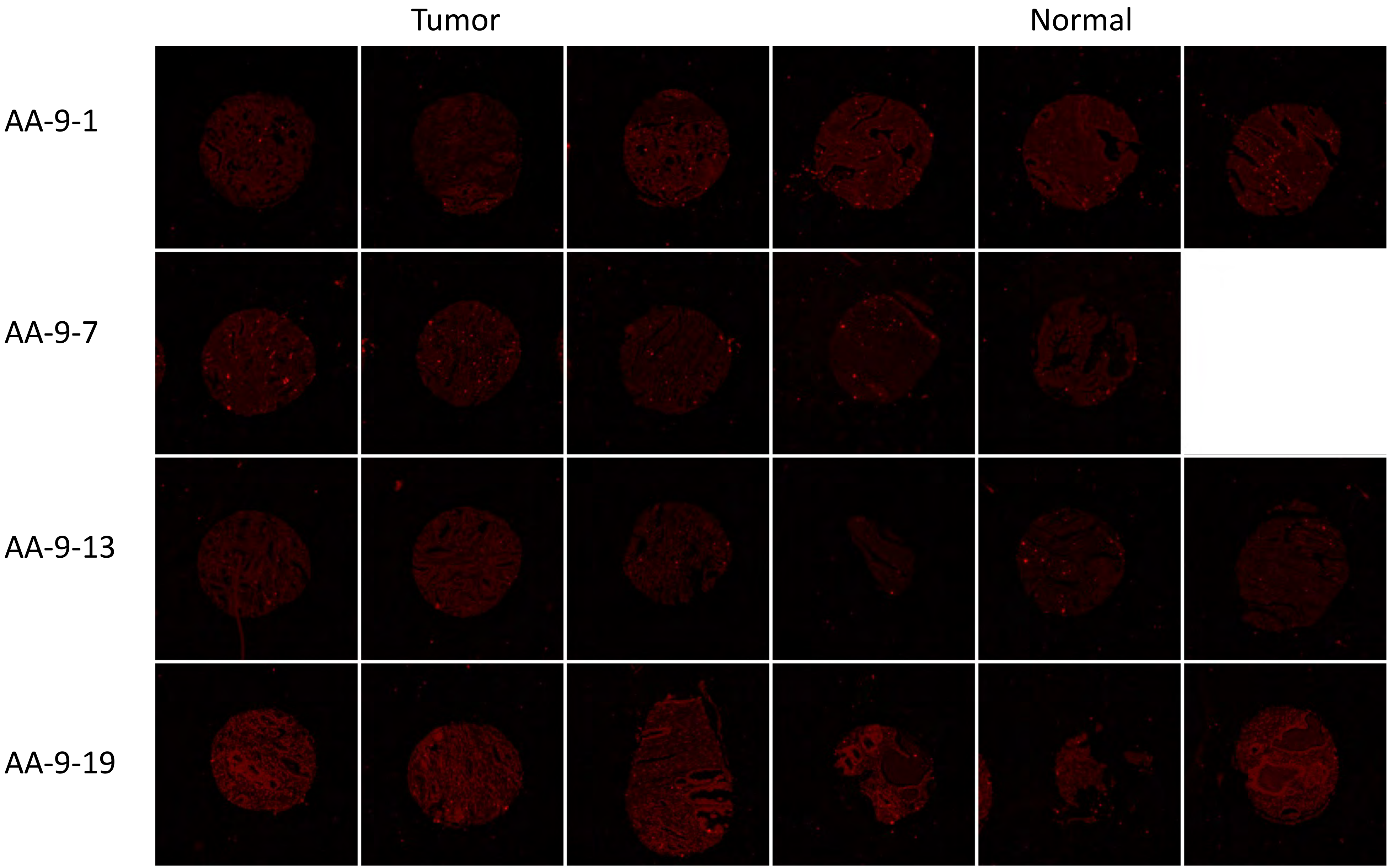

Row 9- XRCC1

Tumor | Normal

AA-9-1

AA-9-7

AA-9-13

AA-9-19

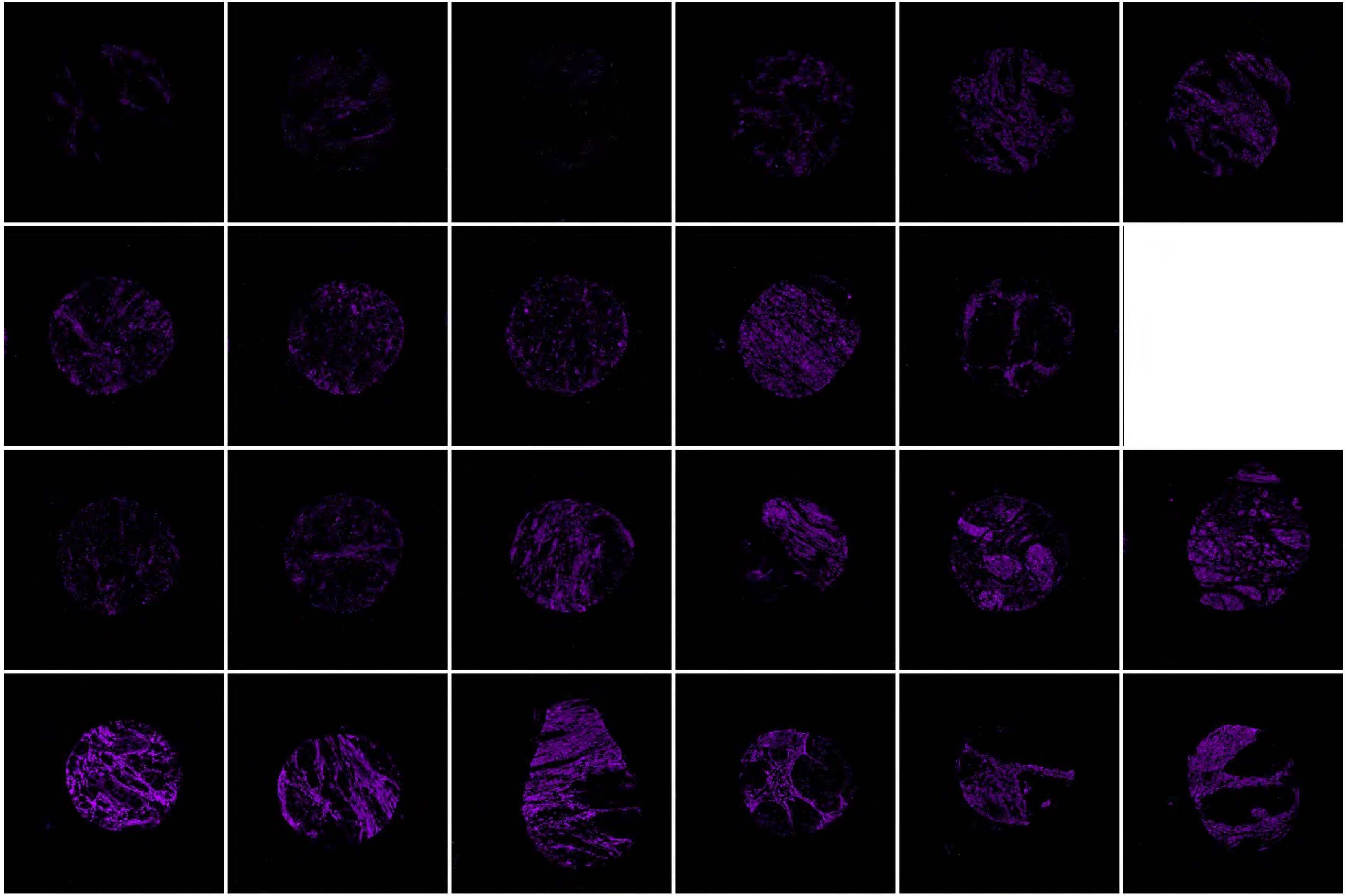

Row 9- PARP1

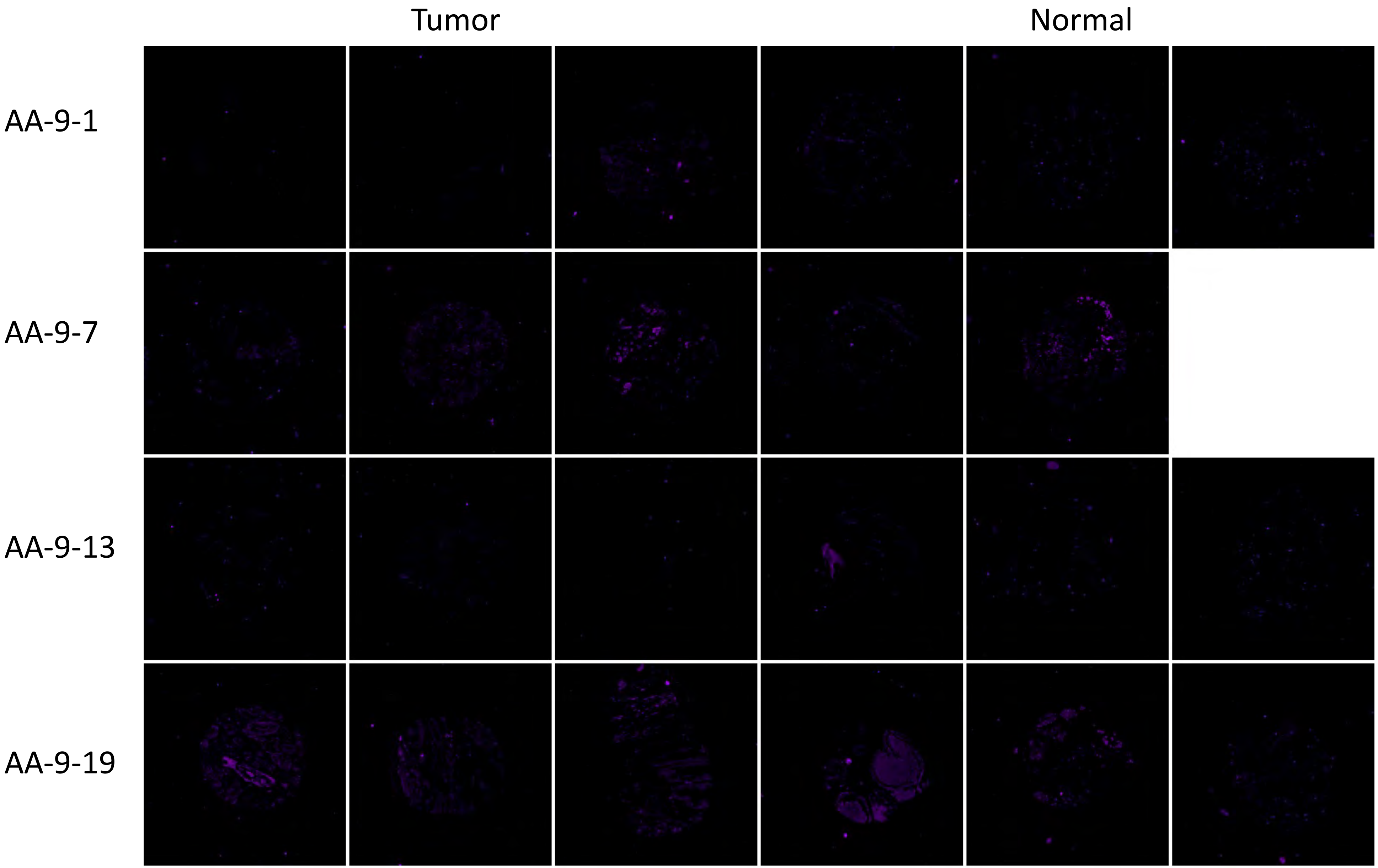

Row 9- UNG

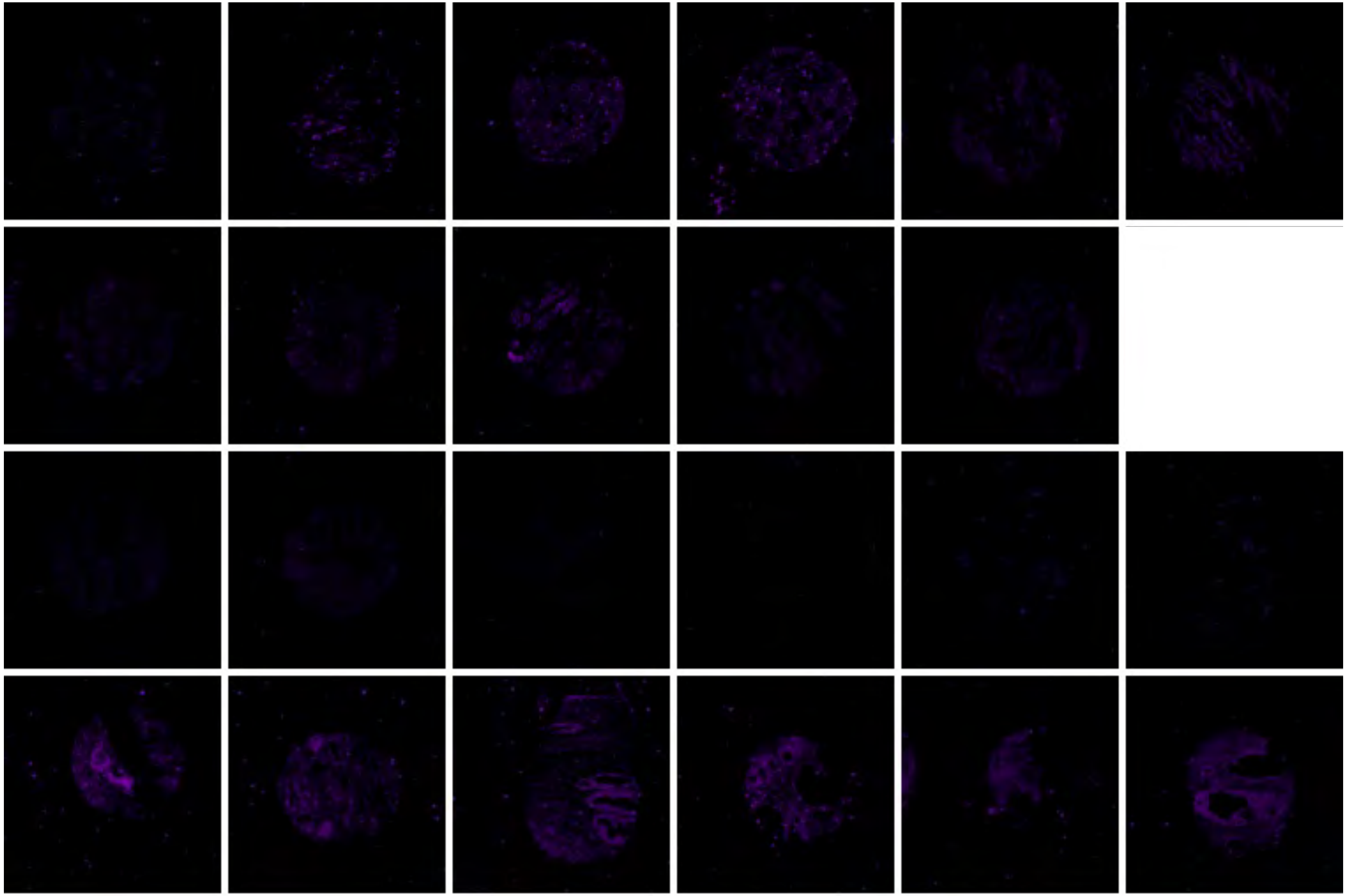

Row 10- Full RADD

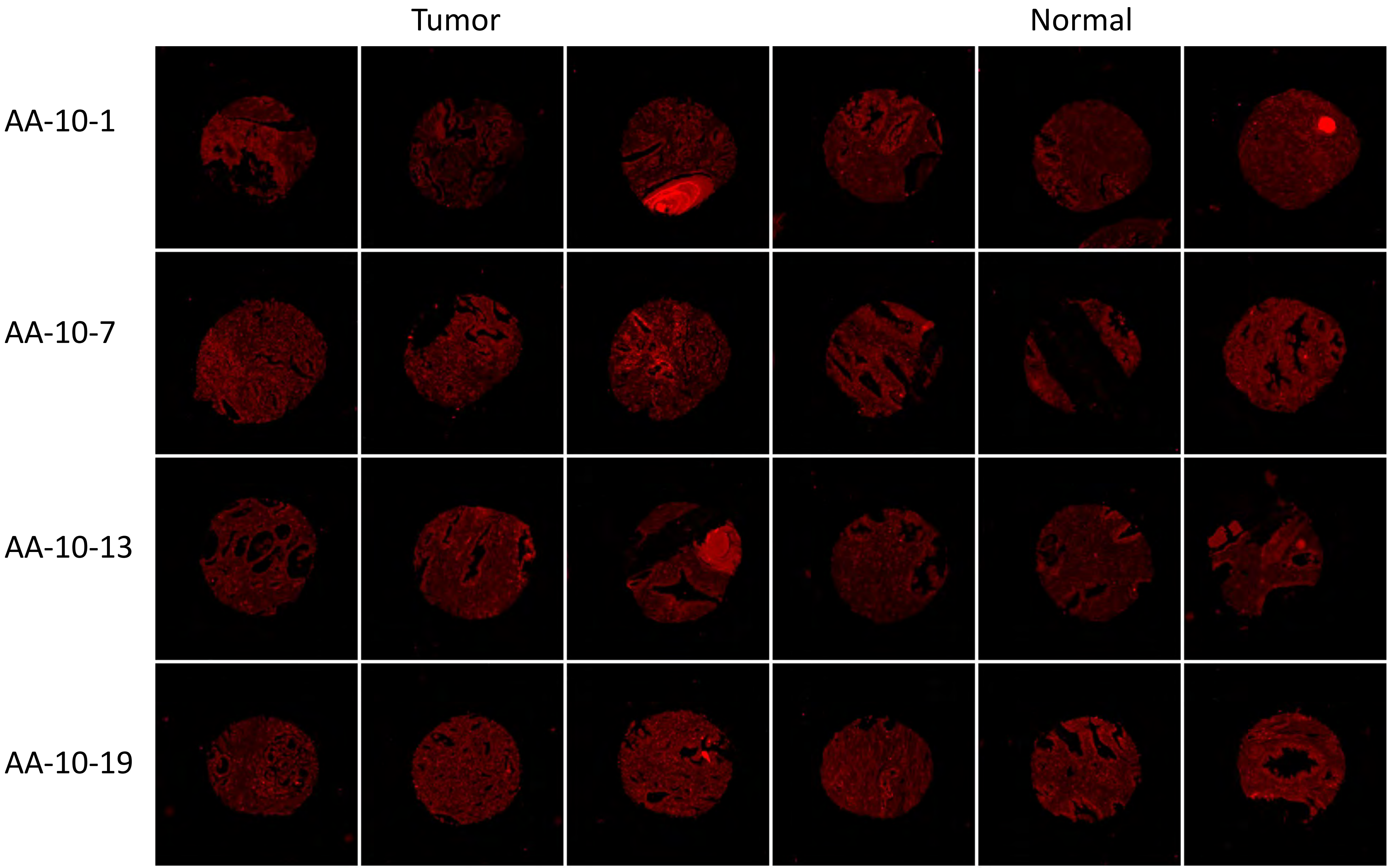

Row 10- oxRADD

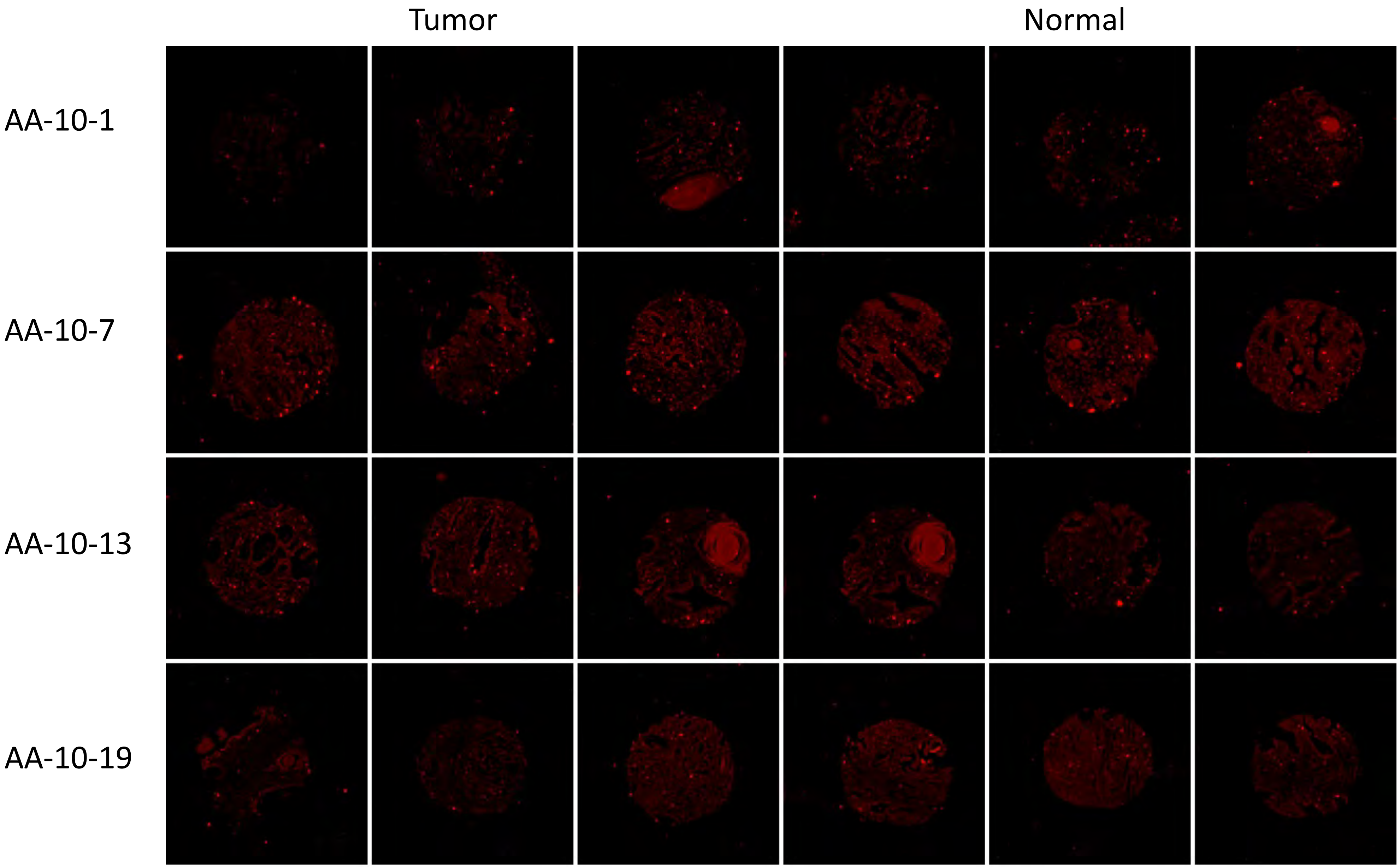

Row 10- UDG

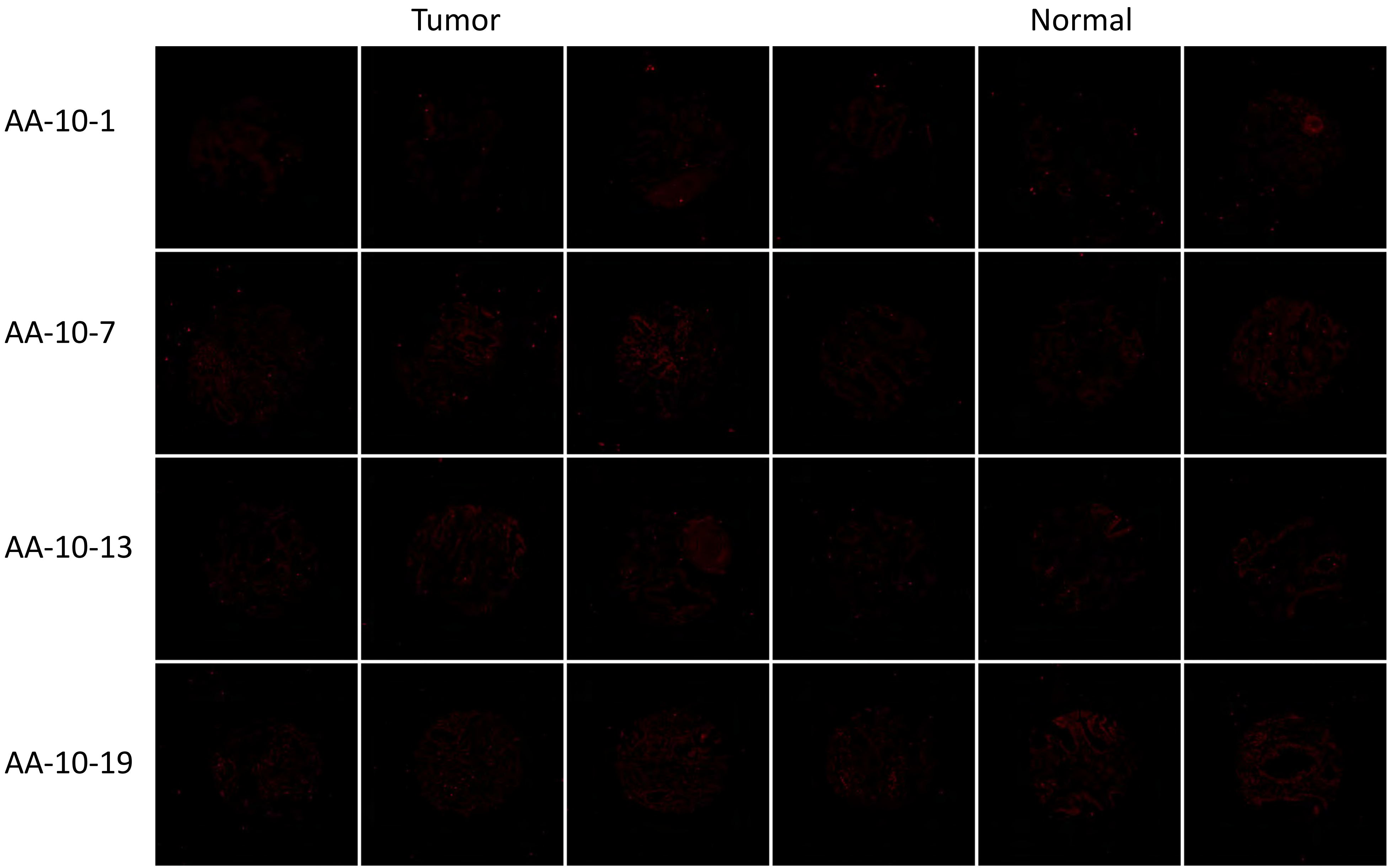

Row 10- T4PDG

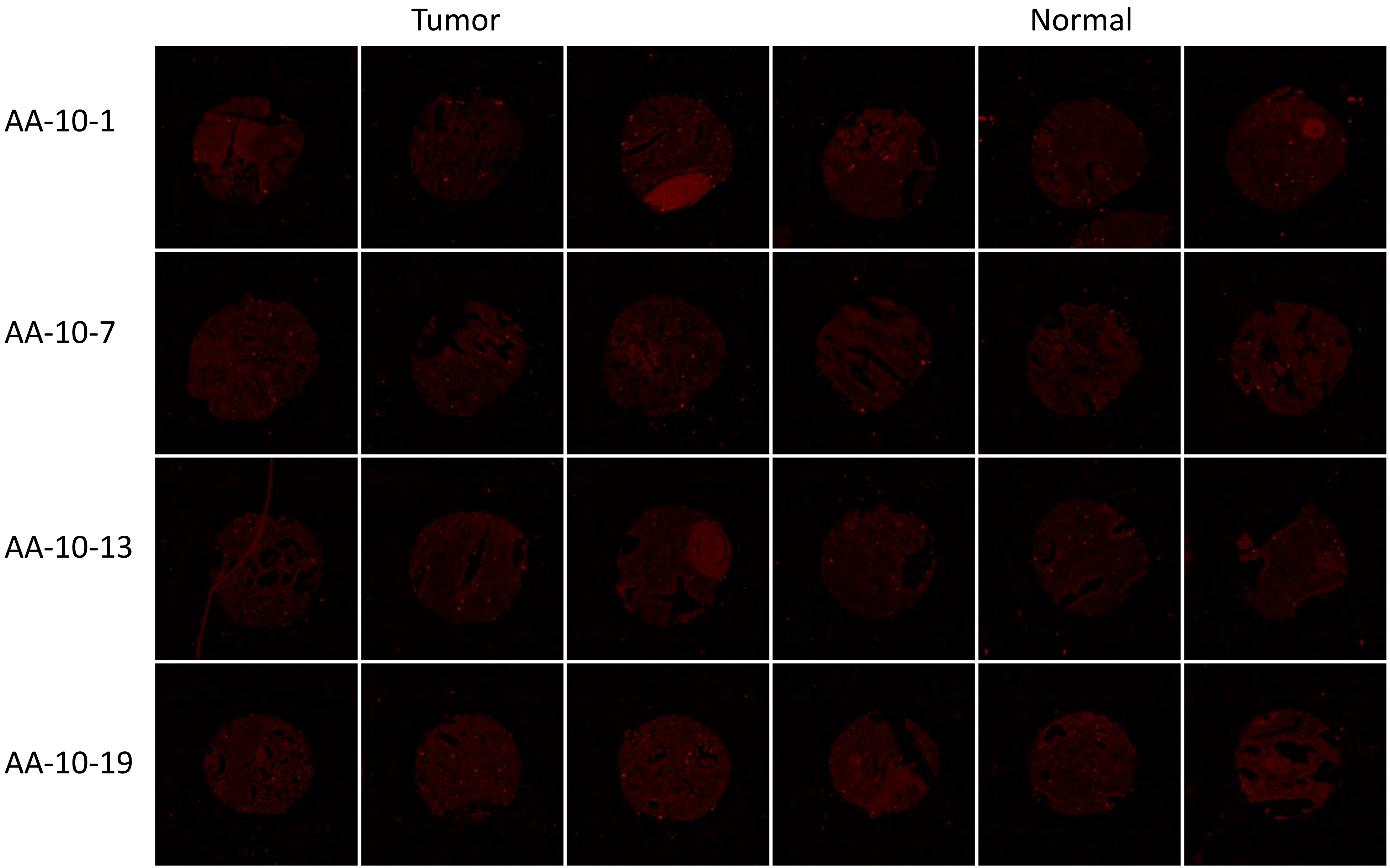

Row 10- XRCC1

Tumor Normal

AA-10-1

AA-10-7

AA-10-13

AA-10-19

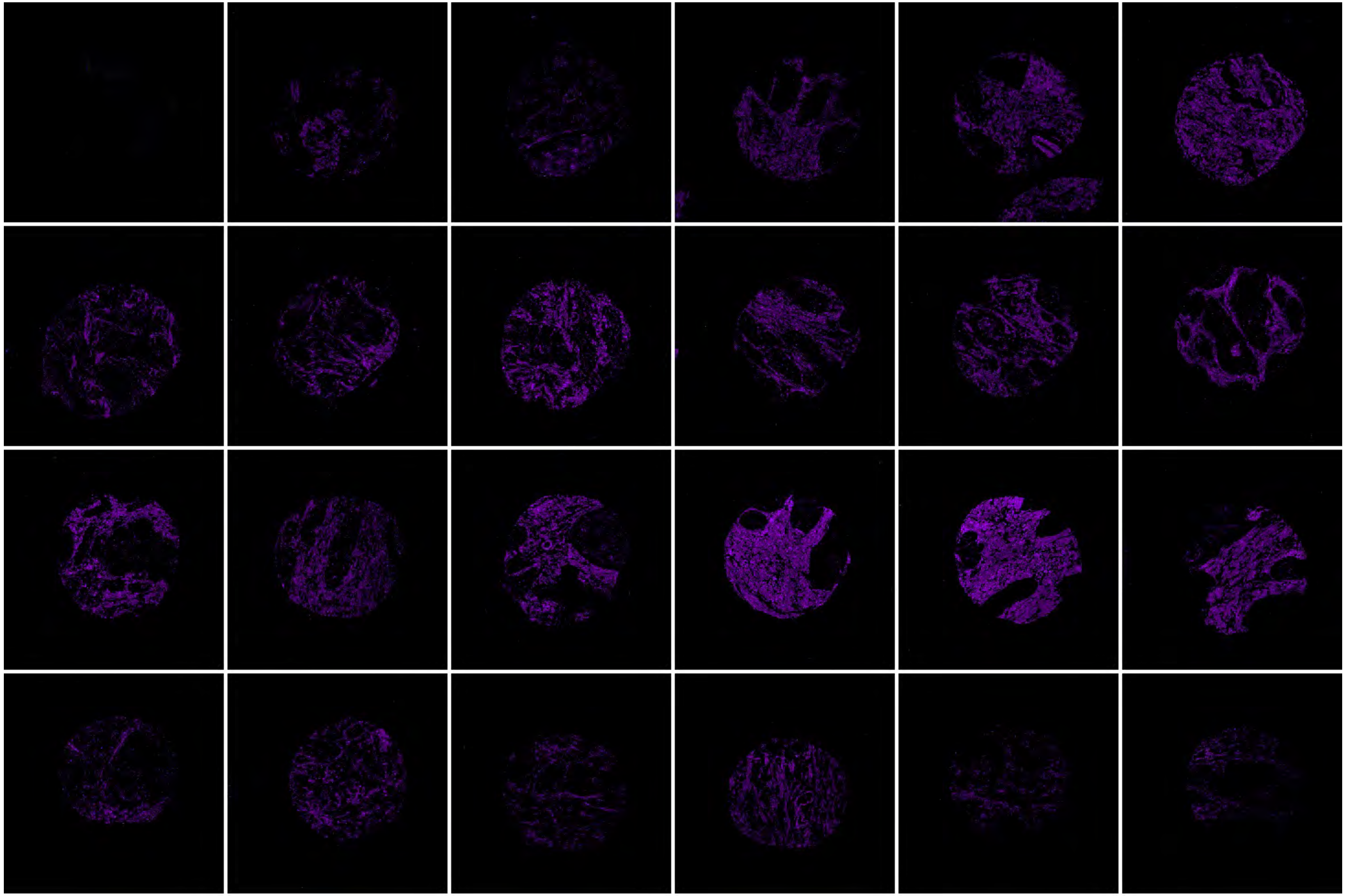

Row 10- PARP1

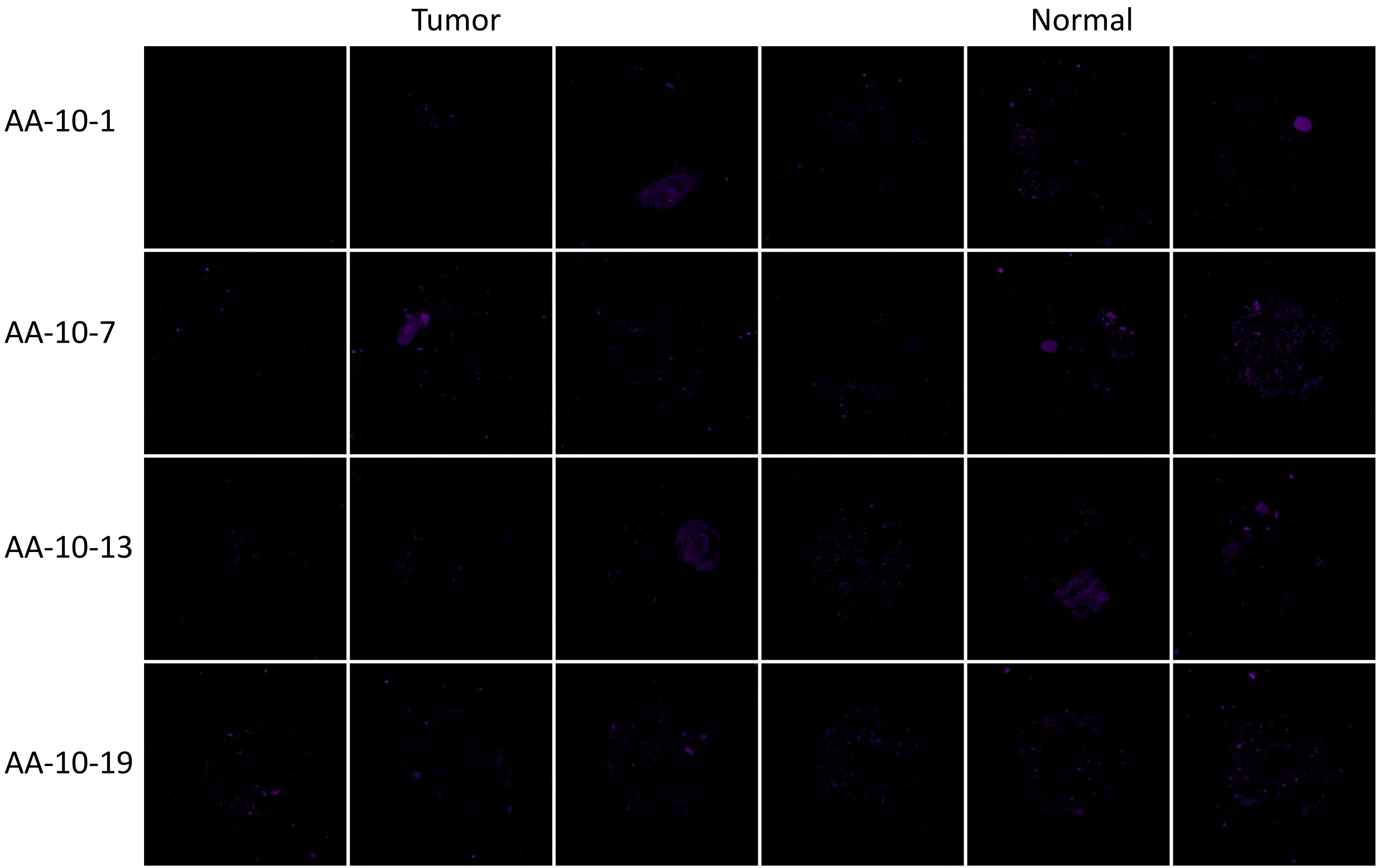

Row 10- UNG

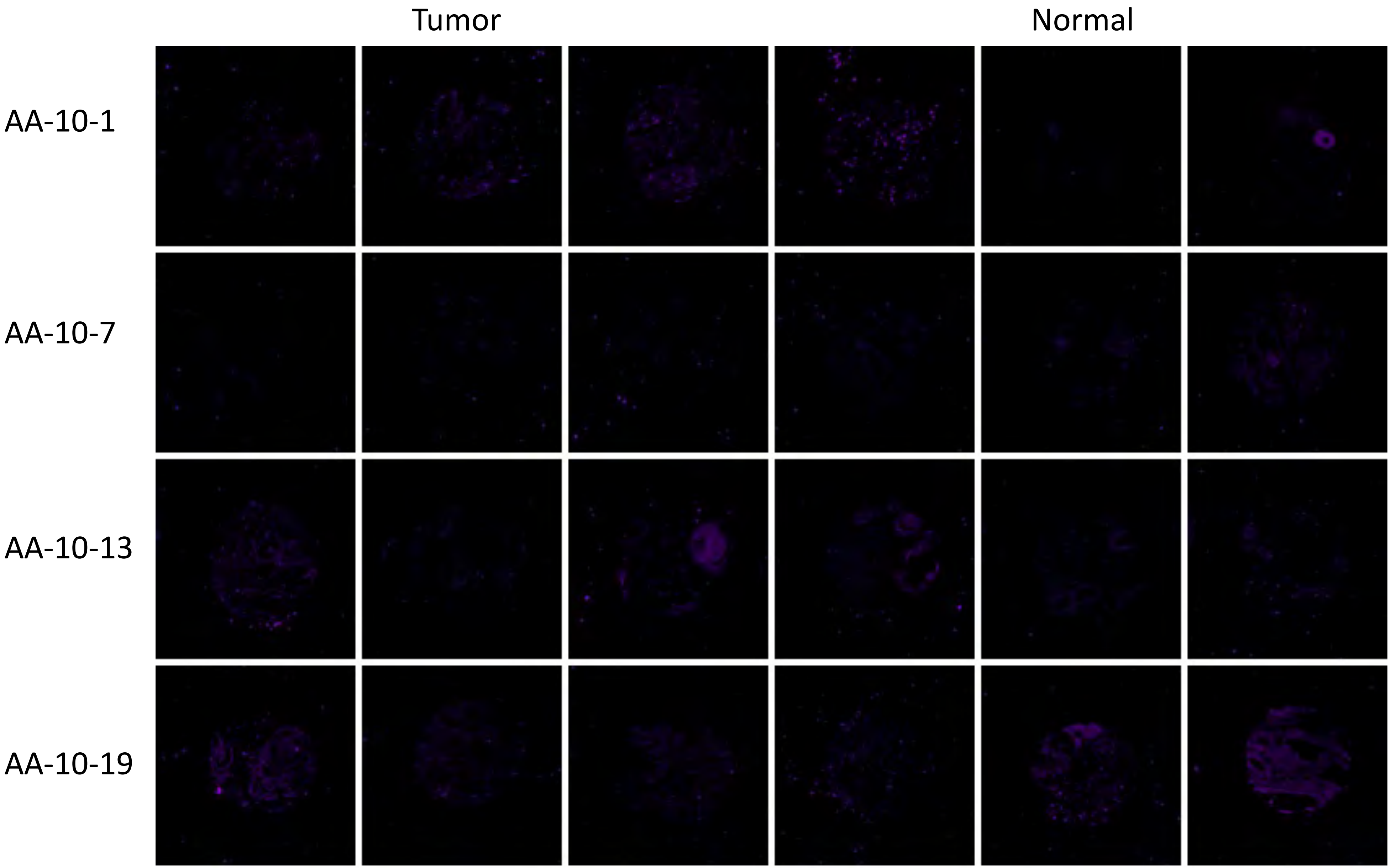

Row 11- Full RADD

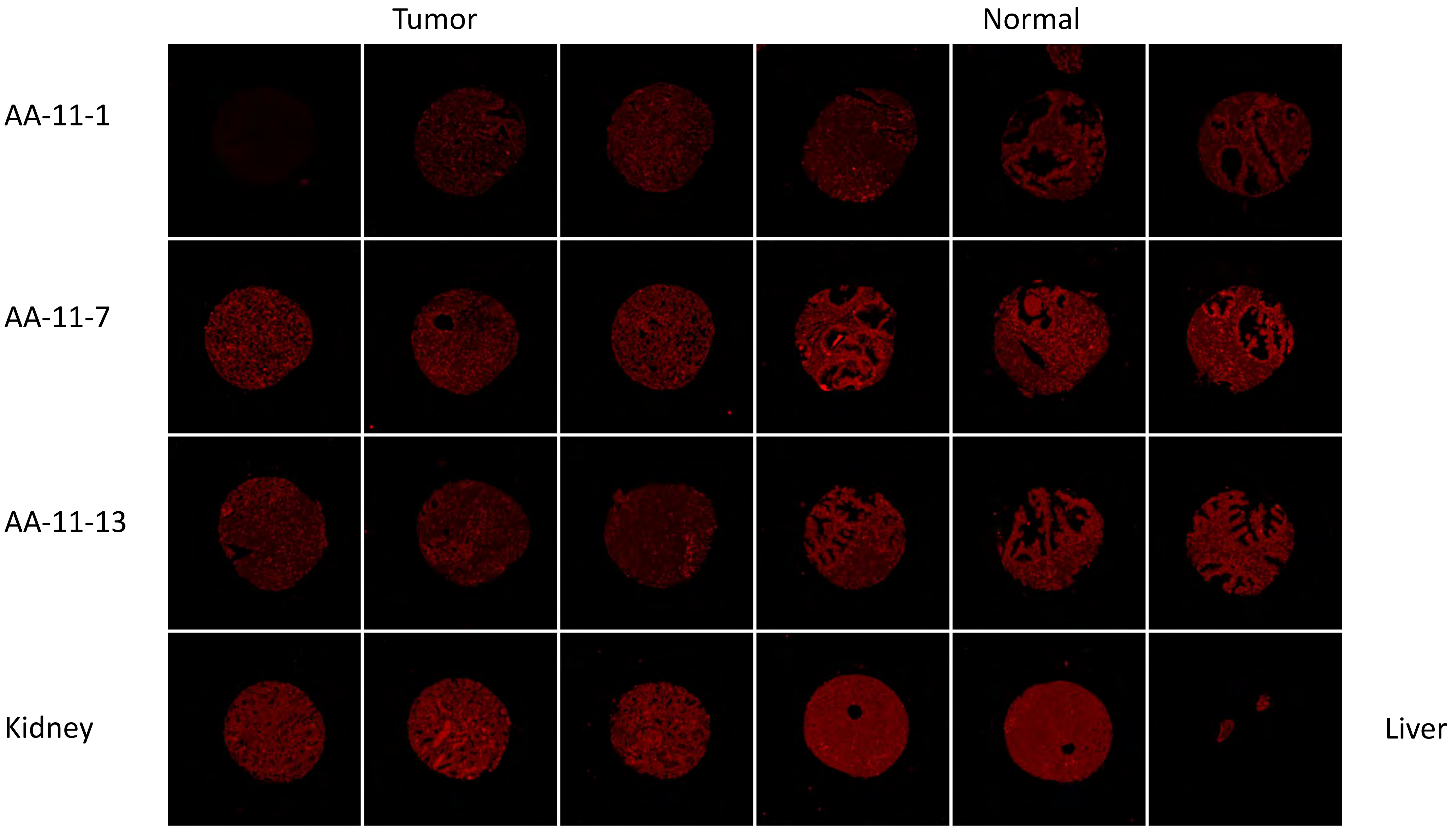

Row 11- oxRADD kidney and liver removed

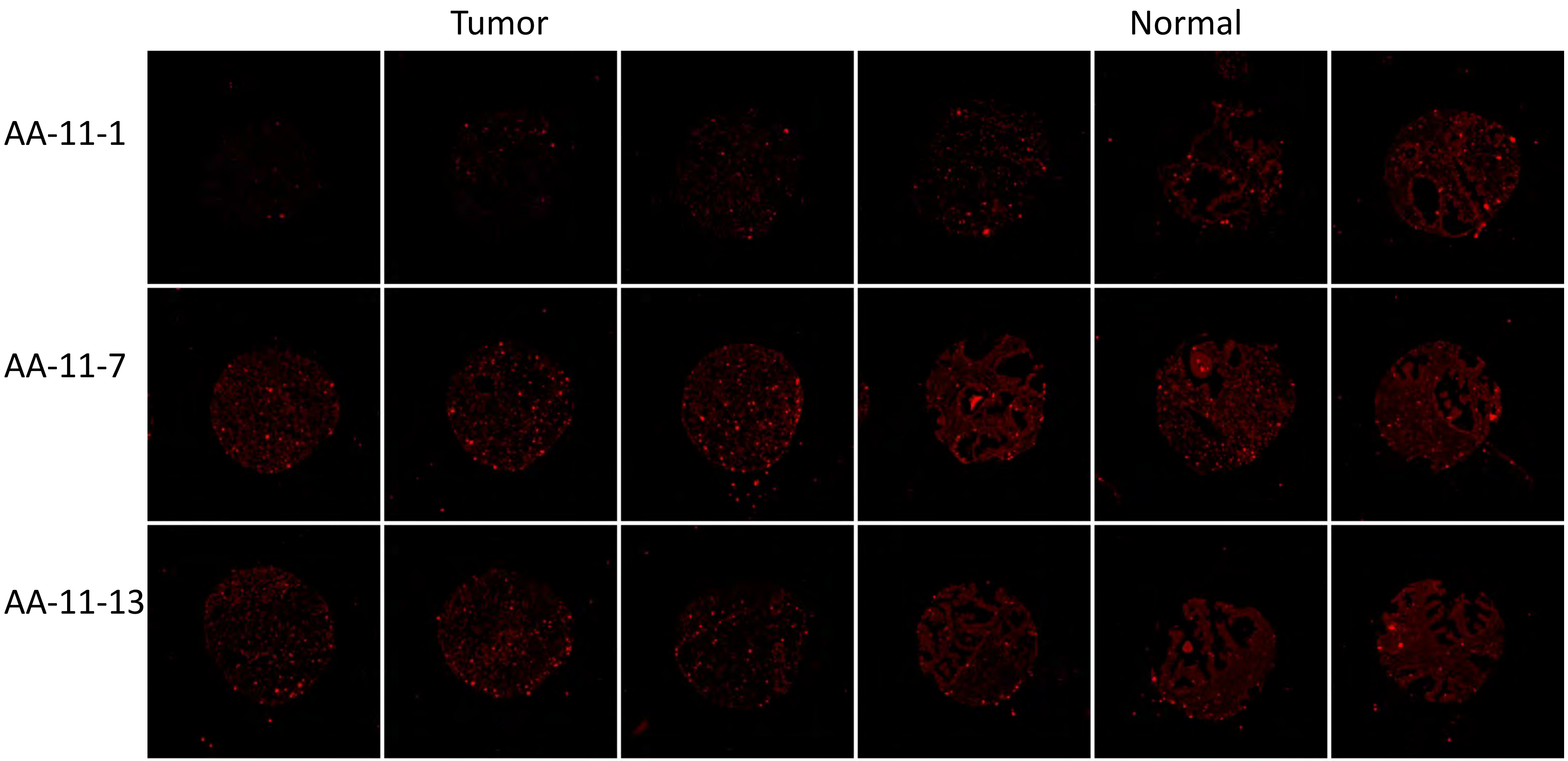

Row 11- UDG

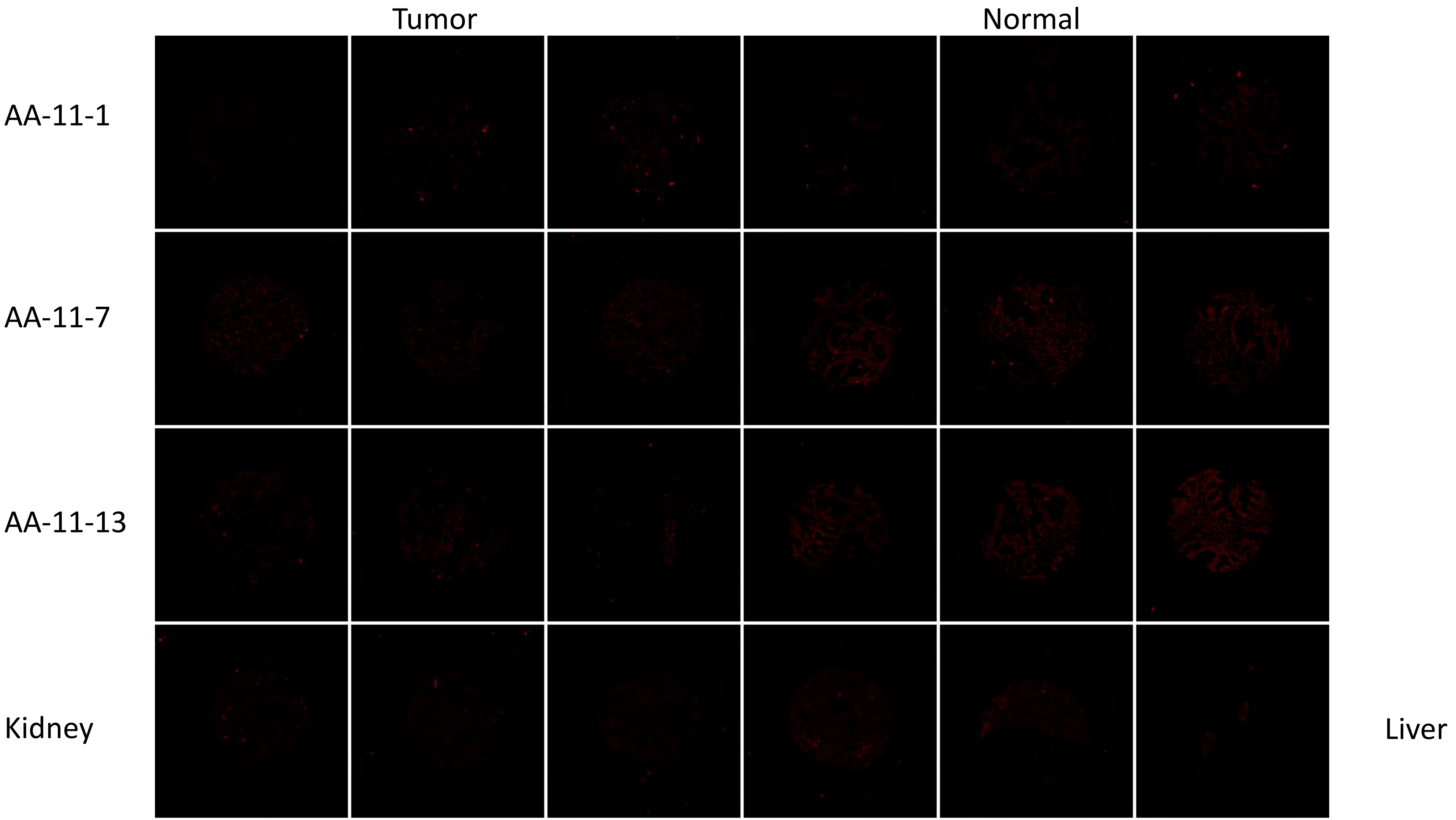

Row 11- T4PDG

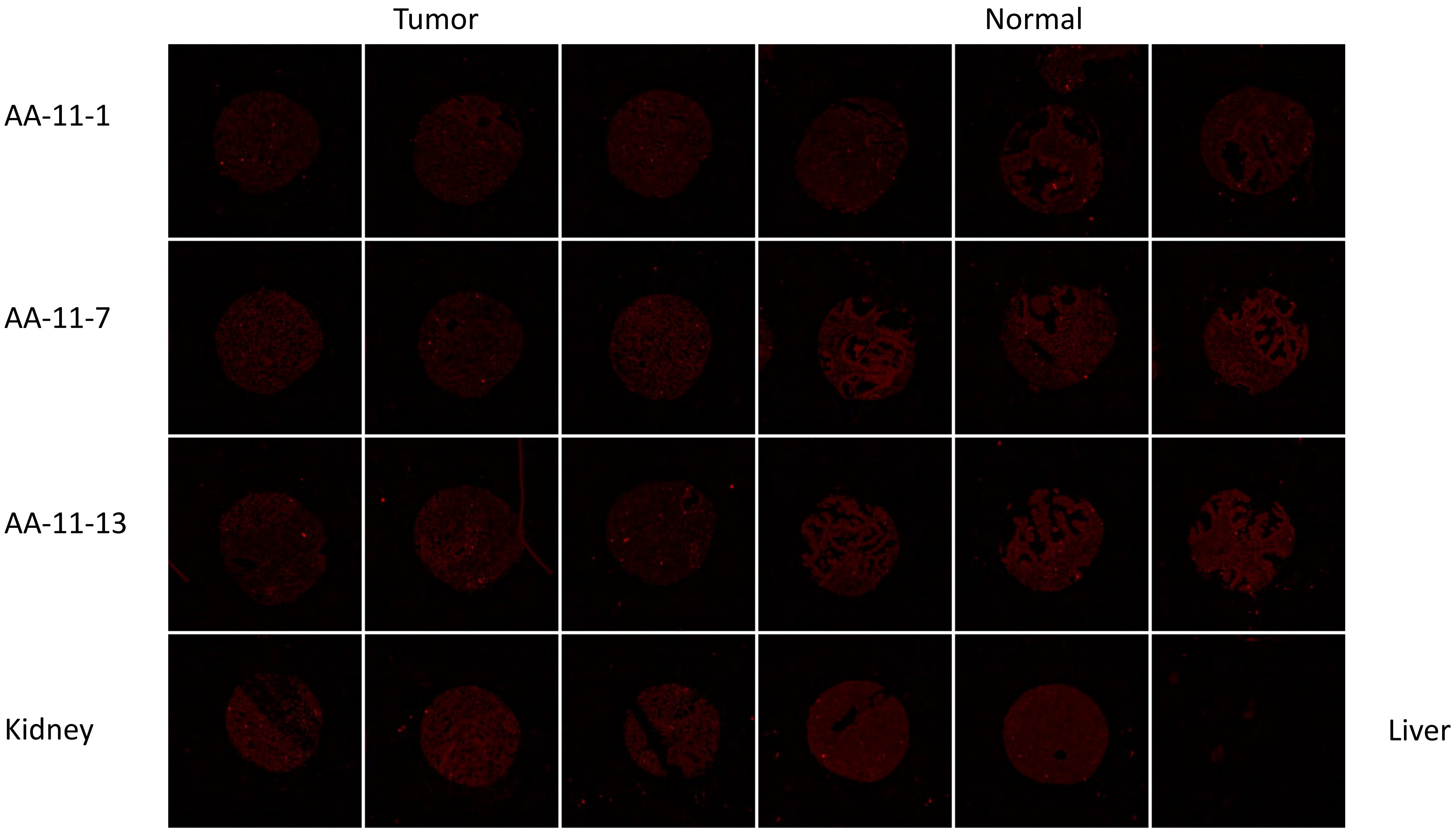

Row 11- XRCC1

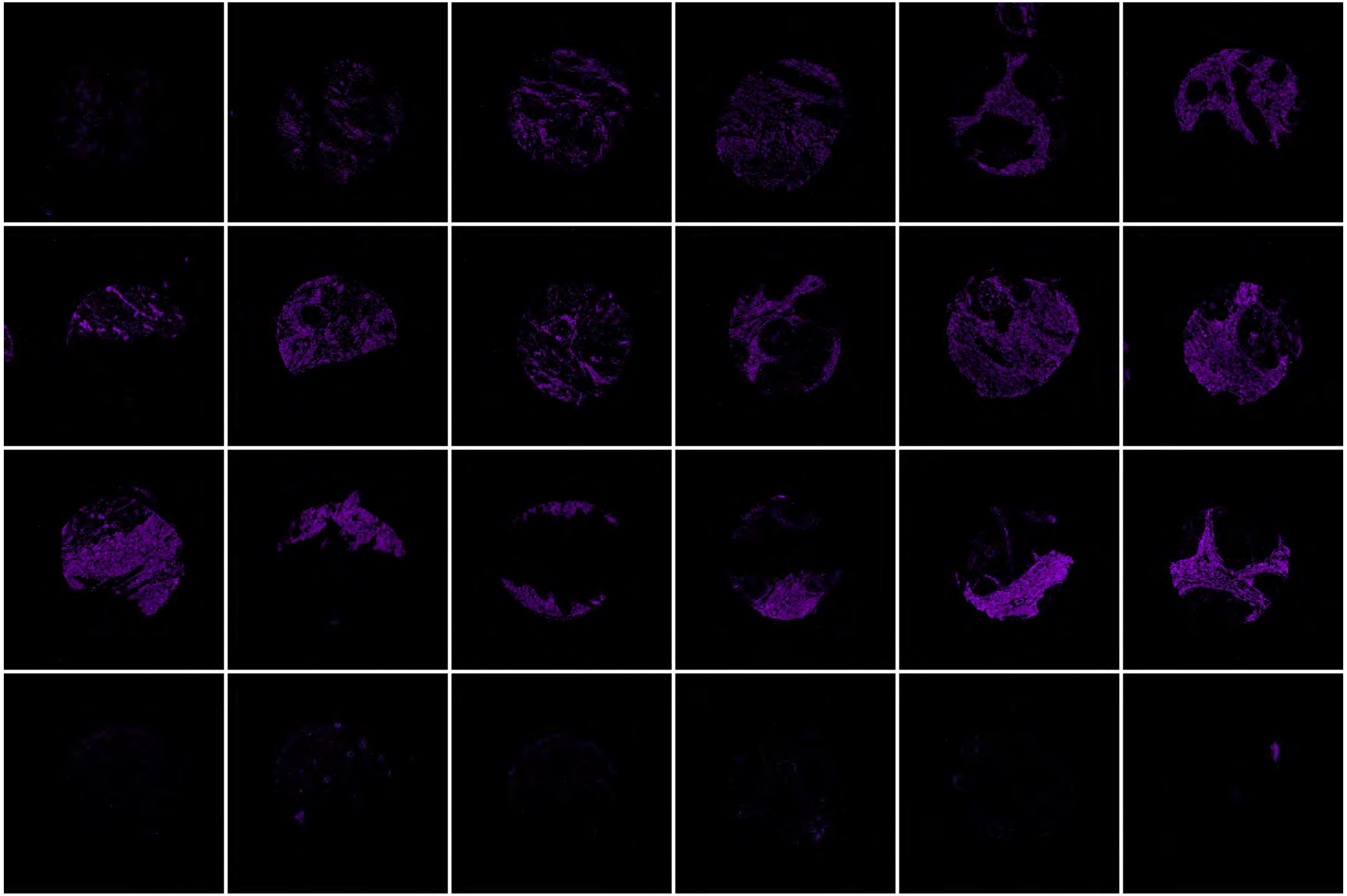

Tumor Normal

AA-11-1

AA-11-7

AA-11-13

Kidney

Liver

Row 11- PARP1

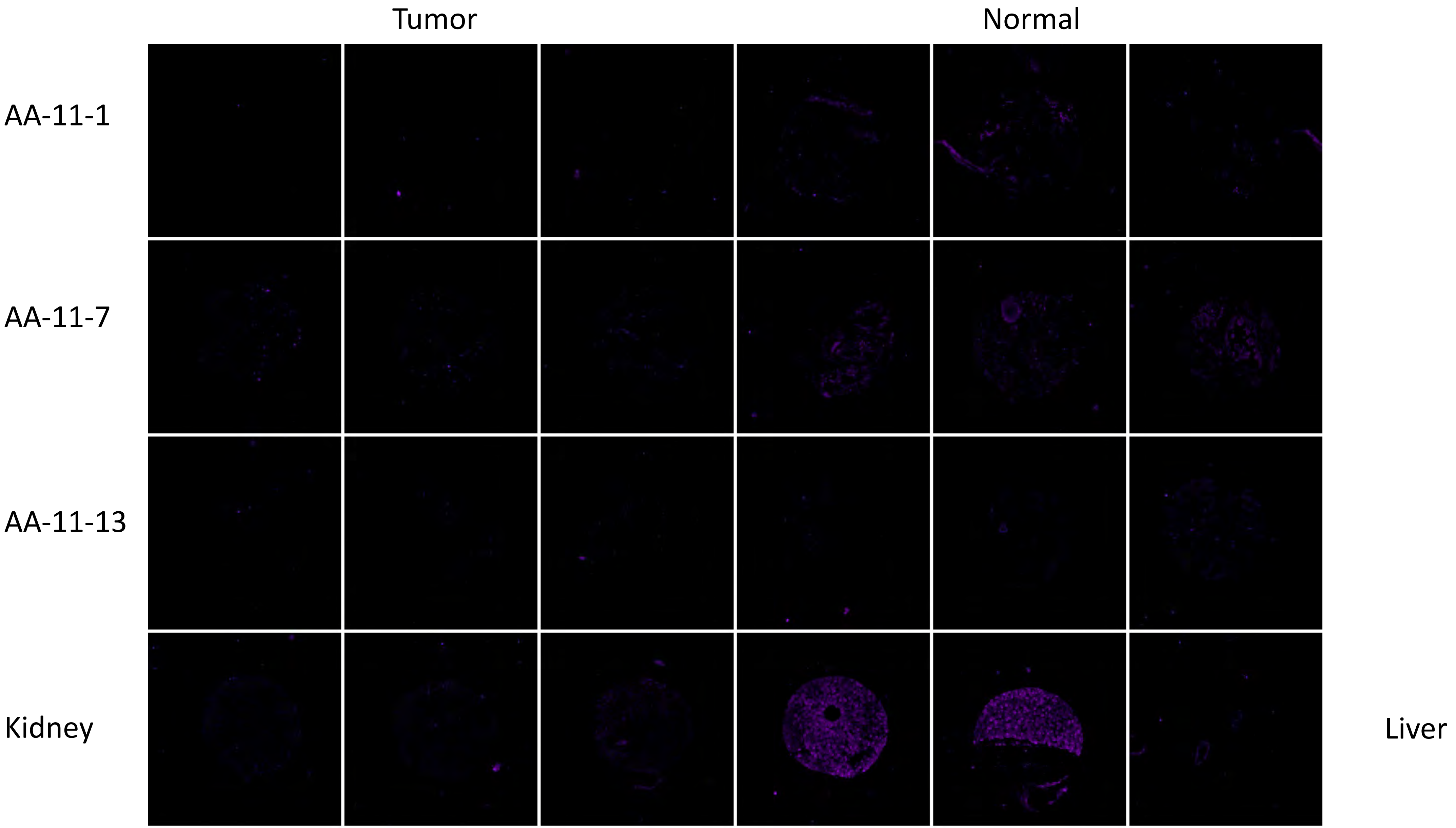

Row 11- UNG

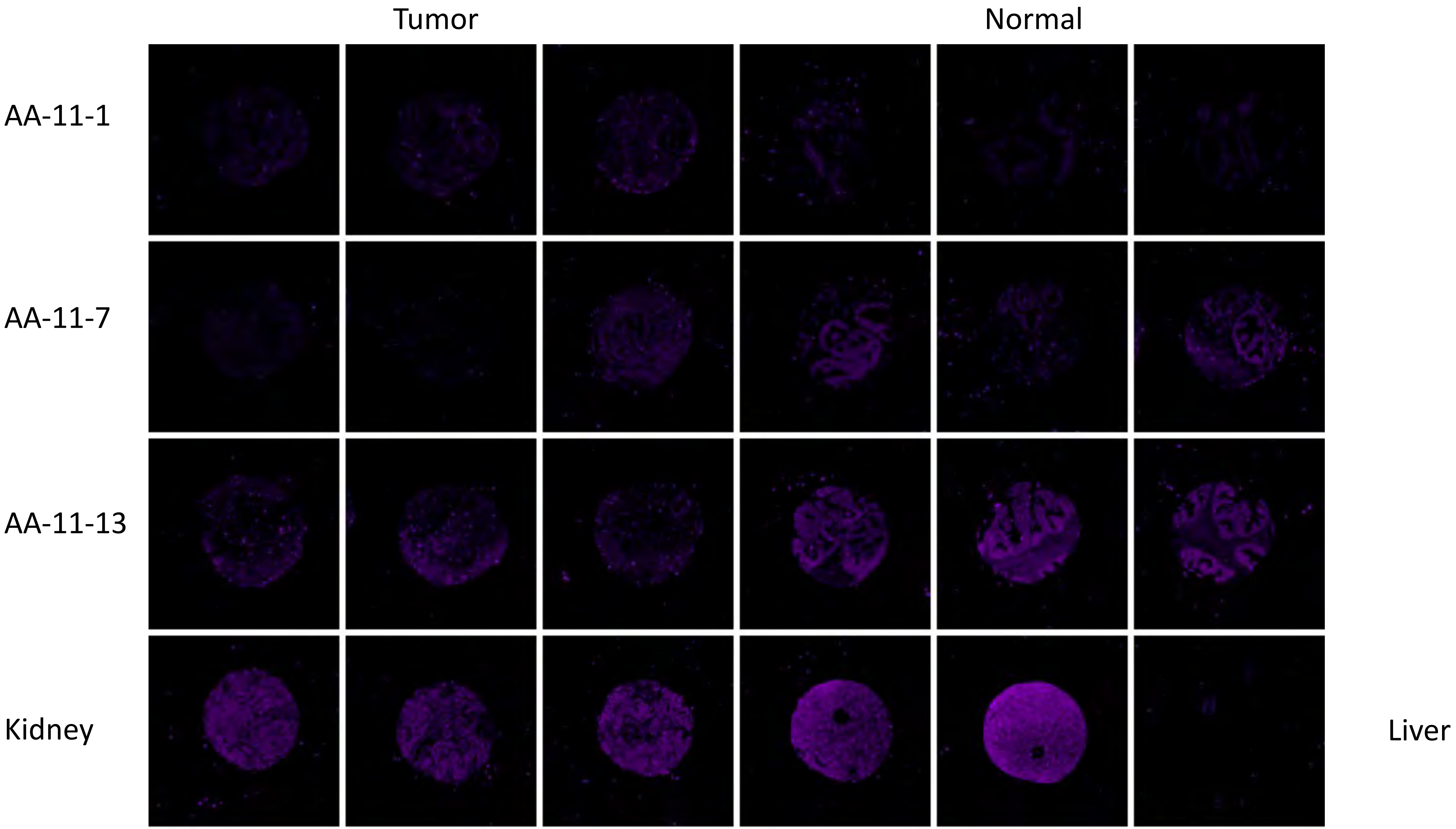

Row 12- Full RADD

Tumor Normal

AA-12-1

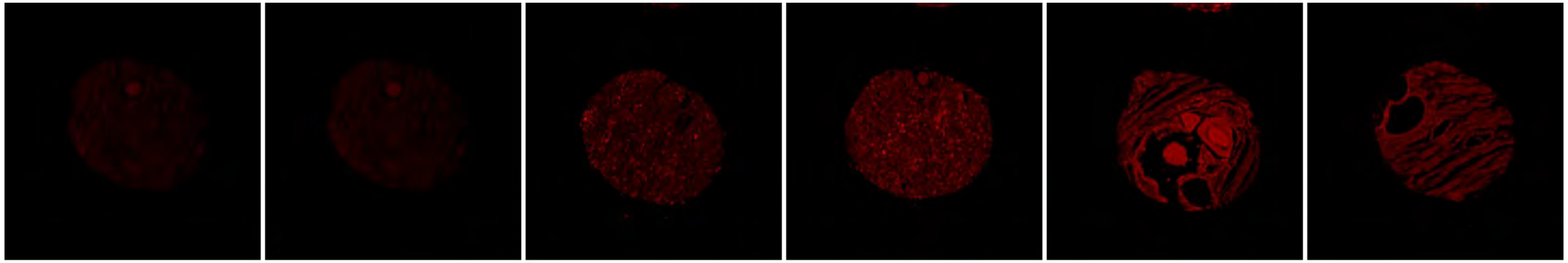

Row 12- oxRADD

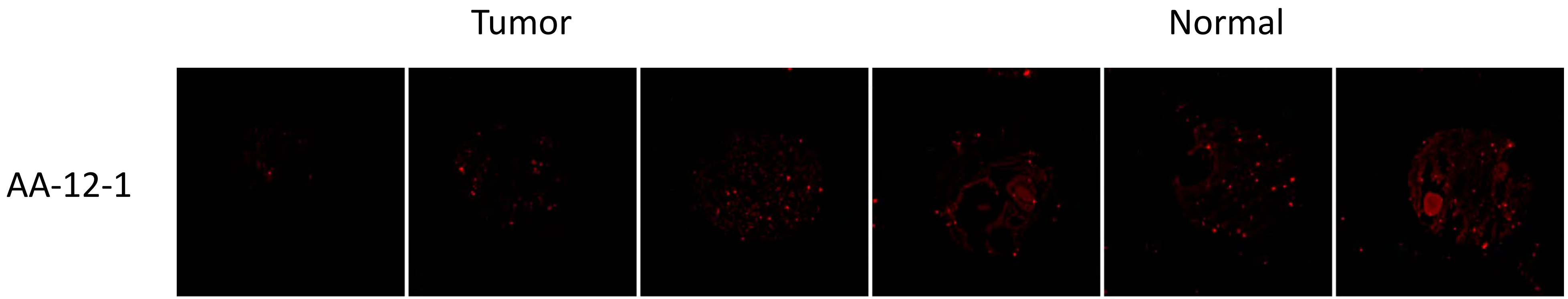

Row 12- UDG

Tumor Normal

AA-12-1

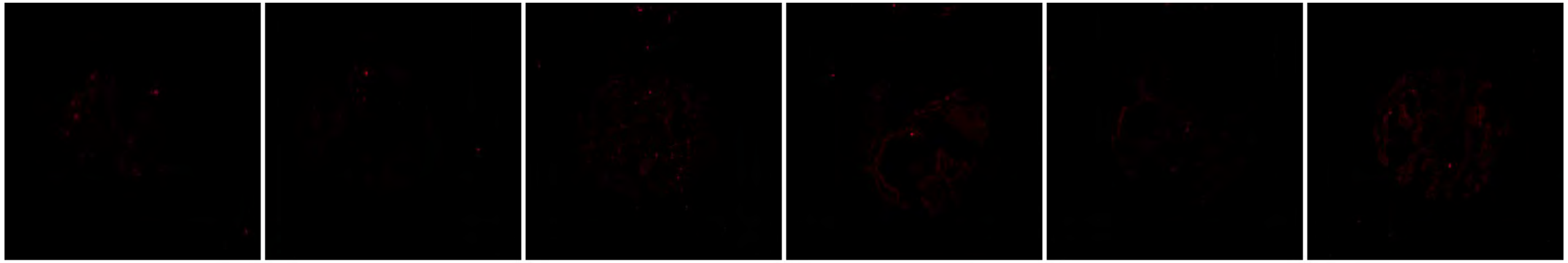

Row 12- T4PDG

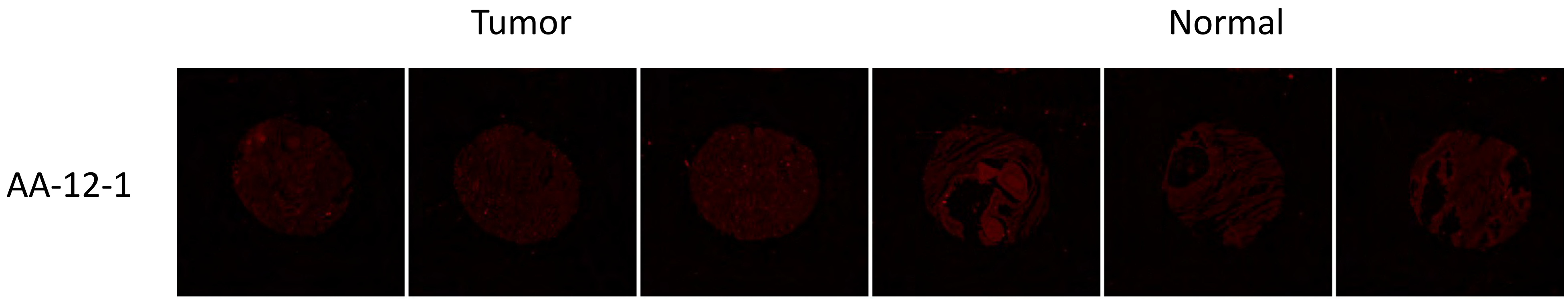

Row 12- XRCC1

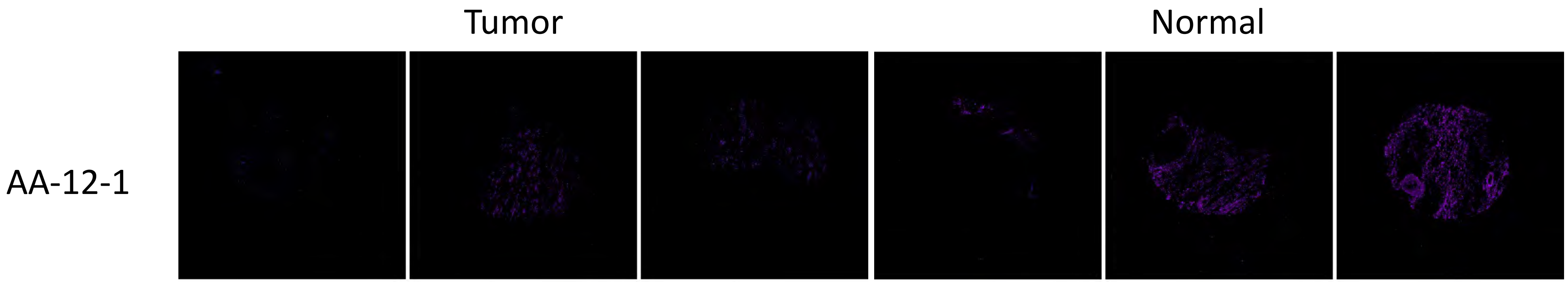

Row 12- PARP1

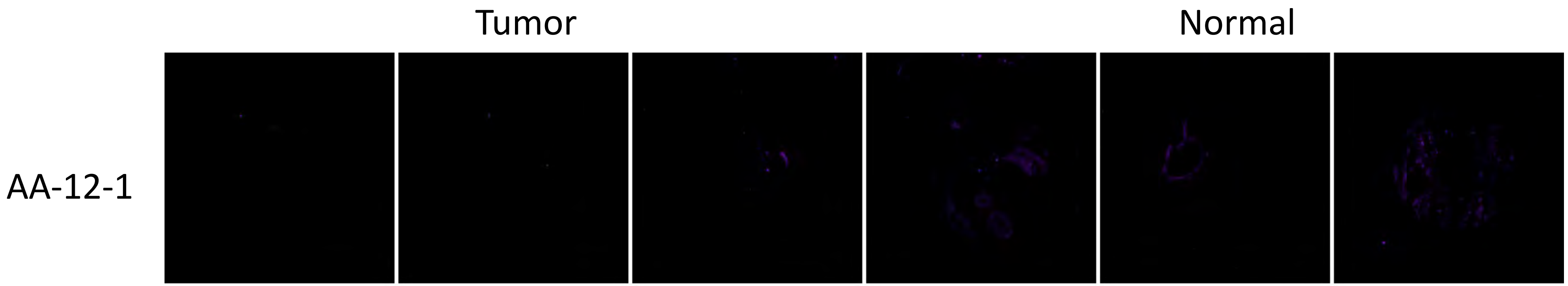

Row 12- UNG

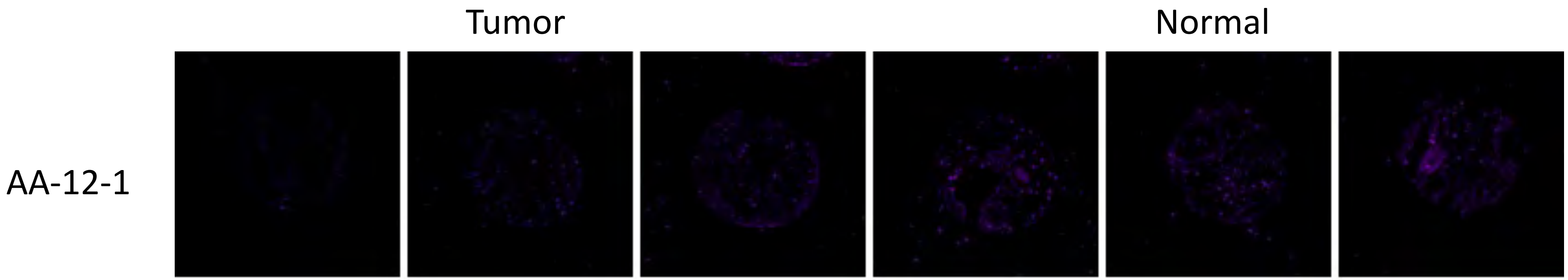

Row 1 Full RADD

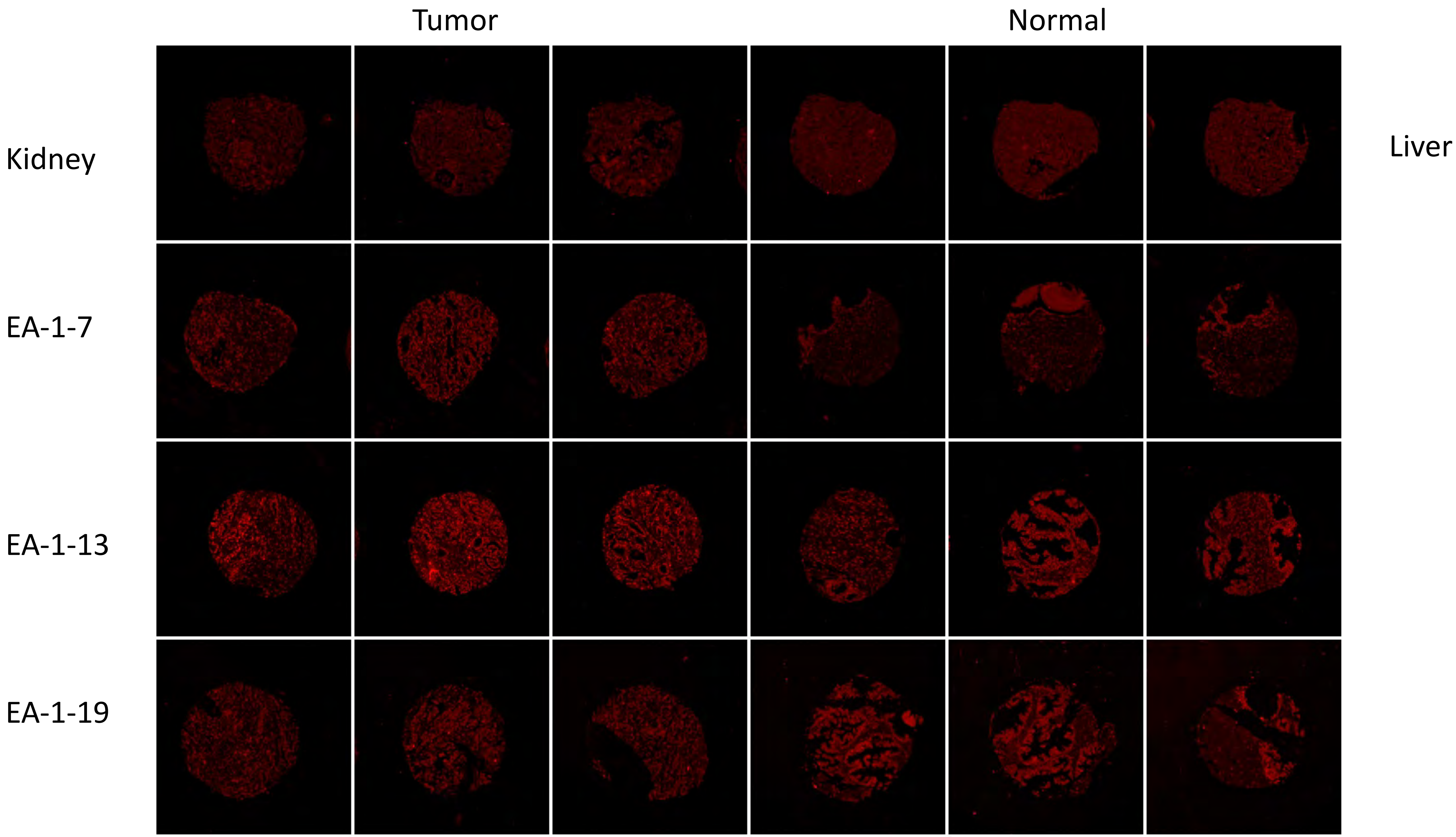

Row 1 oxRADD – Kidney and liver removed

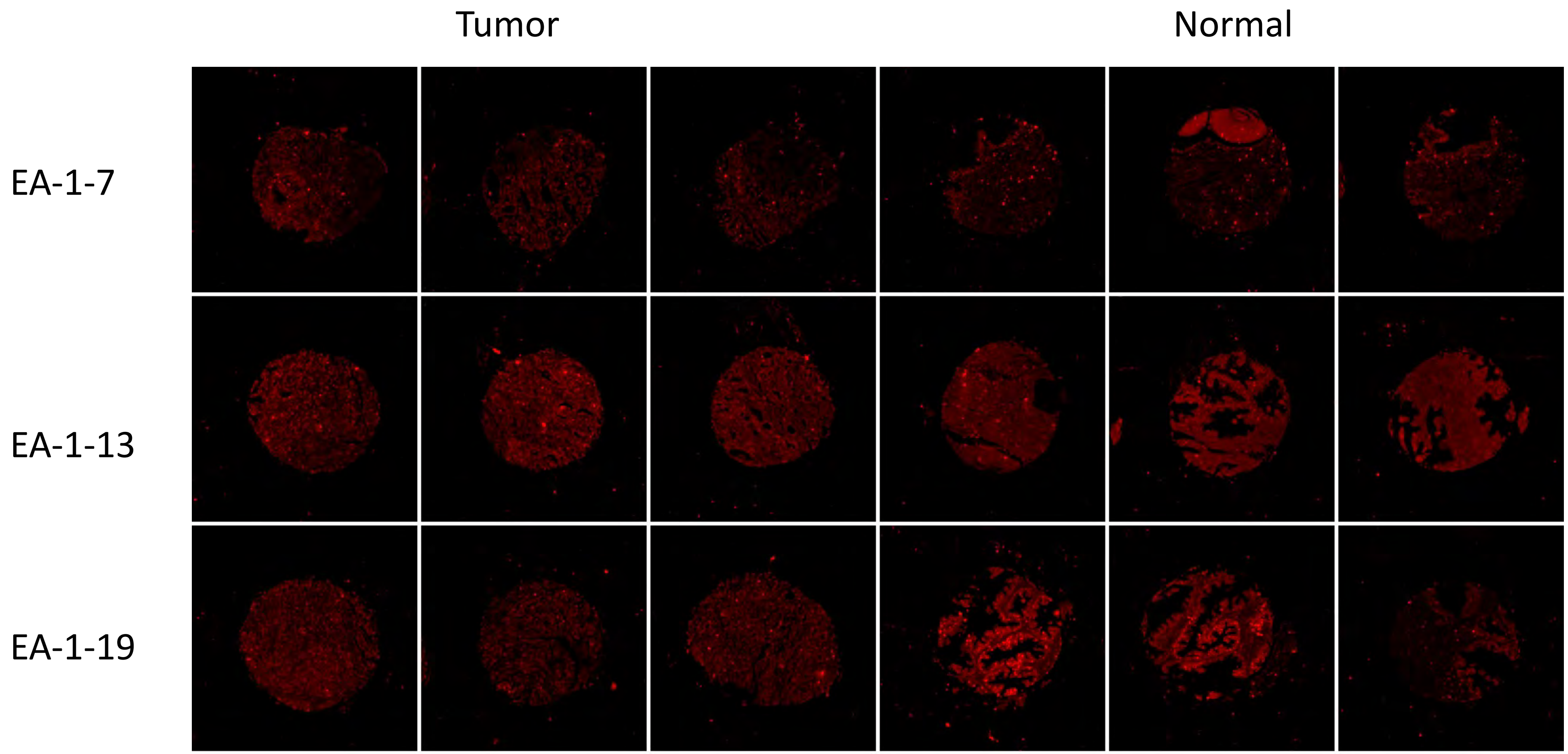

Row1- UDG

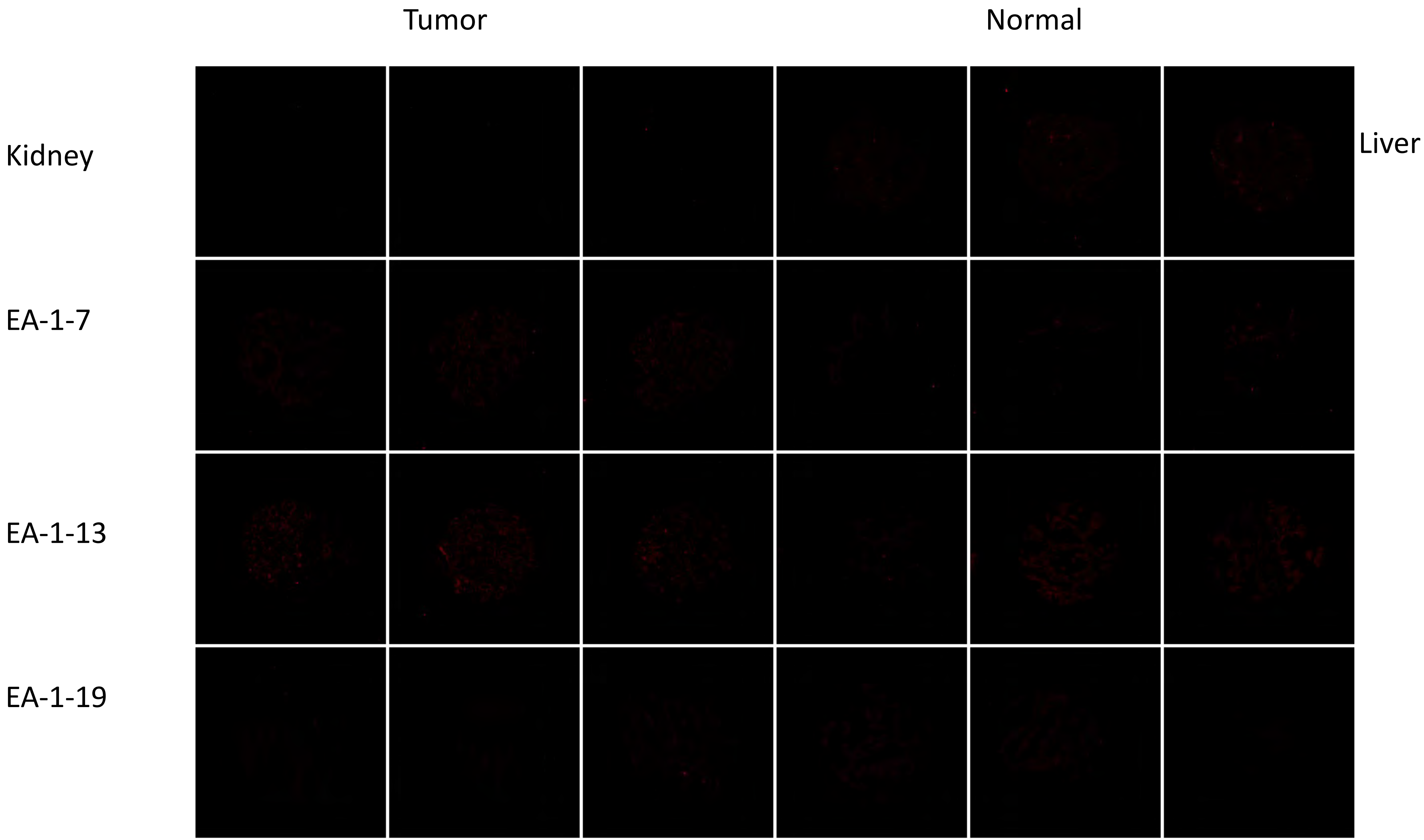

Row 1- T4PDG

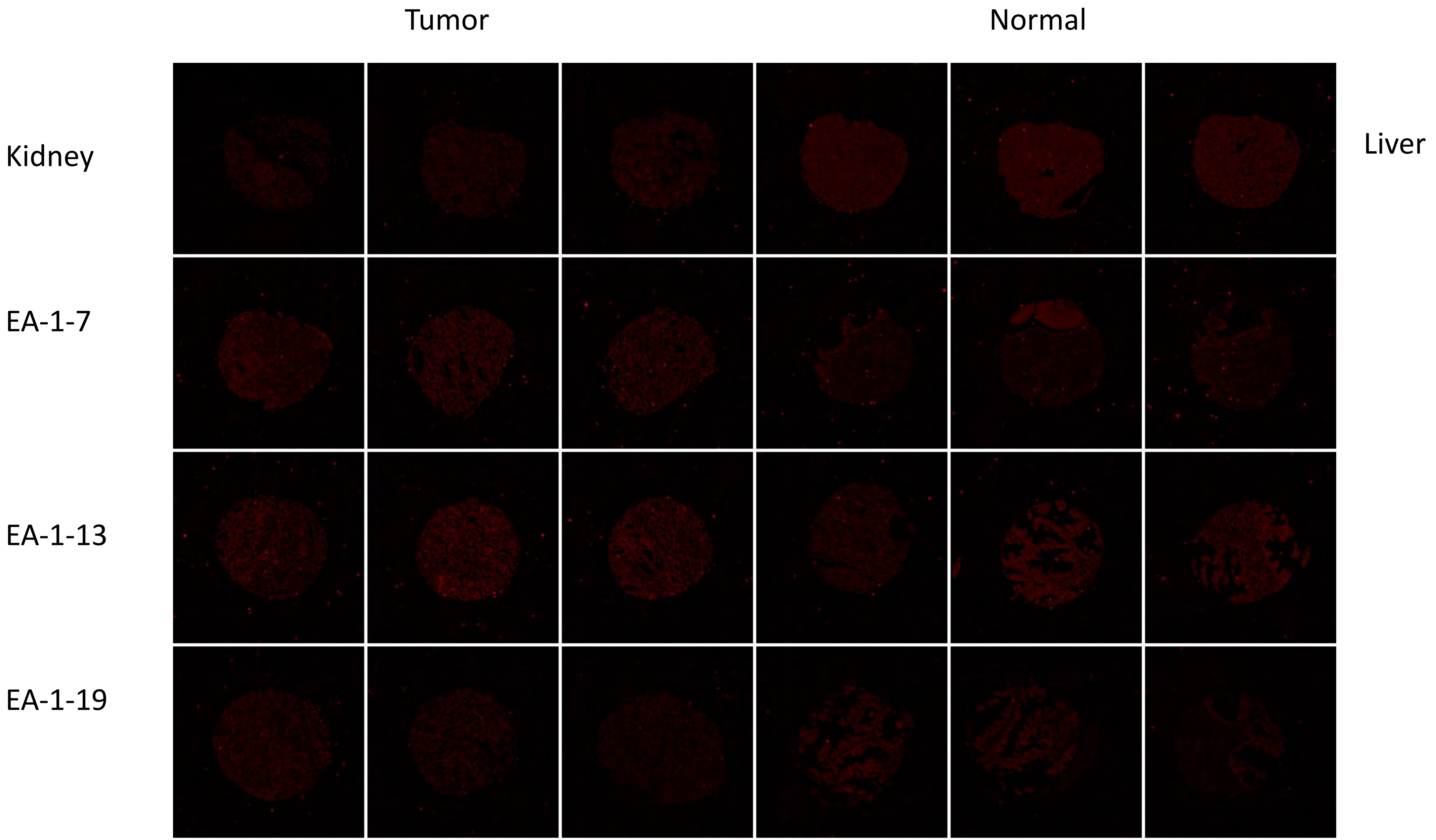

Row 1- XRCC1

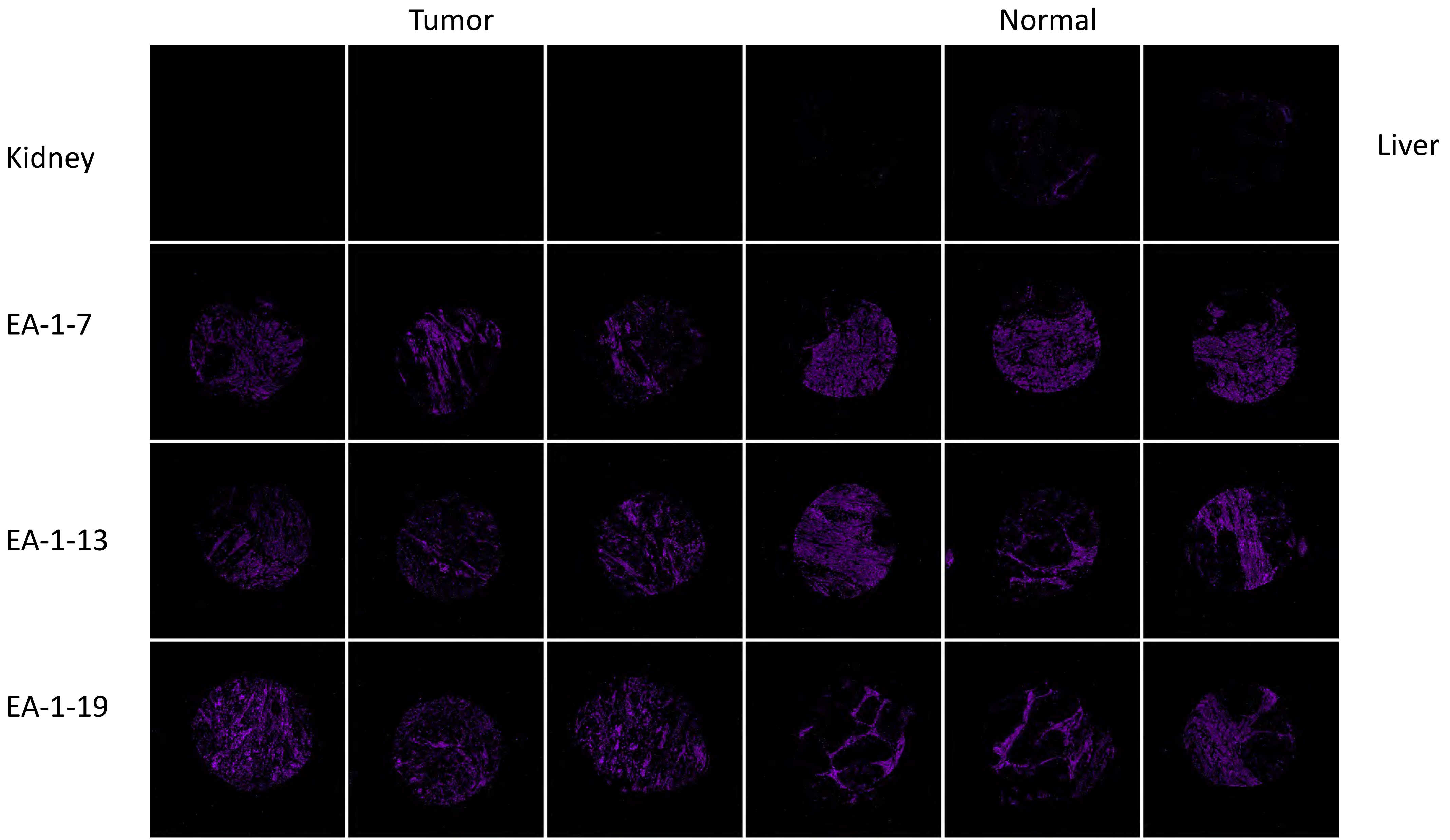

Row 1 PARP1

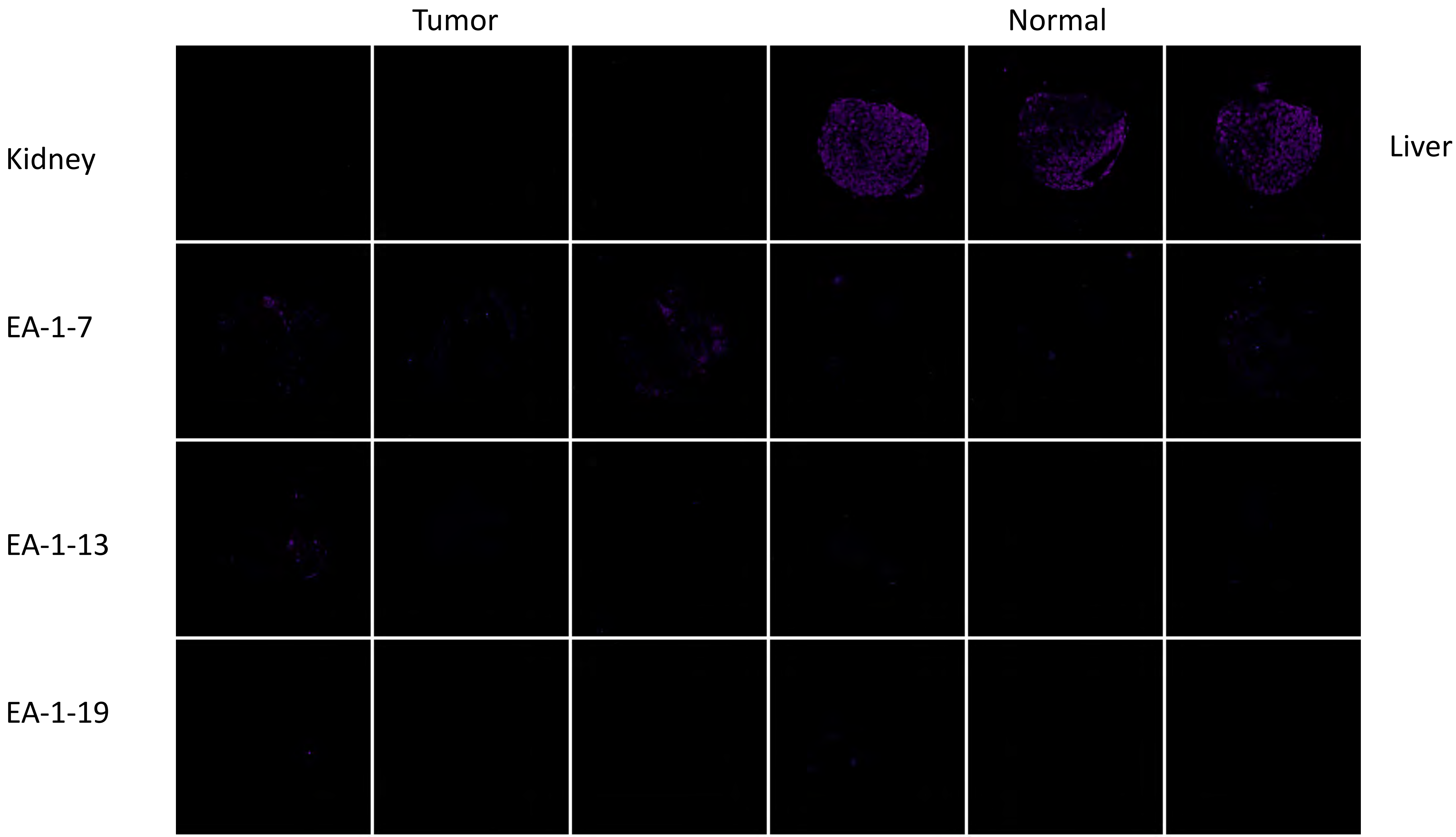

Row1- UNG

Tumor Normal

Kidney

EA-1-7

EA-1-13

EA-1-19

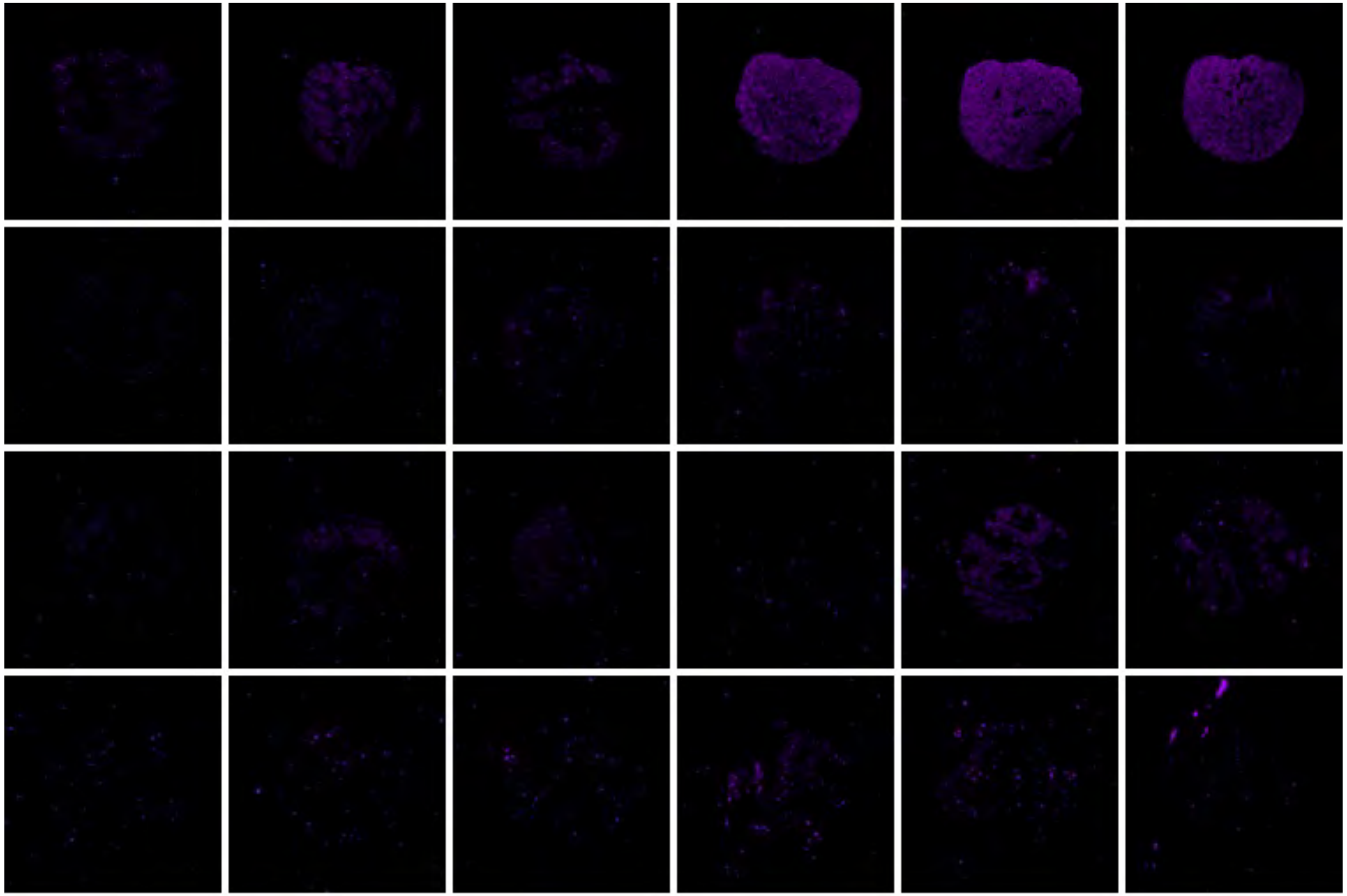

Liver

Row 2- Full RADD

Tumor Normal

EA-2-1

EA-2-7

EA-2-13

EA-2-19

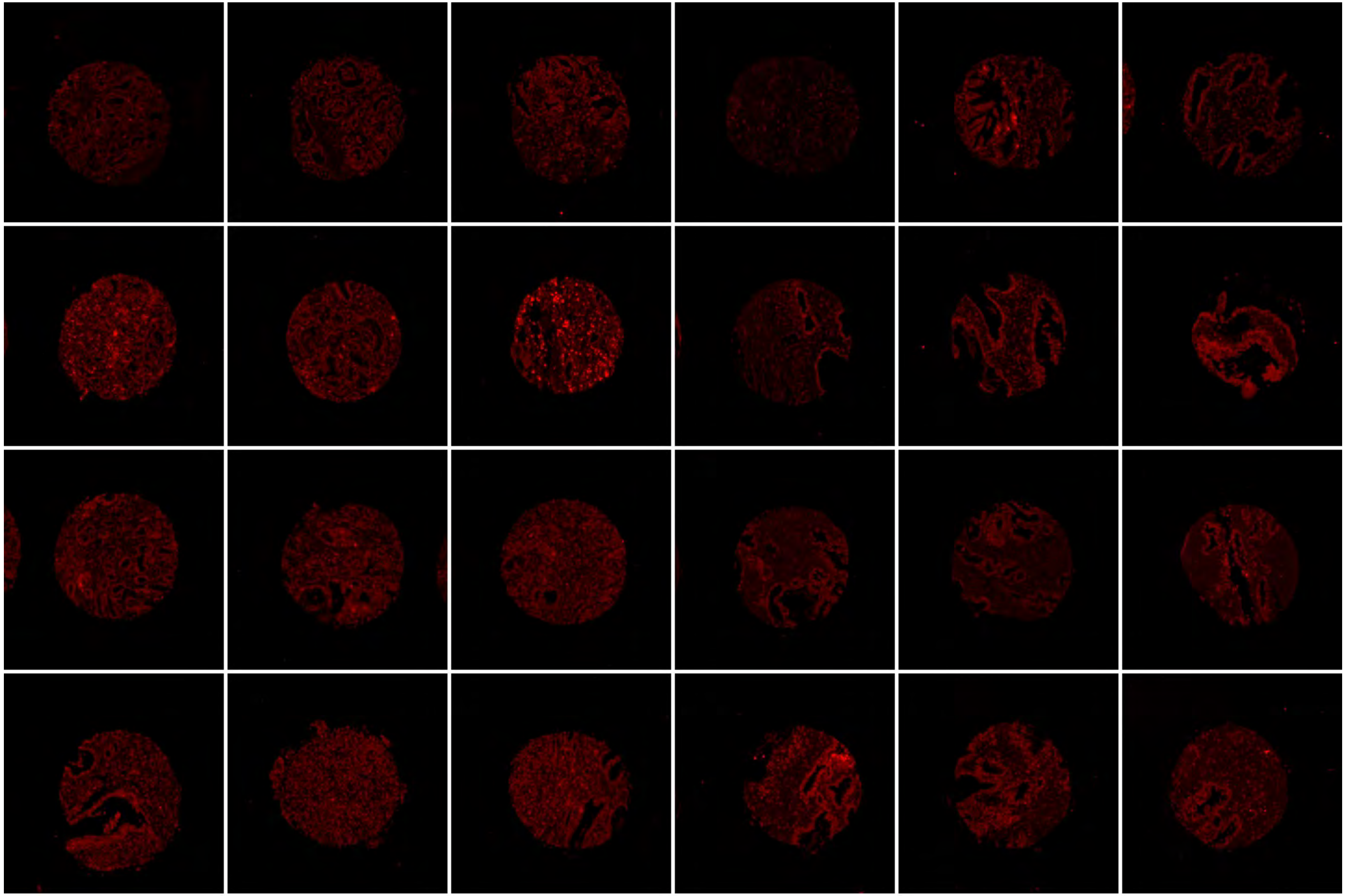

Row 2- oxRADD

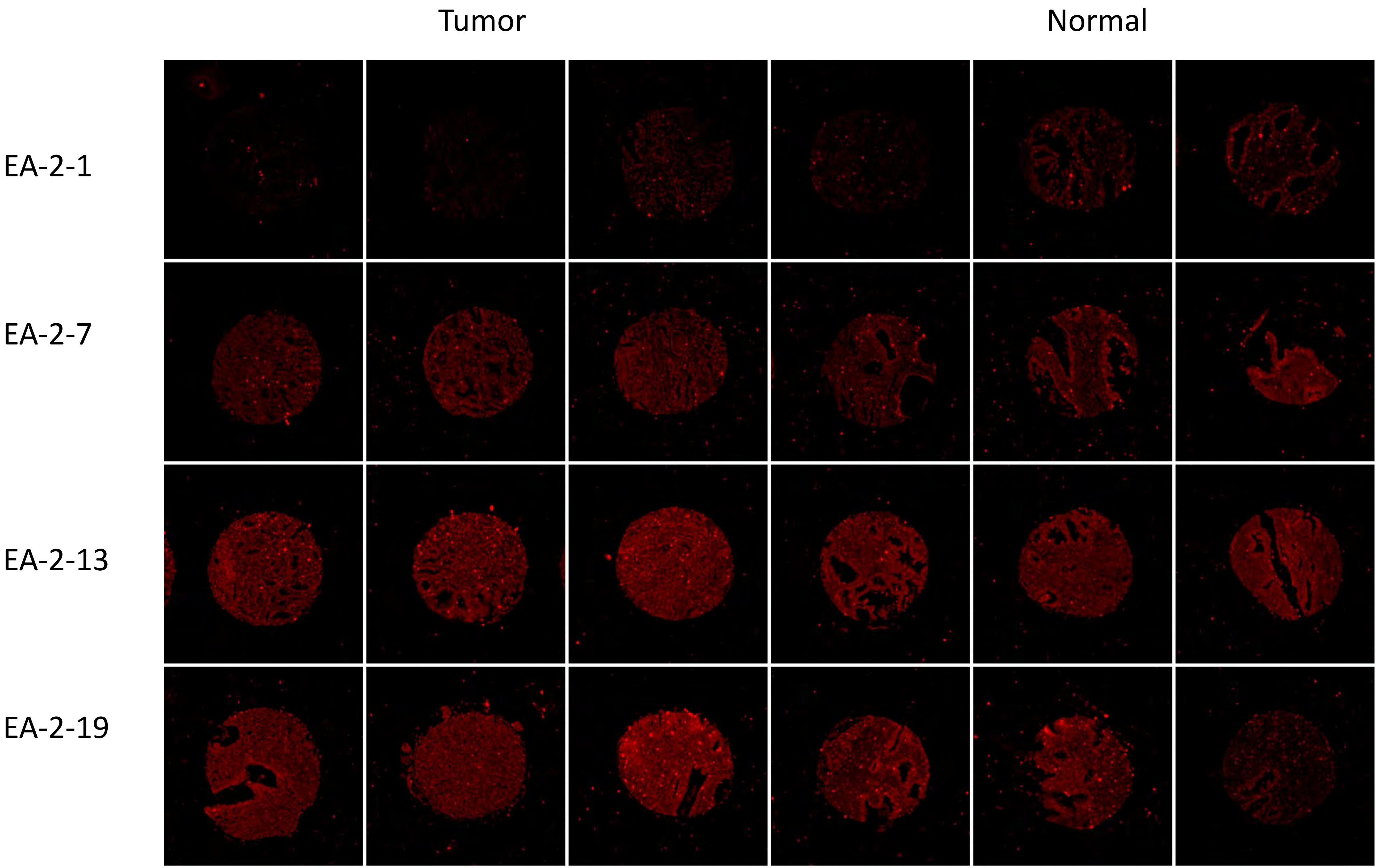

Row 2- UDG

Tumor Normal

EA-2-1

EA-2-7

EA-2-13

EA-2-19

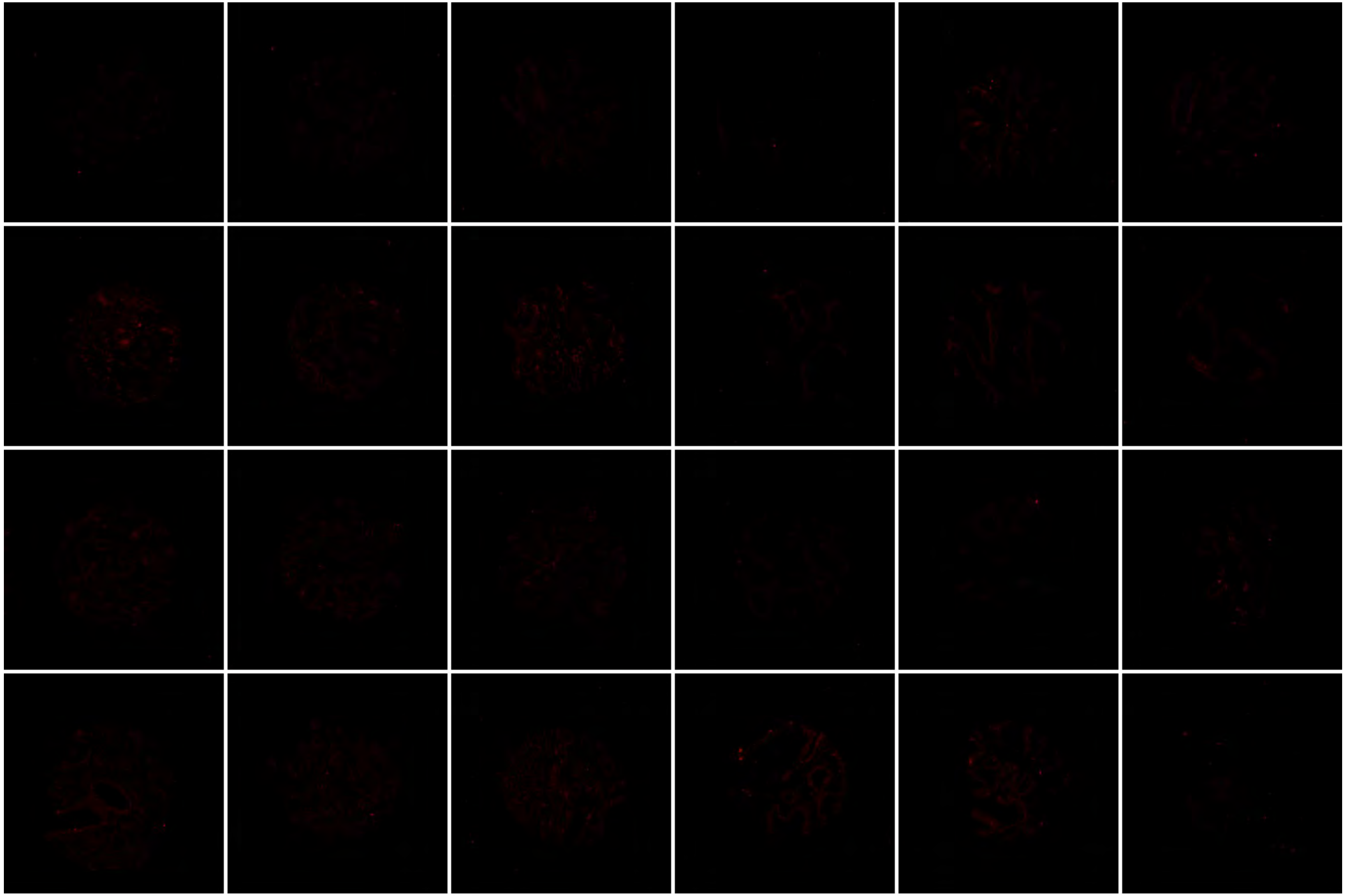

Row 2- T4PDG

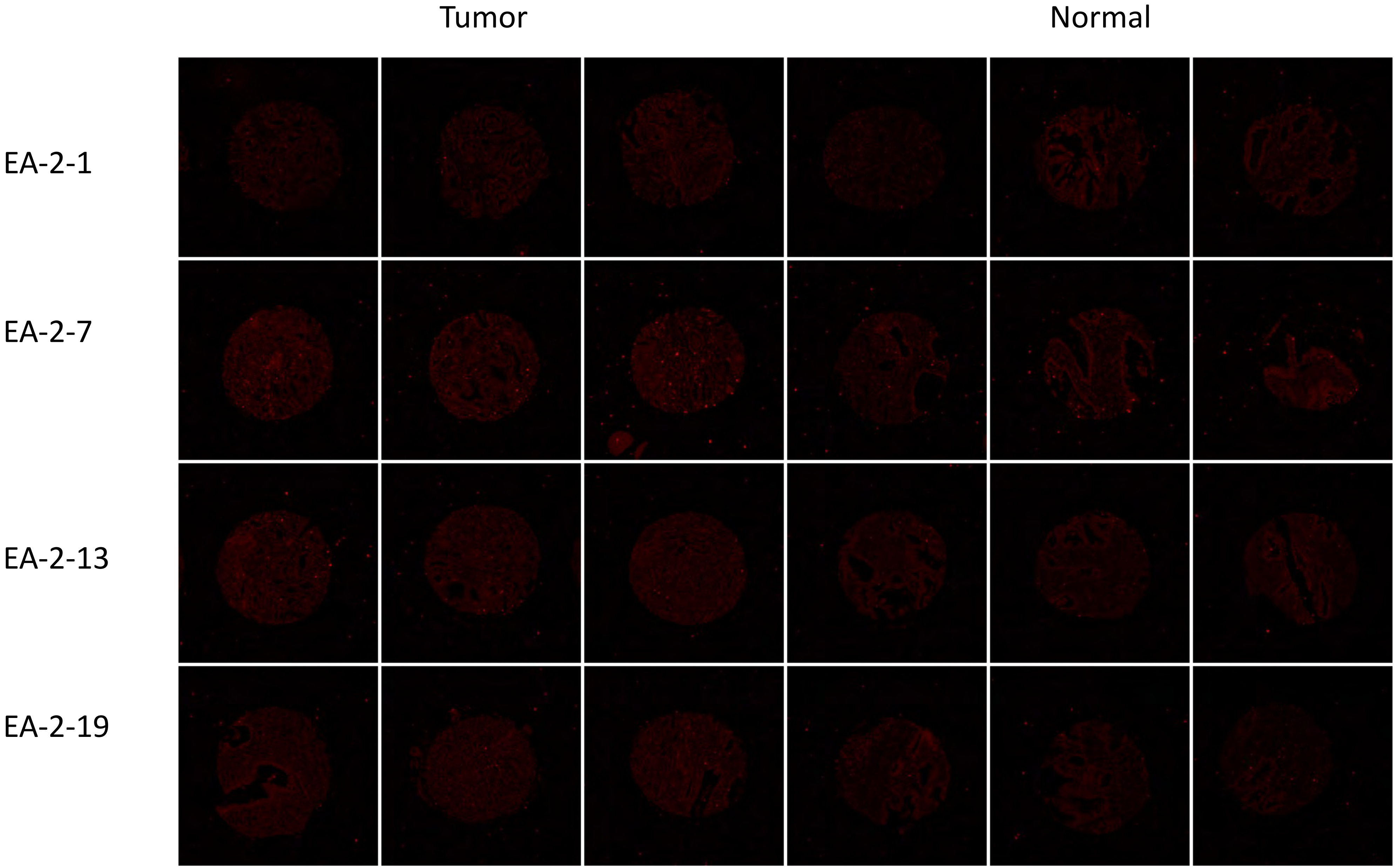

Row 2- XRCC1

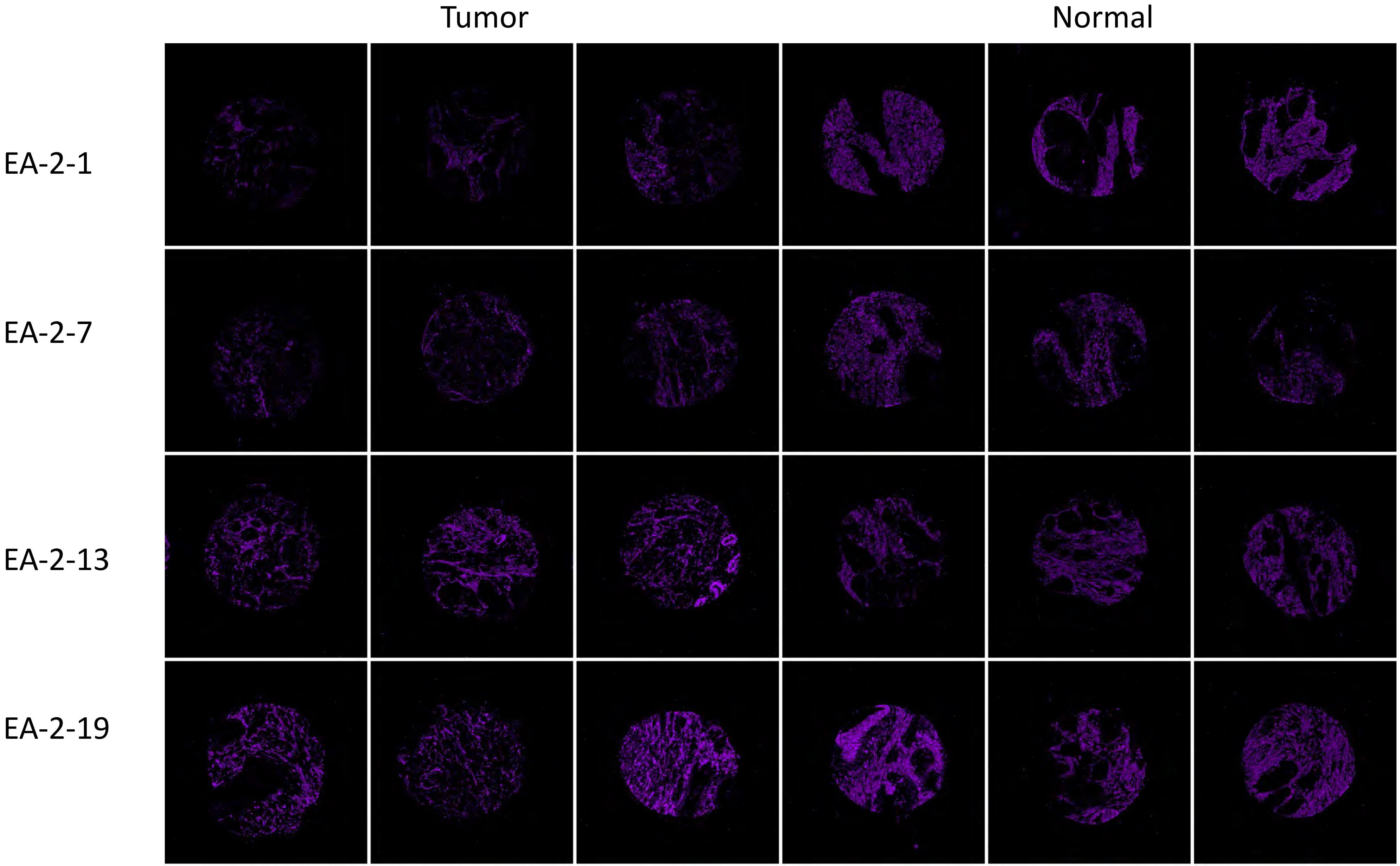

Row 2- PARP1

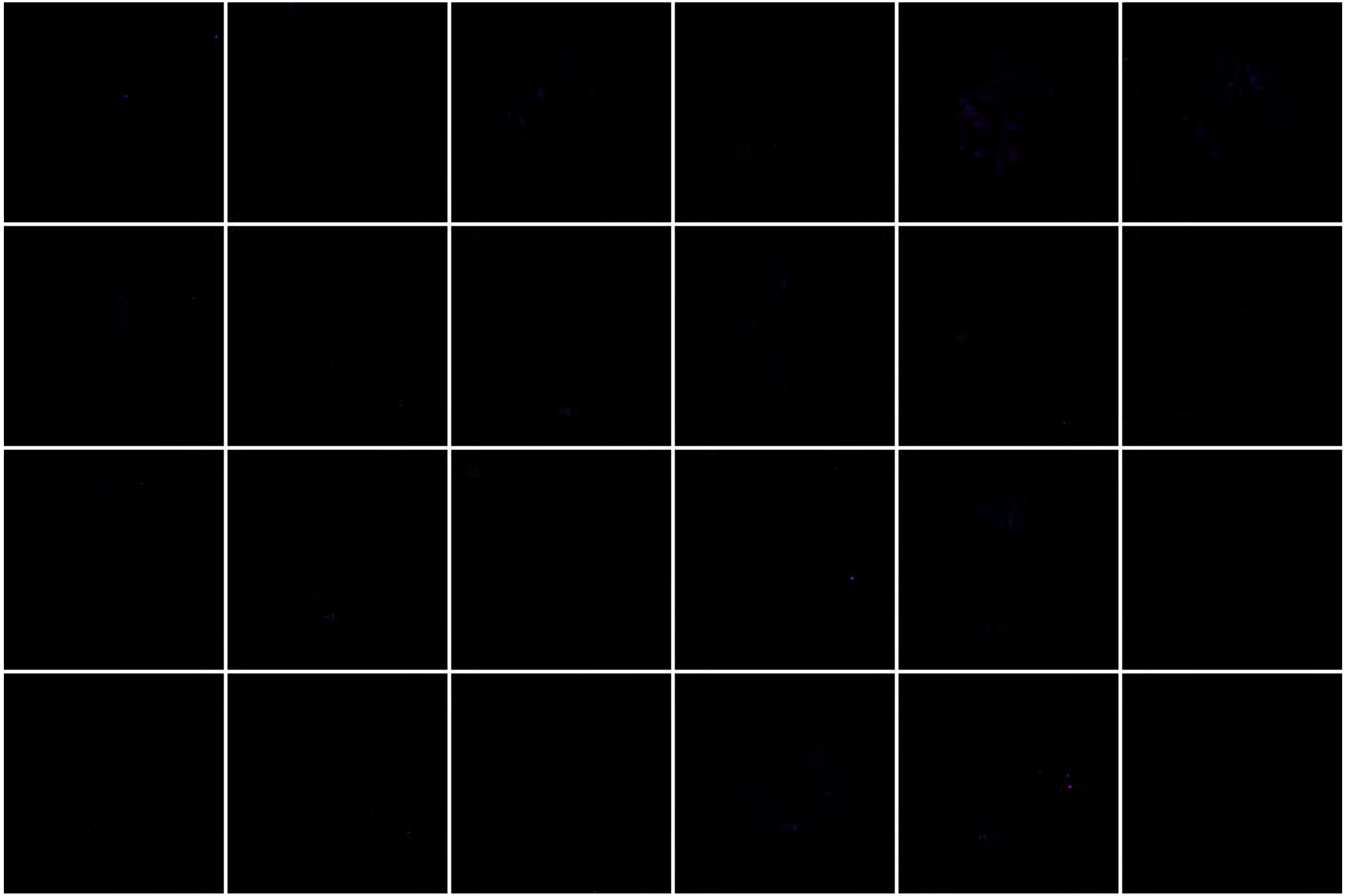

Row 2- UNG

Tumor Normal

EA-2-1

EA-2-7

EA-2-13

EA-2-19

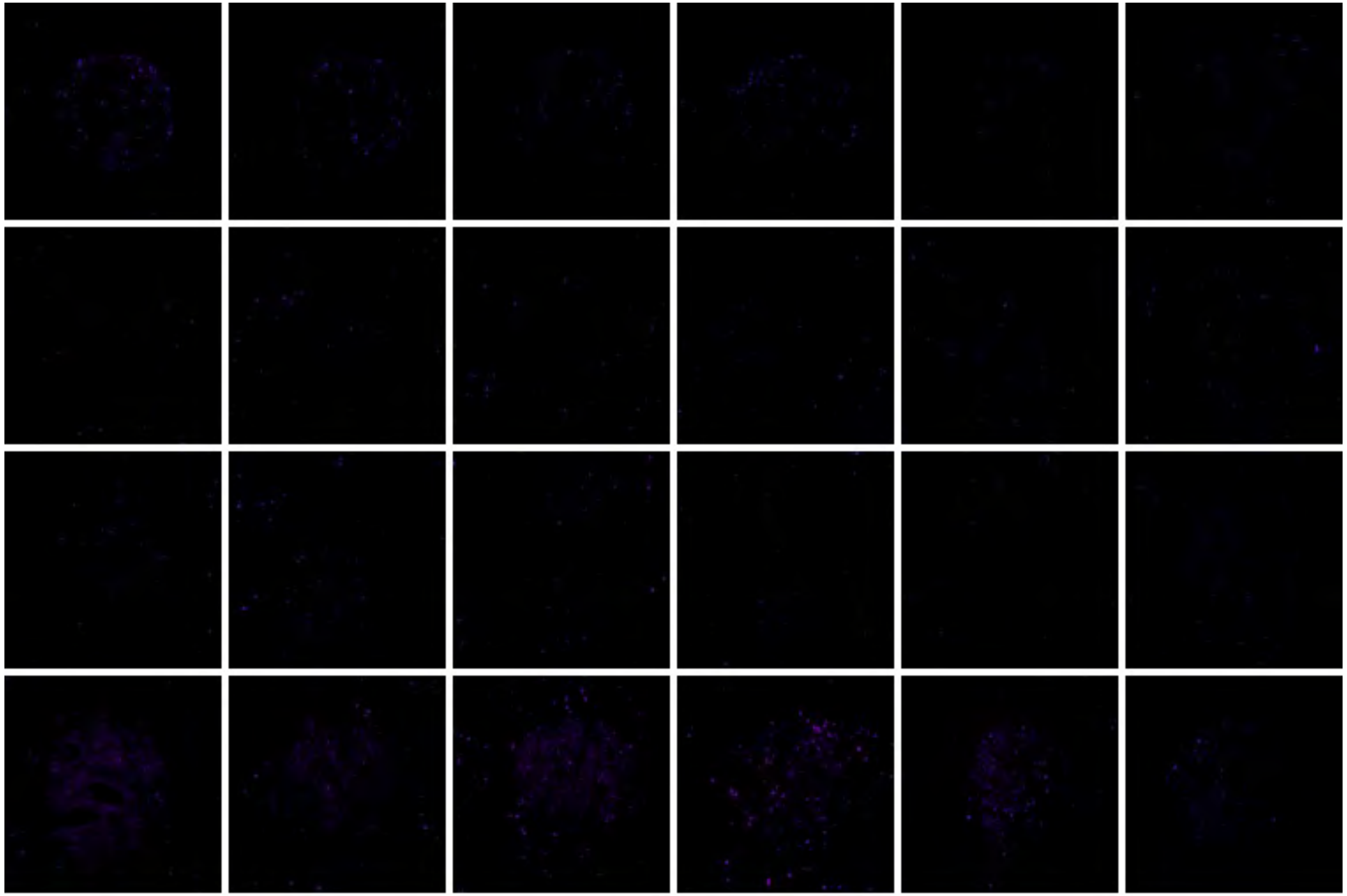

Row 3- Full RADD

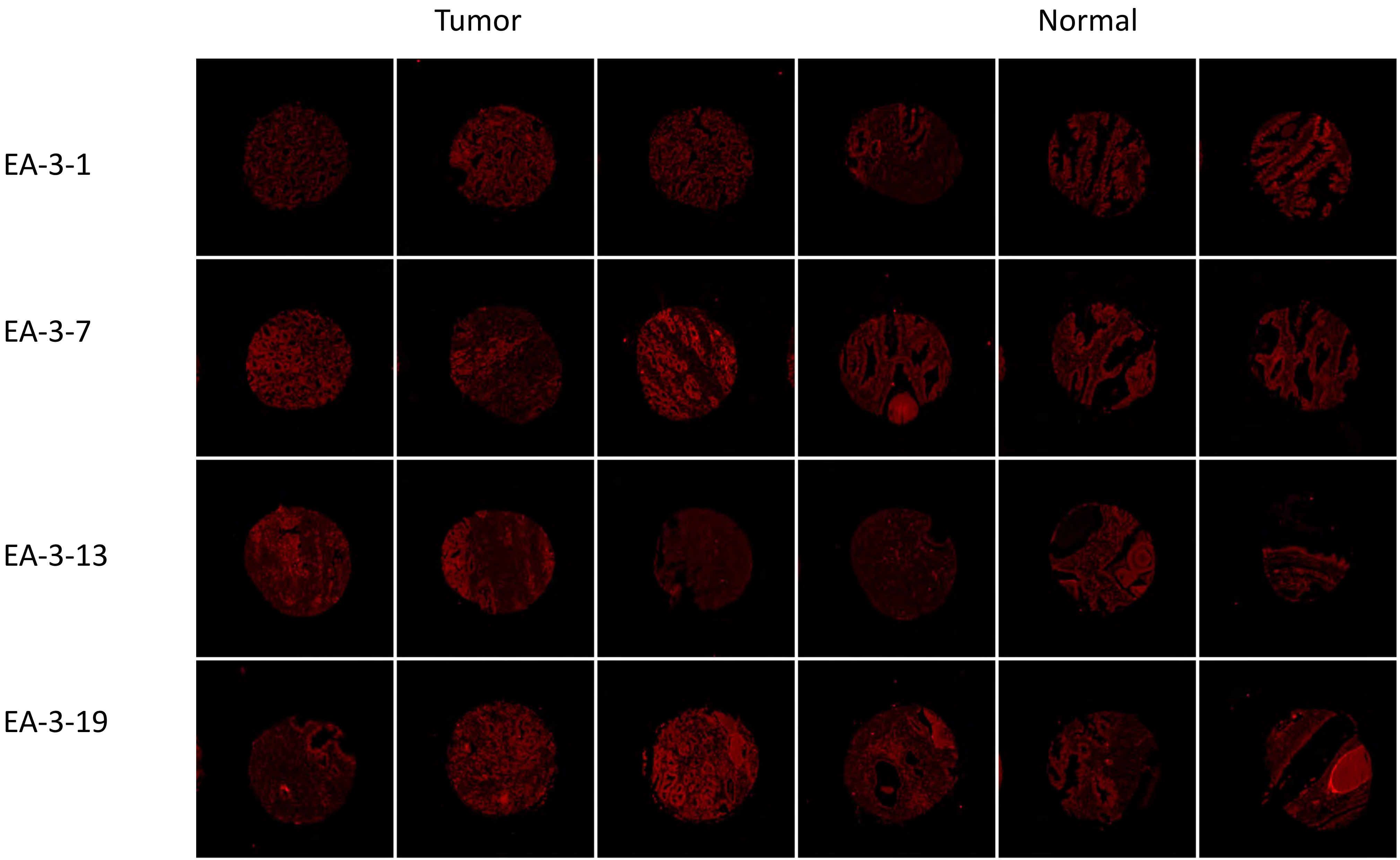

Row 3- oxRADD

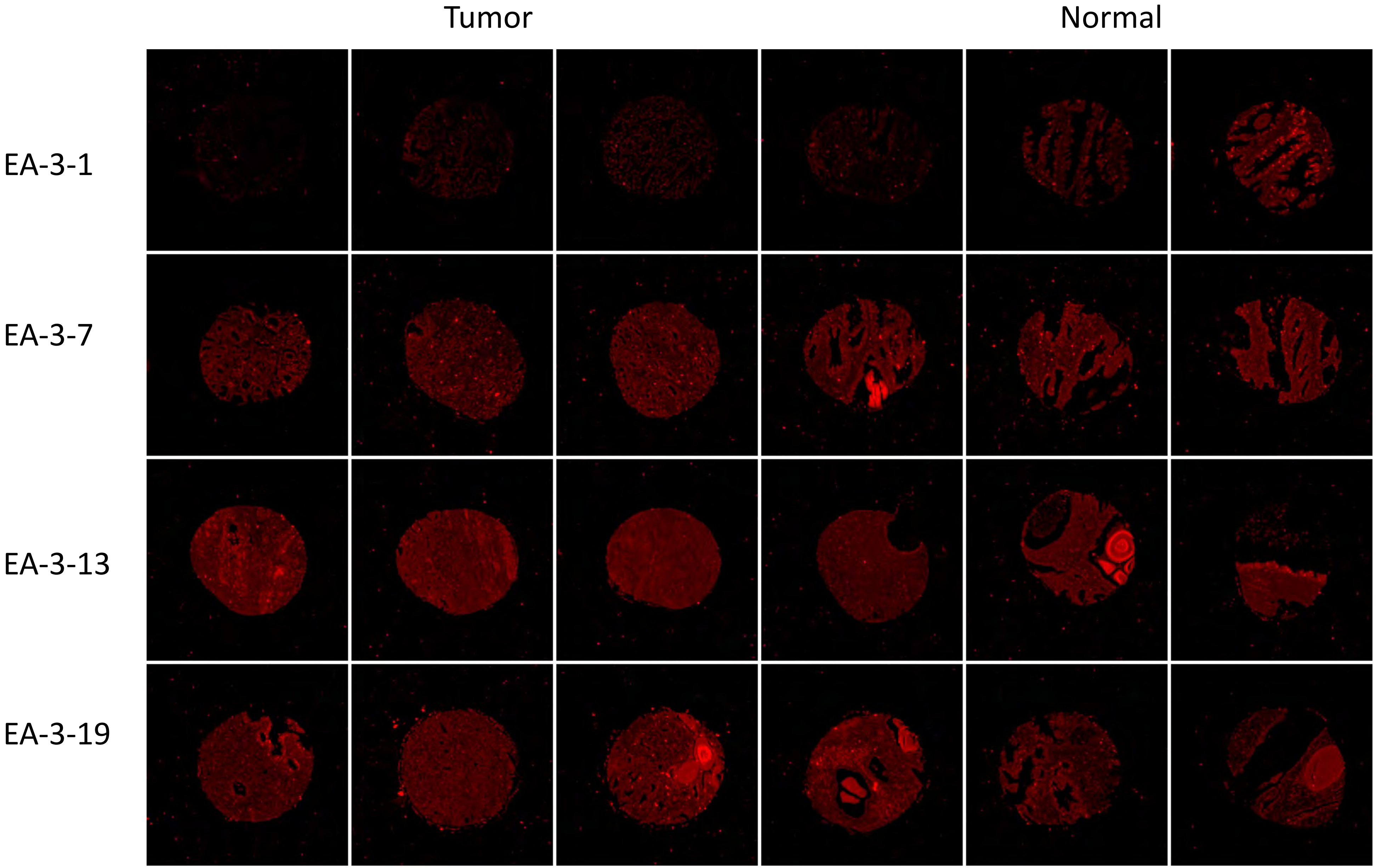

Row 3- UDG

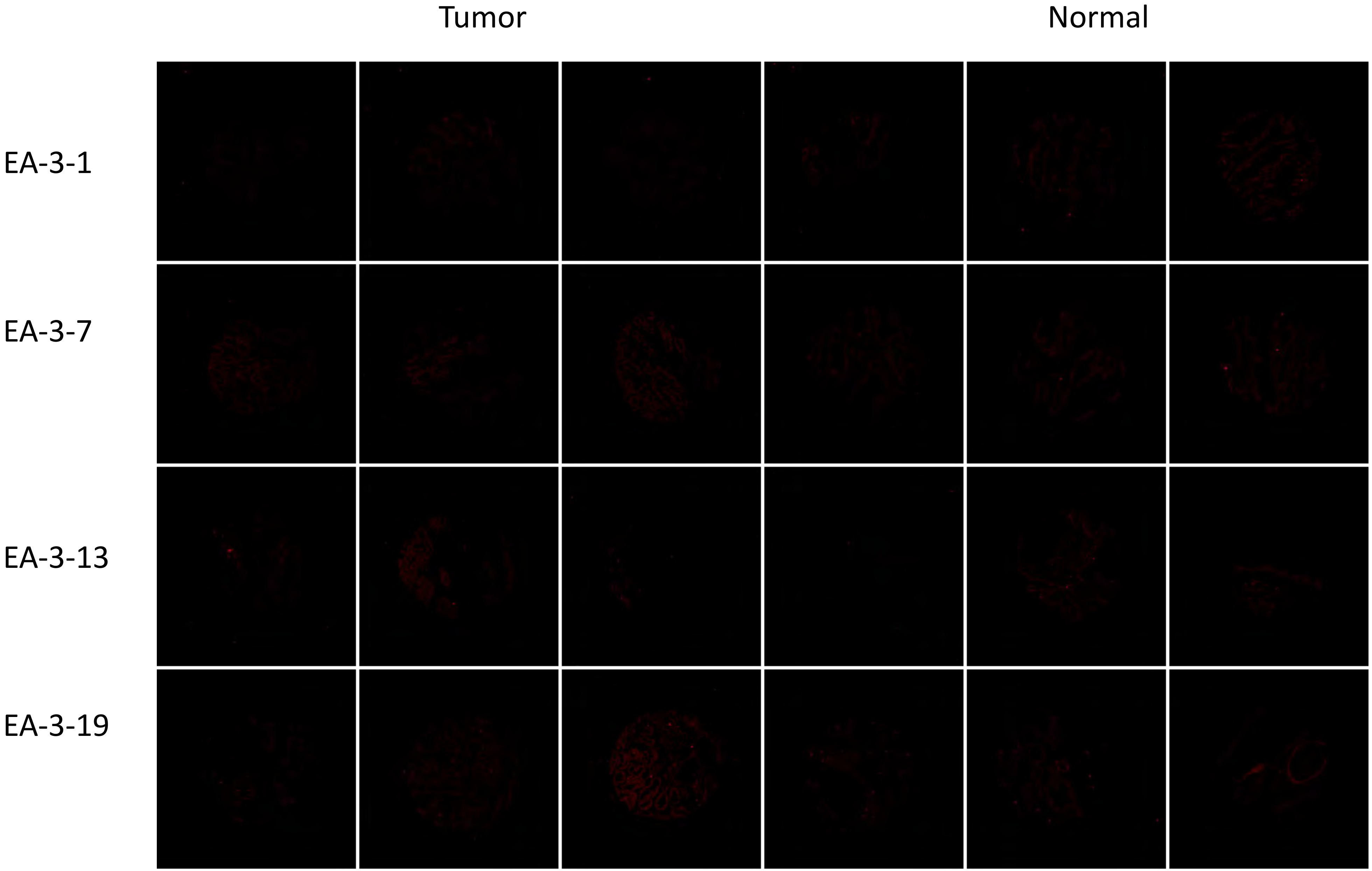

Row 3- T4PDG

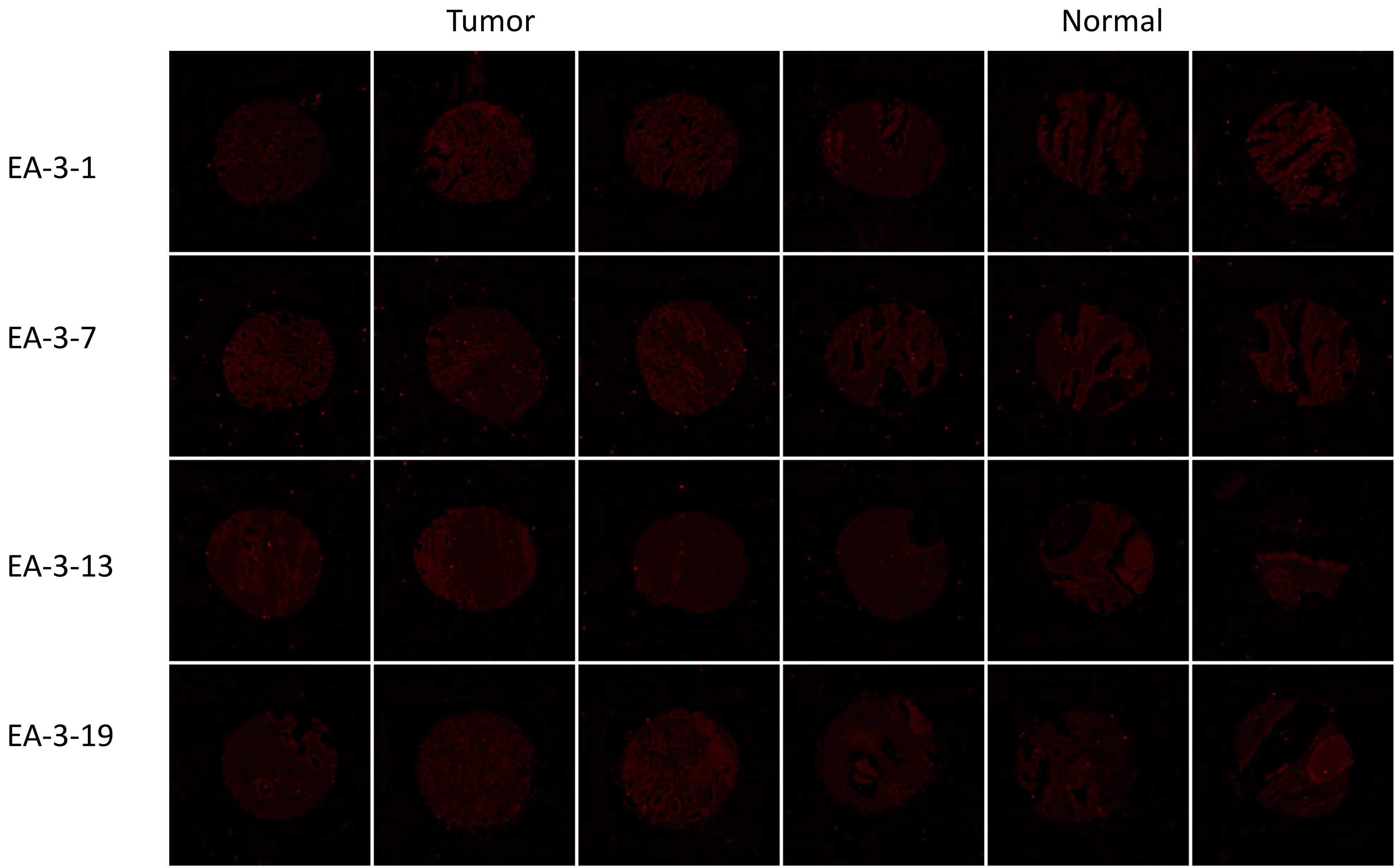

Row 3- XRCC1

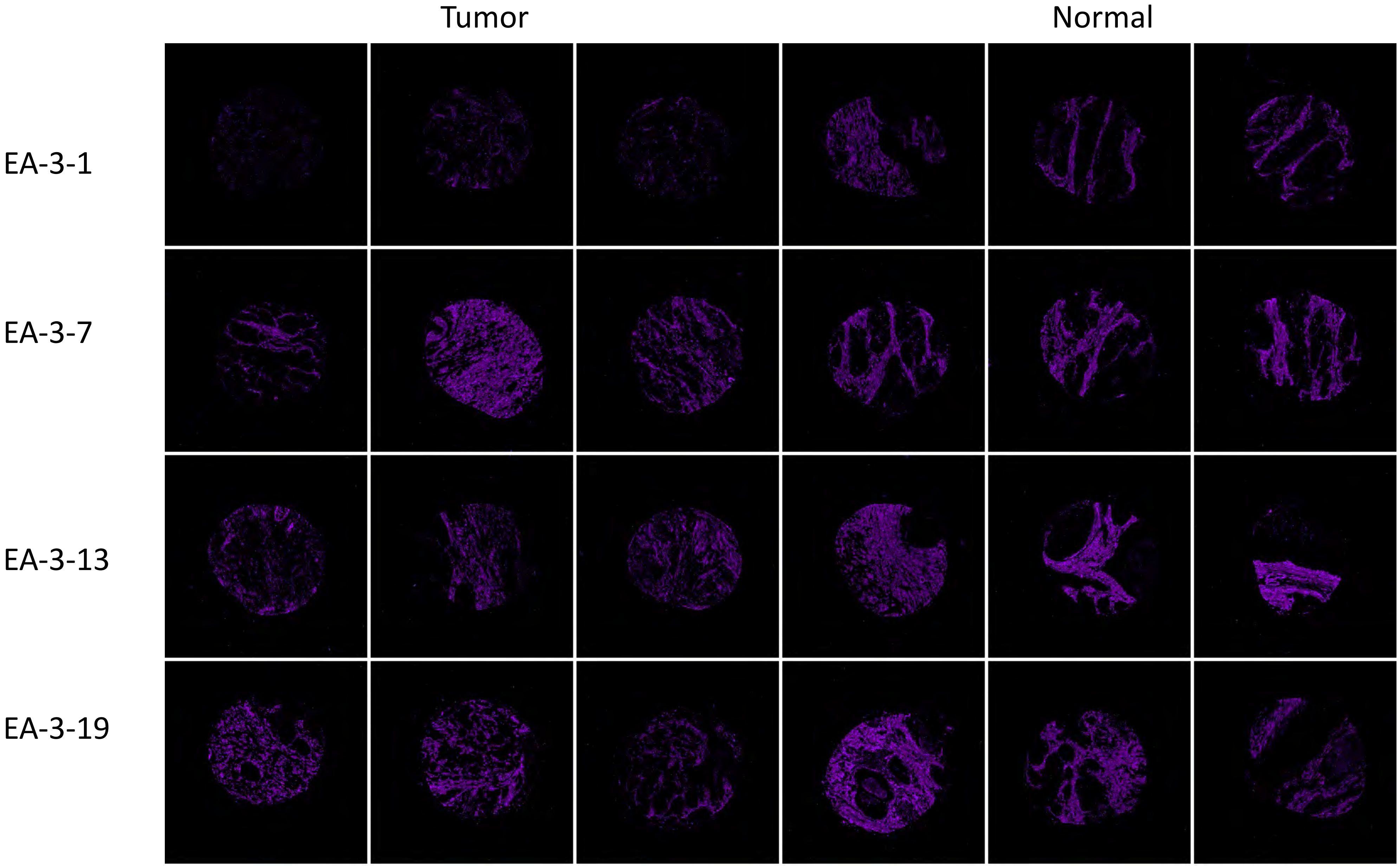

Row 3- PARP1

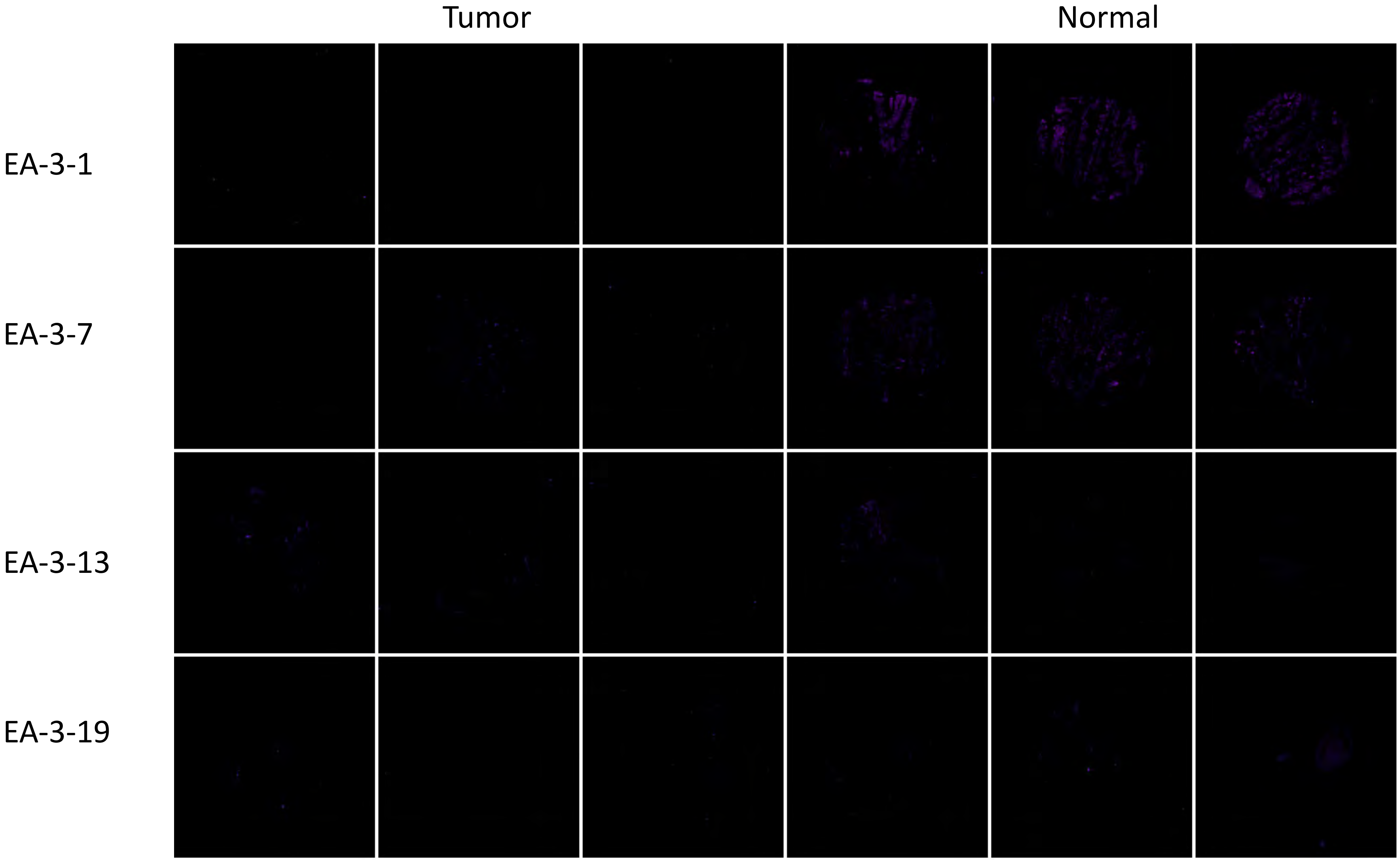

Row 3- UNG

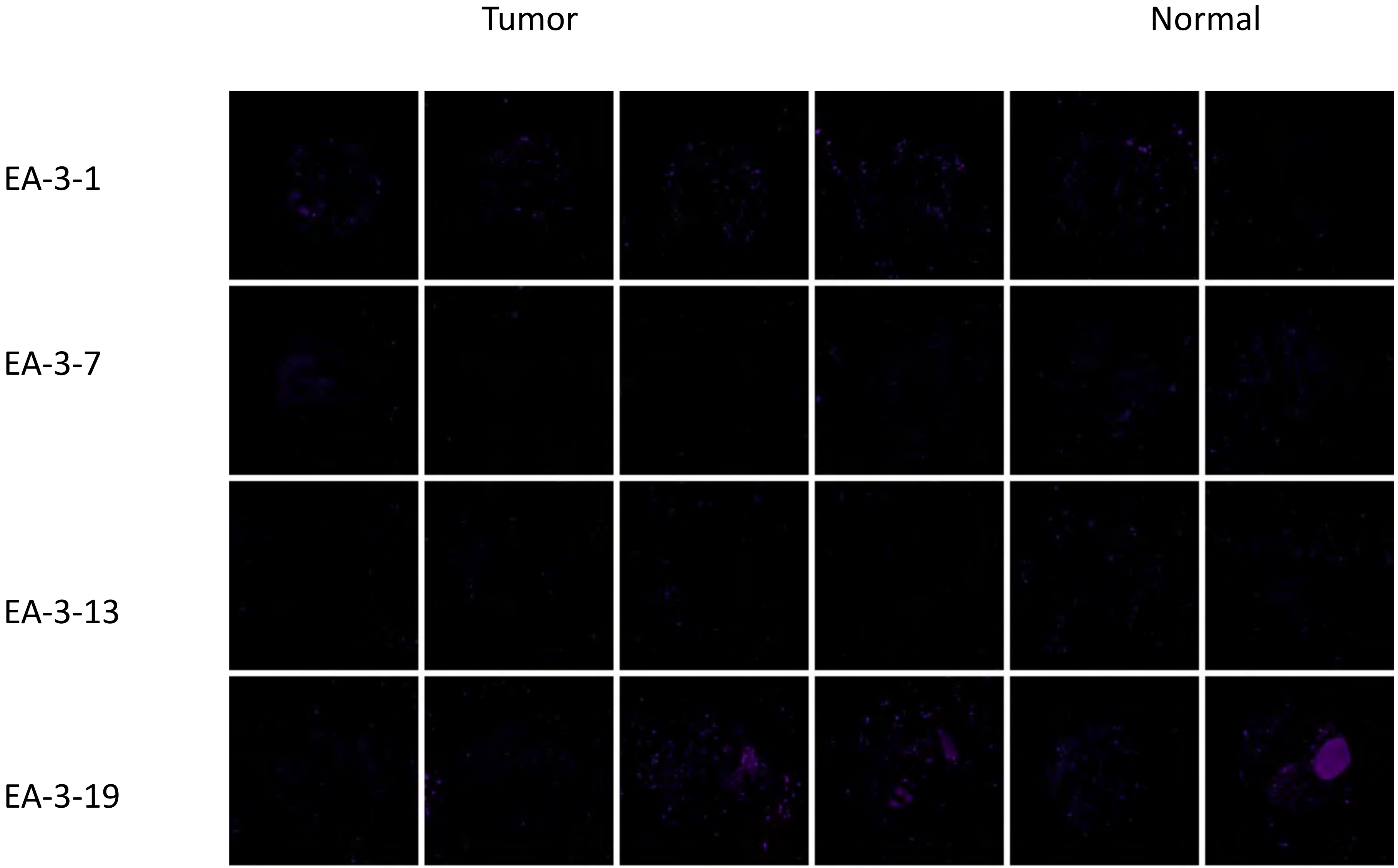

Row 4- Full RADD

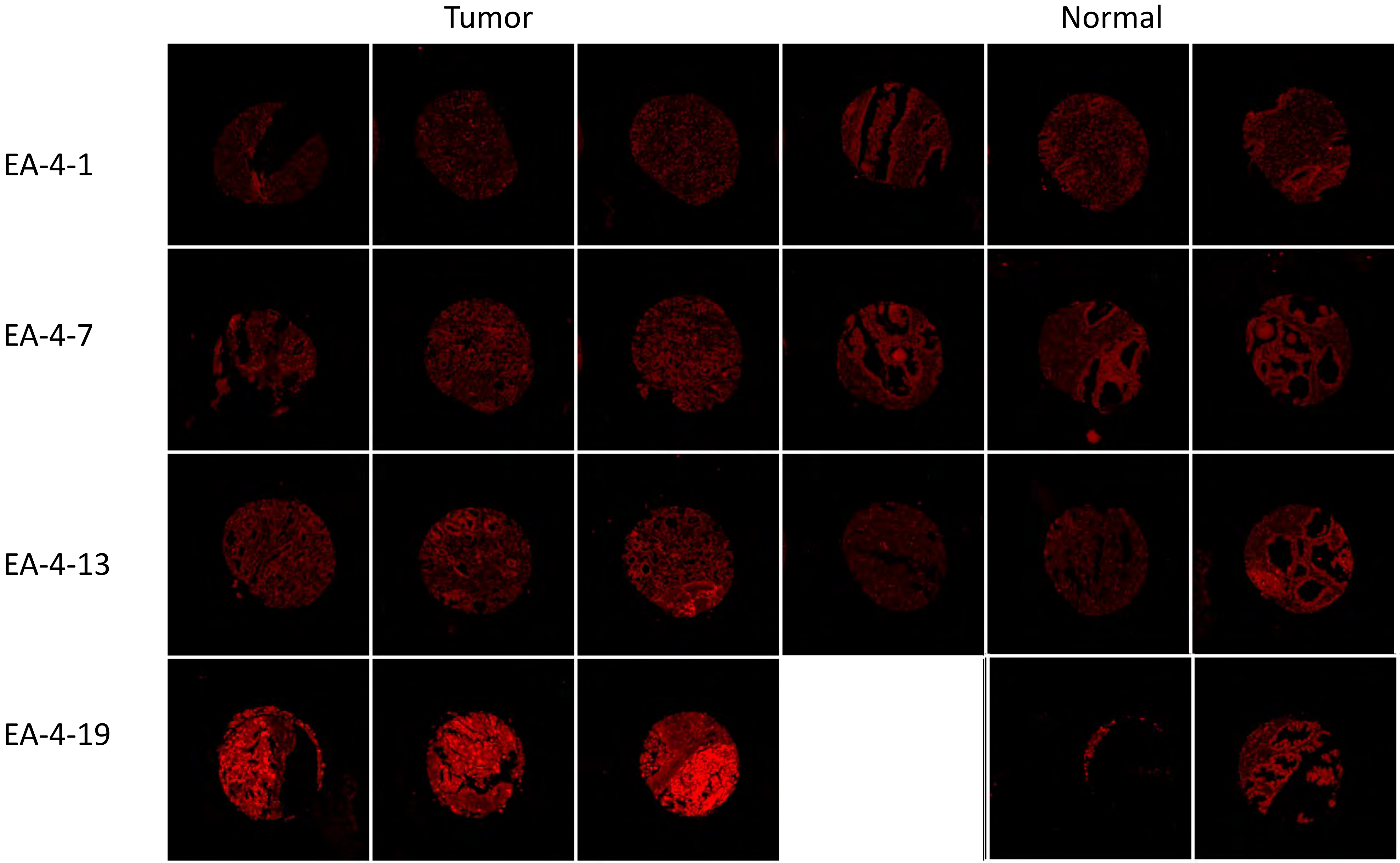

Row 4- oxRADD

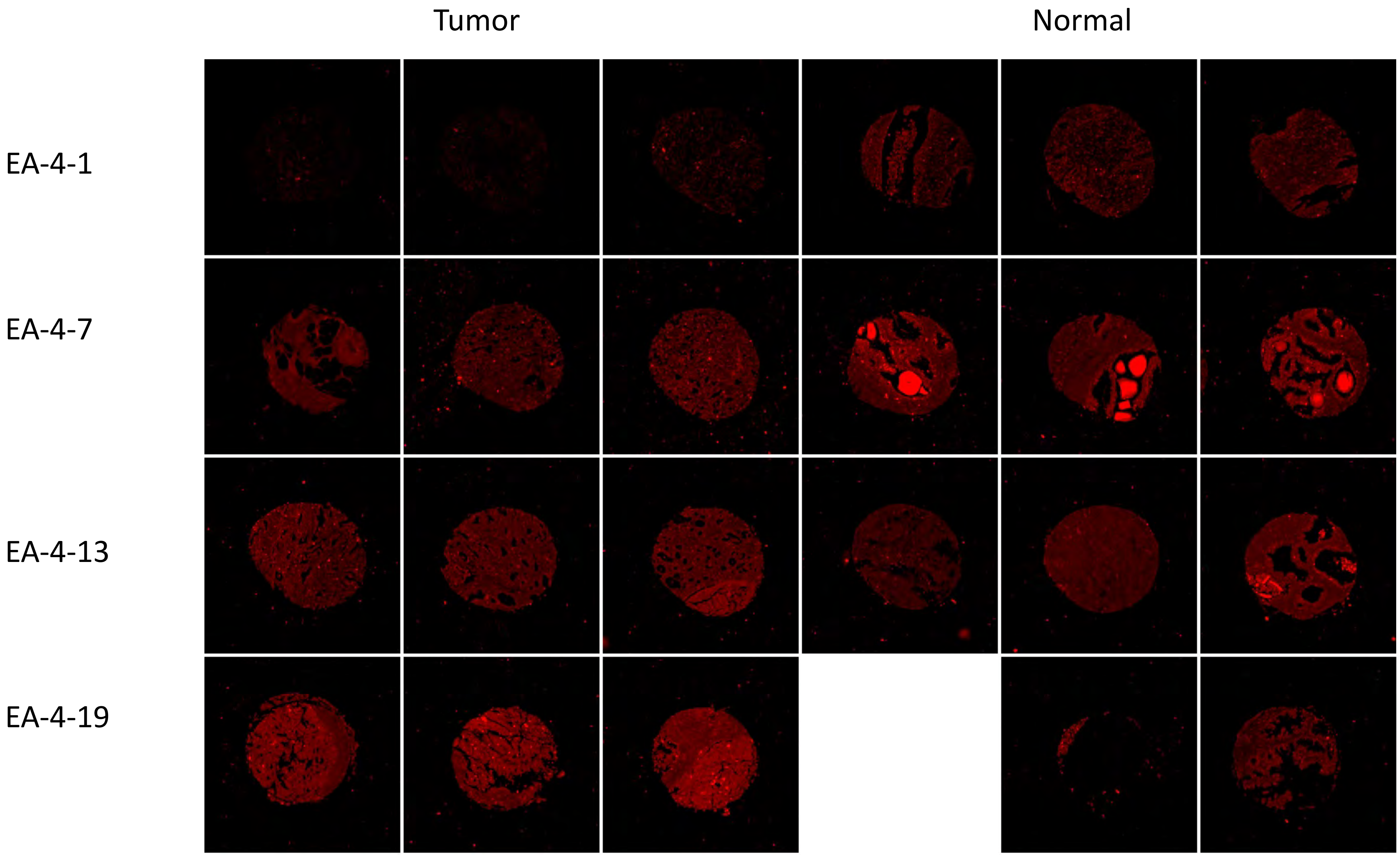

Row 4- UDG

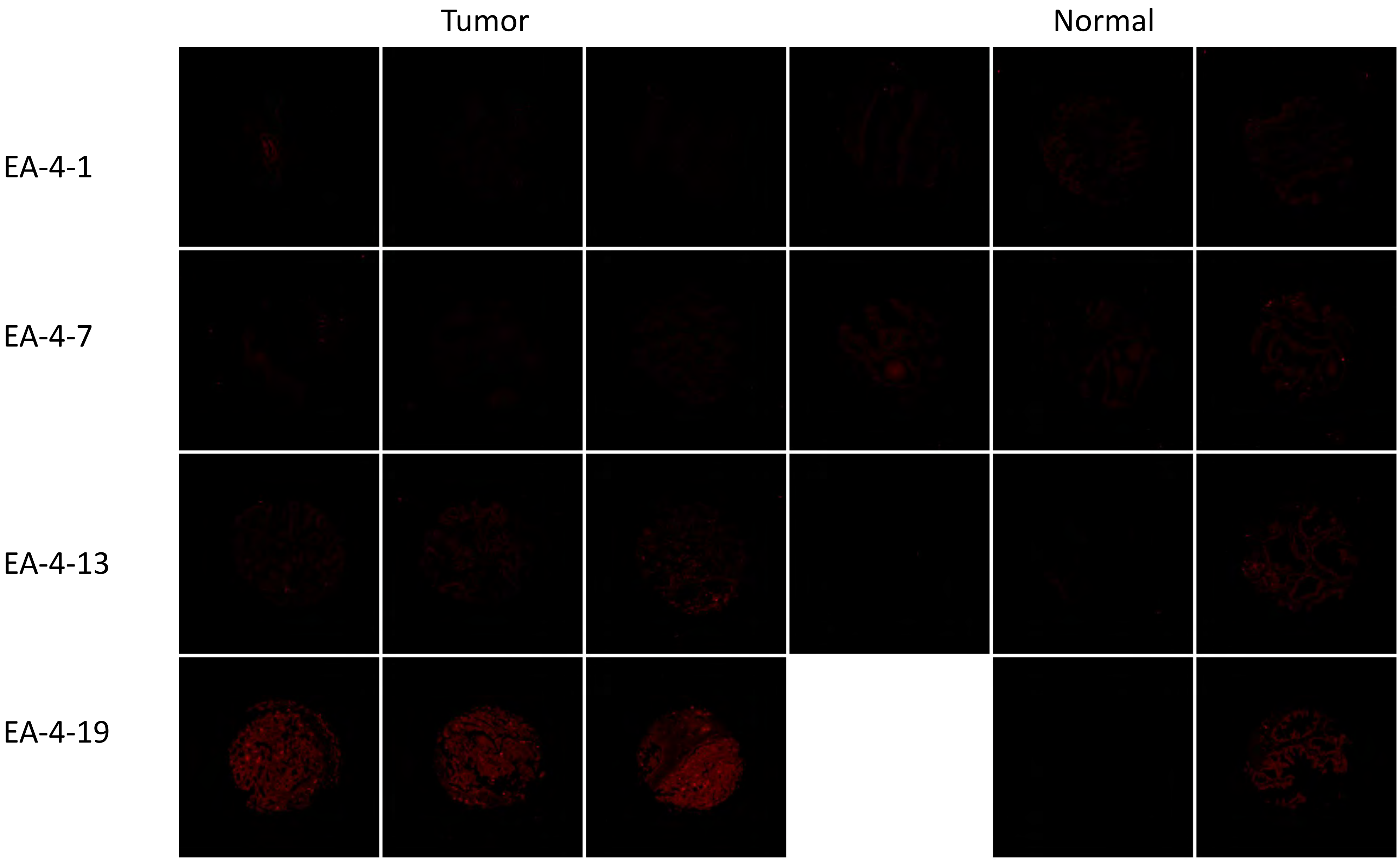

Row 4- T4PDG

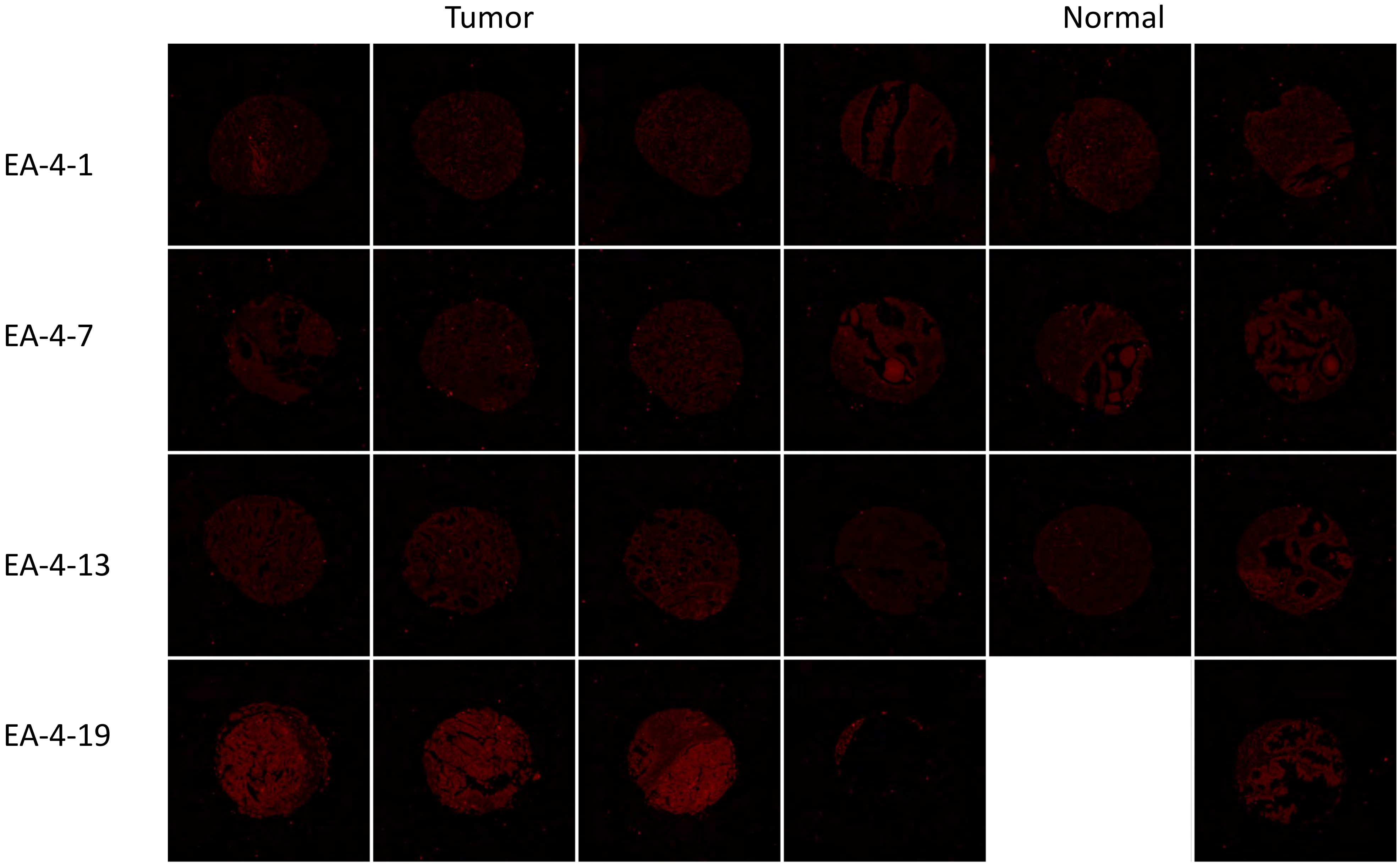

Row 4- XRCC1

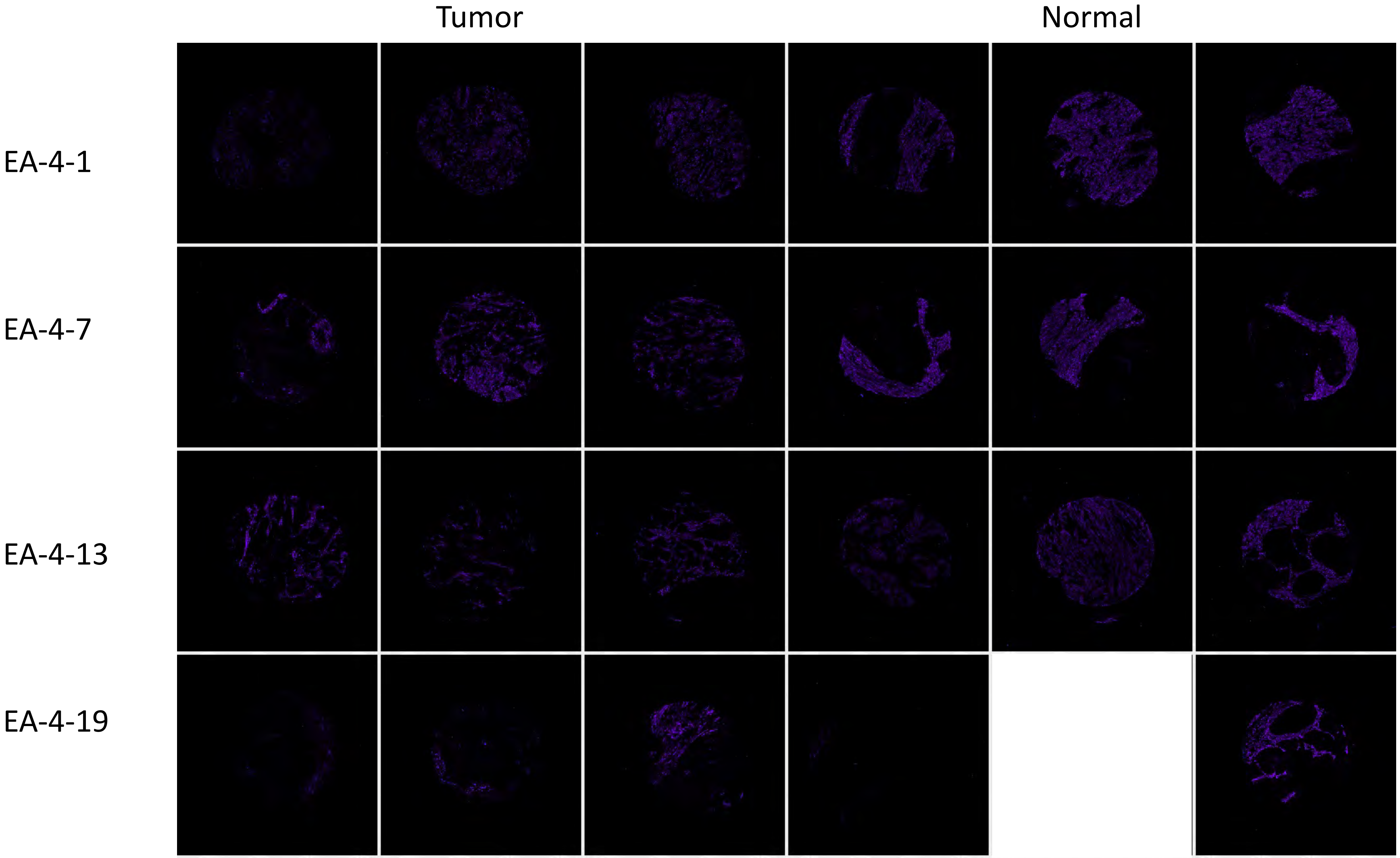

Row 4- PARP1

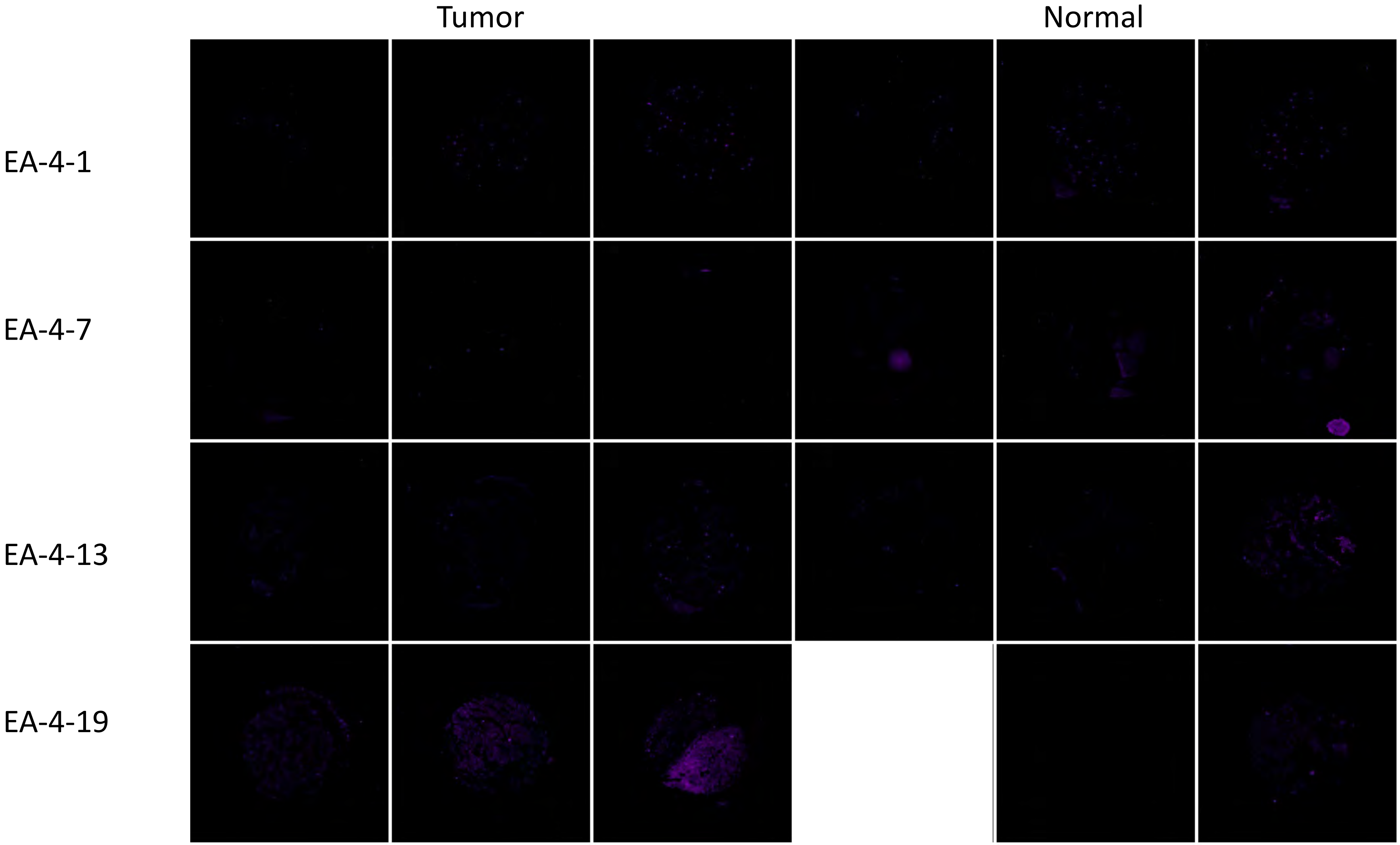

Row 4- UNG

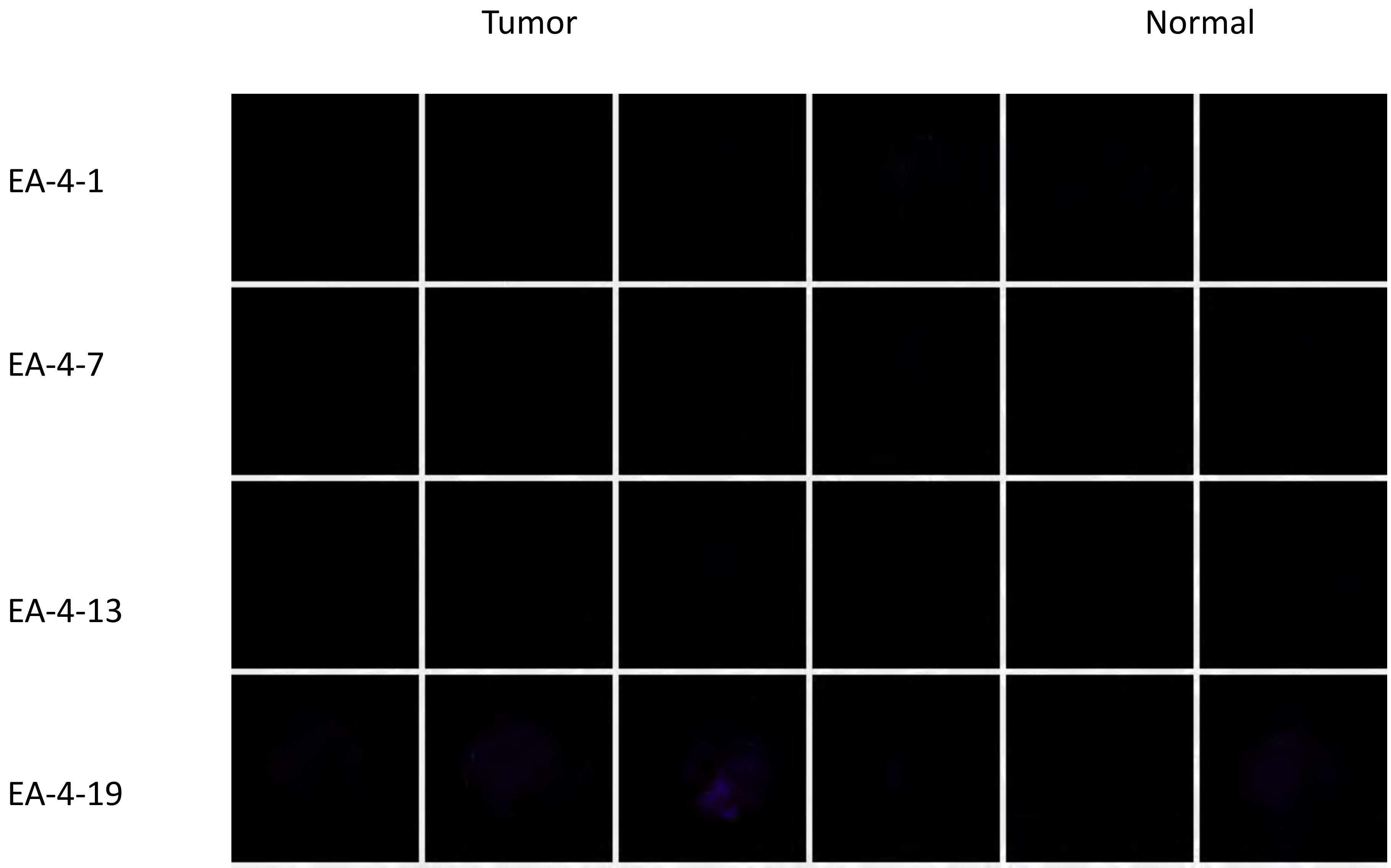

Row 5- Full RADD

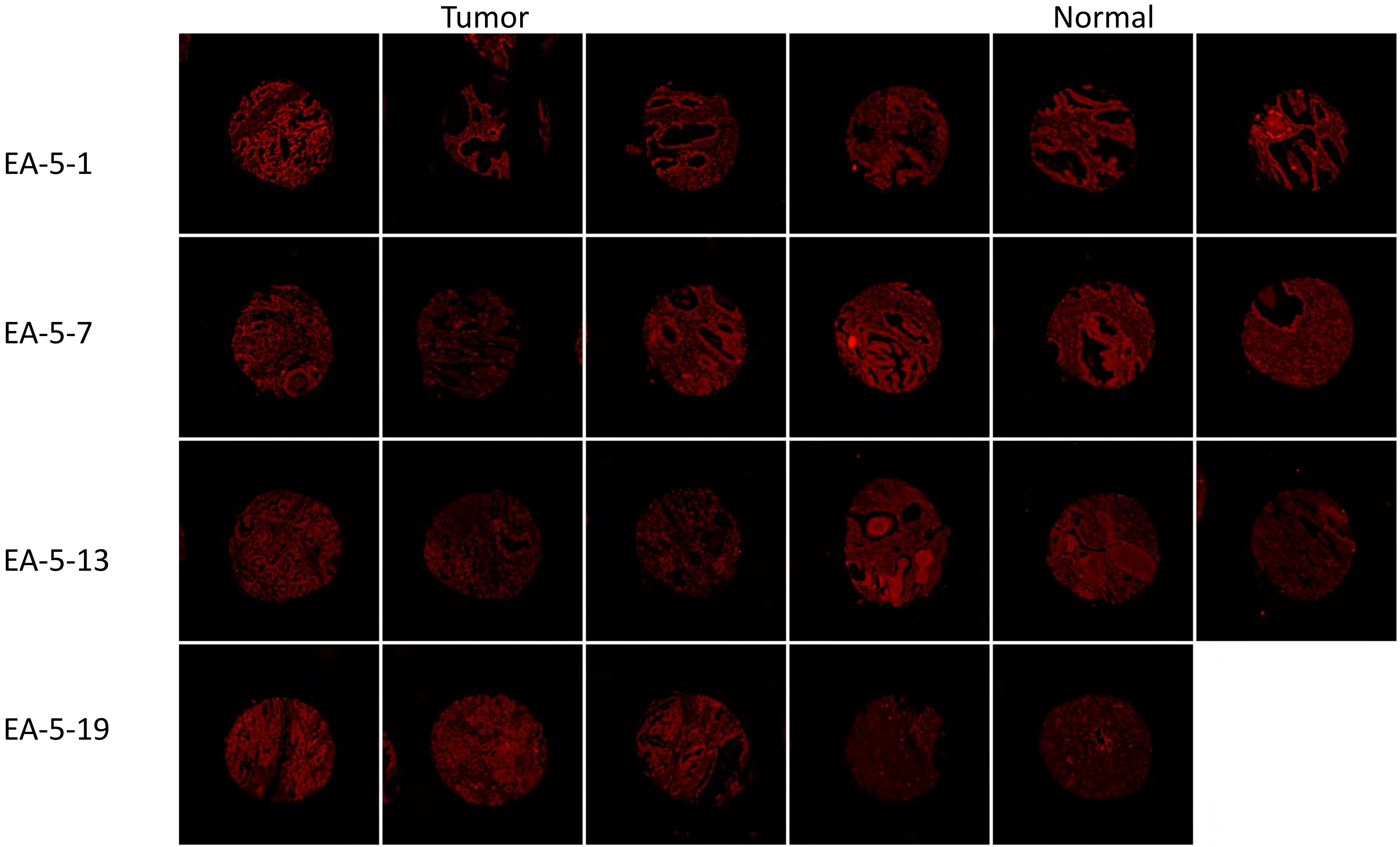

Row 5- oxRADD

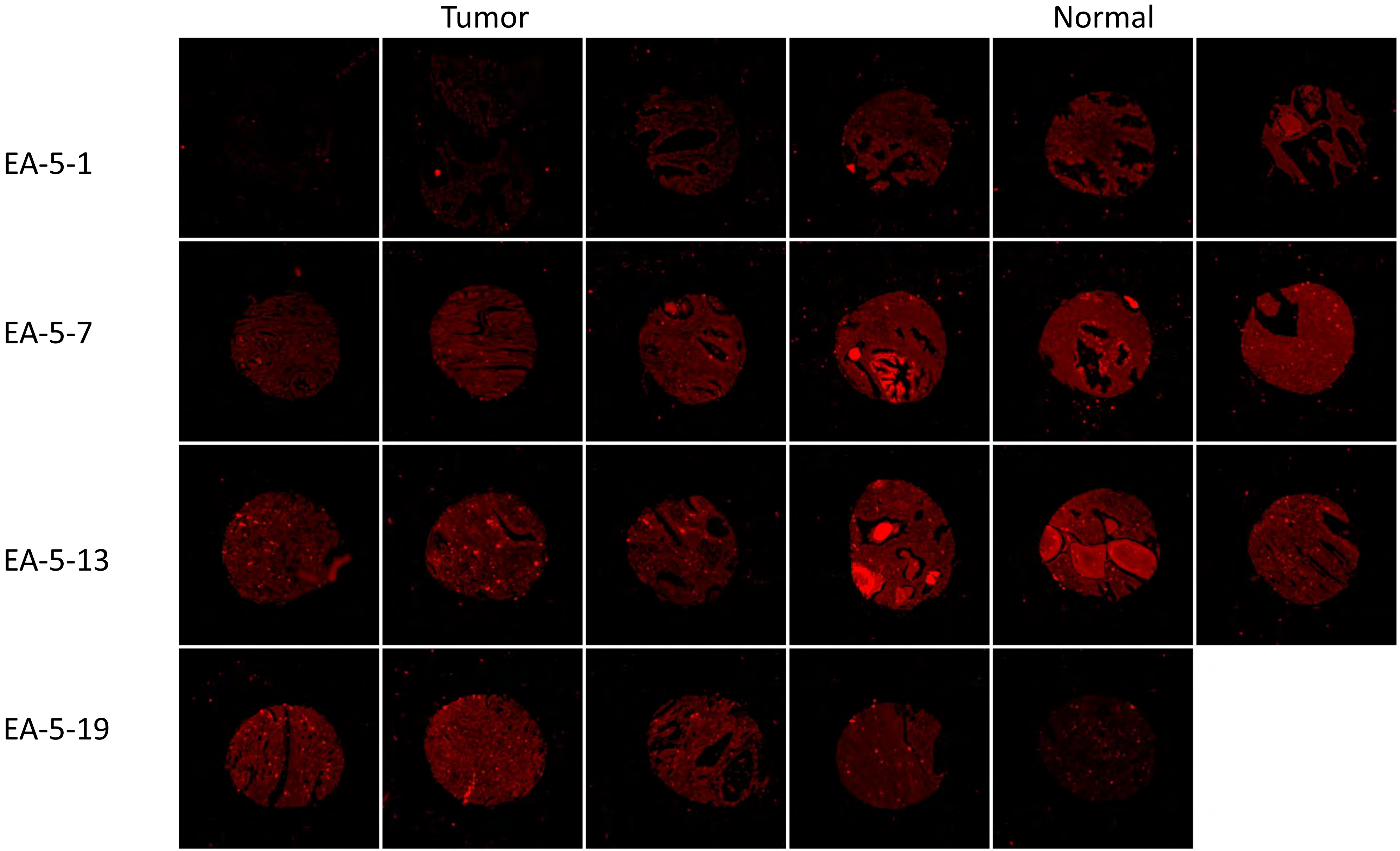

Row 5- UDG

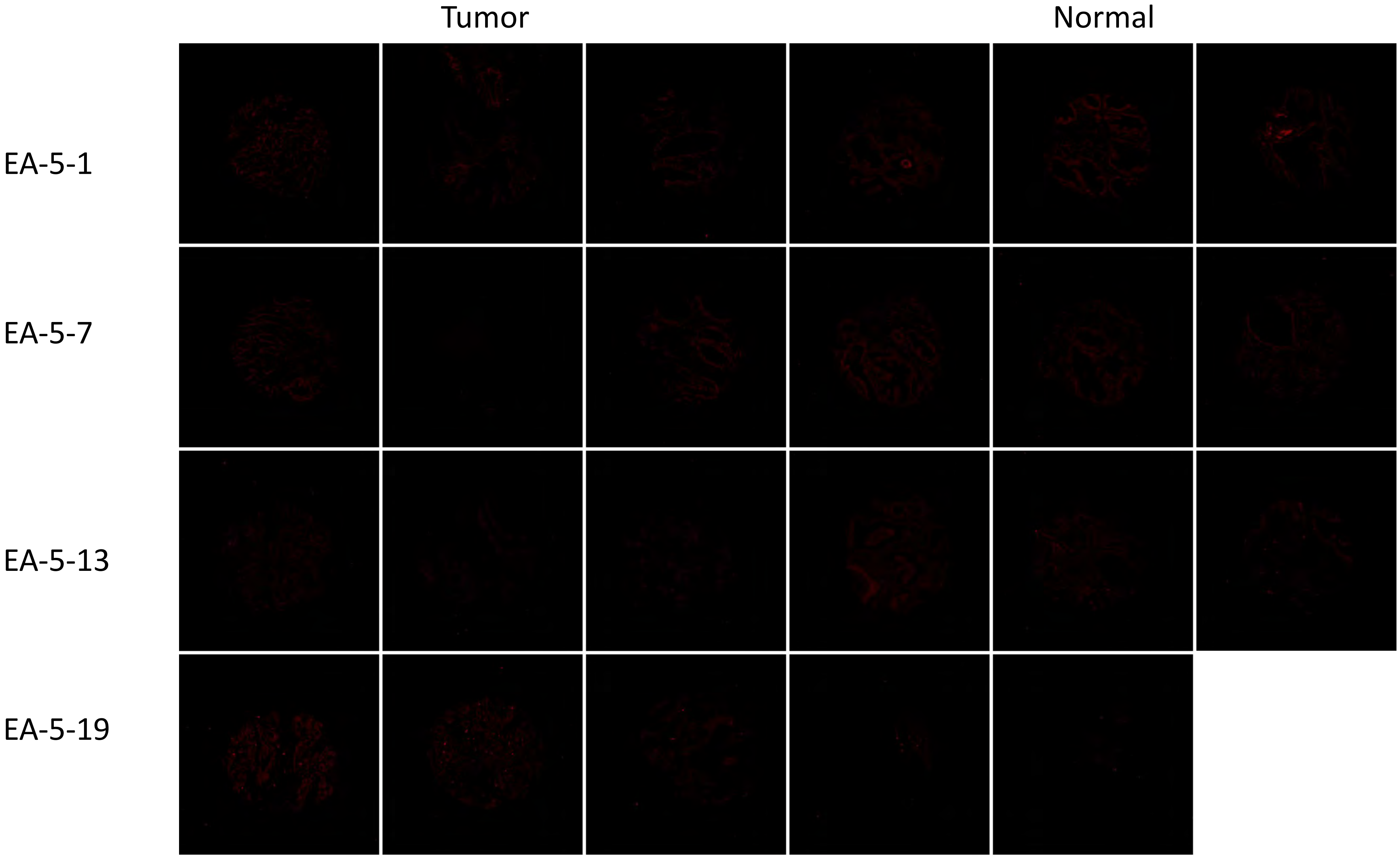

Row 5- T4PDG

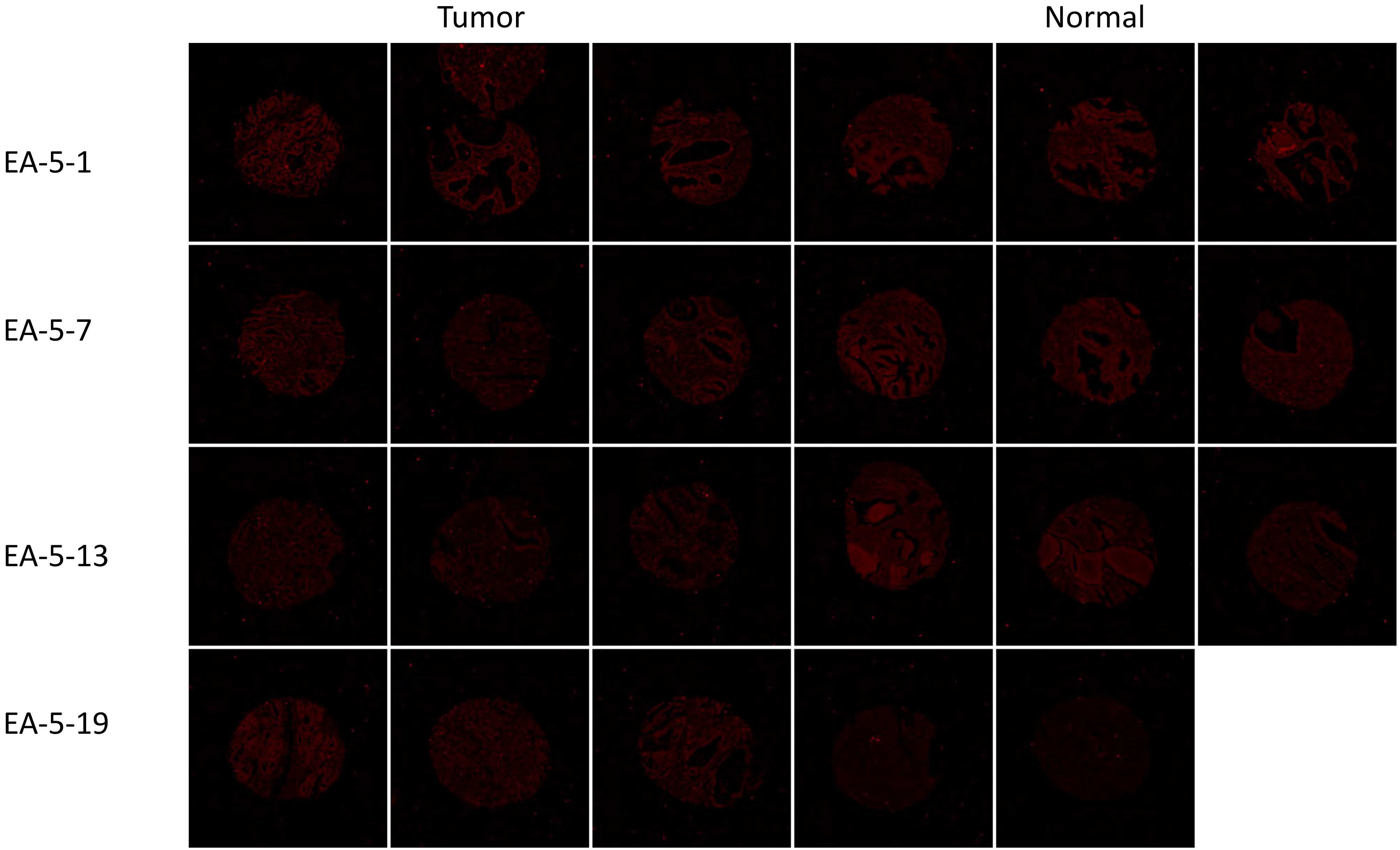

Row 5- XRCC1

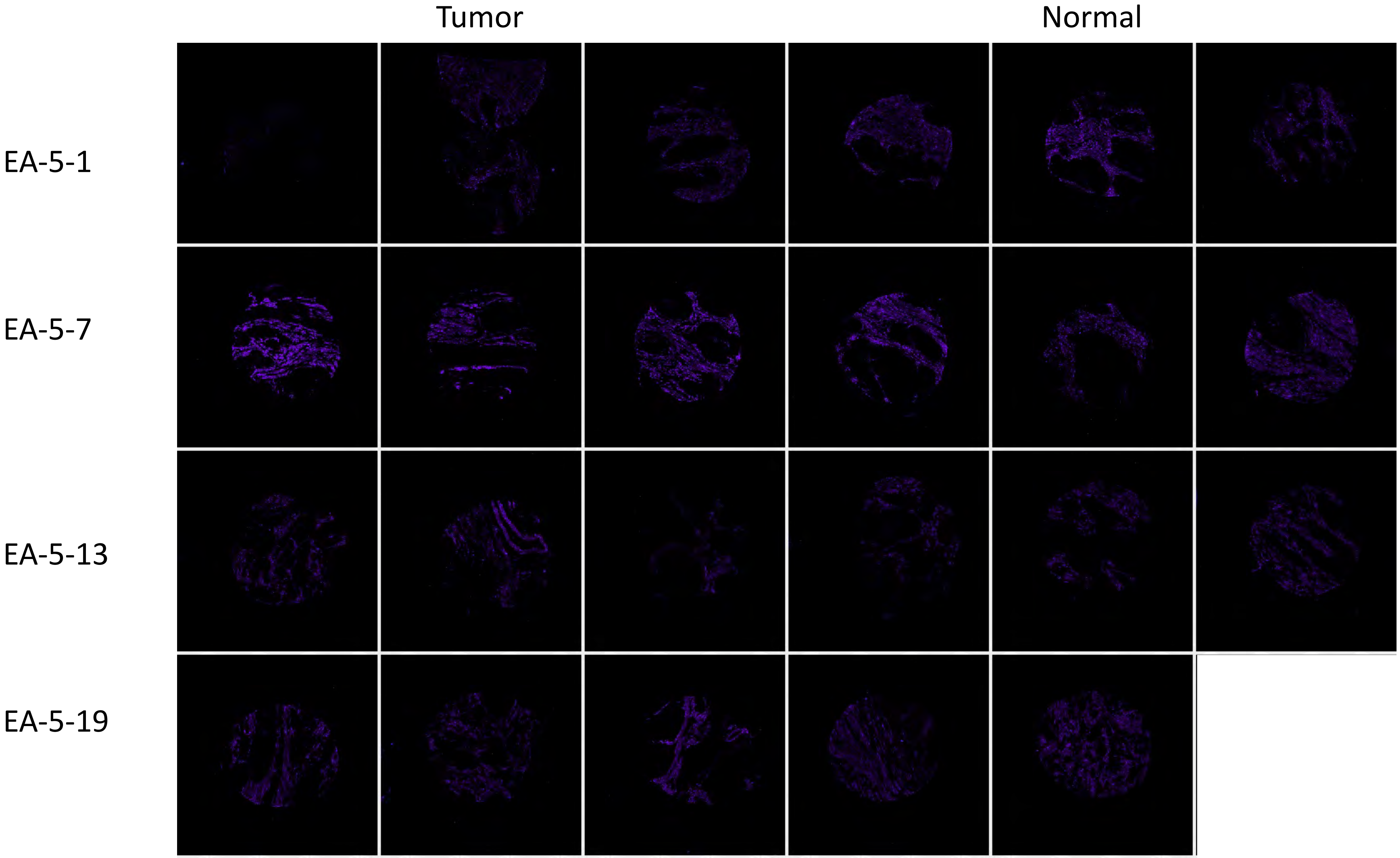

Row 5- PARP1

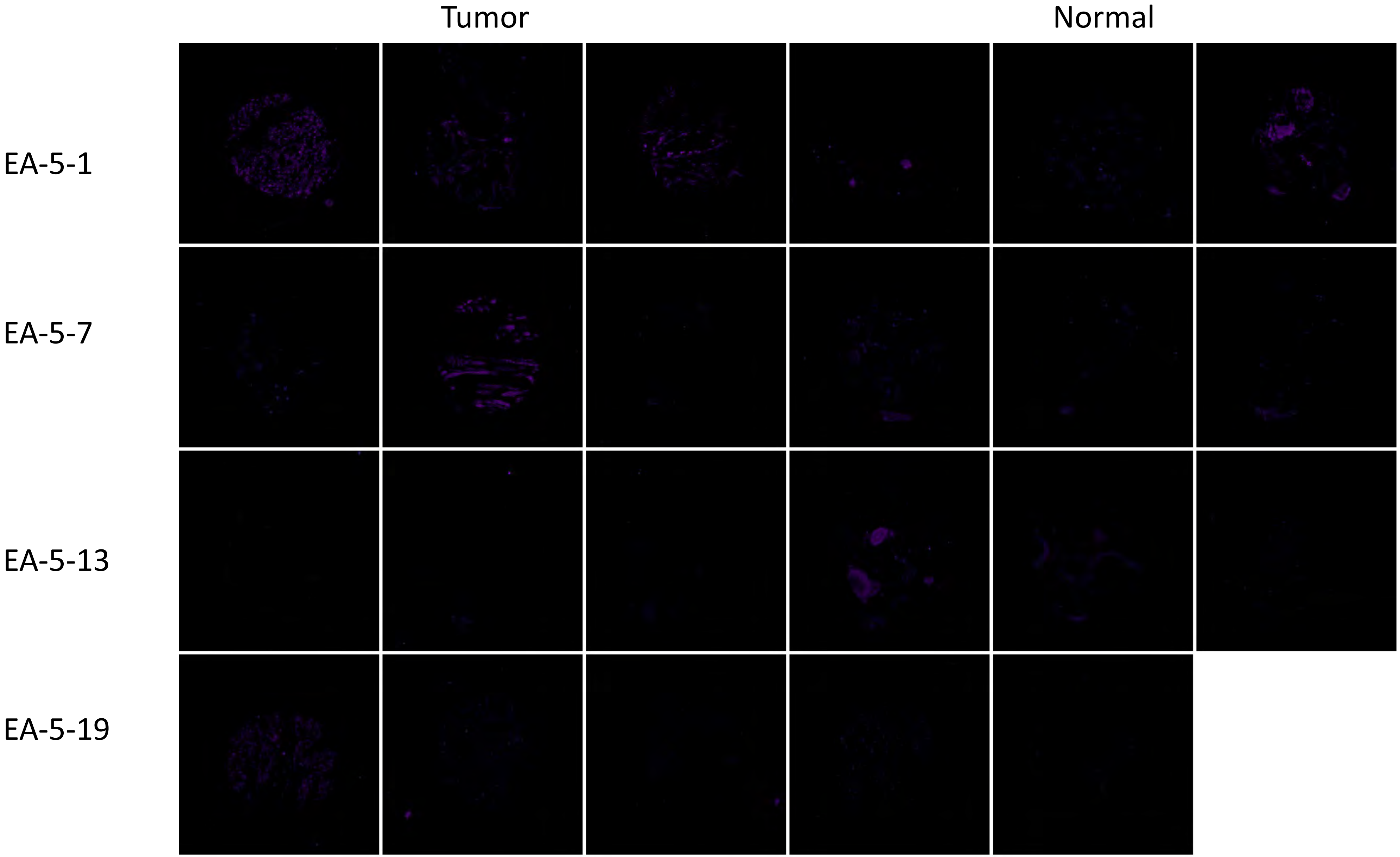

Row 5- UNG

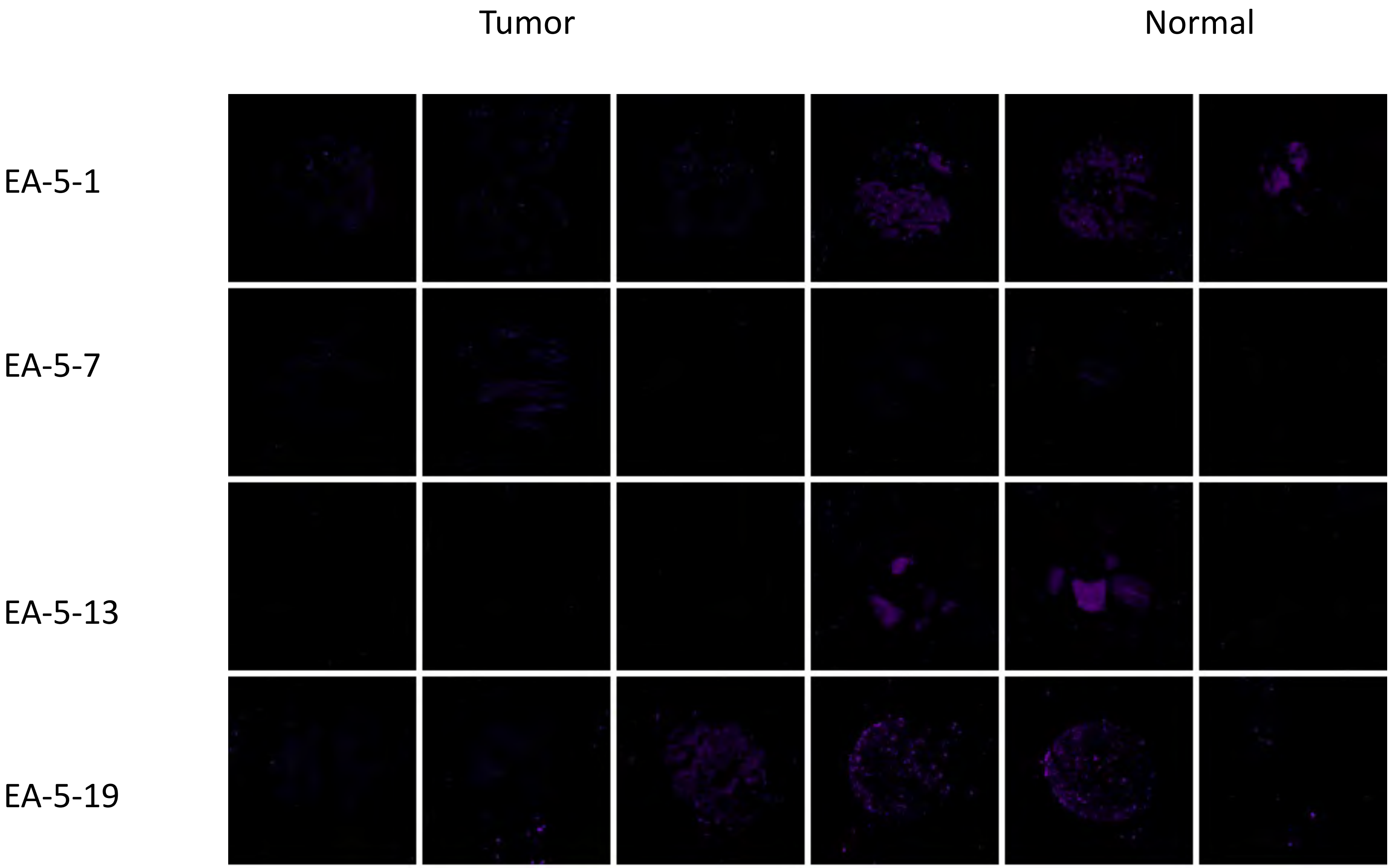

Row 6- Full RADD

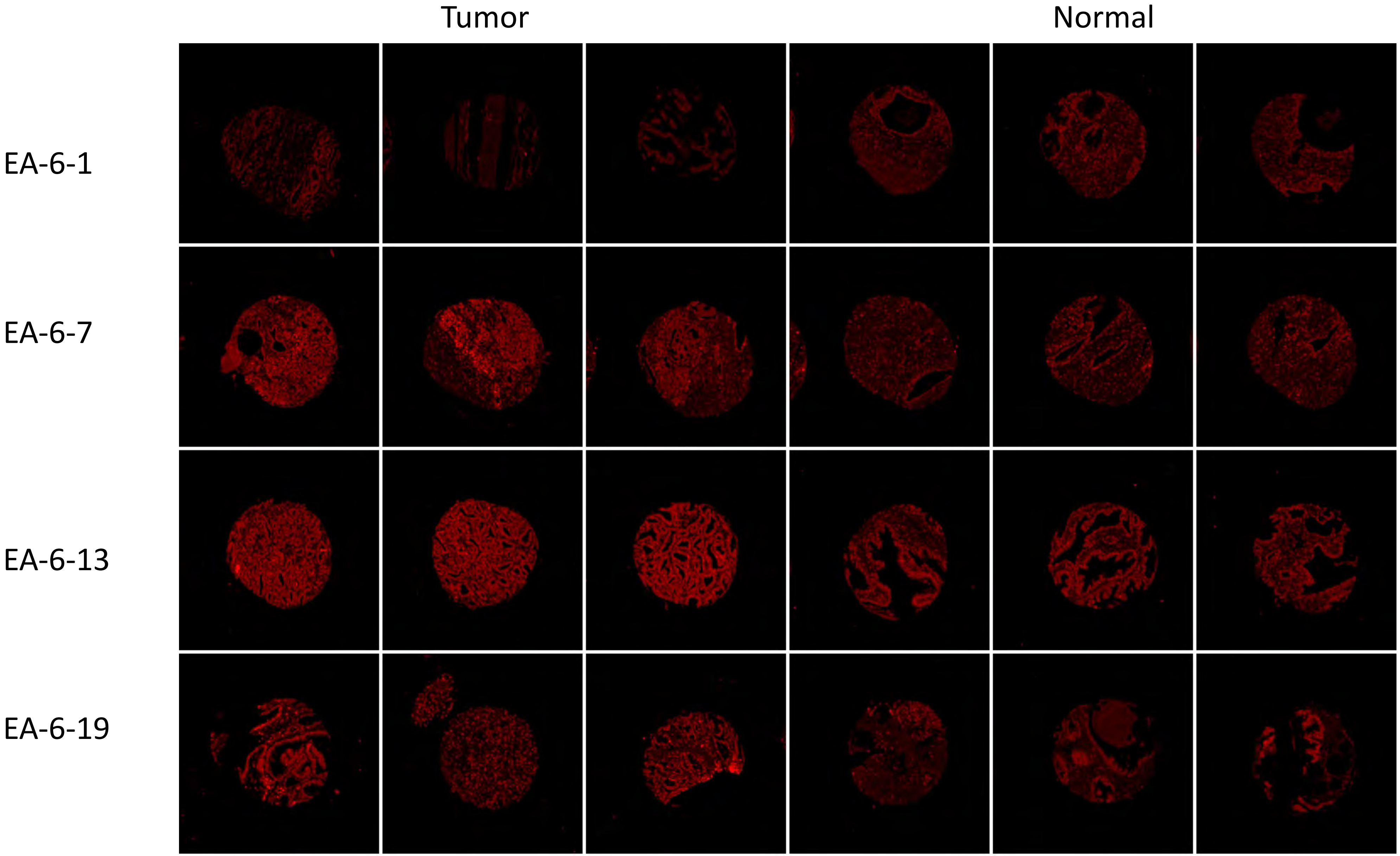

Row 6- oxRADD

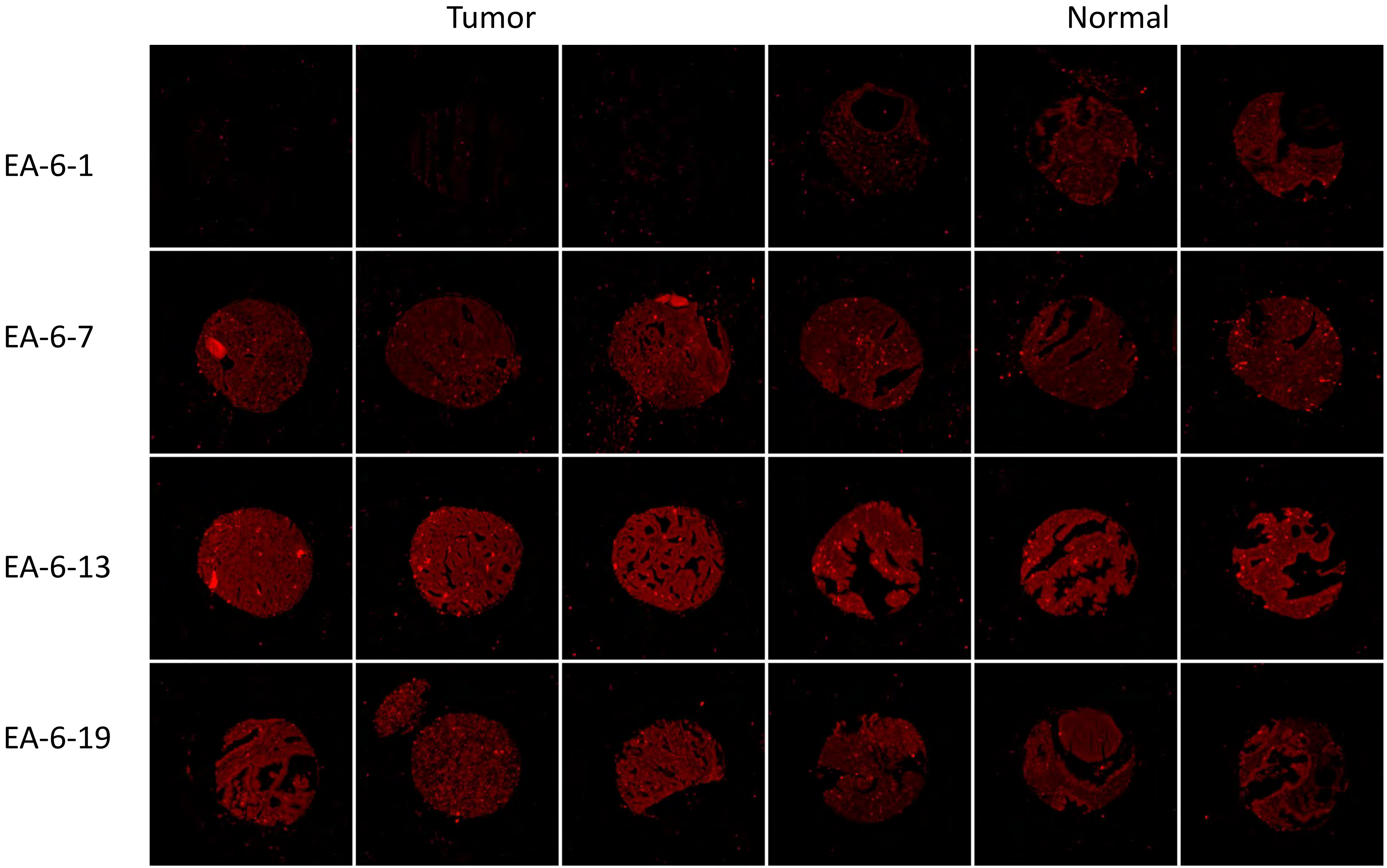

Row 6- UDG

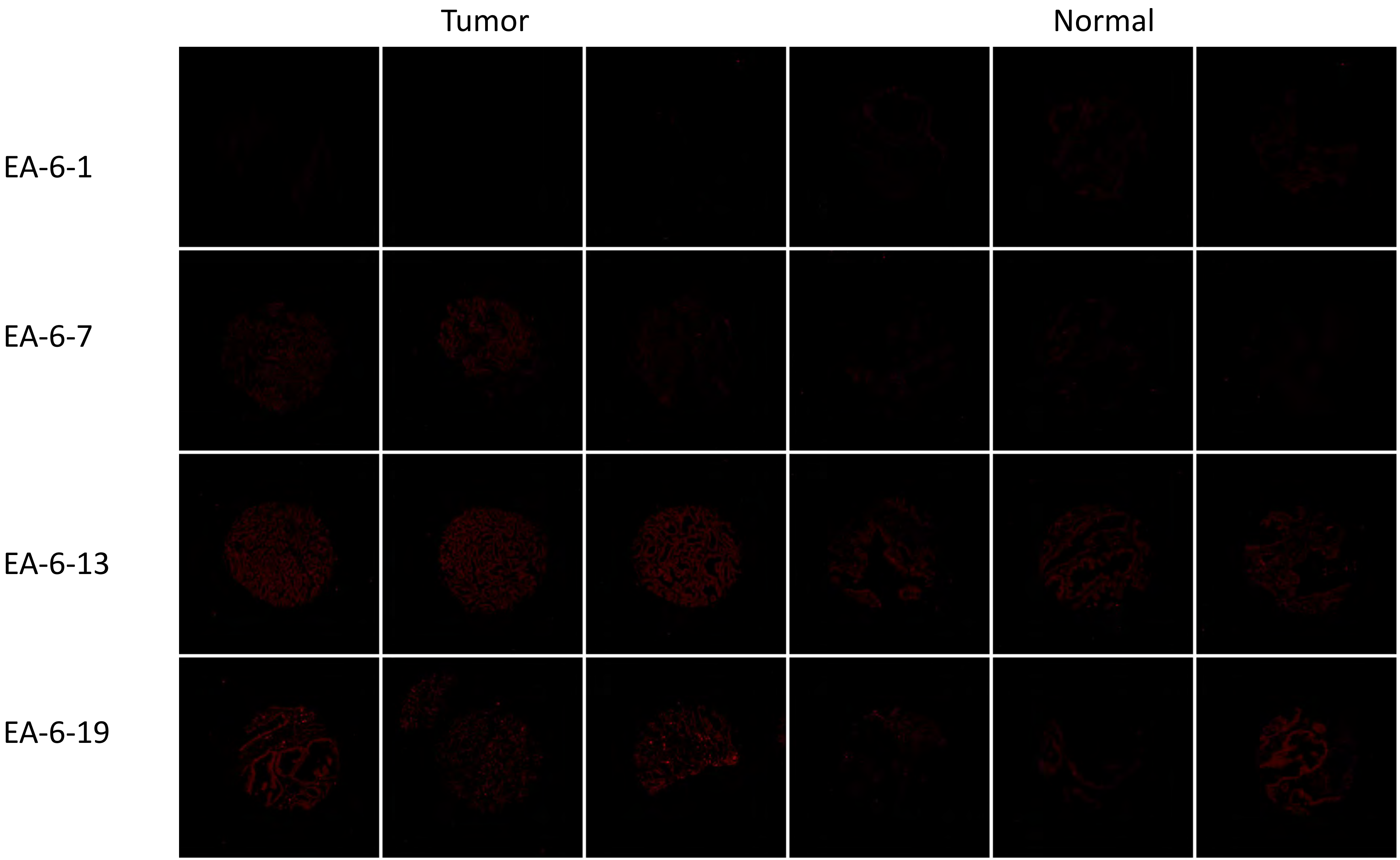

Row 6- T4PDG

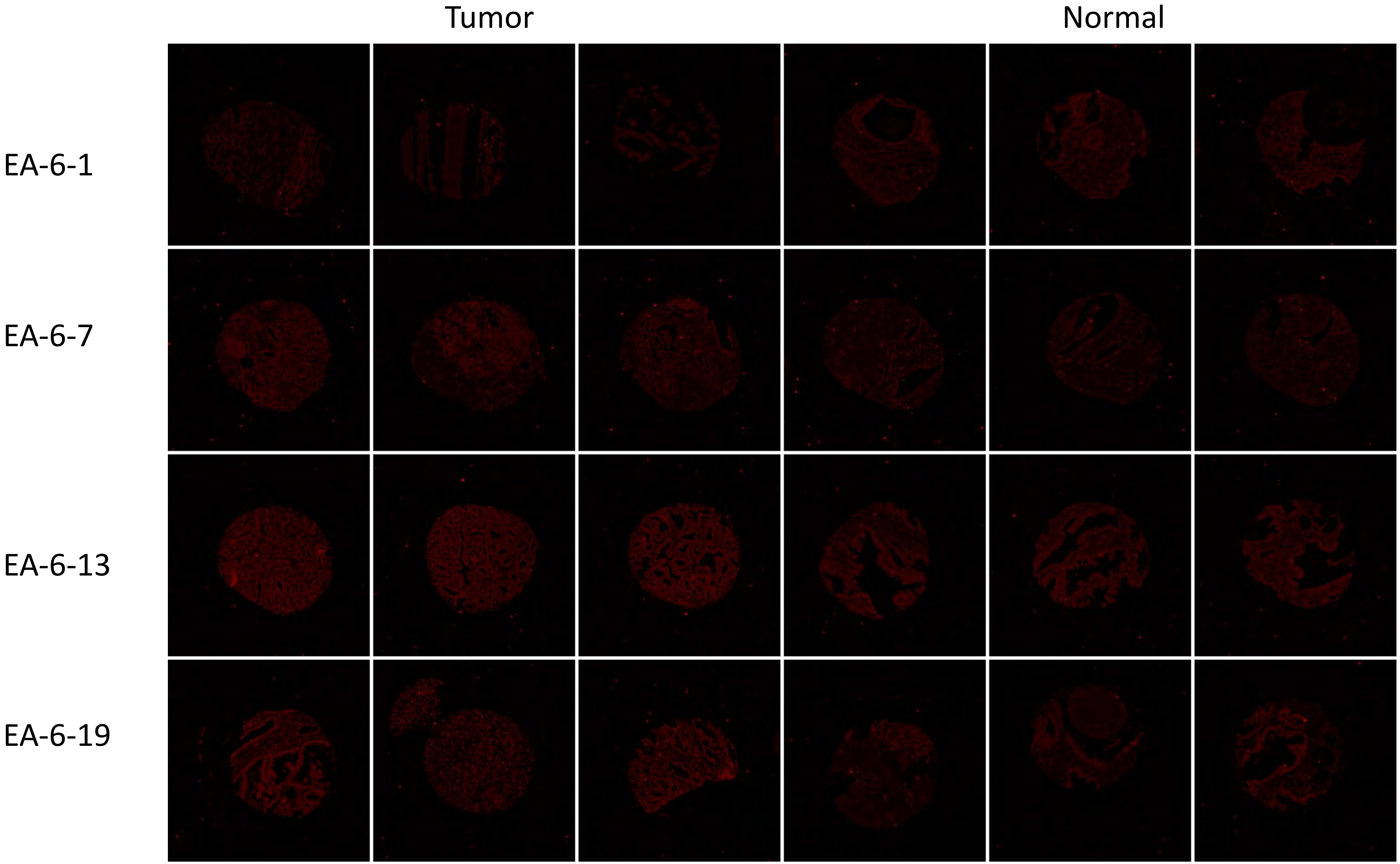

Row 6- XRCC1

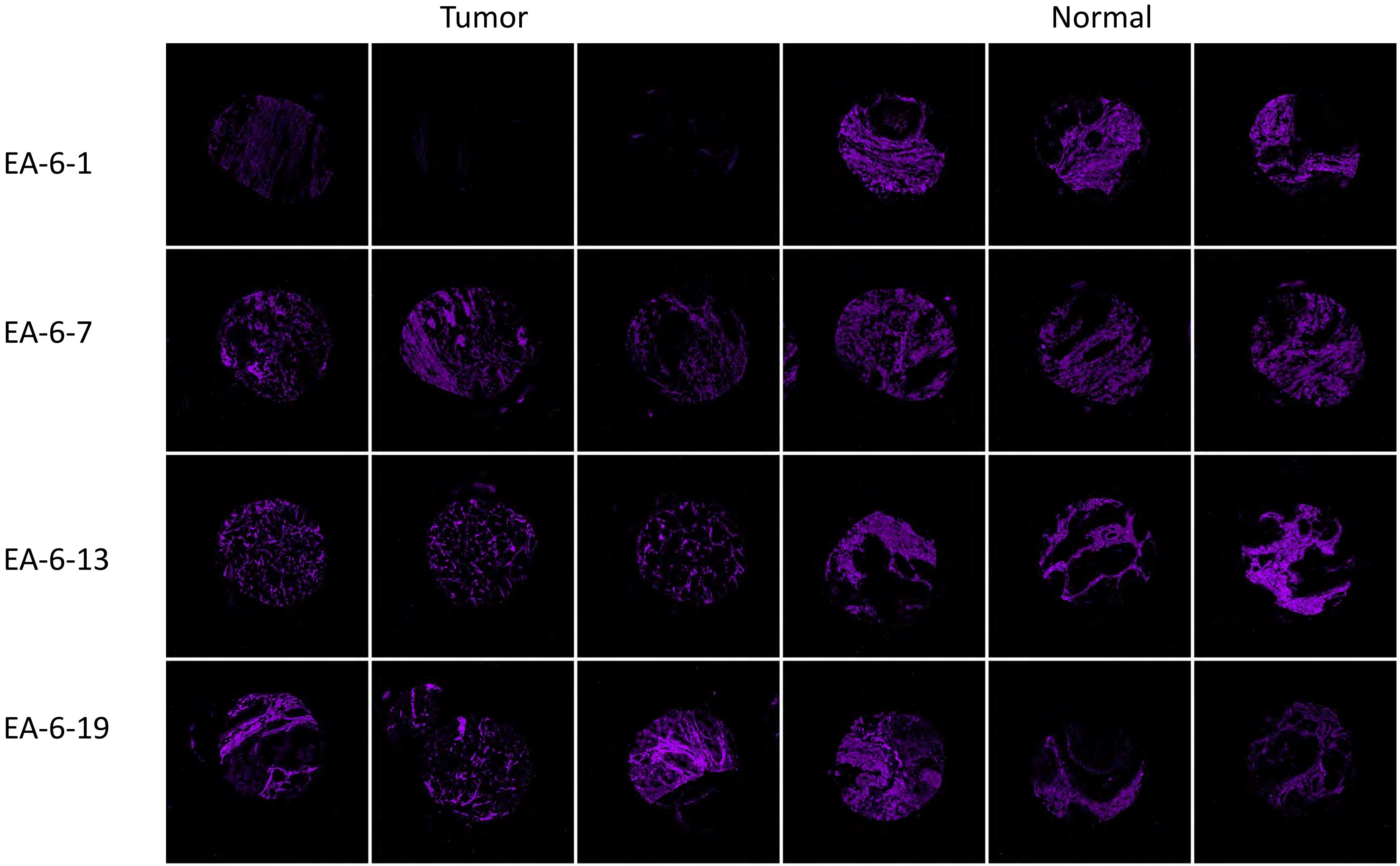

Row 6- PARP1

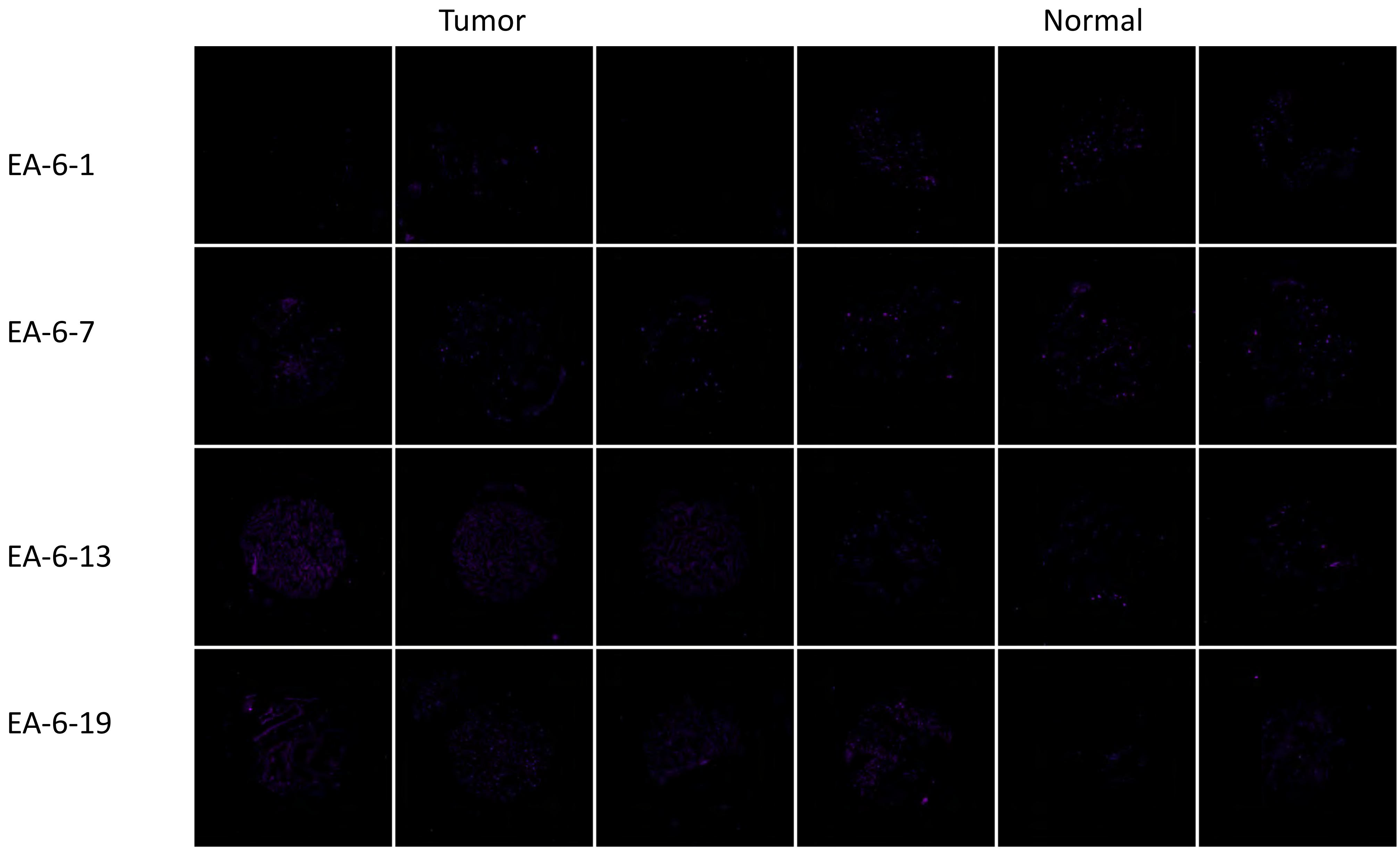

Row 6- UNG

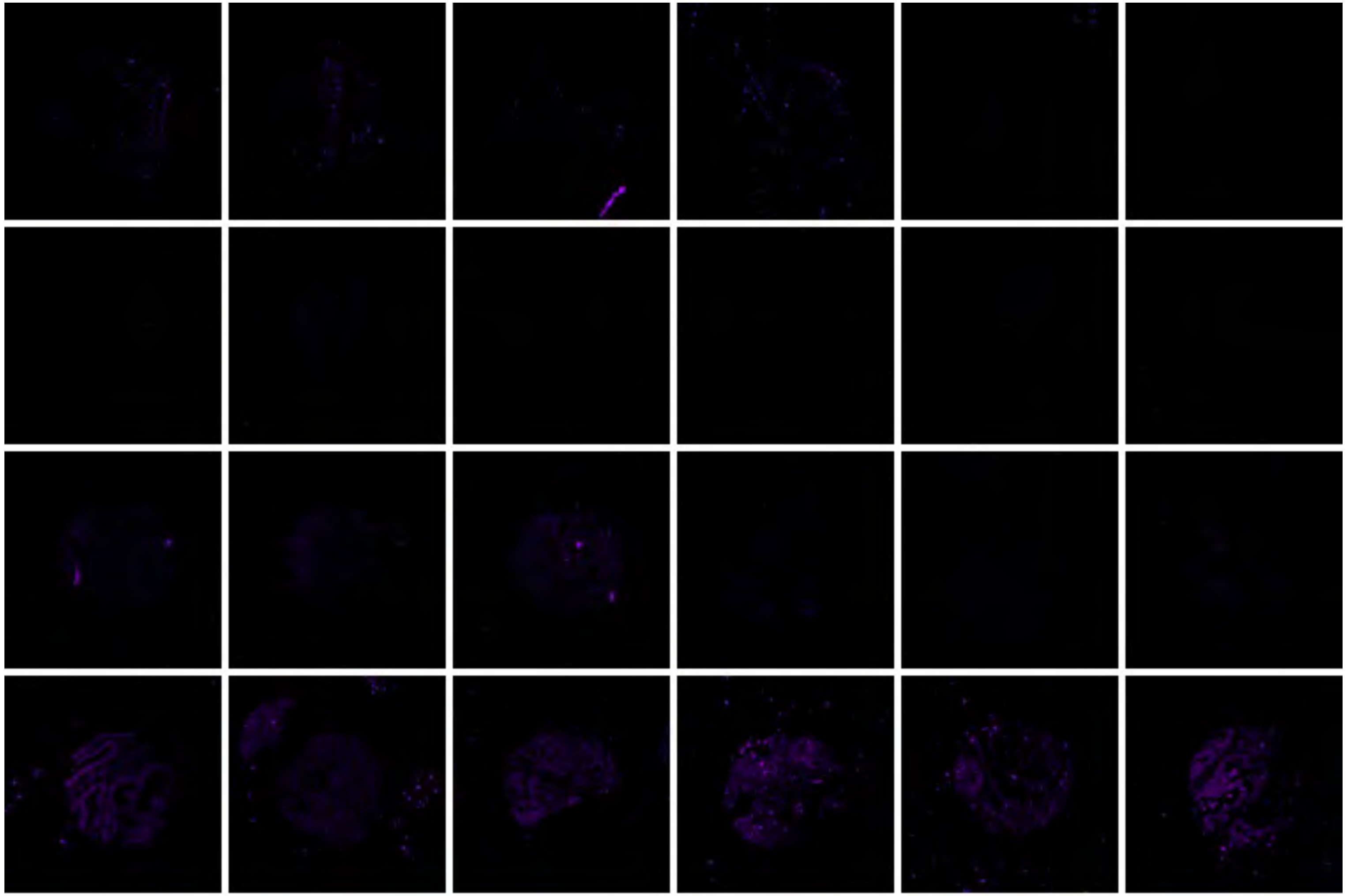

Row 7- Full RADD

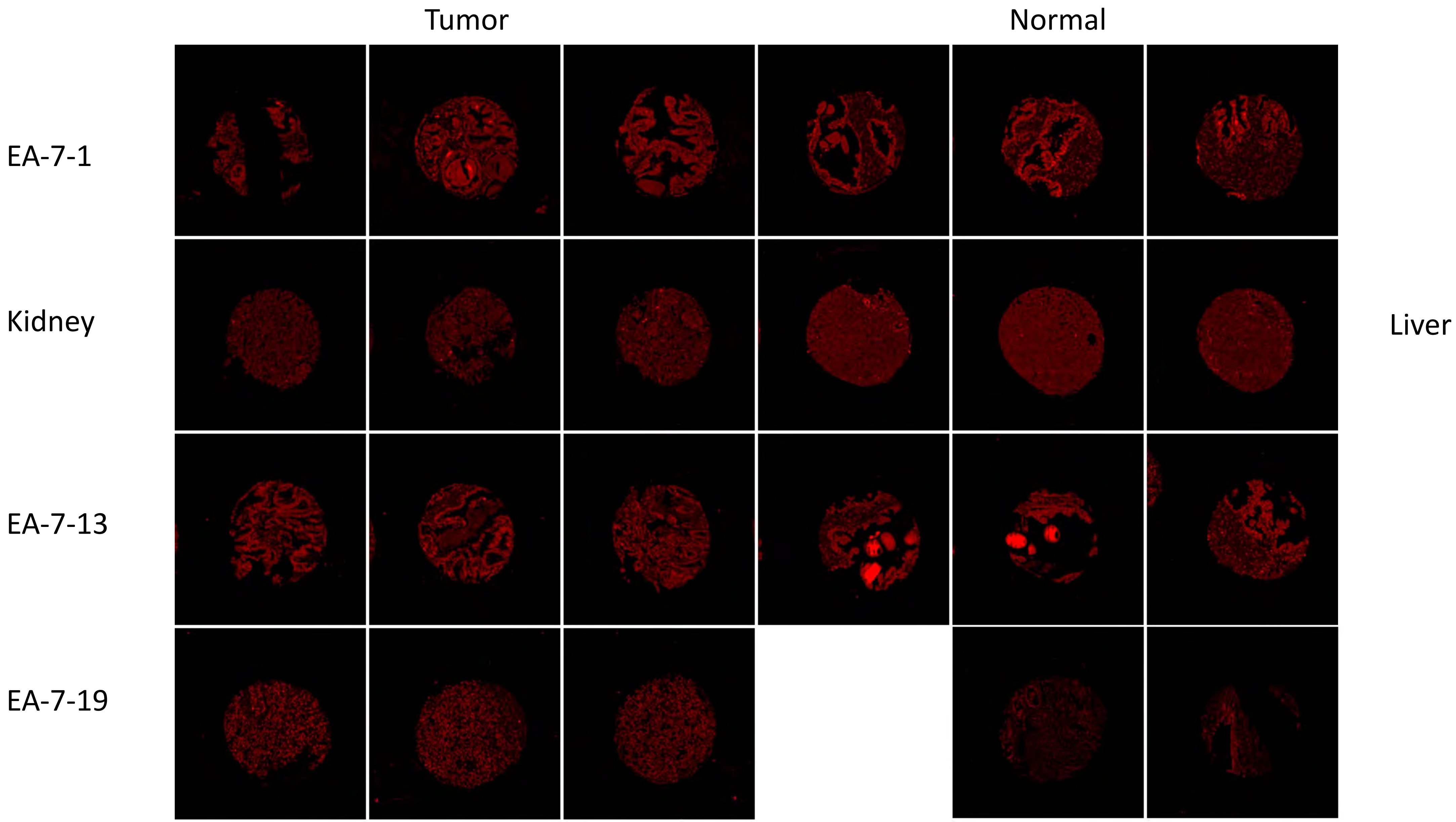

Row 7- oxRADD

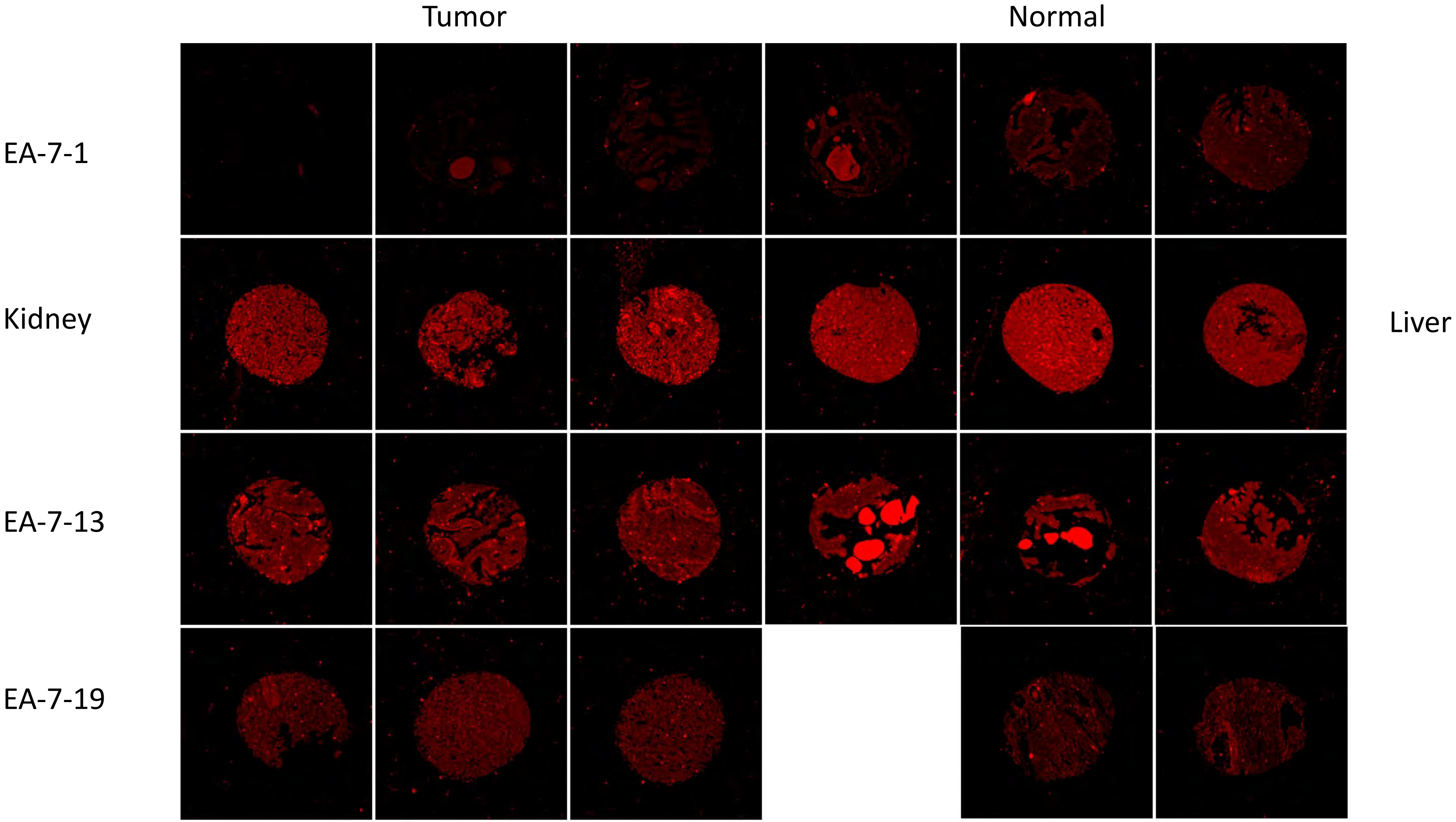

Row 7- UDG

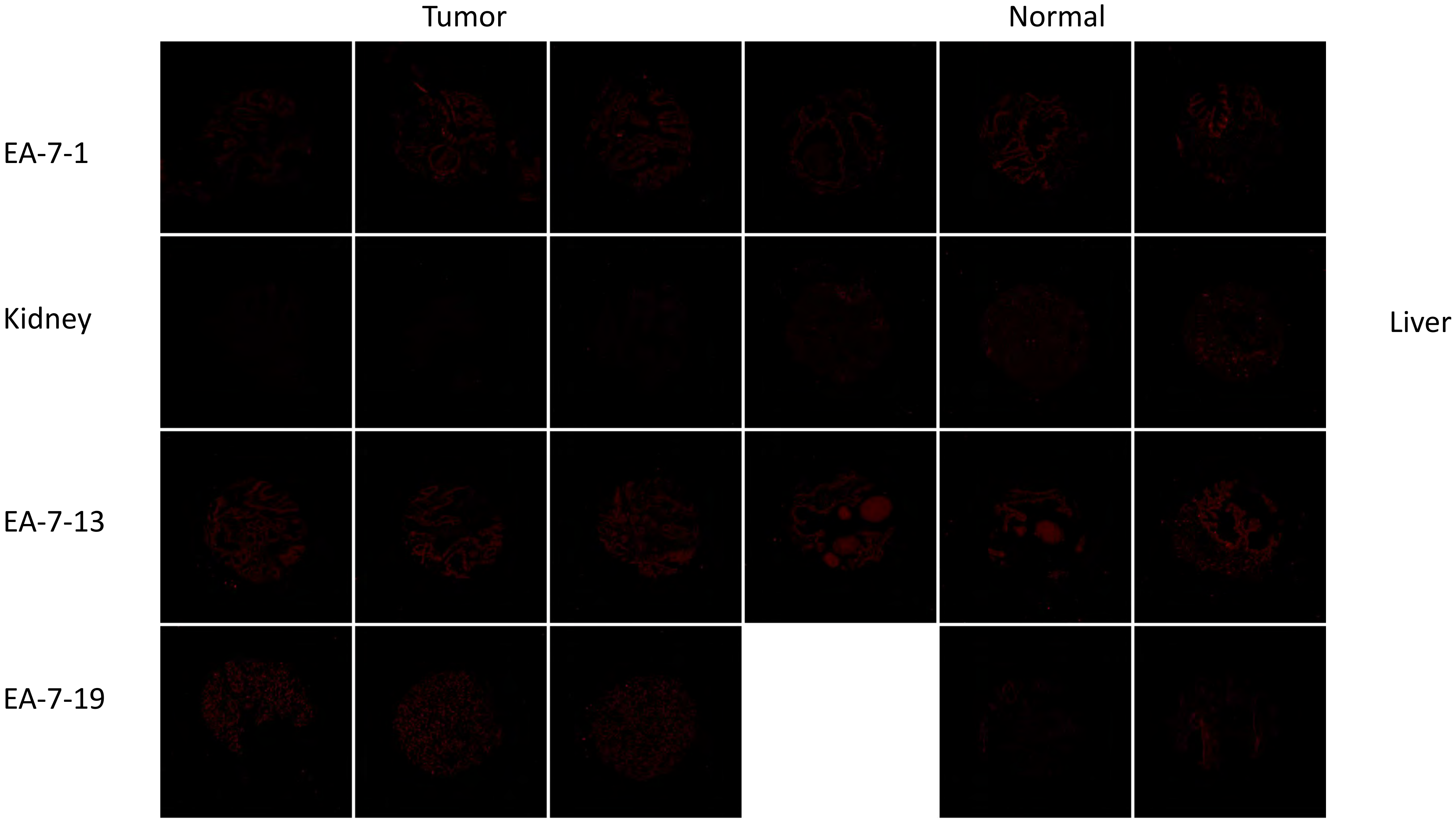

Row 7- T4PDG

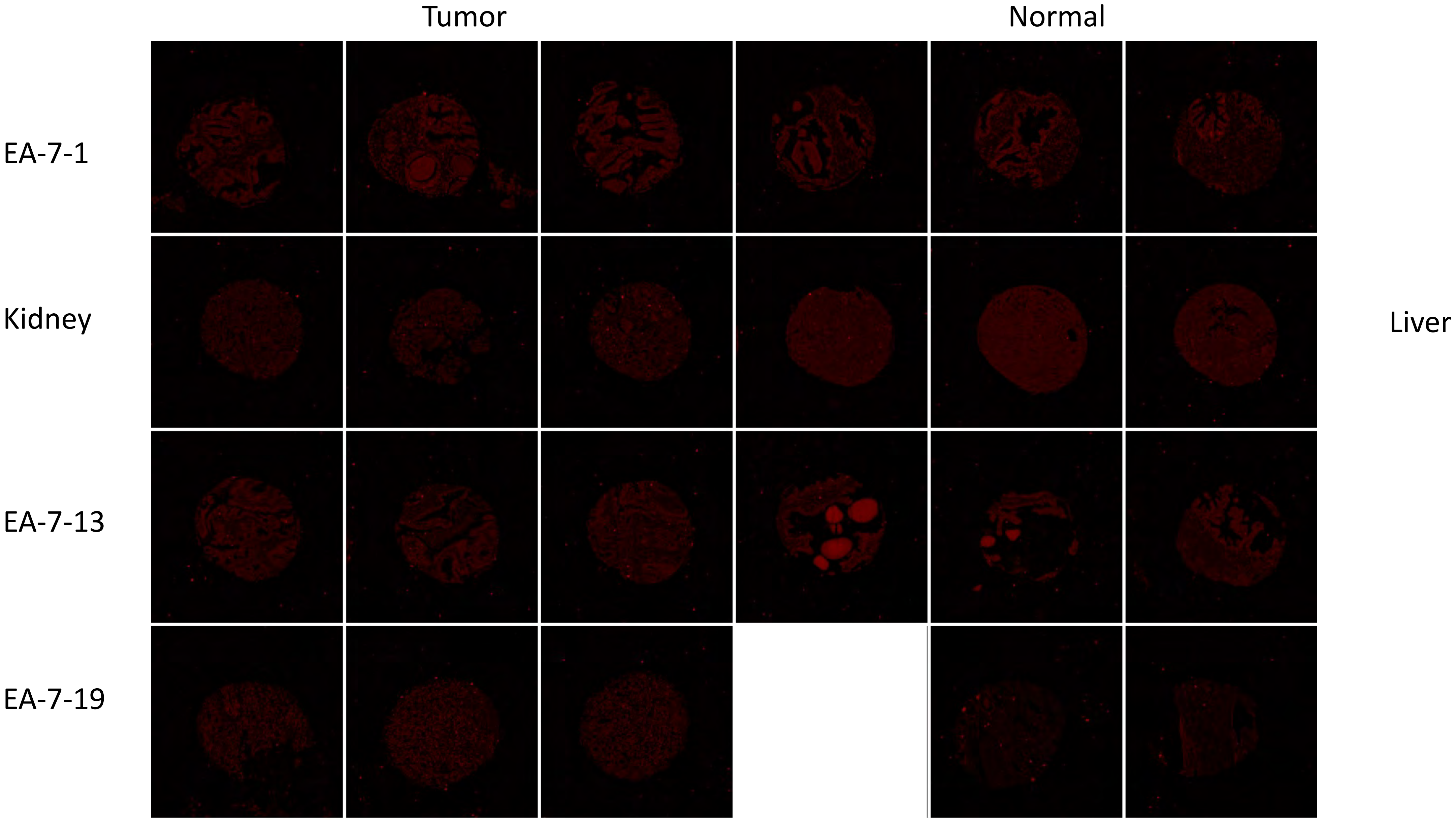

Row 7- XRCC1

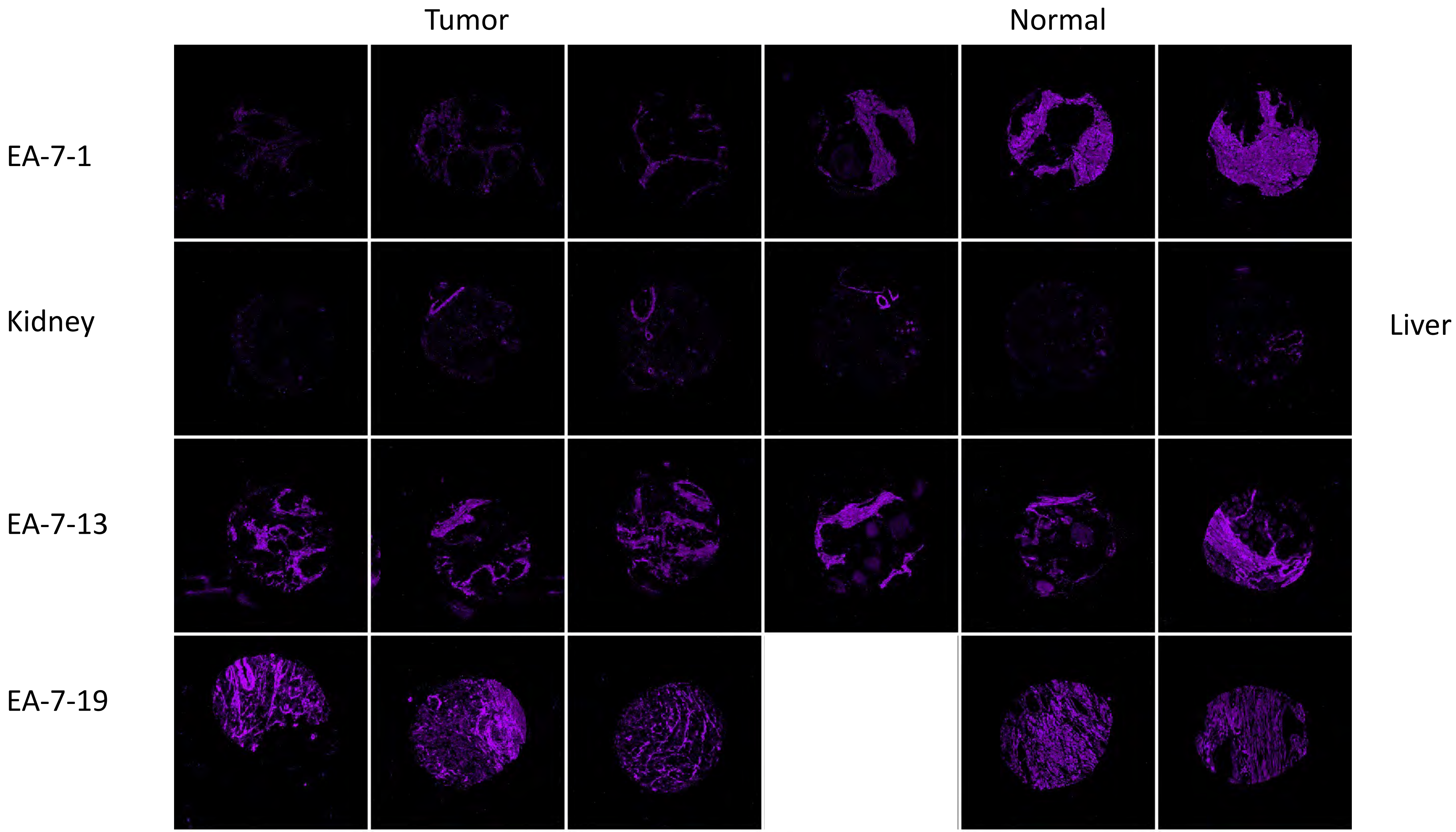

Row 7- PARP1

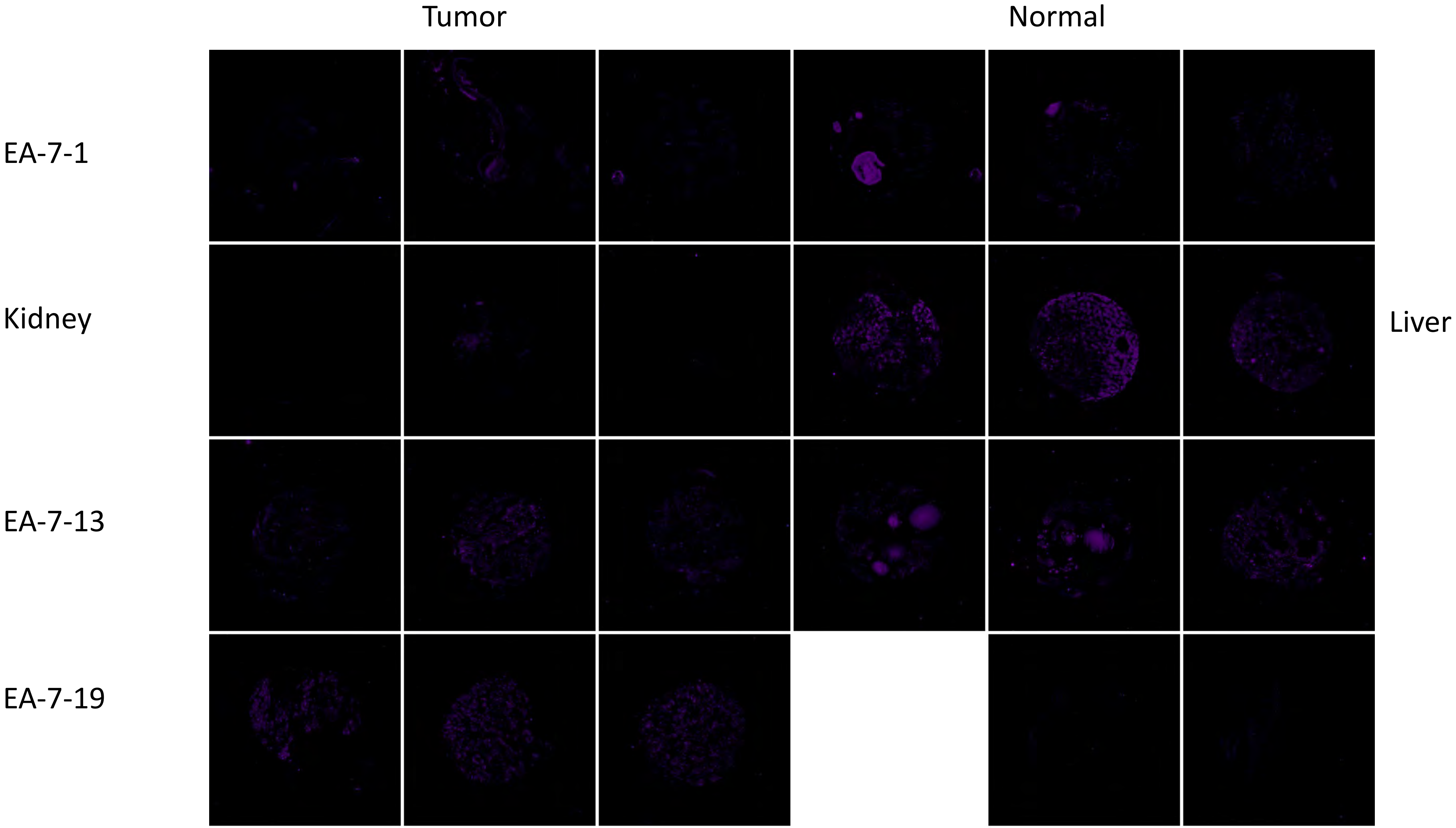

Row 7- UNG

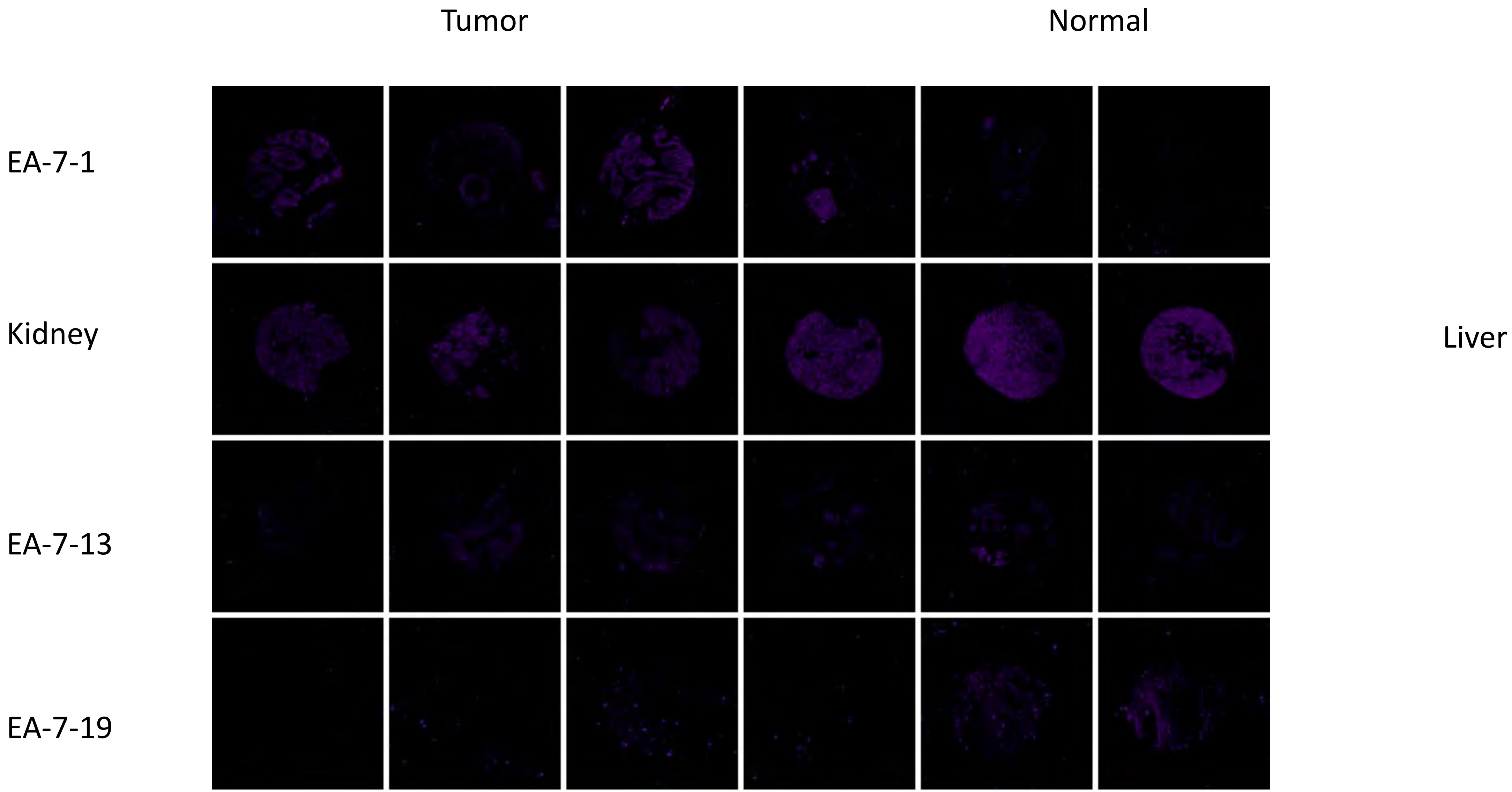

Row 8- Full RADD

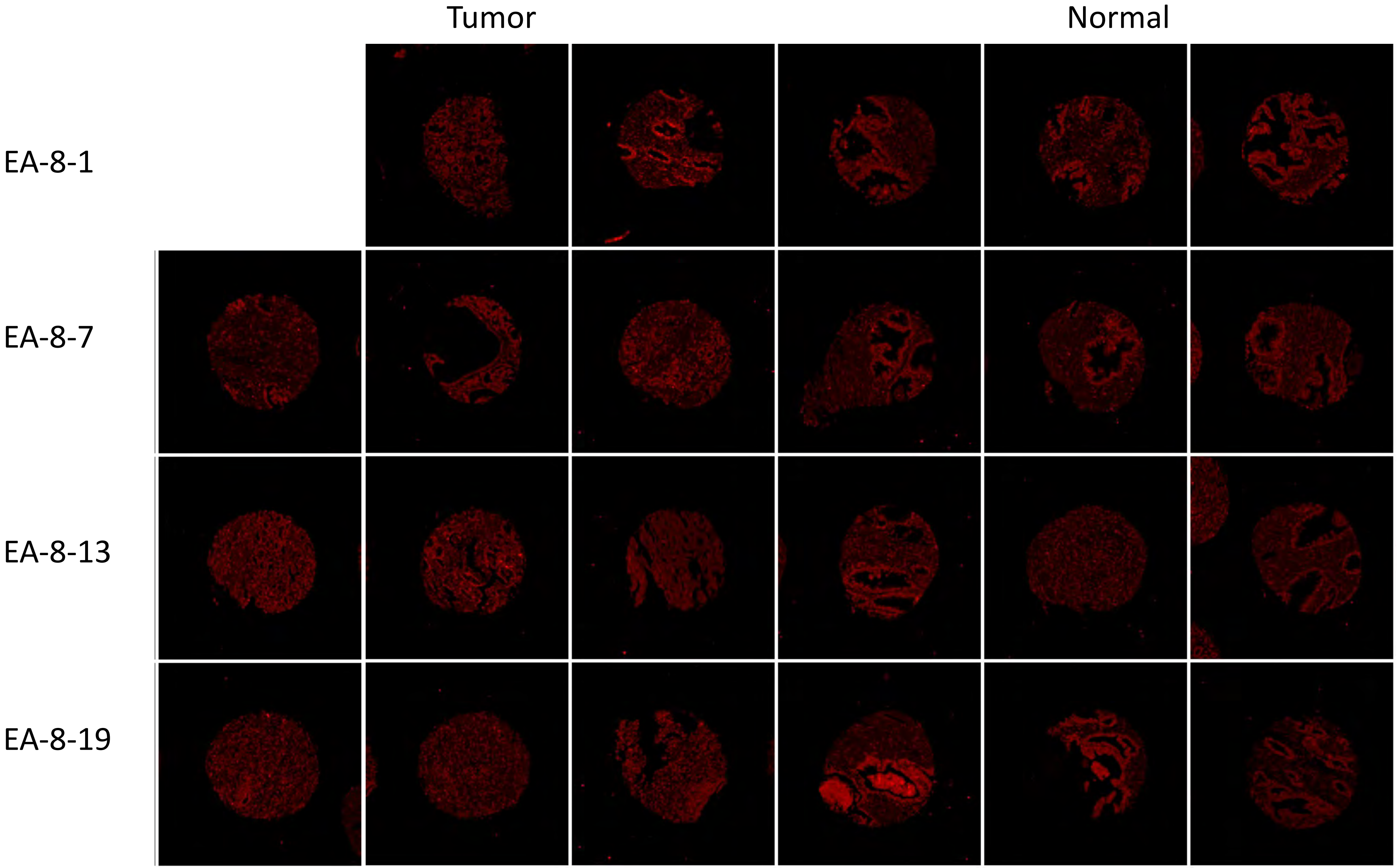

Row 8- oxRADD

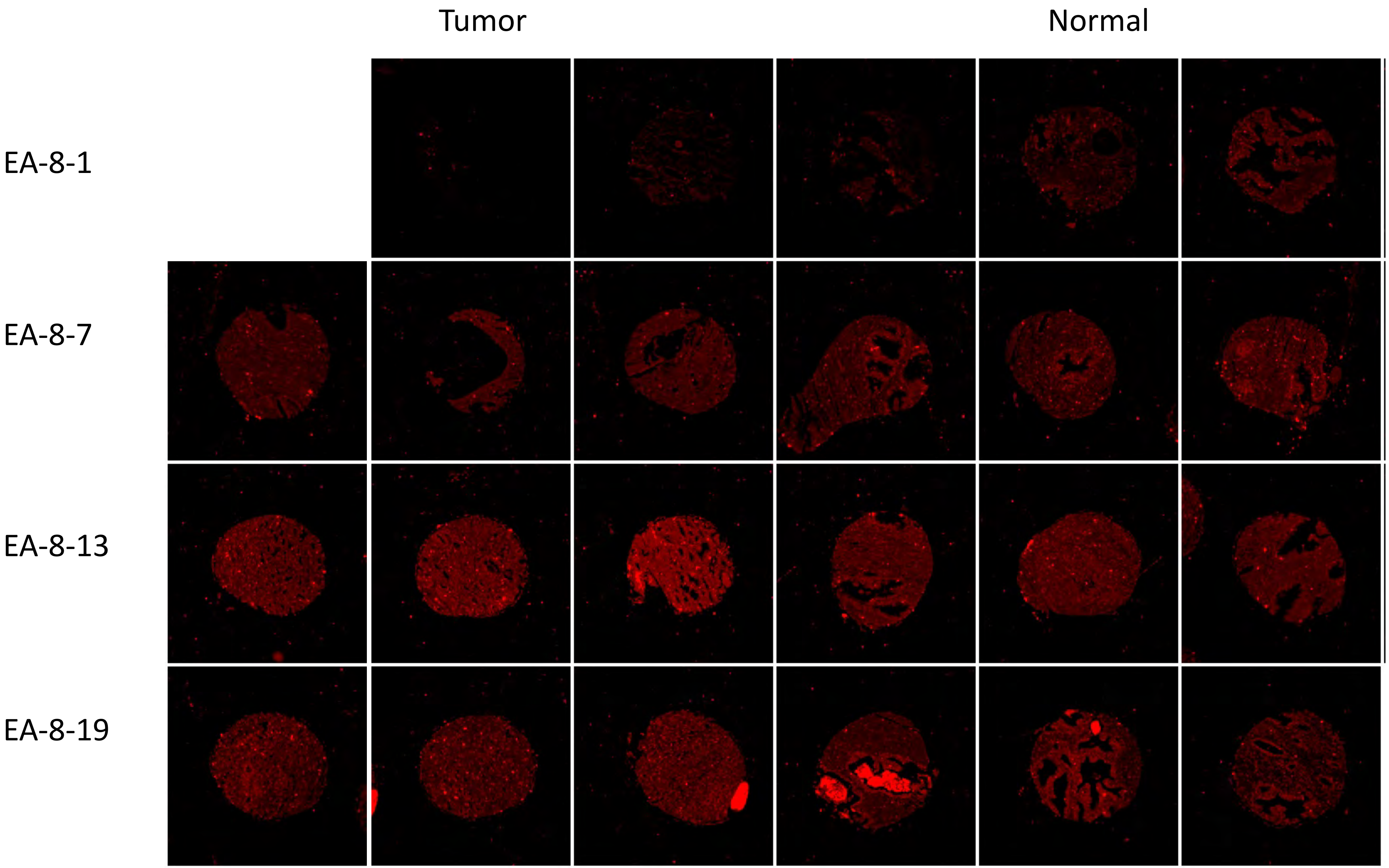

Row 8- UDG

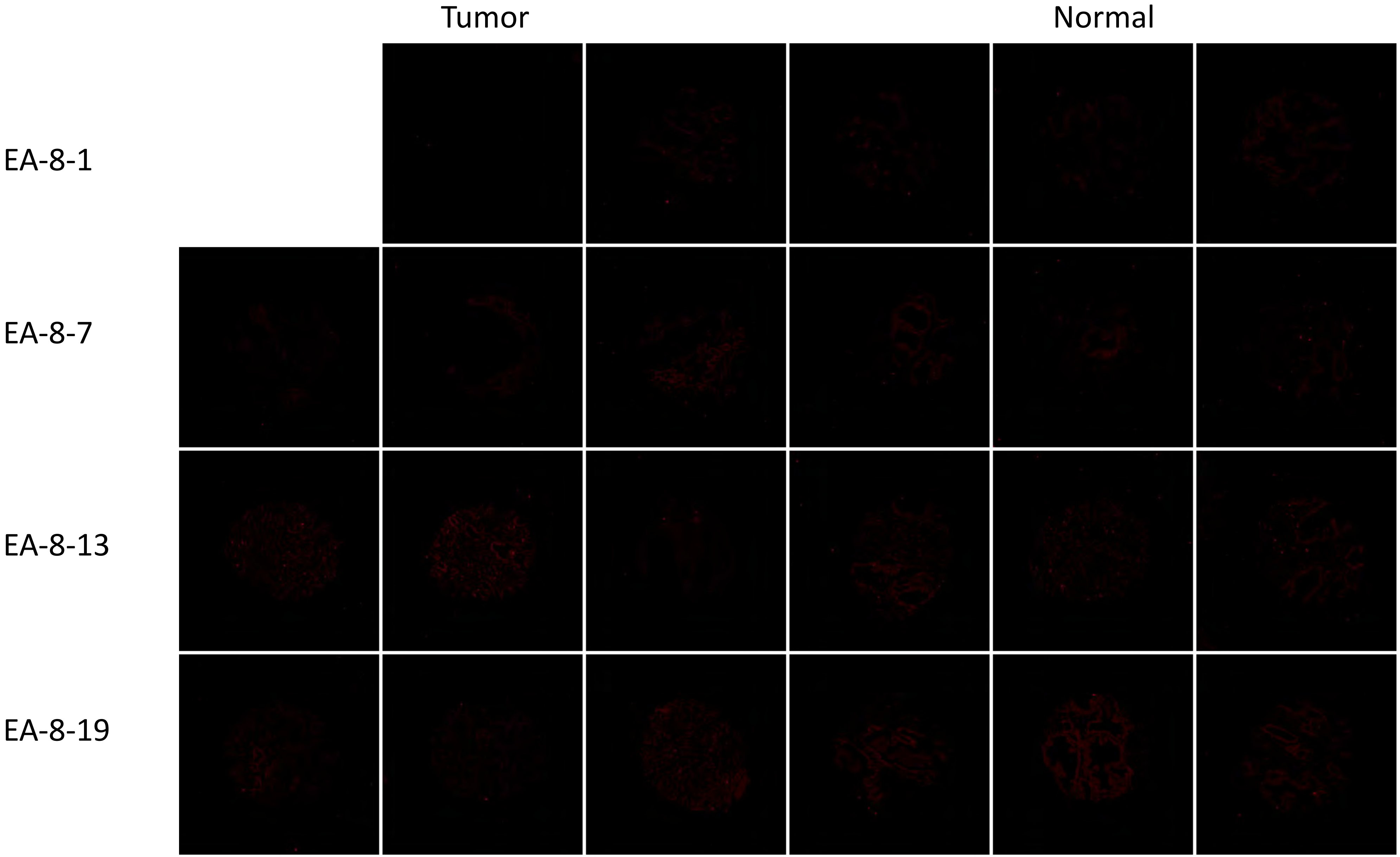

Row 8- T4PDG

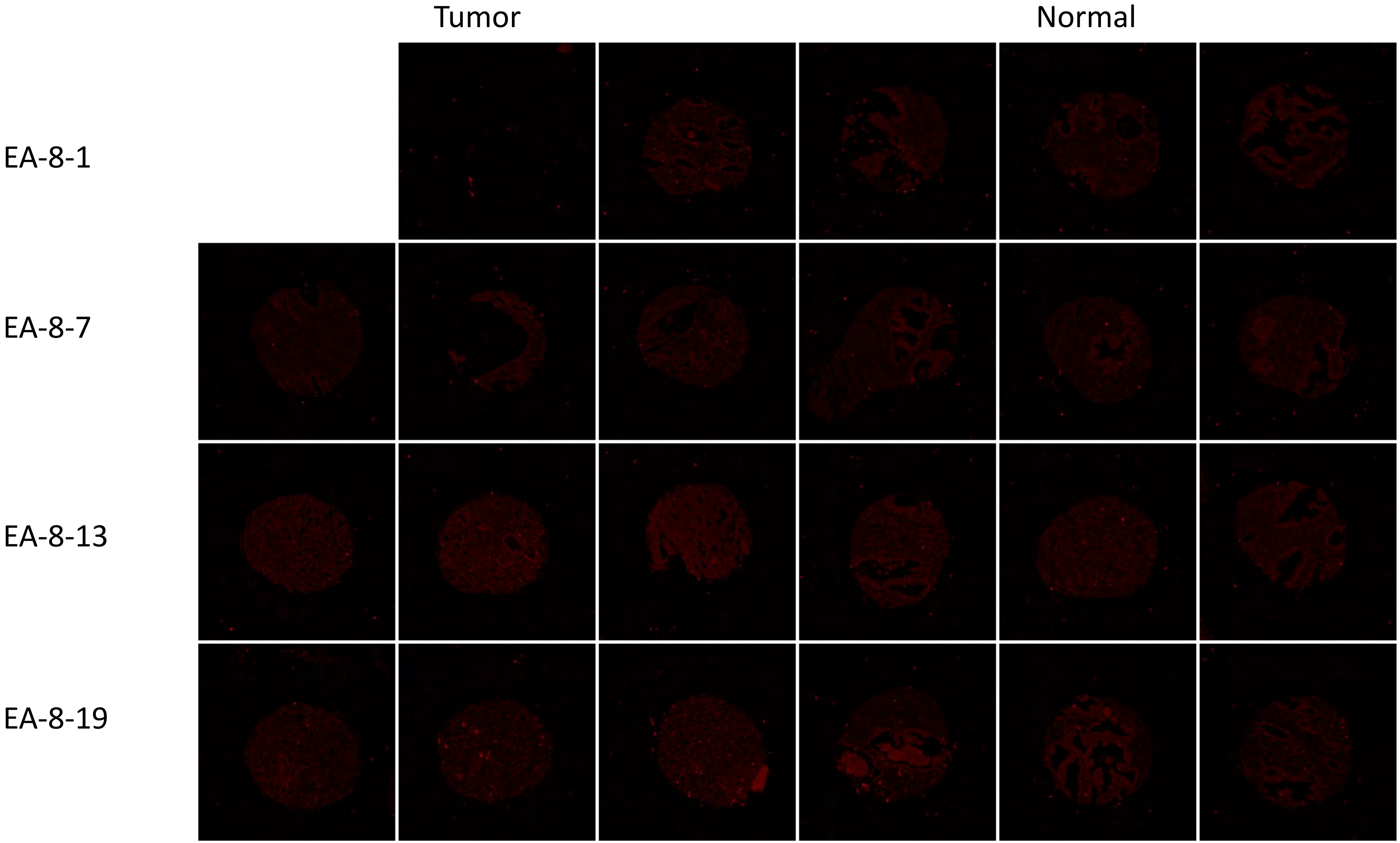

Row 8- XRCC1

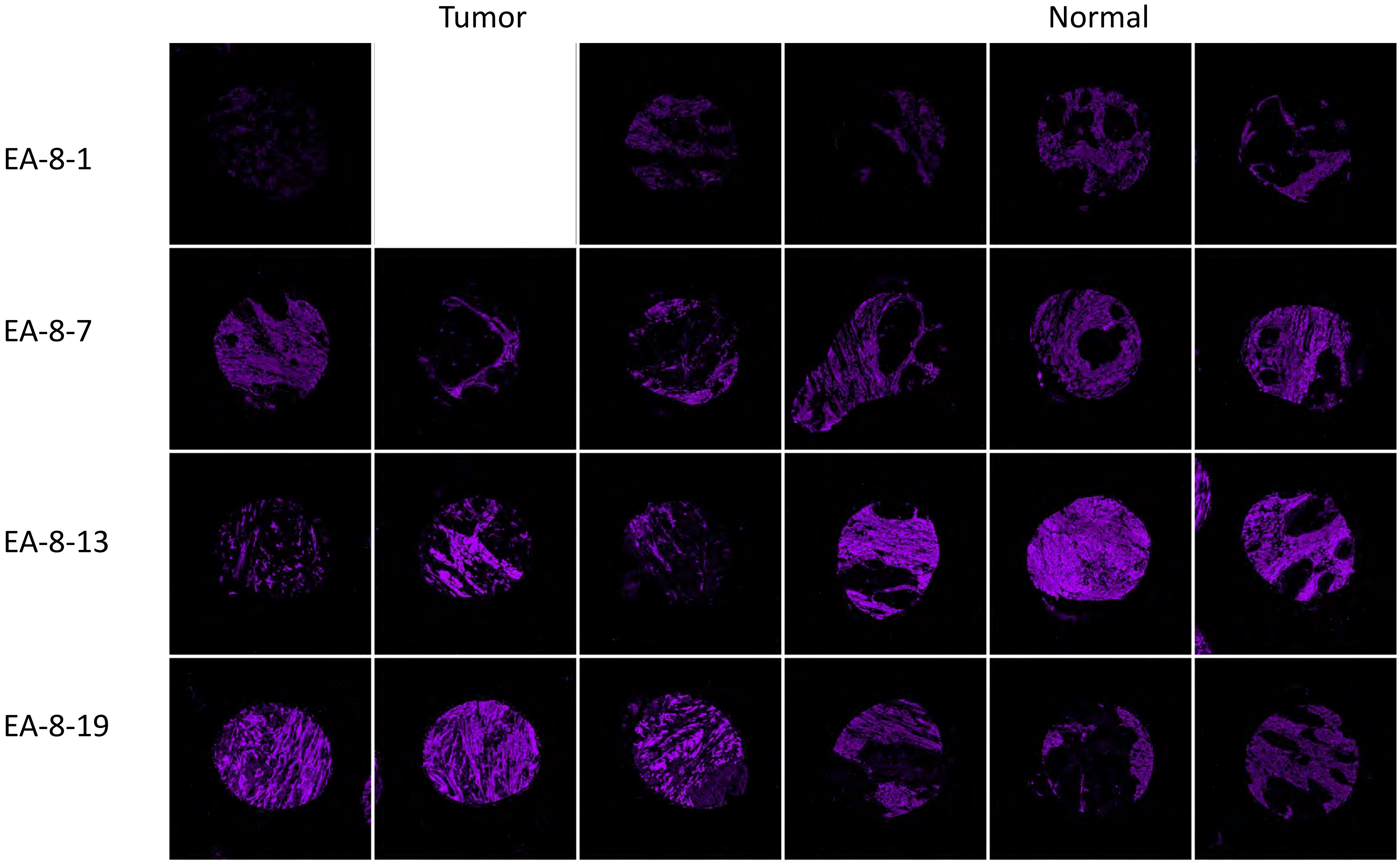

Row 8- PARP1

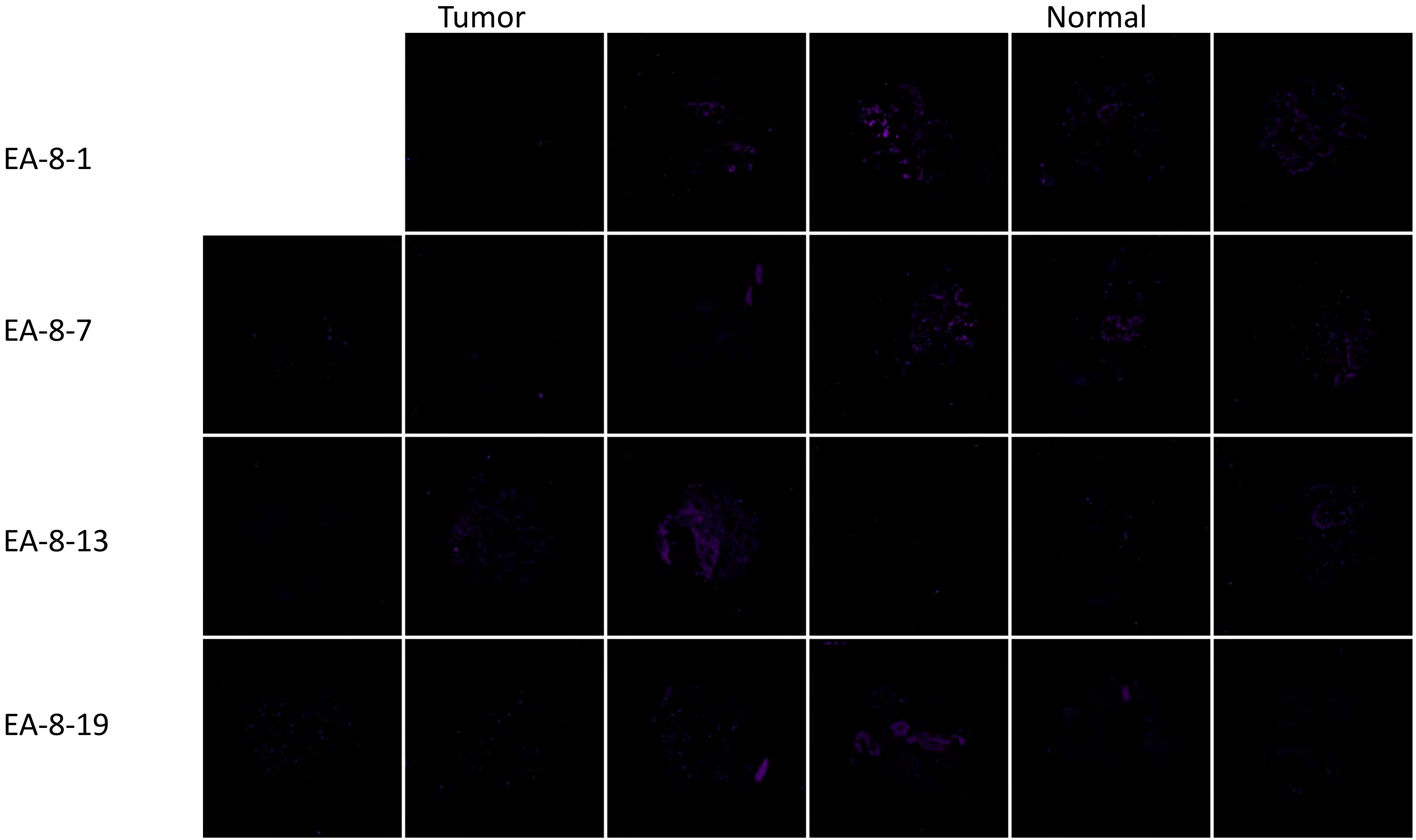

Row 8- UNG

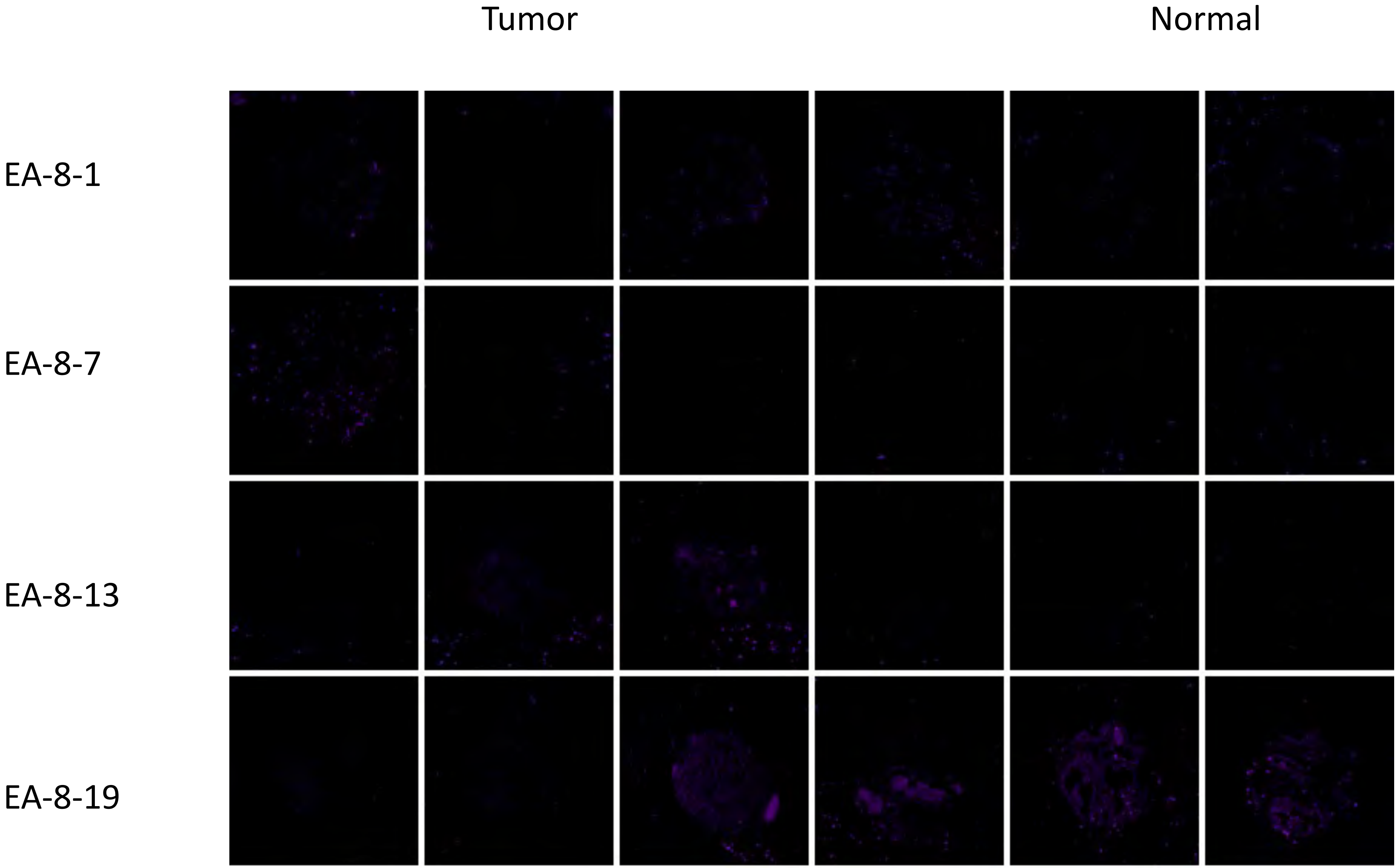

Row 9- Full RADD

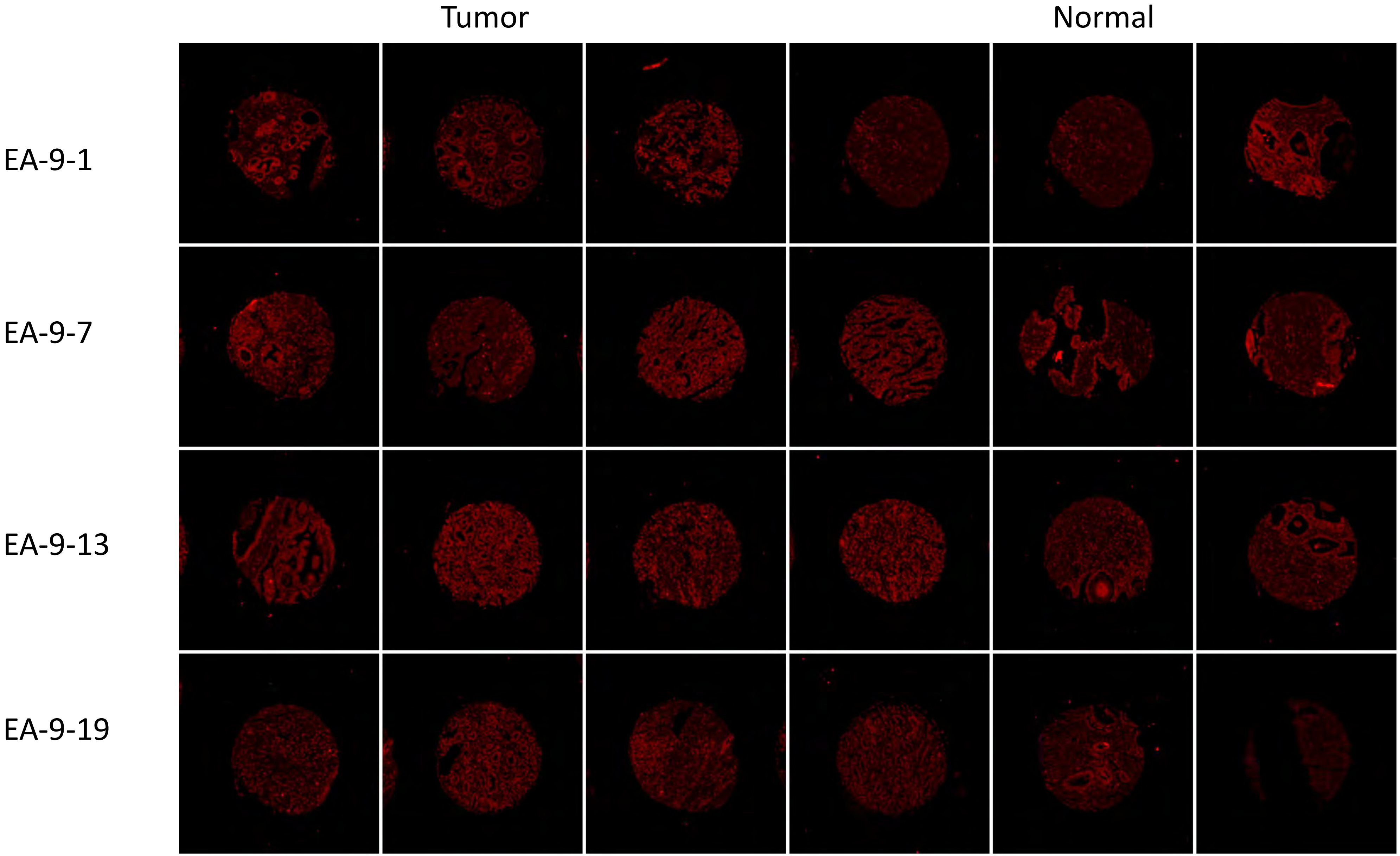

Row 9- oxRADD

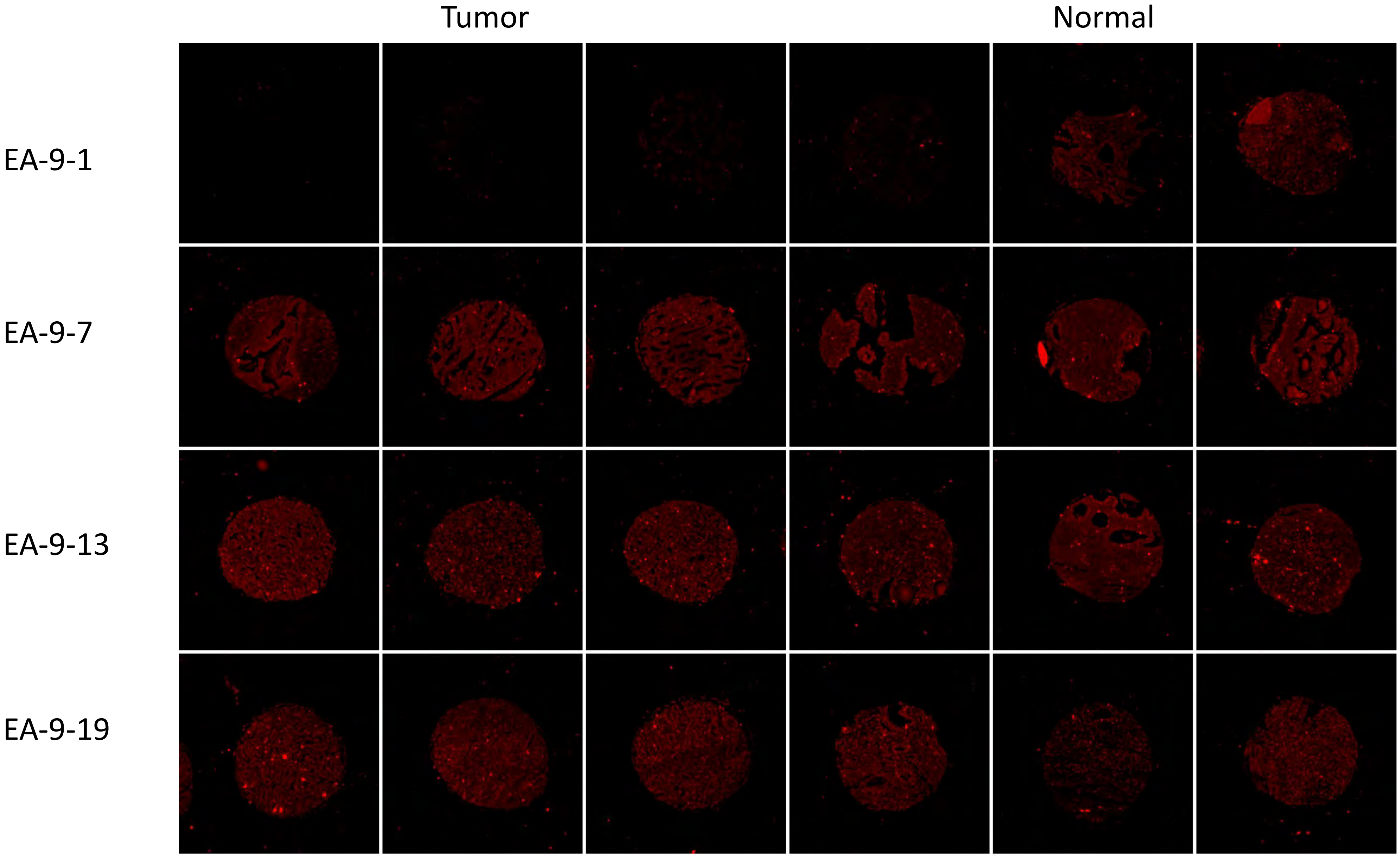

Row 9- UDG

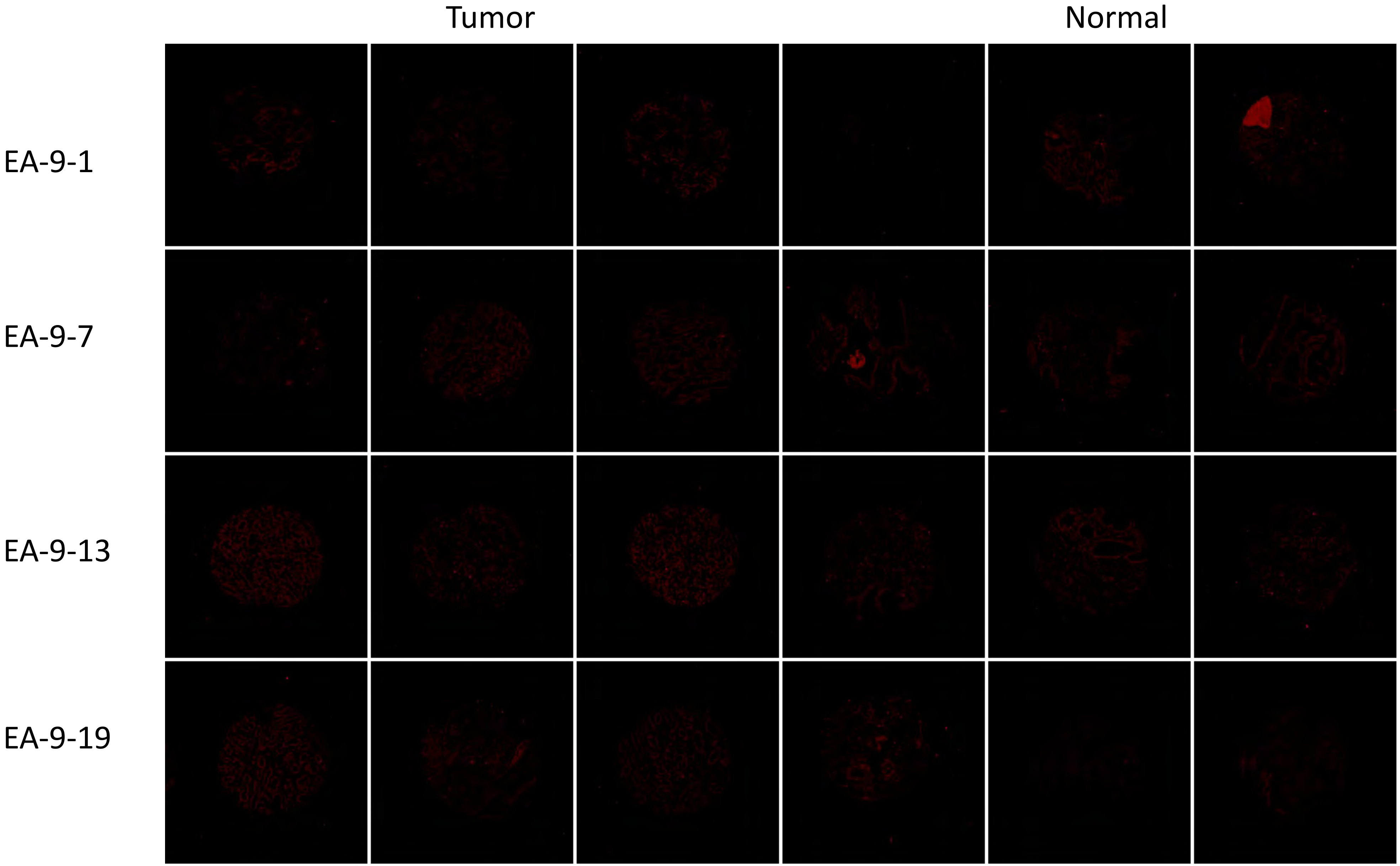

Row 9- T4PDG

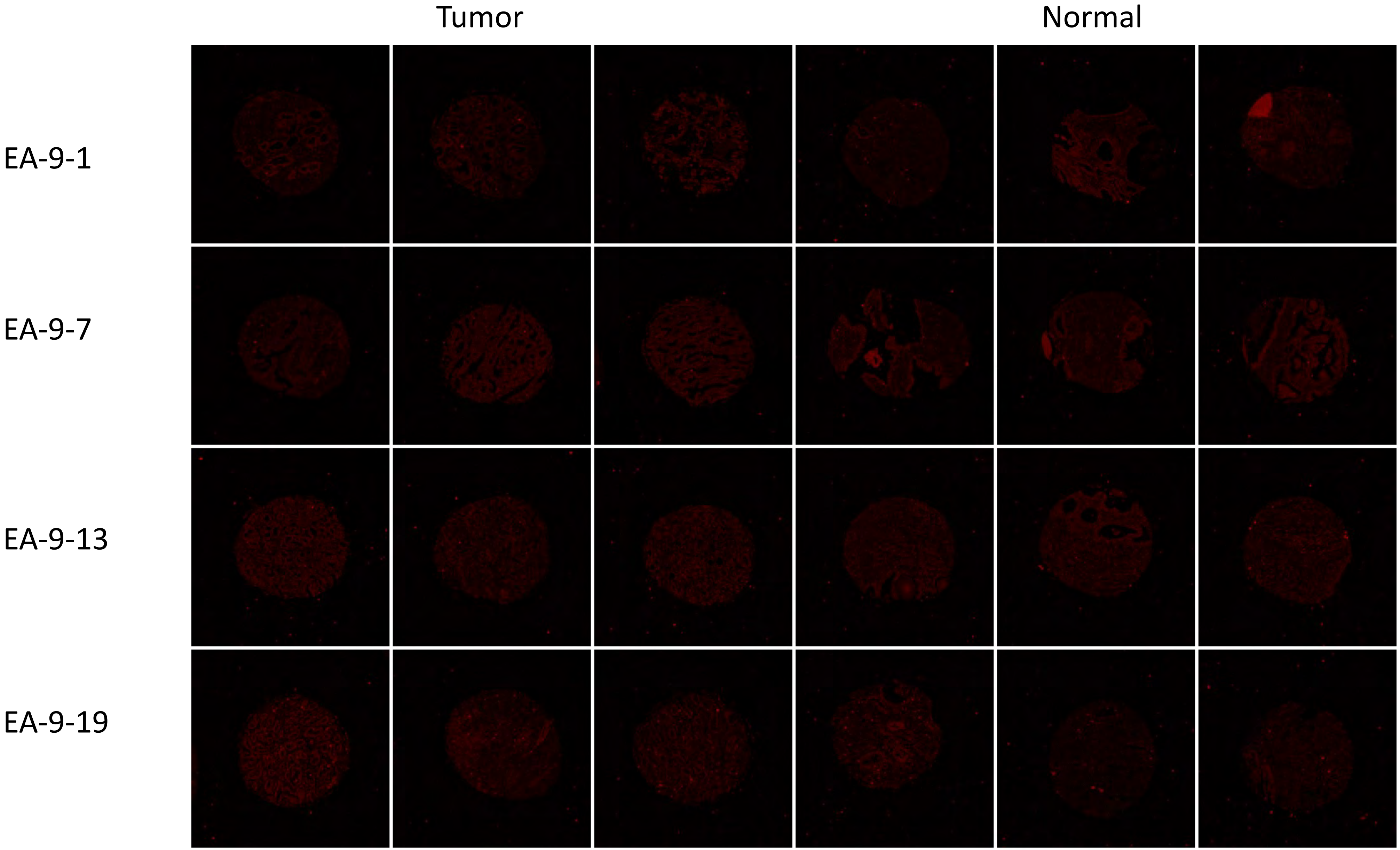

Row 9- XRCC1

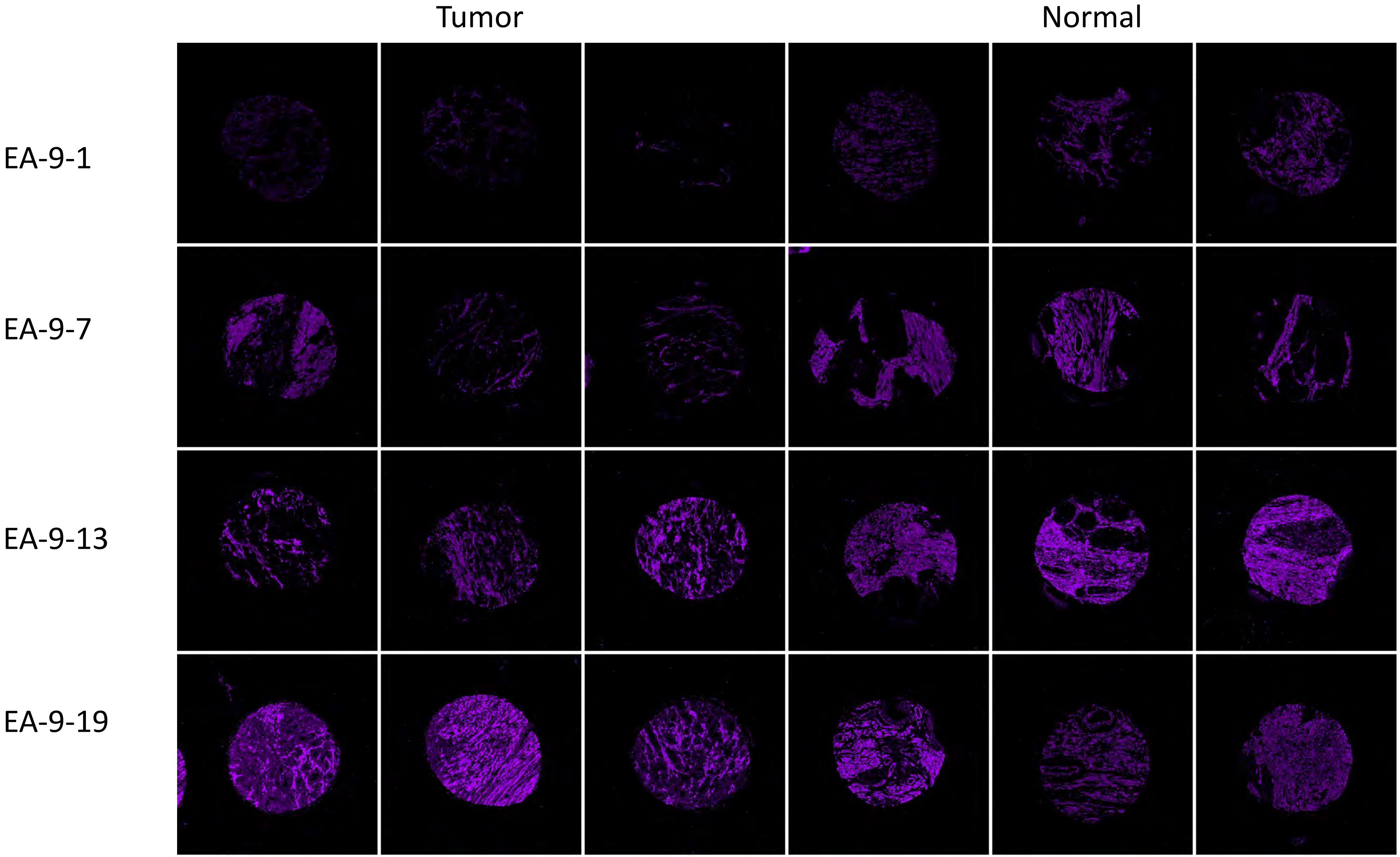

Row 9- PARP1

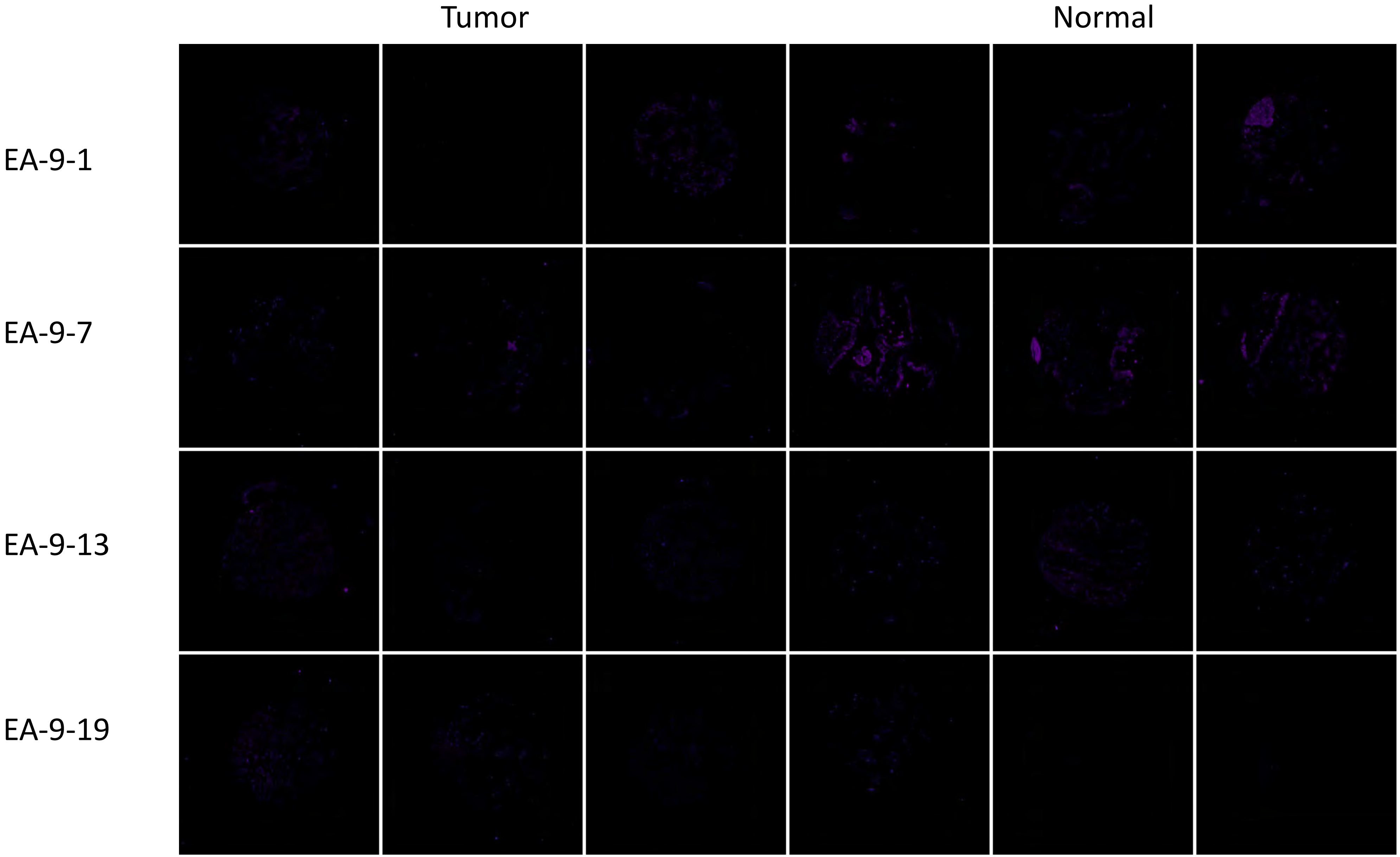

Row 9- UNG

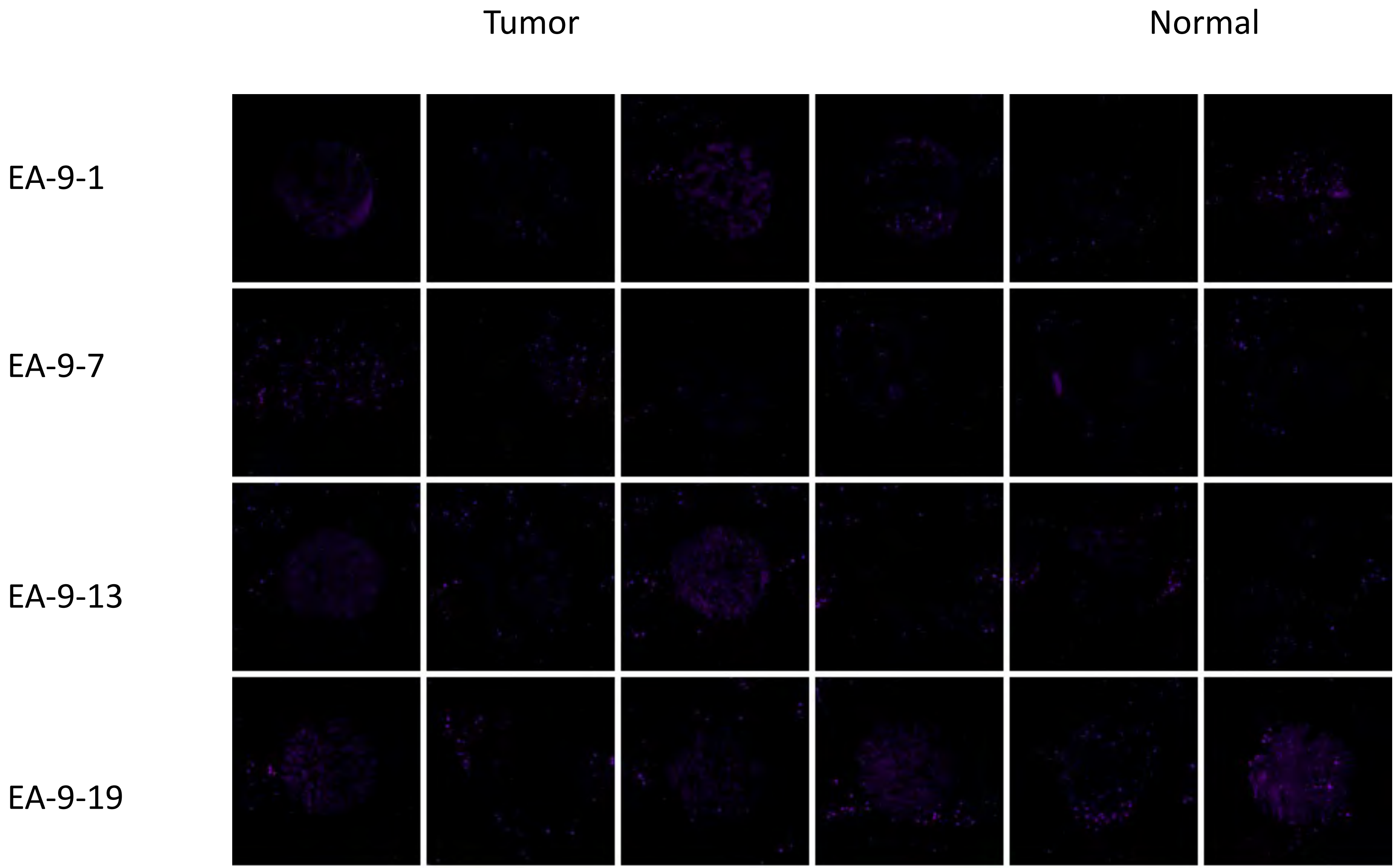

Row 10- Full RADD

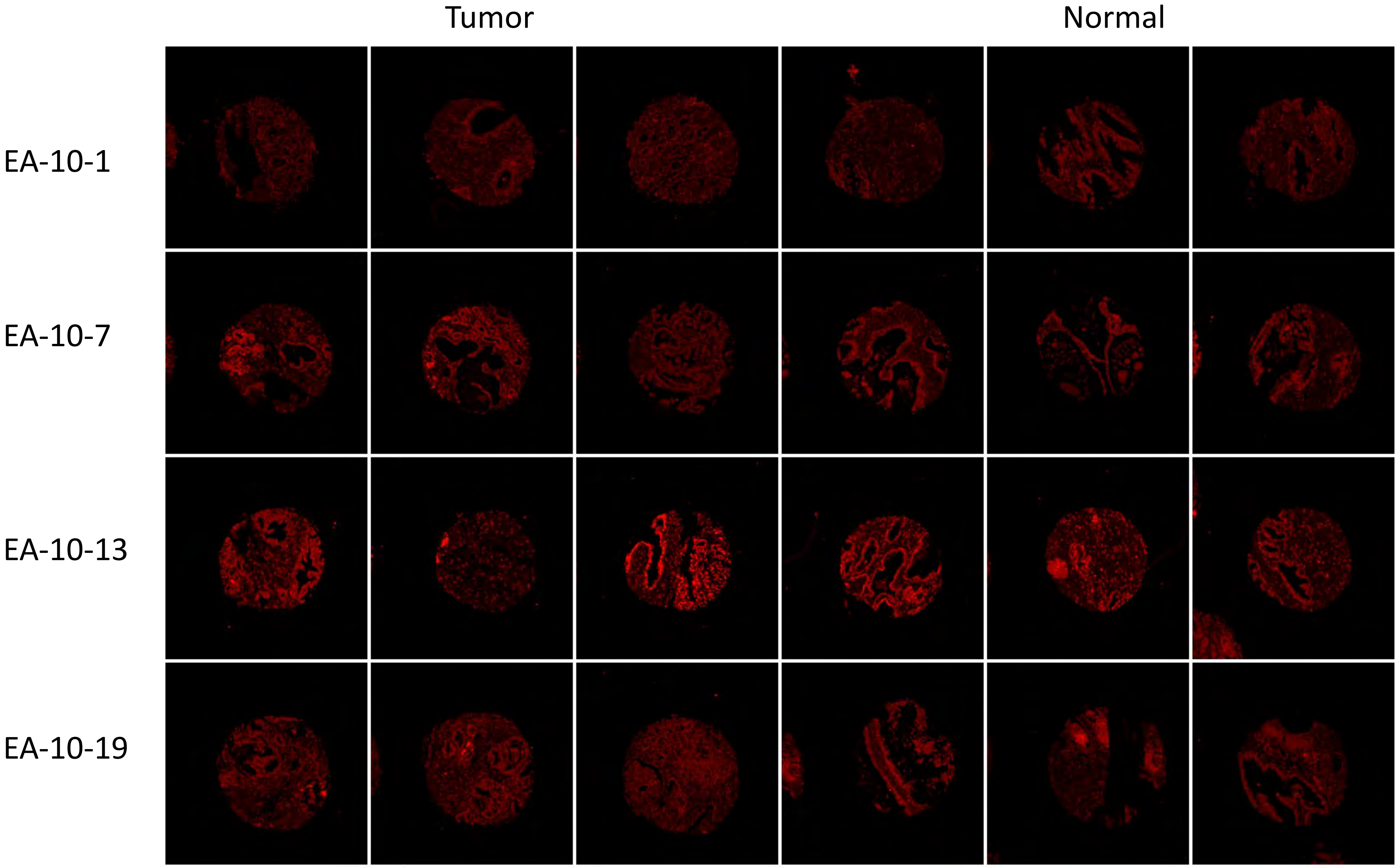

Row 10- oxRADD

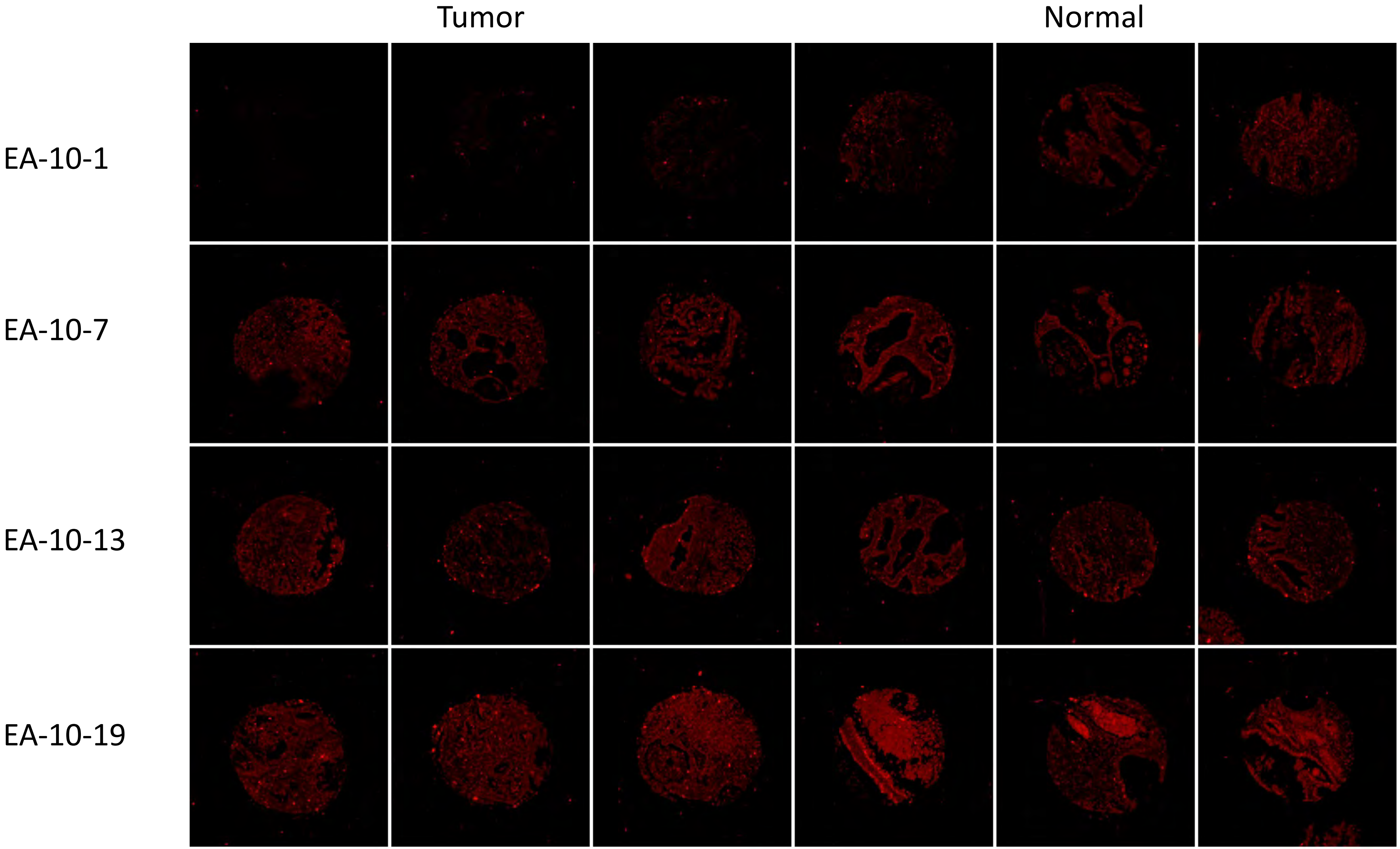

Row 10- UDG

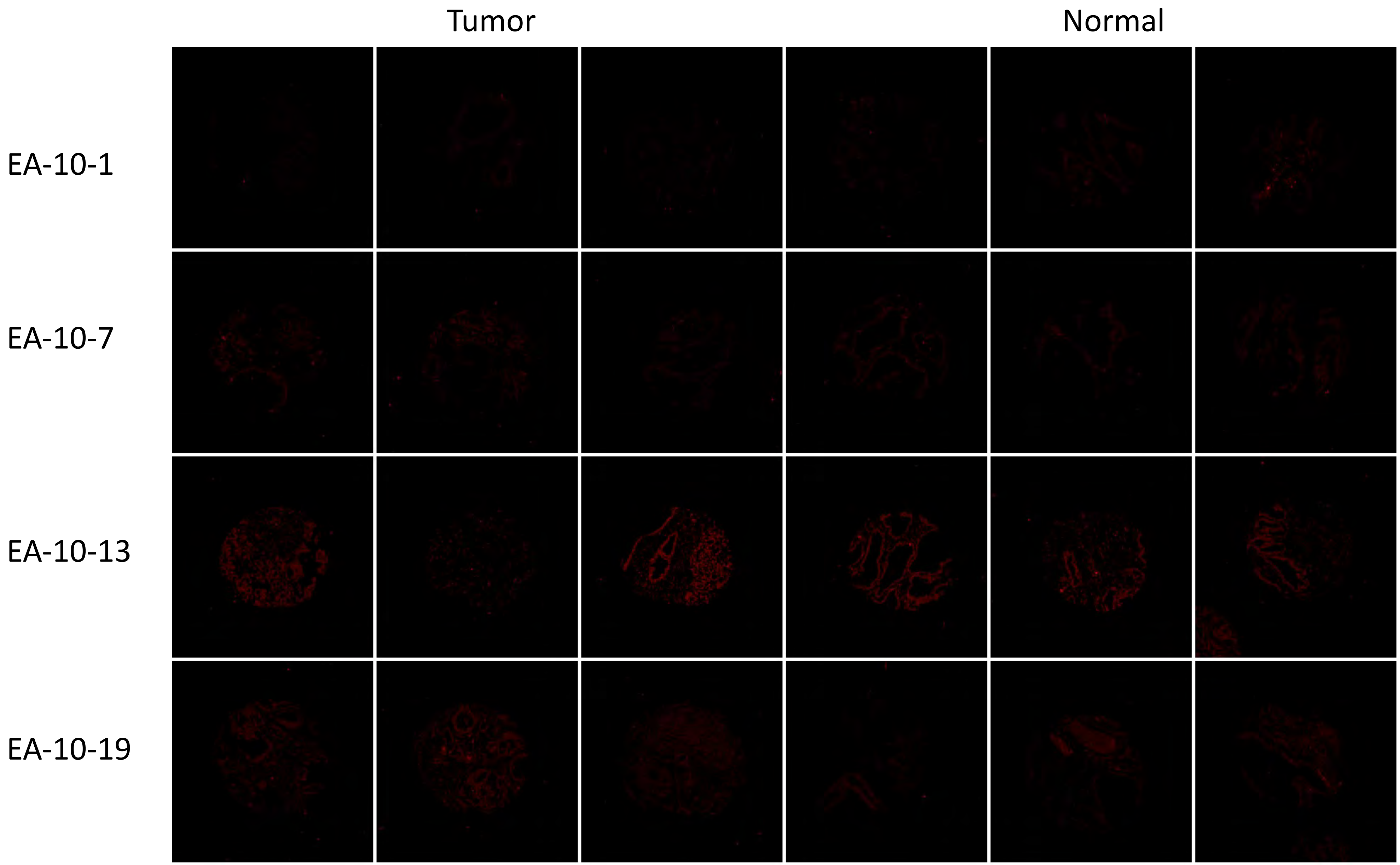

Row 10- T4PDG

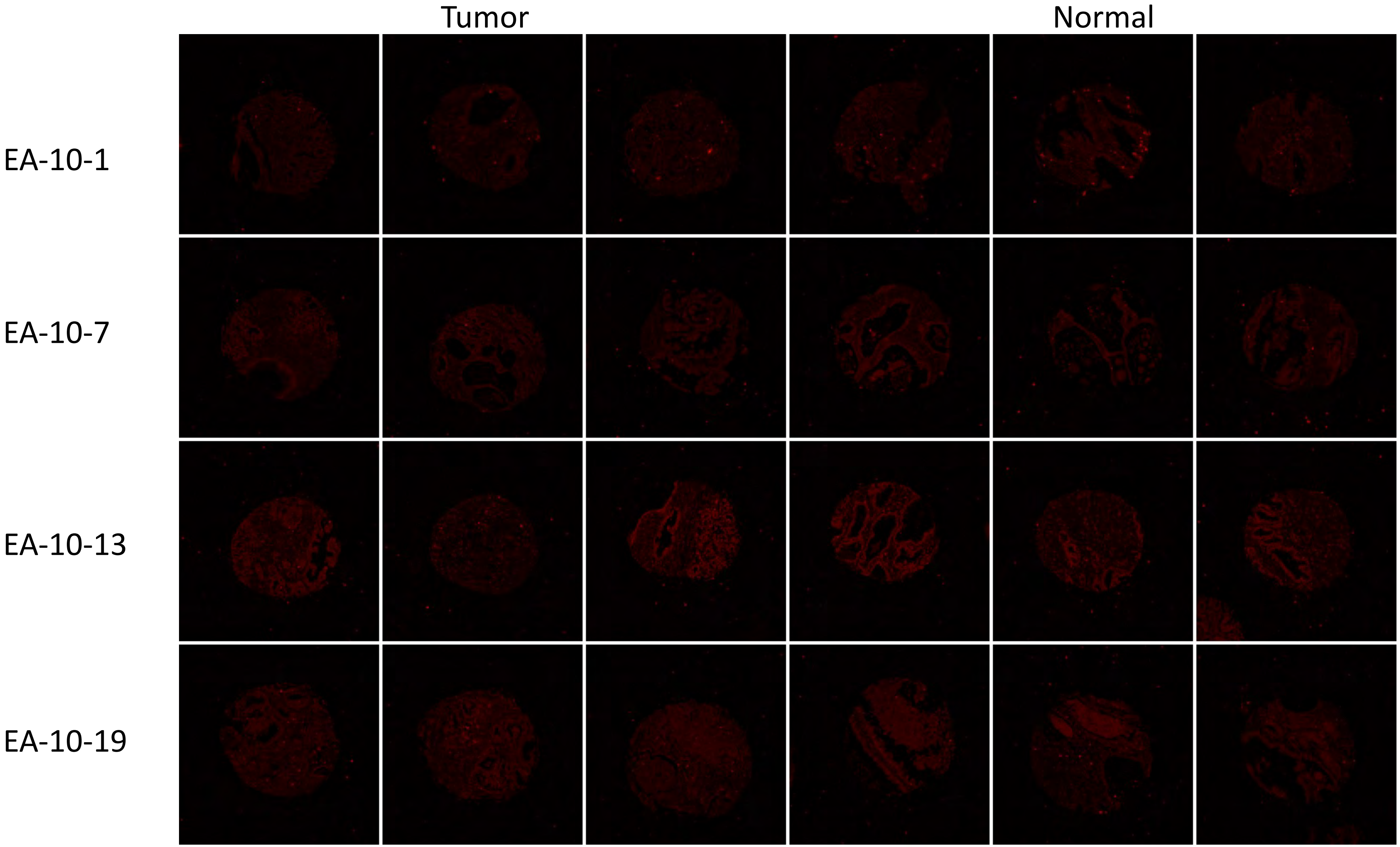

Row 10- XRCC1

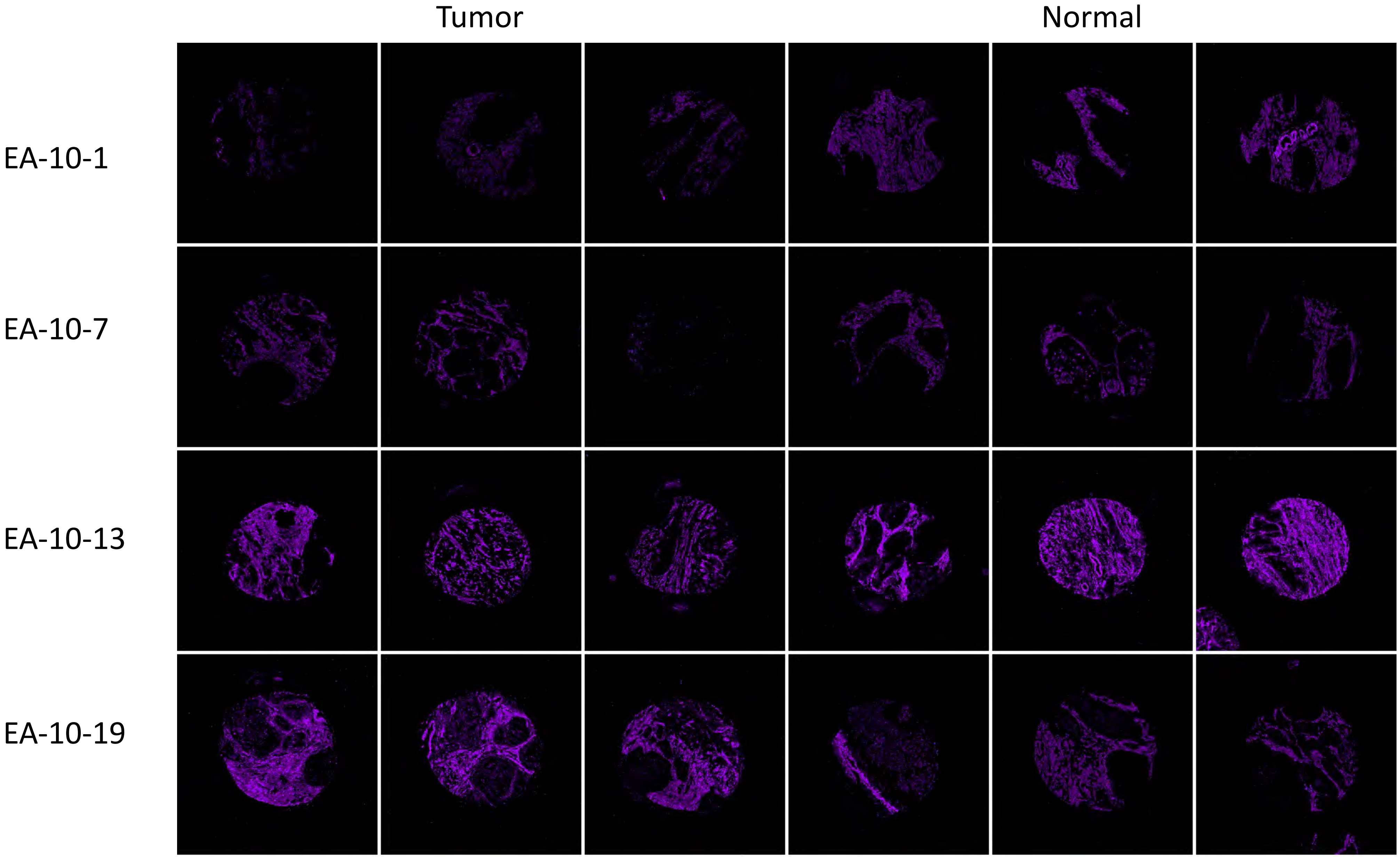

Row 10- PARP1

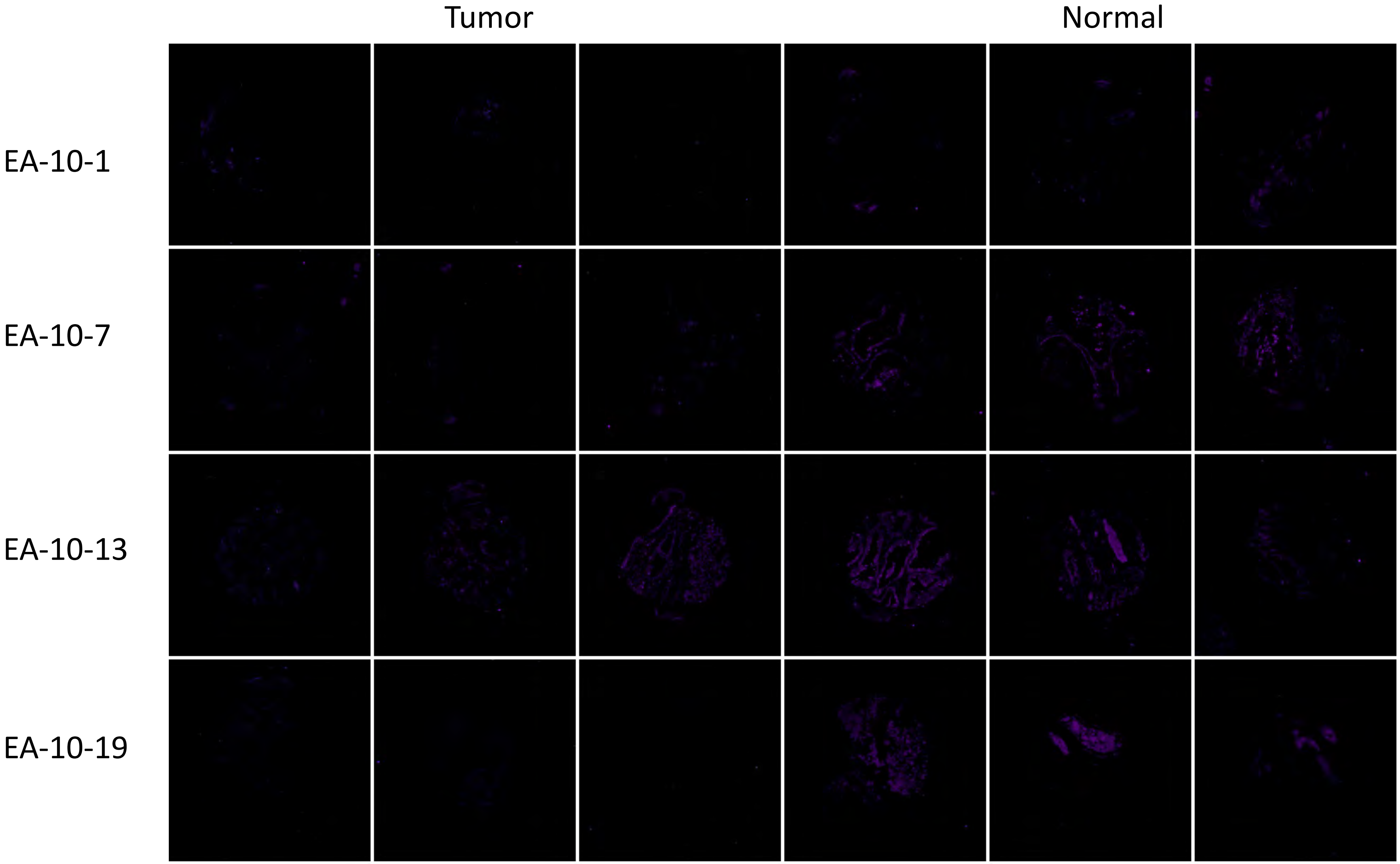

Row 10- UNG

Tumor Normal

EA-10-1

EA-10-7

EA-10-13

EA-10-19

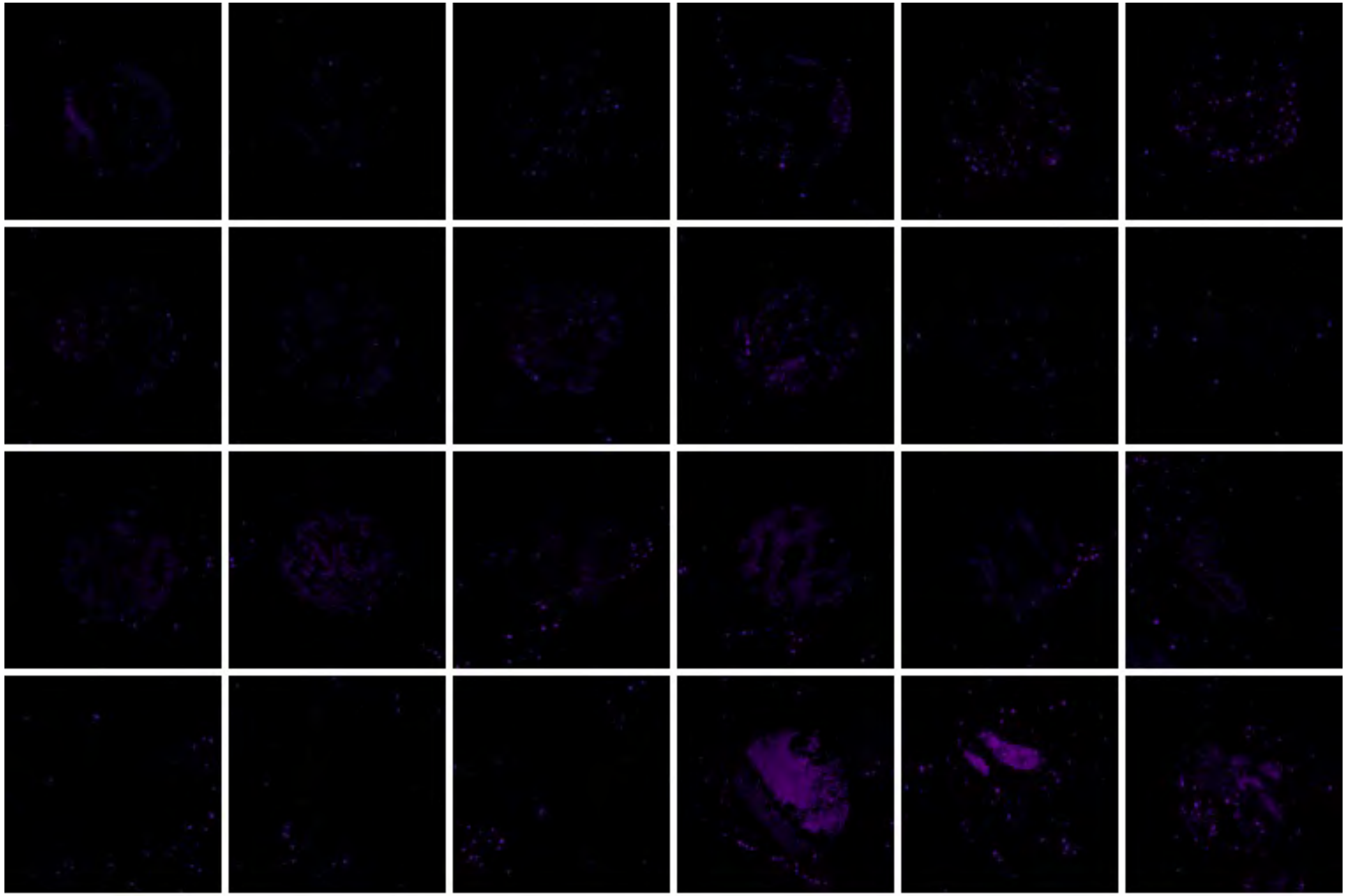

Row 11- Full RADD

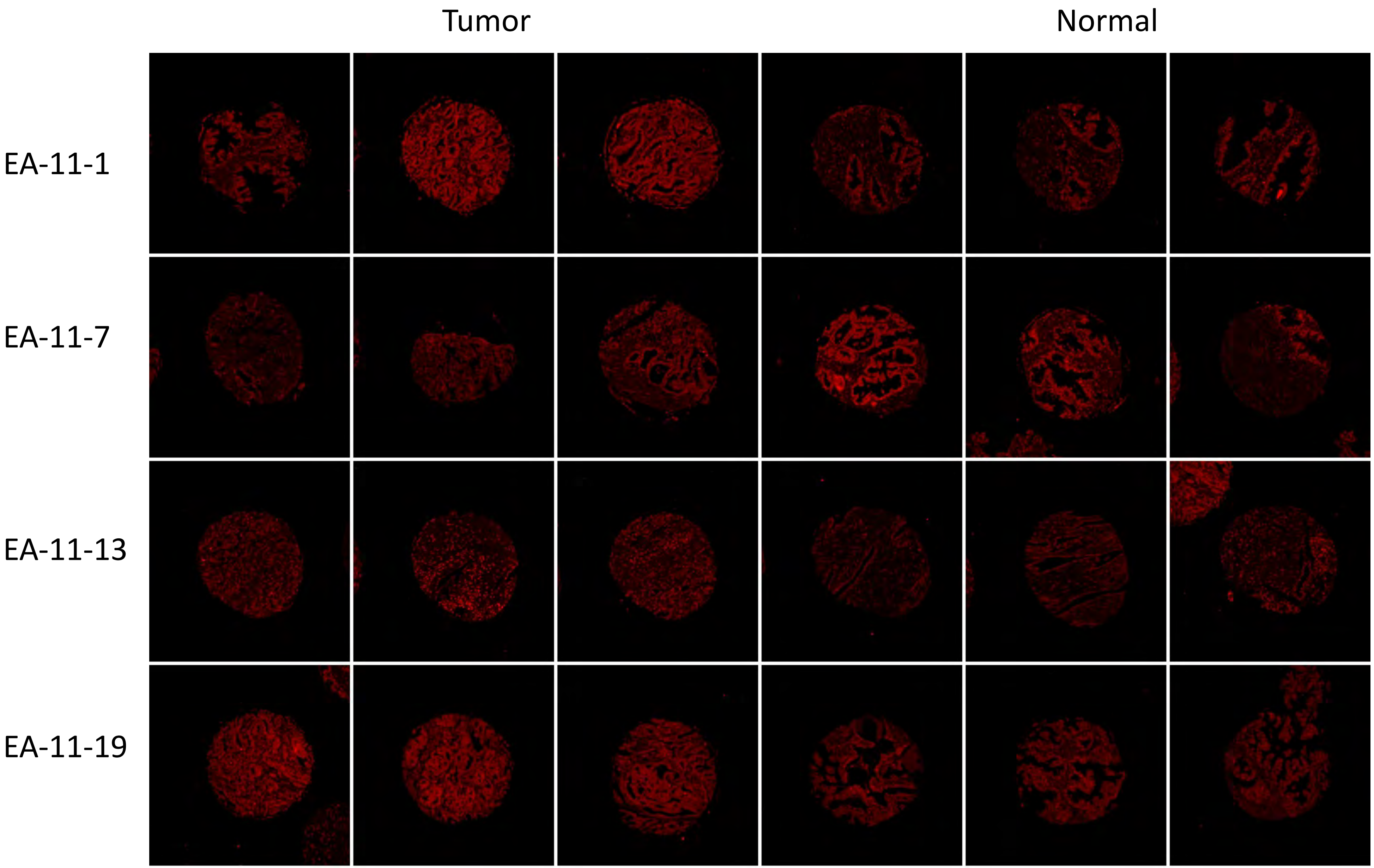

Row 11- oxRADD

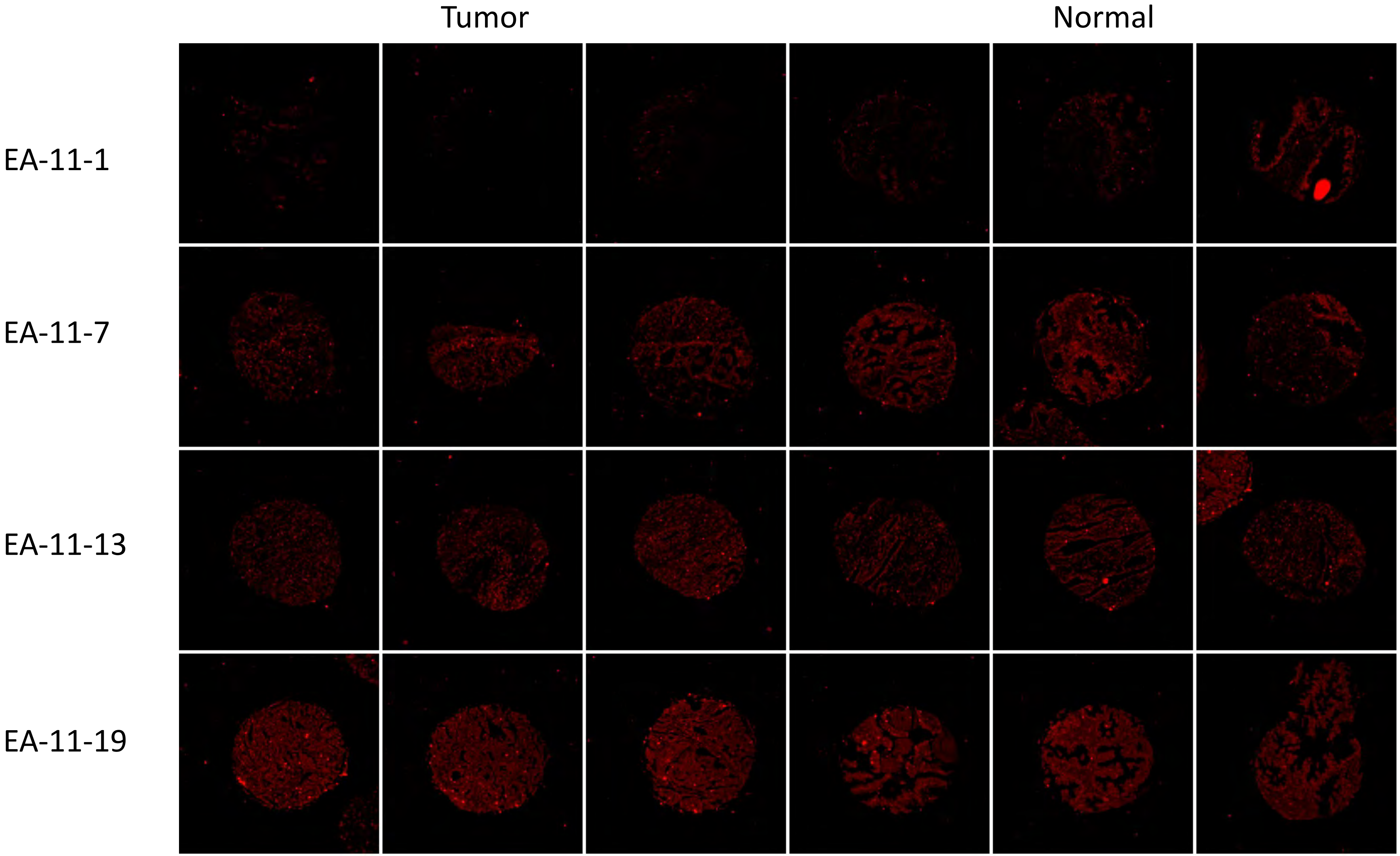

Row 11- UDG

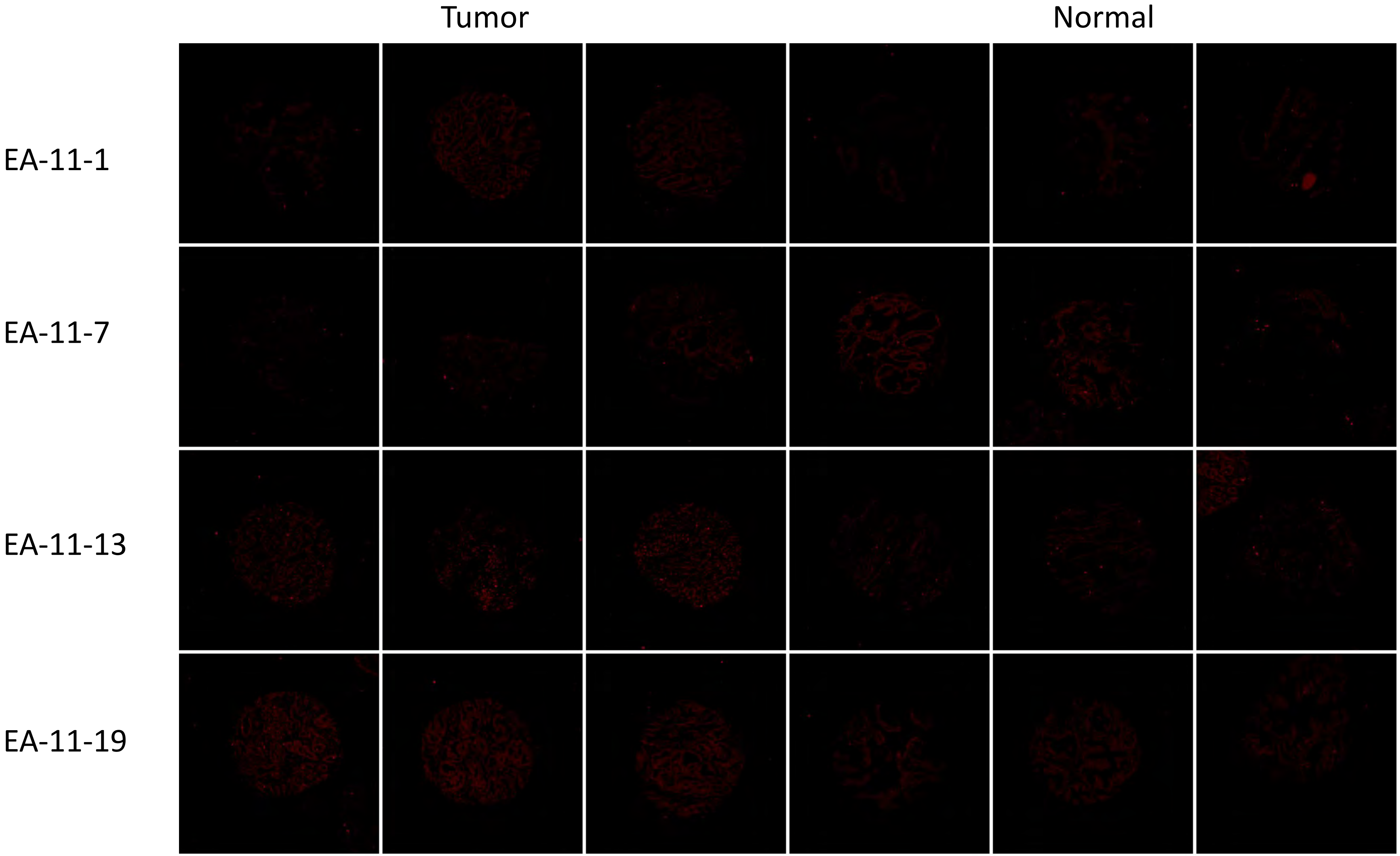

Row 11- T4PDG

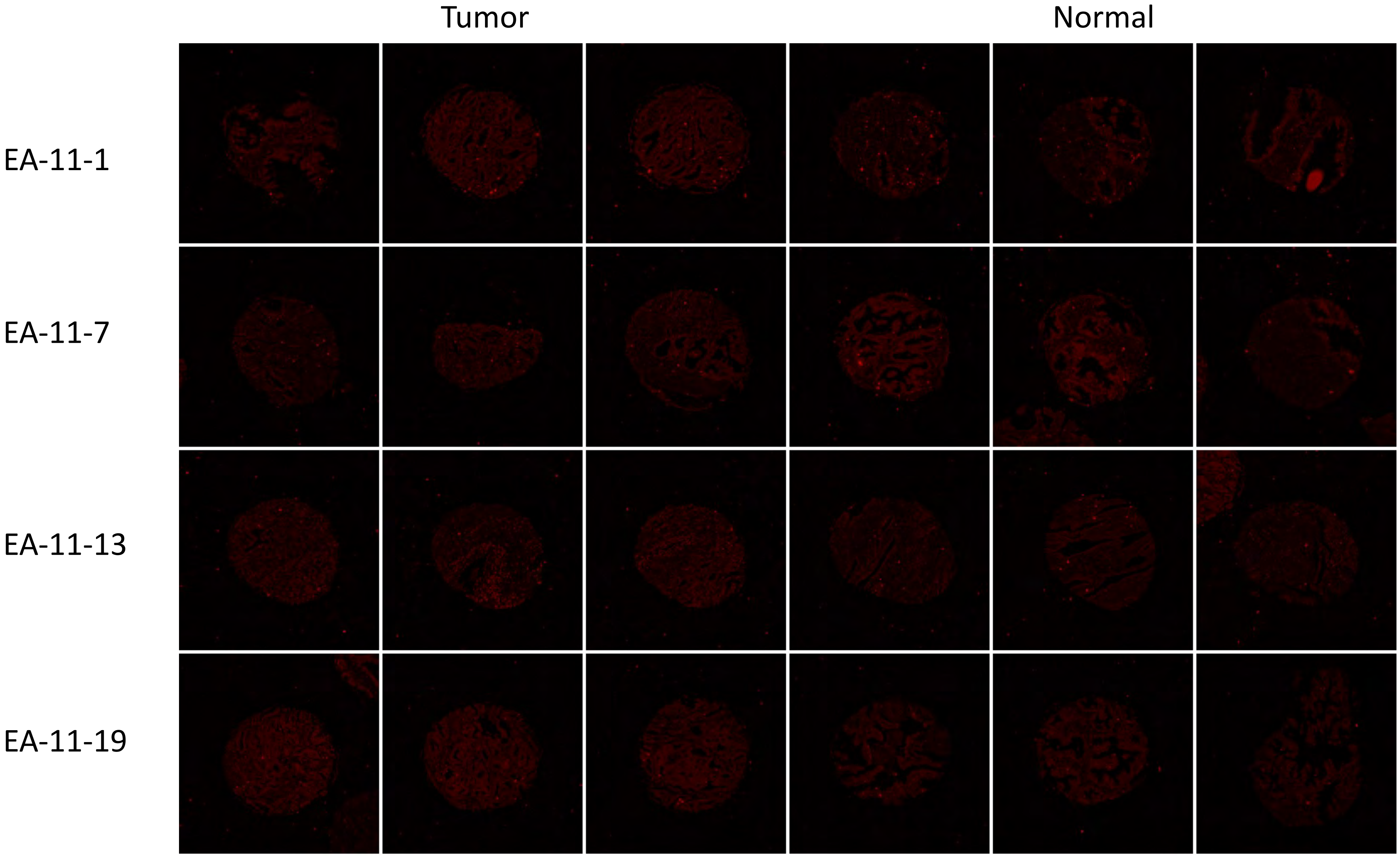

Row 11- XRCC1

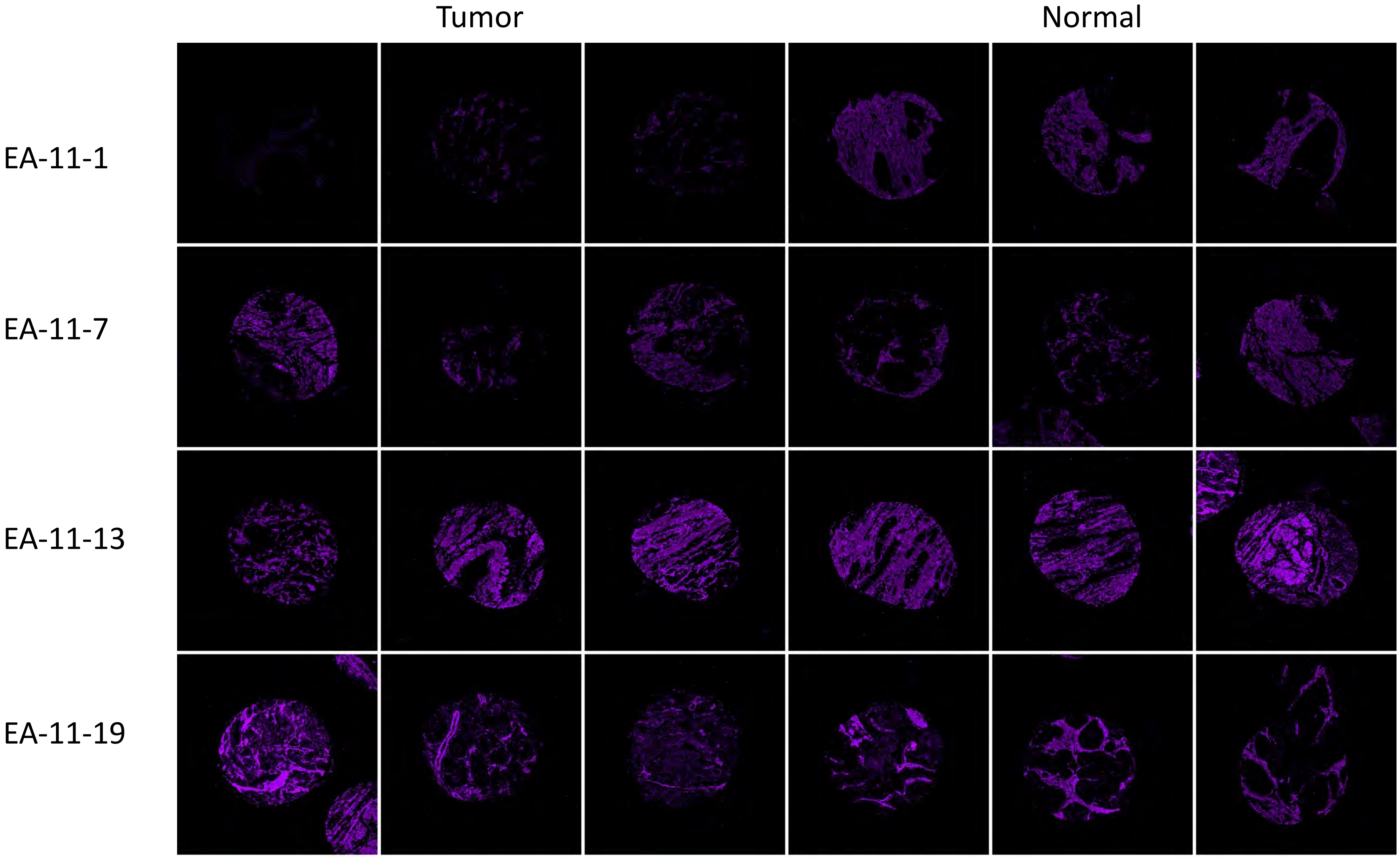

Row 11- PARP1

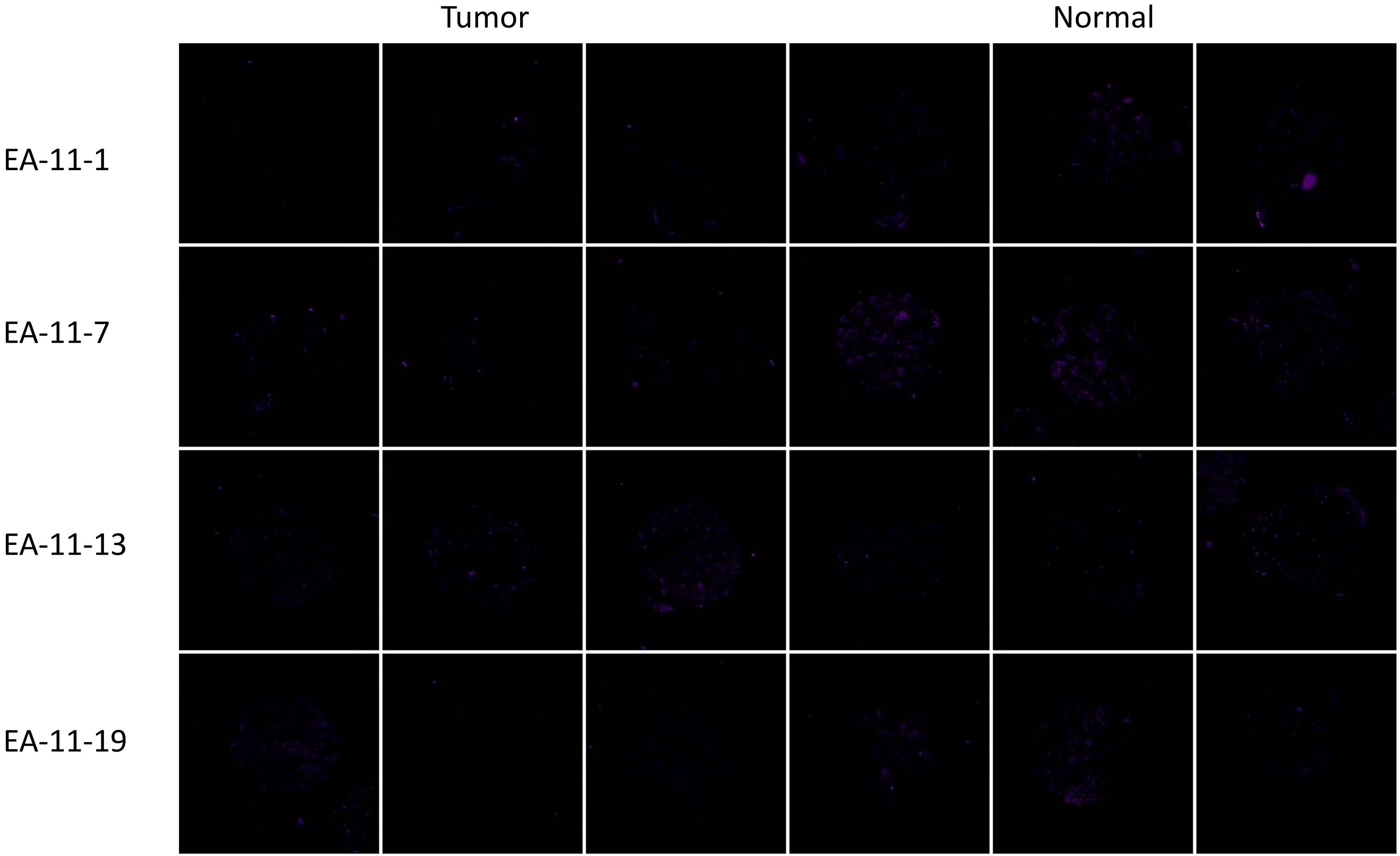

Row 11- UNG

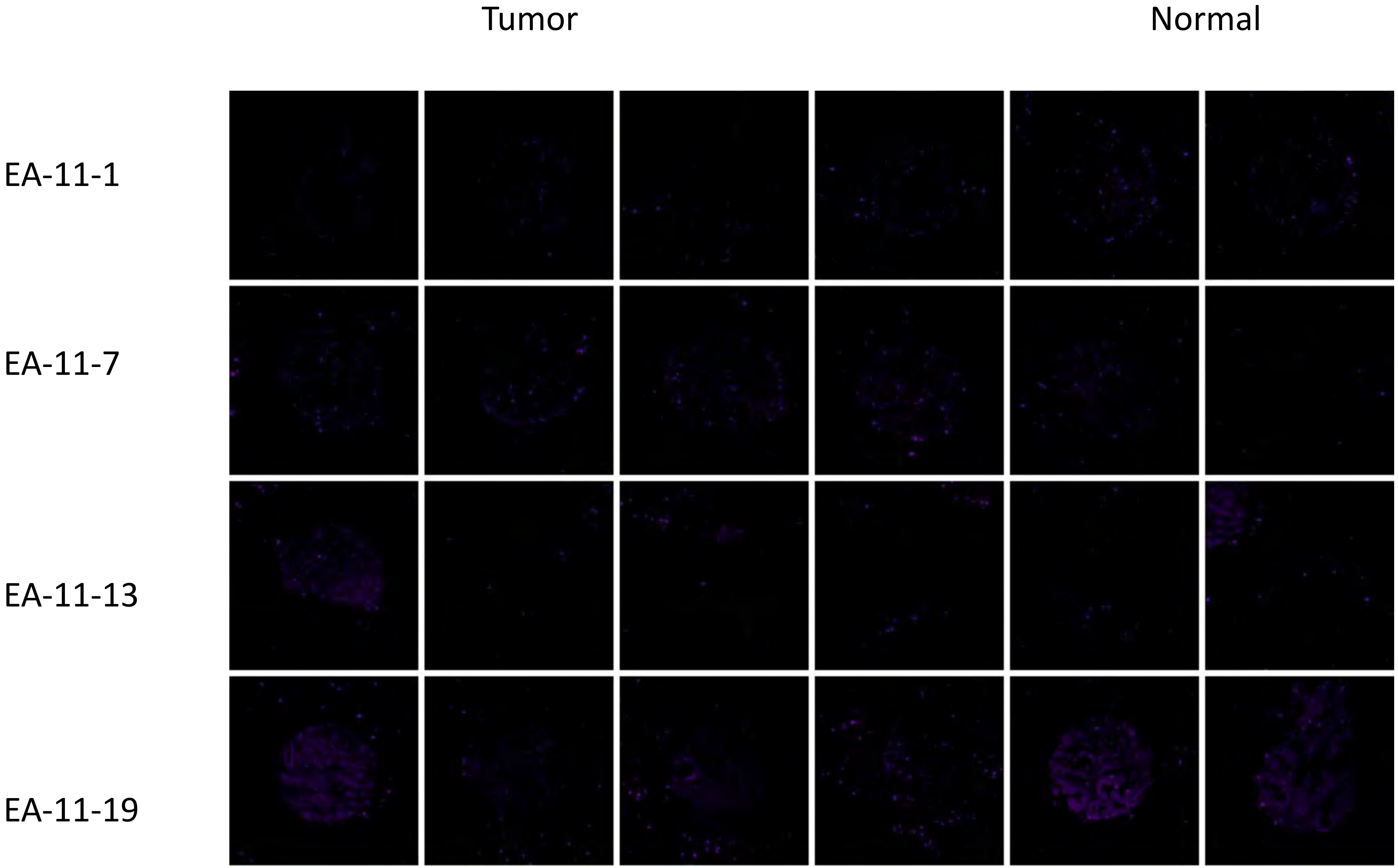

Row 12- Full RADD

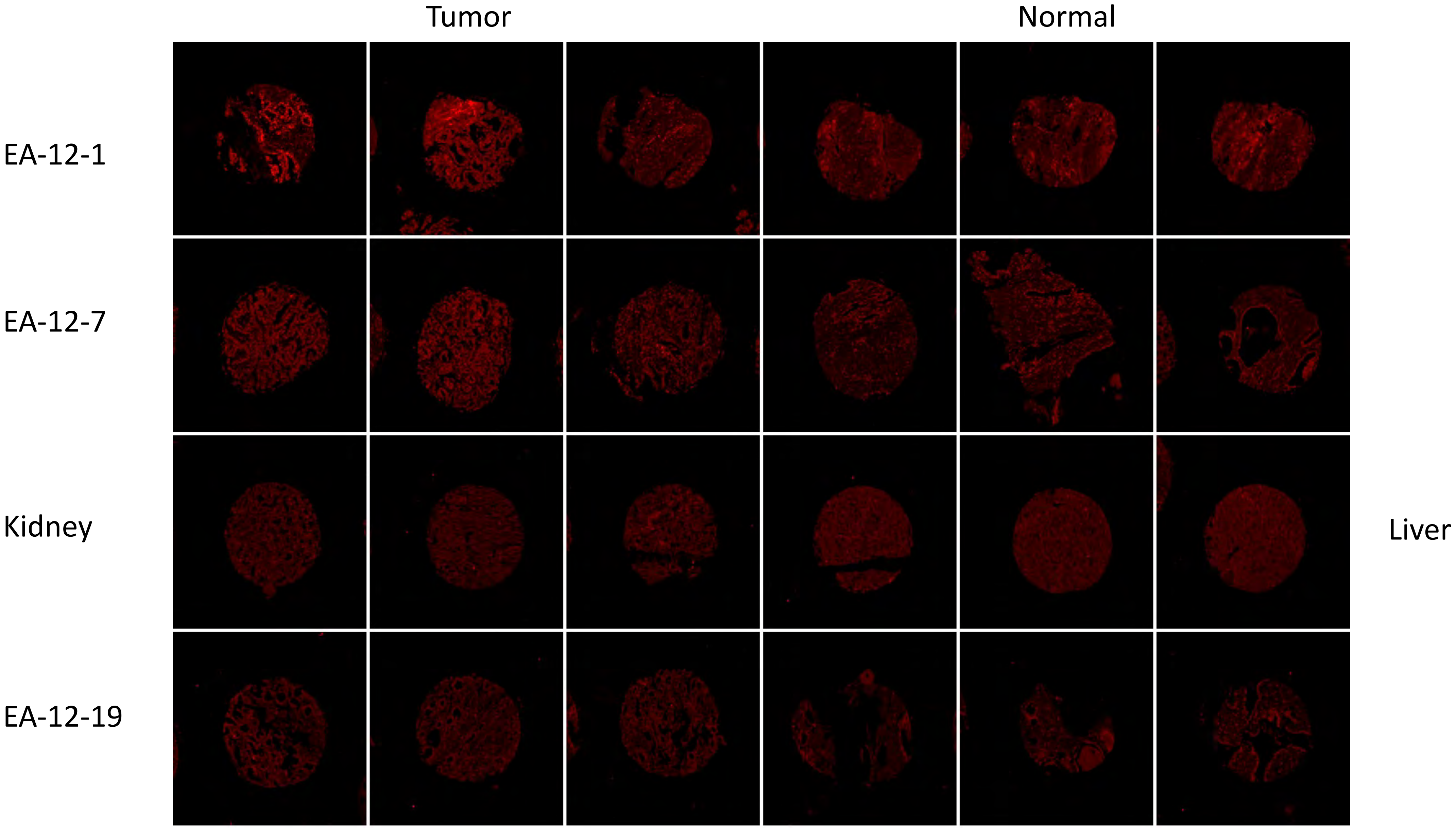

Row 12- oxRADD

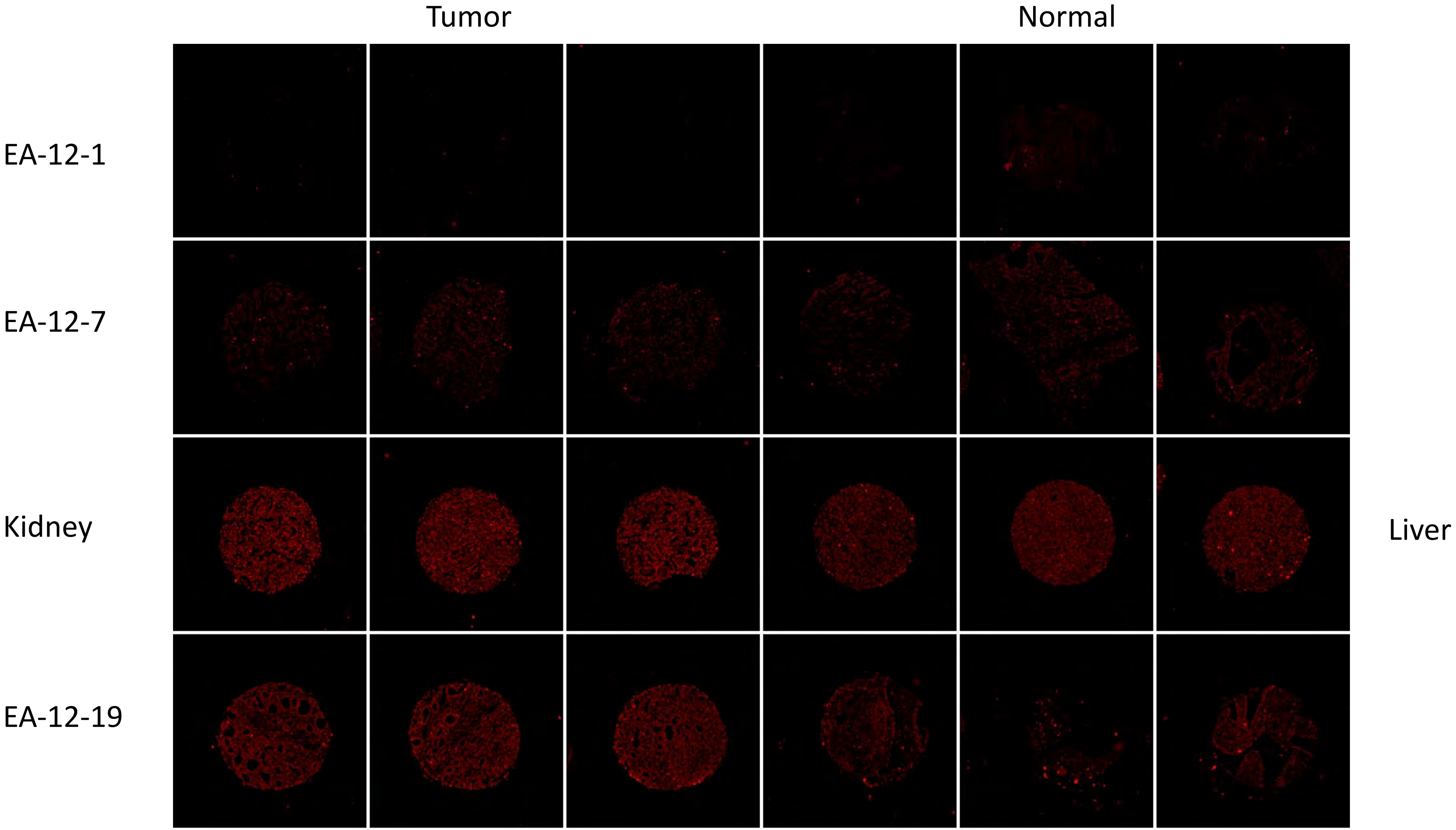

Row 12- UDG

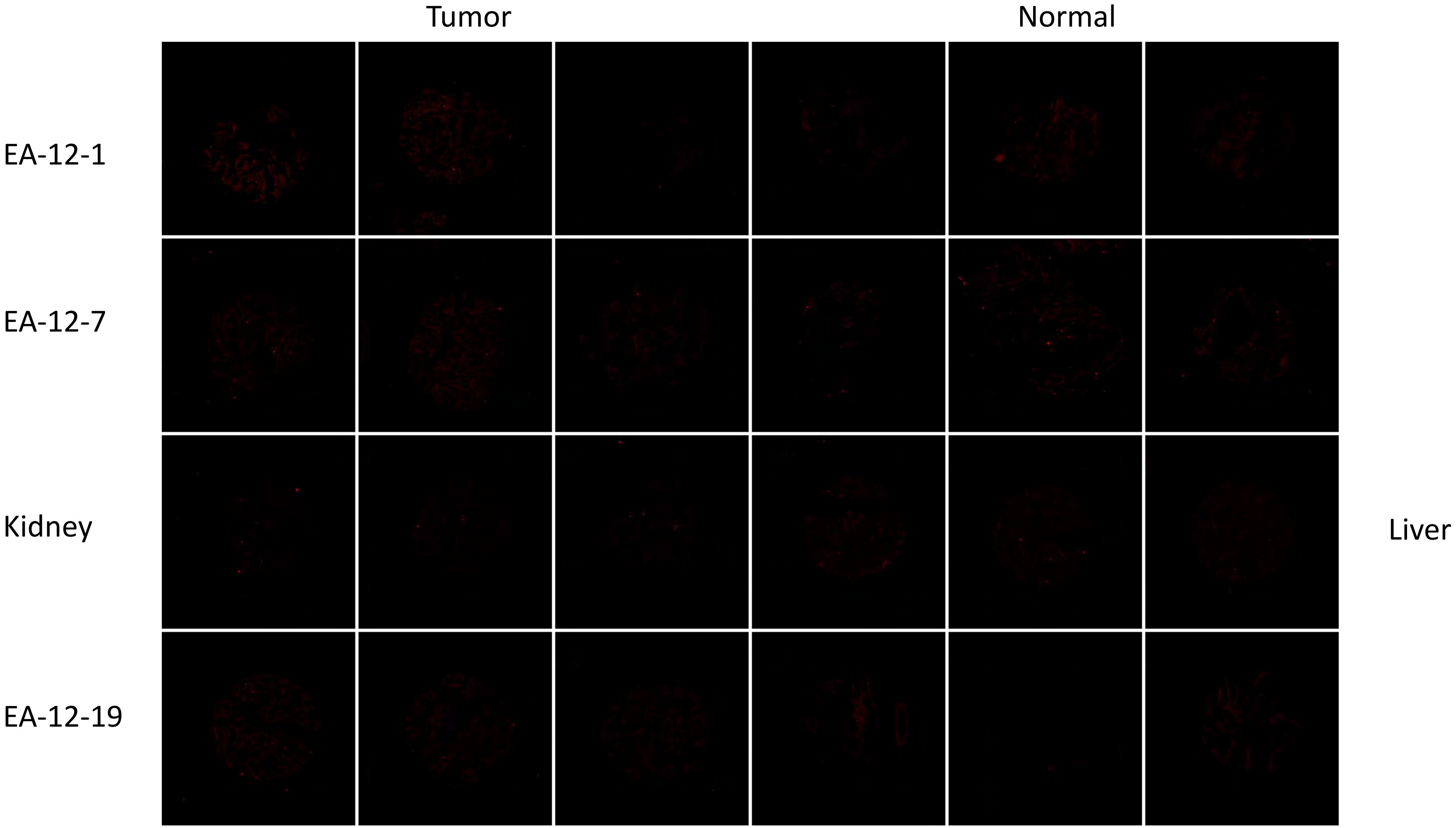

Row 12- T4PDG

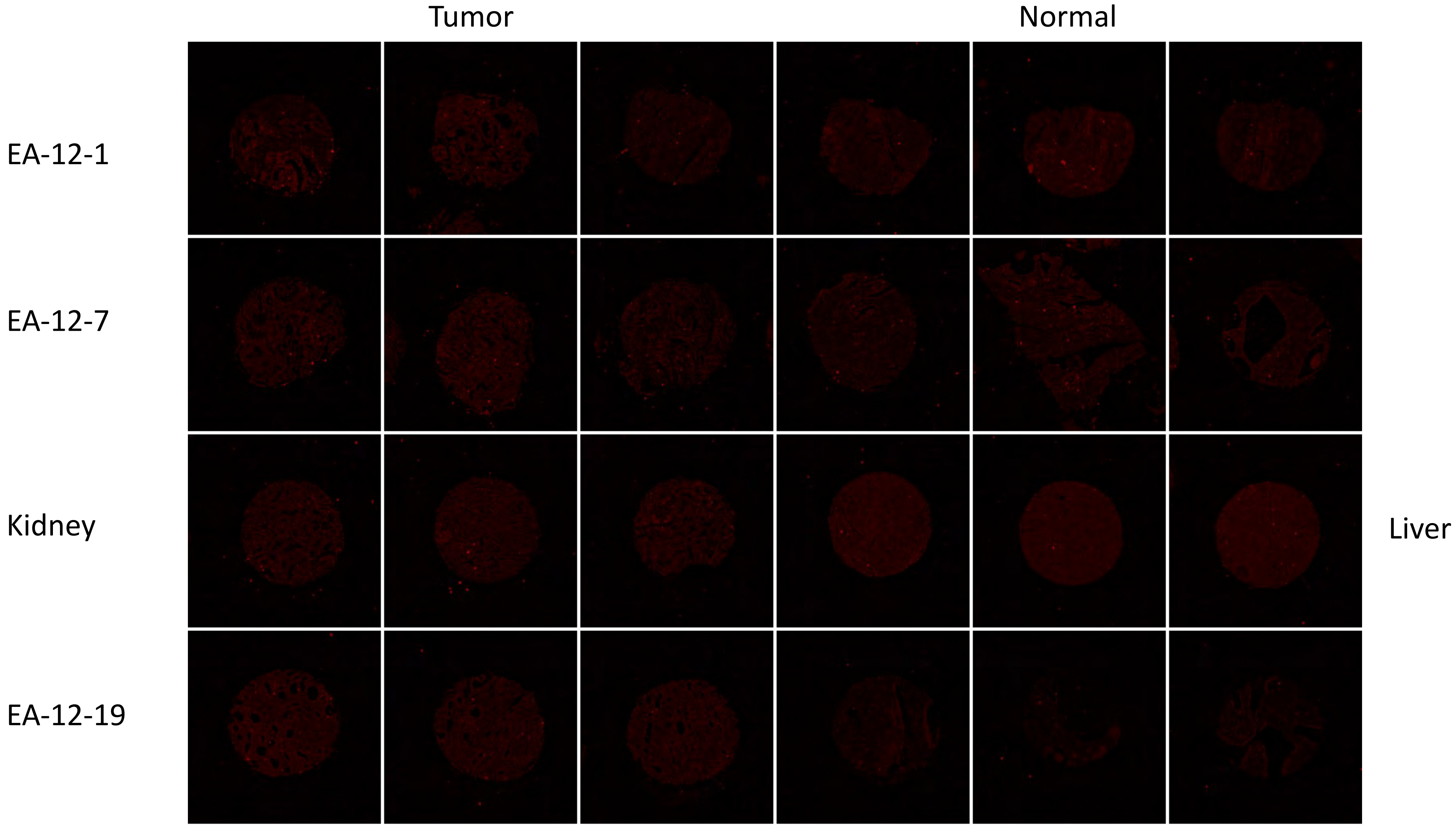

Row 12- XRCC1

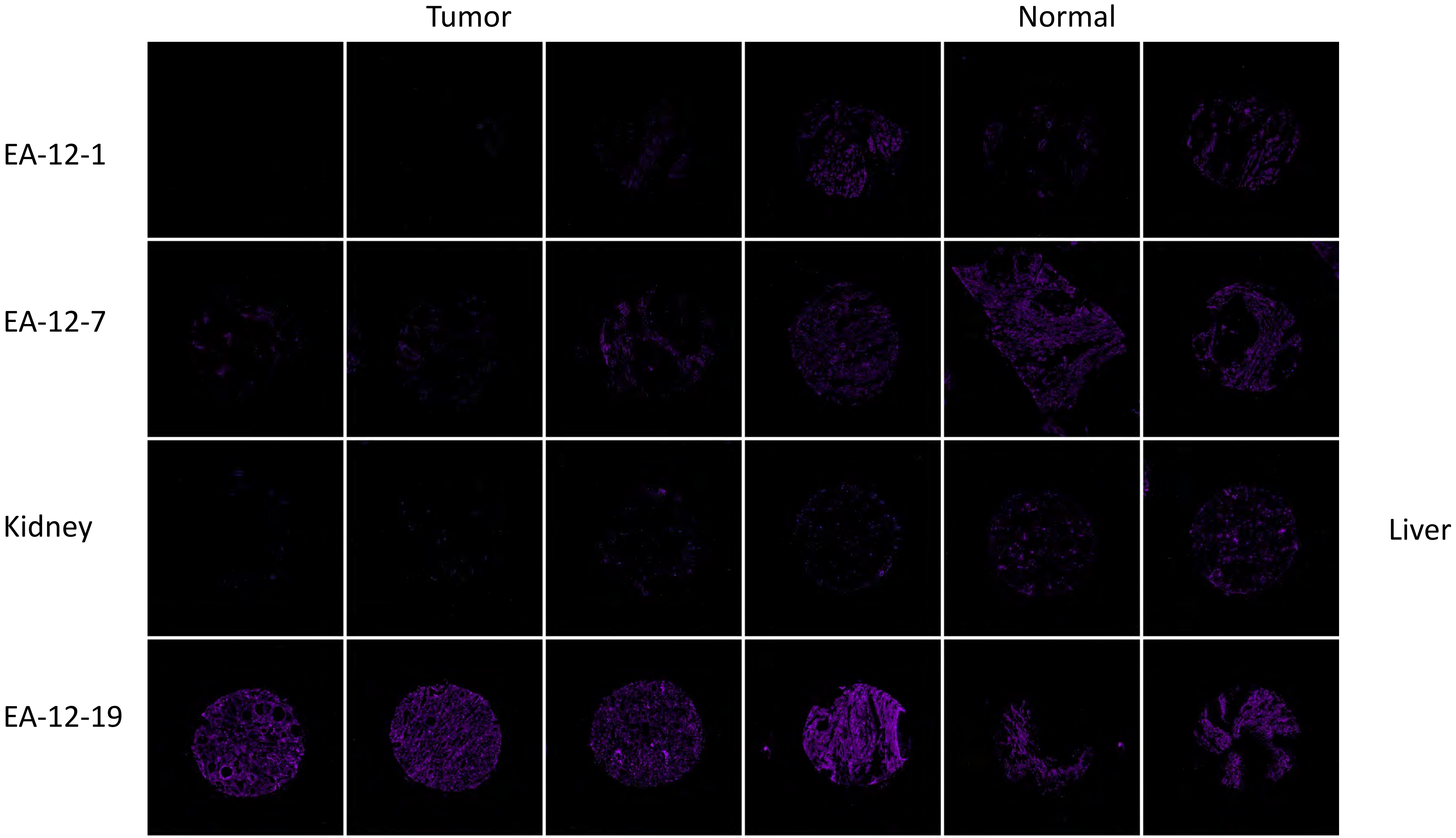

Row 12- PARP1

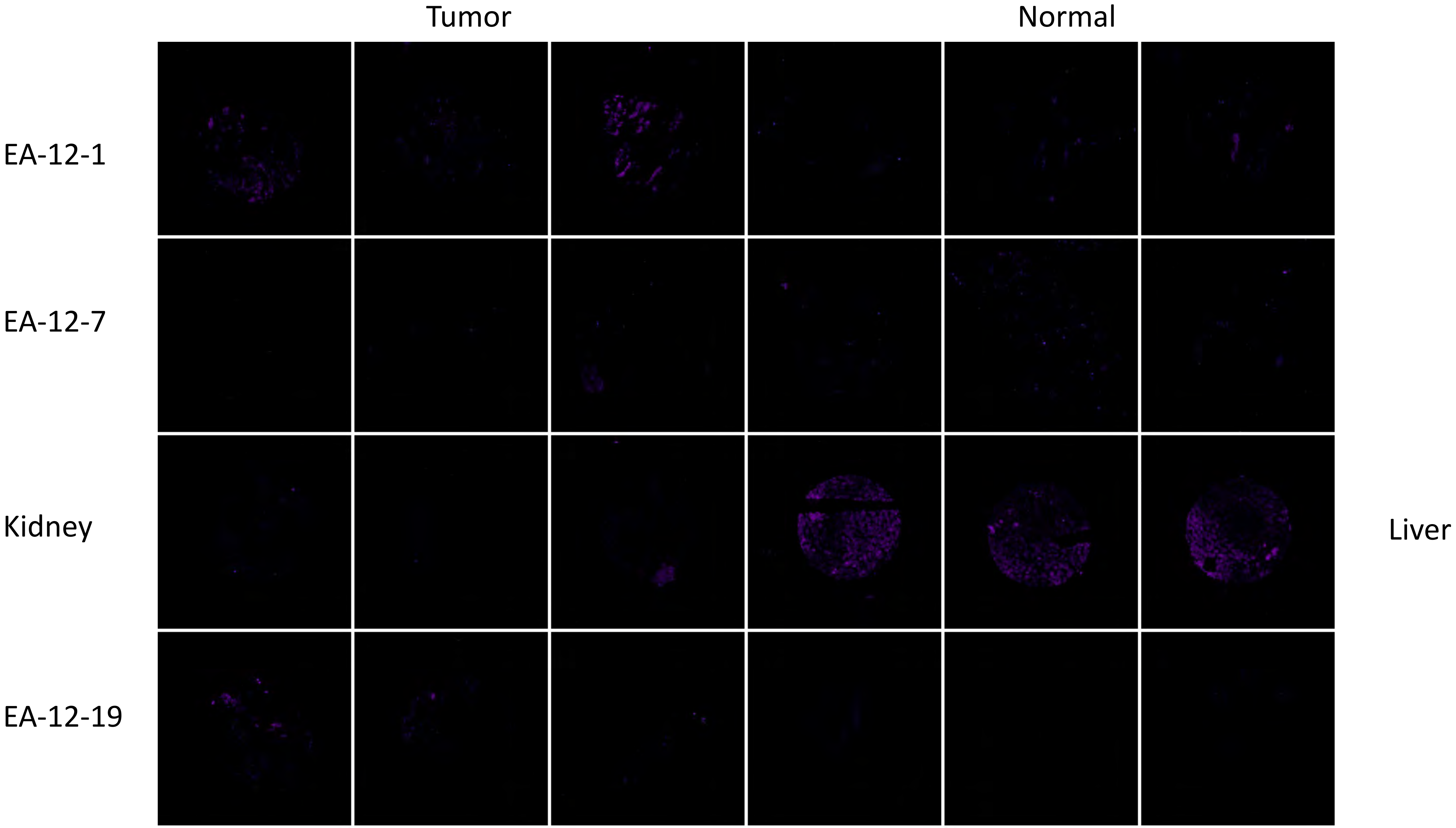

Row 12- UNG

Tumor Normal

EA-12-1

EA-12-7

Kidney

EA-12-19

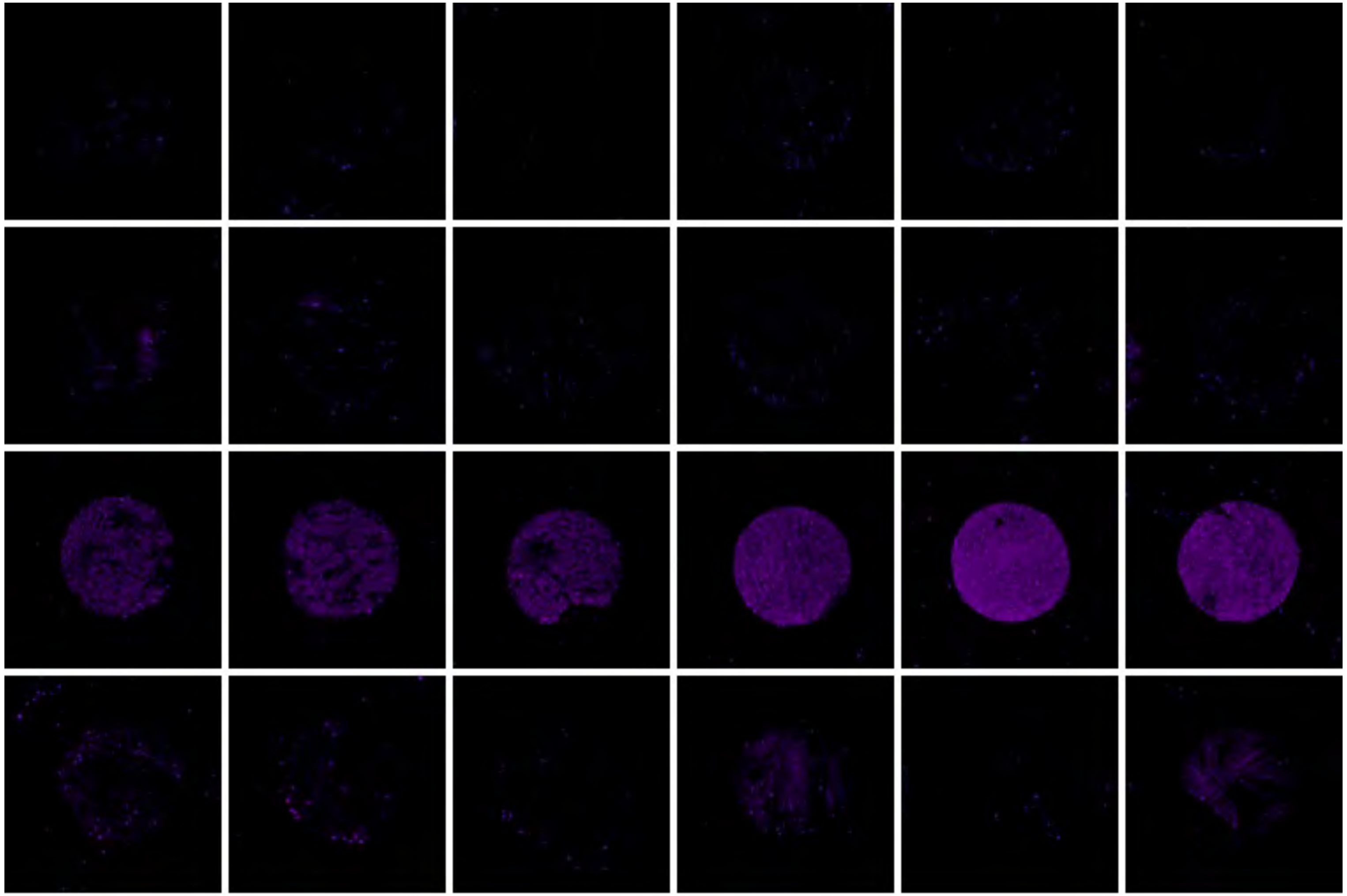

Liver

Row 13- Full RADD

Tumor Normal

EA-13-1

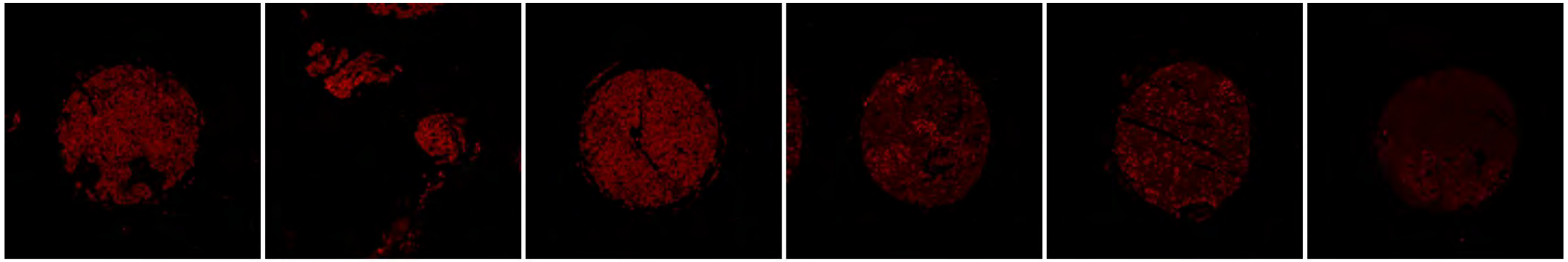

Row 13- oxRADD

Tumor Normal

EA-13-1

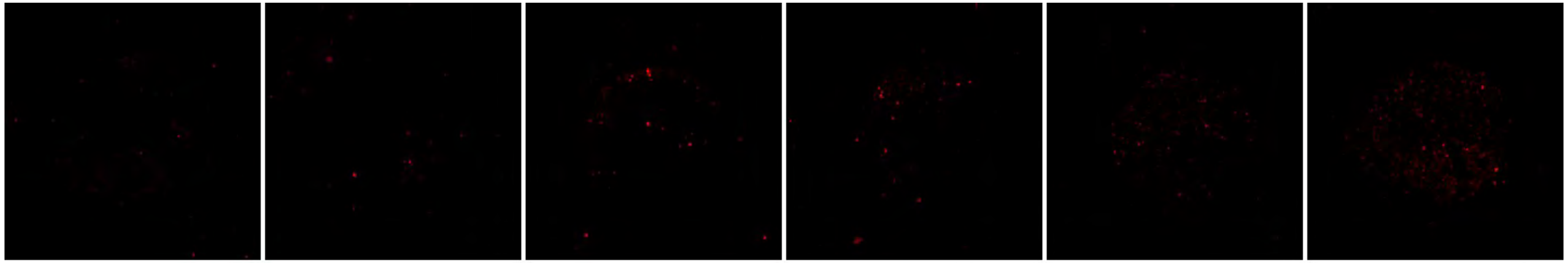

Row 13- UDG

Tumor Normal

EA-13-1

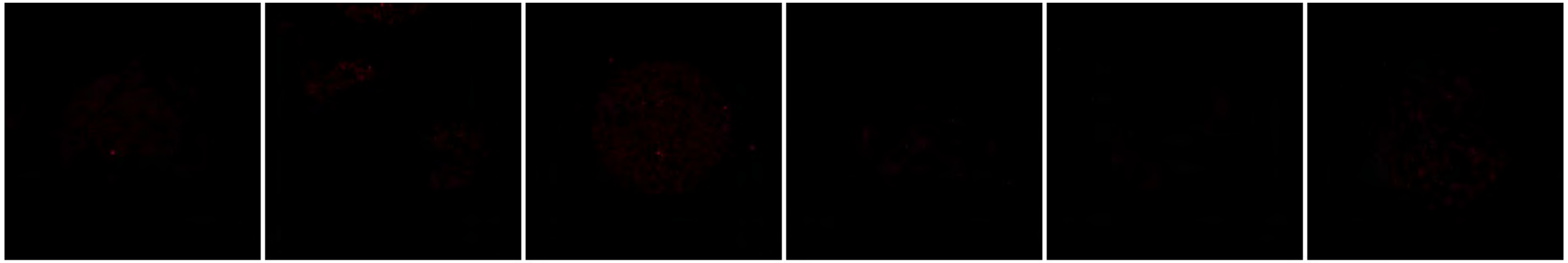

Row 13- T4PDG

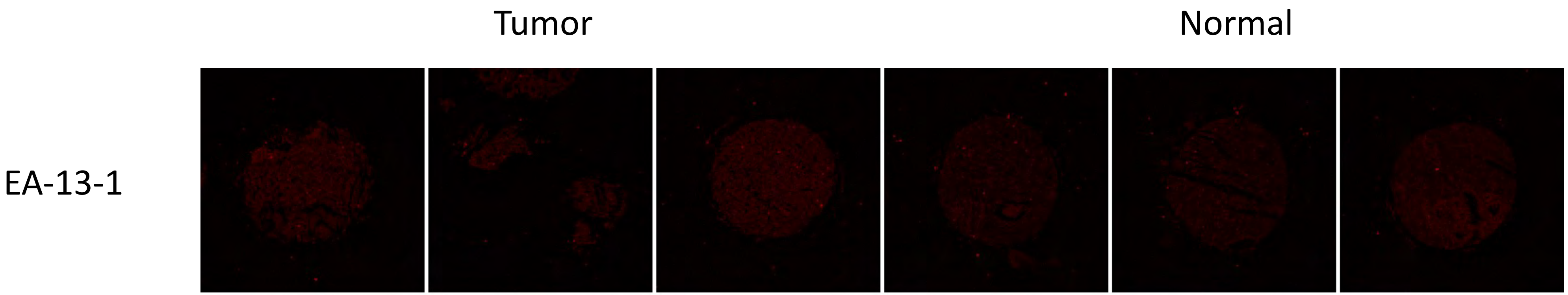

Row 13- XRCC1

Tumor

Normal

EA-13-1

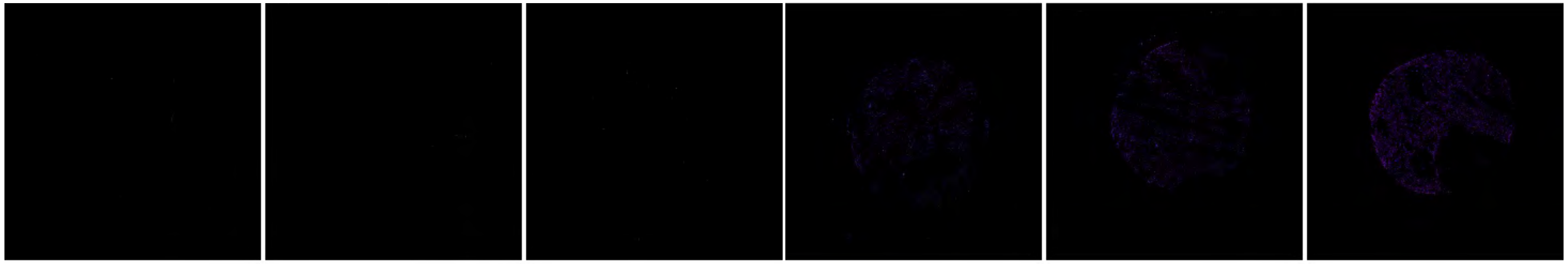

Row 13- PARP1

Tumor Normal

EA-13-1

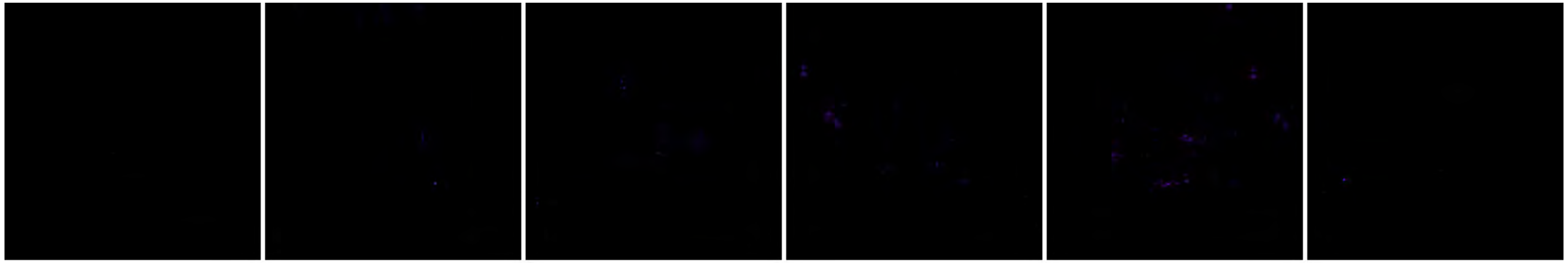

Row 13- UNG

Tumor Normal

EA-13-1

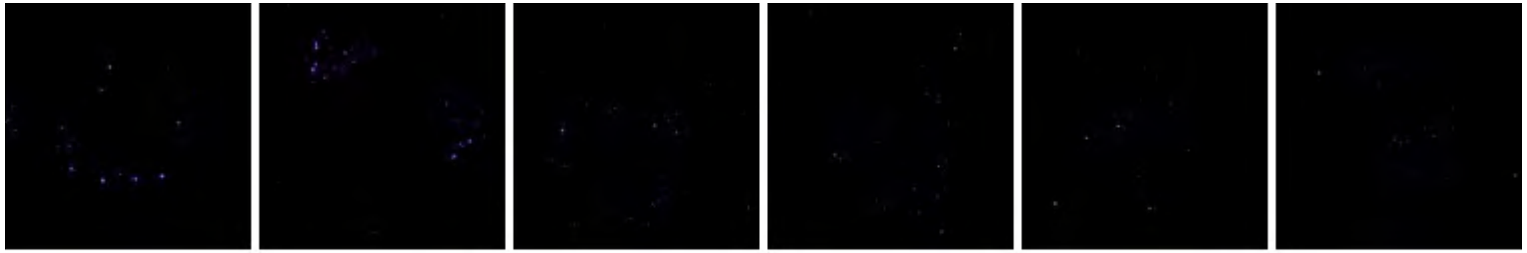

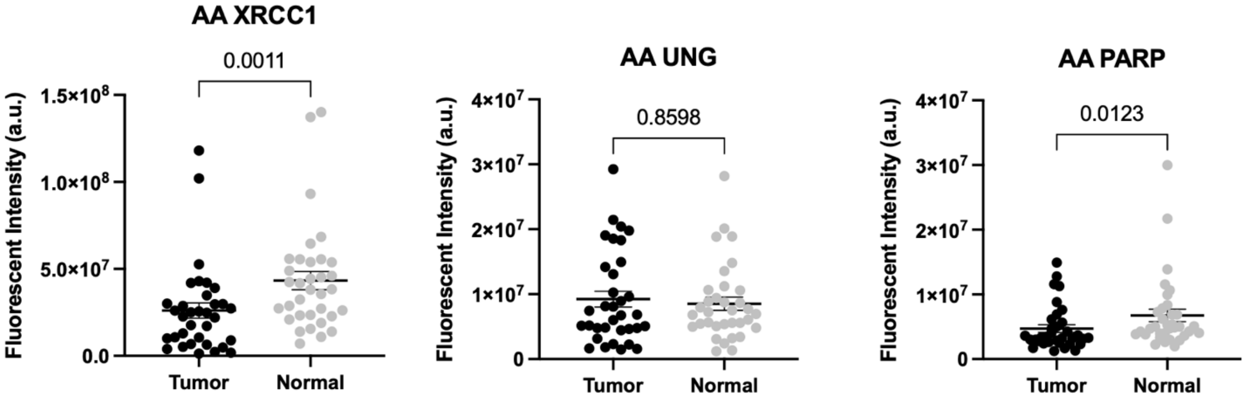

Supplemental Figure S3. African American prostate cancer tumors exhibit lower XRCC1 expression, impairing base excision repair of uracil and pyrimidine damage. Quantitative graphs measuring immunofluorescence for (**A**) XRCC1, (**B**) UNG, and (**C**) PARP1 in AA patients (n = 34). Data represented as the mean ± the standard error of the mean. The Mann-Whitney test was used to calculate statistical significance. Images for each patient are provided in **Supplemental Figure S1**.
